# Supplementary material for: Perspectives on health, illness, disease and management approaches among Baganda traditional spiritual healers in Central Uganda
Source: PLOS Glob Public Health. 2024 Sep 6;4(9):e0002453. doi: 10.1371/journal.pgph.0002453 (PMC11379289; doi:10.1371/journal.pgph.0002453)
Supplement: S1 Data — (PDF) [file pgph.0002453.s001.pdf]

Study Participant 1

Contents

Study Participant 1 1
socio-demographics 2
Mulutansozi / Abaluntansozi 3
The shrine and how it was acquired 3
Mulubaale 3
Problems associated with becoming a Mulubaale 3
Becoming a Mulubaale 4
Omukongozzi *wa Lubaale* – The Human Medium for Ancestral spirits 4
Ancestral spirits – Lubaale 5
Lubaale Kiwanuka 6
Lubaale Mukasa 7
Lubaale Musoke 7
Muwanga 7
What are the characteristics of Muwanga? (Requirements for harmonization) 7
What role does Muwanga play in healthcare management / treatment? 8
Misambwa 8
Misambwa emitonde 8
Ndawula 9
Communal meal for ancestral Misambwa (*Ekijjulo kye Misambwa emizaale)* 9
Musambwa Ddungu 9
Muzimu 10
Abalongo 11
Mayembe 11
Composition of Jembe 12
Characteristics and functions of Mayembe 12
Acquisition of healthcare knowledge. (Sources and access) 12
Health, Illness and Disease 13
Health 13
Mukisa 13
Ekyoogo 13
Problems 13
Illness (*Olumbe*) 13

Disease (*Obulwadde*) 13
Obulwadde bw'omubiri – Diseases of the physical and biological body 13
Obulwadde obw'obwongo n'ebilowoozo - Mental and psychological diseases 14
Obulwadde obw'omwoyo – Spiritual diseases 14
Places with spiritual powers 14
Natural places 14
Forests 14
Waters 15
Shrines 15
Main shrine used by Muwanga, Kawumpuli, Mukasa, Musoke, Kiwanuka, Kadduwanema, Mayembe 15
Other Shrines for: Muzimu, Bulamu, Ndawula, Ddungu, Bamweyana, Kaliisa 15
Health management 15
Causes of illness and diseases 15
Kinene 16
Plants commonly used in Health Management 16
Plants used in empowering (*Mukuwanga ensawo ya Lubaale, ebbibbo bya baloongo*) 16
Others used 17
Witchcraft 17

socio-demographics

My name is (Study Participant 1). I am a married, Muslim, 42-year-old male Muganda from Ngeye clan. I did not attend any school and I have been practicing as a Mulubaale for 20 years. I stay here at (x) village, (xx) parish-Zirobwe, (xxx) Sub-county, Bulemezi County (Saza), Luwero District. I am also a substance Farmer *(ndi mulimi era mulunzi).*

I do not belong to any traditional healer's association. However, I used to belong to Uganda N'eddagala N'obuwangwa Bwaffe, ekikulira Maama Fiina Sylvia Namutebe, because its leader knows how to handle traditional healthcare spiritualists, is fairly knowledgeable about healthcare spirituality and she acknowledges her limits within healthcare spirituality.

Ndi mulubaale era nkozesa Muwanga we kika kyange. I am a Mulubaale and I use Muwanga spirit of my clan.

My work include; (1) *nungamya empewo z'abantu. (2) ntuuza n'okutereza empewo z'abantu, (3)* I use herbs to heal and manage health conditions and concerns *(4) ntendeka Lubaale w'abantu*

Mulutansozi / Abaluntansozi

Abaluntansozi amanyi gaabwe bagaja munsozi. Abaluntasozi derive their powers for the mountains.

The shrine and how it was acquired

This shrine is a Kiggwa, *Mbuga ya Muwanga* which has been maintained over 100 years. I inherited it from my great-grand father. When my great-grand father died, my uncles, brothers to my father separated from this *Kiggwa* and made their *Sabo* shrine at Kasawo. This place was abandoned until the spirits identified me to come and restore the *Kiggwa* to functional status which it has now. Since I inherited the ancestral spirituality that had existed for many years, I was introduced into the system, had to do some rehabilitation of the things that were not going on well.

*Obulombolombo, ennono n'obuwangwa bwaffe jebuli byaddala (*culture and its norms are a reality)

Mulubaale

Nze ndi Mulubaale agobelera ennono y'enkuliiti, era fee abalubaale amanyi n'obuyinza ebijjanjaba biva mu mubujjajjaffe. I am a Mulubaale who follow issues in originality and our healing abilities are ancestral in origin.

Some people are endowed with innate natural forces and abilities, but such people may or may not be aware. Some people are aware that they are endowed with natural powers and innate forces, but do not know how to nurture these natural forces (amanyi n'*obuyinza*), so they end up suffering with physical manifestations like jiggers, bad skin.

Ability to heal as a natural gift; - *Omuvumuzi ayazalibwa nga muvumuzi aba muvumuzi, nebwayenga efuufu ewonya.* Those who were gifted from birth to be healers, are healers naturally even when they mingle dust that dust will heal.

omukono gwomusawo owekitone gweguwonya - The ability to heal is not necessarily the medicine but a healing-hand of the healer

Problems associated with becoming a Mulubaale

I had a constant severe headache since I was four (4) years old, that failed medical doctors to diagnose nor offer relieving treatment. The cause of the headache failed to be established. I was not eating, I become malnourished, stunted and very thin like a stick. My parents had separated and when my mother saw me after twelve years, she become very furious, insisted that my father was aware of my problem and unless he addressed it immediately, she was taking legal action against him. After exchange of many bitter words, my father entered the family shrine (*ekiggwa*), I do not know what he did while inside shrine, but he came out with backcloth (*Ekifundikwa)* which he made me wear. After wearing the backcloth (*ekifundikwa),* my long-lived and prolonged sickness of the headache disappeared and I become healthy again.

As I grew up, I was a very strong *"Tablic"* Muslim in transport system taking merchandize across Eastern African countries. I stubbornly refused and ignored all the indictors relating to my ancestral roles, since I thought of ancestral roles as un-religious "*Shirik - Kafirun"*. My

problems intensified. I survived car accidents many times and whatever I used to do got wrong. One time, I become unaware how I moved on foot tens of miles from Kampala to my uncle's shrine in Kasawo - Bugerere County, where I involuntarily stayed for 2 years. My wife divorced me and everything was stolen from my home, leaving me with only the cloths I was putting on. With increased pressure, I gradually submitted to the spirits desires for me to become a Mulubaale and offer healthcare services to others.

Becoming a Mulubaale

"Bikongoolo aze nomusik'awe" *nze nazalibwa nga ndi mulubaale. Jaja wa taata wange lweyafa lwenazalibwa, nga maama atembese n'olubuto okumala emyaka esatu (3).* "Bikongoolo has come with his heir" *I was born a Mulubaale (traditional healthcare spiritualists).* My mother had stayed pregnant for three (3) years, and the day my great-grandfather died was the day I was born, and immediately I was given his name, Bikongoolo**.** My great-grandfather was a known experienced traditional healer using ancestral spirits (Mulubaale). When the family gave me his name, the community postulated that I would be a Mulubaale, like the namesake.

sibuli muntu nti asobola okufuka omulubaale - it is not that anybody can become a Mulubaale.

These ancestral spirits normally choose from their genetic descendants. I was picked by my ancestral spirits (powers) to serve this role in my clan. It may follow that when I pass on, my spirit will choose from those of my children to do this work.

It was the ancestral spirit of Muwanga who possess me first and demand for servicing other *Lubaale* spirits *(Lubaale Mukasa, Lubaale Musoke and Lubaale Kiwanuka).* The family members requested to know from *Muwanga* what each of those spirits required to be done. *Muwanga spirit* replied that each of the spirits will come, possess him (me) and talk for themselves what rituals and specified animals each requires. Within a short while, they each came and specified their respective rituals and nature and colour of the animals they needed and the way the processes would follow.

The security spirits (*amayembe)* needed to be serviced (*okunywesebwa).* Each Mayembe informed us, the family members, the required rituals and the specified nature and colours of the animals to be sacrificed in the rituals, which we successfully did.

The Lubaale ancestral spirits of *Lubaale Mukasa, Lubaale Kiwanuka, Lubaale Musoke and Muwanga* also showed up and made their demands for harmonization.

Omukongozzi *wa Lubaale* – The Human Medium for Ancestral spirits

Lubaale yelondera omukongizi we - The ancestral spirits pick their human medium

Omukongozi wa Lubaale bwaffa, Lubaale yelondera omukongozzi omulala – When the spirit medium dies, the ancestral spirit picks another one.

Omukongozi asamira Lubaale na nakakasibwa kumutwe gwe, – The spirit medium goes through the process of harmonising with the ancestral spirits which process is repeated for all the newly selected and confirmed spirit medium.

Ancestral spirits – Lubaale

Spirits respect one another and cooperate during an activity

The word Lubaale means upward space (*Kulubaale, Mubaaga)*.

Spirits Kadduwanema, Mukasa, Kiwanuka and Musoke are water spirits that originated from Sese Island

Kadduwanema is the father to Mukasa and Kiwanuka. Mukasa associated most with waters while Kiwanuka associated more with the sky.

Kadduwanema is a grandfather to Musoke.

Musoke is a grandson to Kadduwanema and he spent much of his life with his grandfather who taught him to associate with both the waters and the sky. – *Musoke anywa amazi munyanja nagatwaala wa ggulu mubwengula* - - Musoke in the form of rainbow, sips water from the lake and takes up in the sky.

Lubaale Kiwanuka, Mukasa ne Musoke basibuka mu bizinga bye Sese. Ancestral spirits of Kiwanuma, Mukasa and Musoke originated from Sese Islands.

Lubaale Kiwanuka, Mukasa ne Musoke bakulu nyo eli obulamu n'embera ya Buganda, - Ancestral spirits of Kiwanuka, Mukasa and Musoke are very important in the health and life of Baganda people.

Lubaale maliba – Ancestral spirits are symbolised by the skins of the sacrificed animals and are privately secured since they are the only tangible remains of the whole process of its harmonization with its human medium

Ancestral spirits are inheritable and whatever is done to any category of the ancestral spirits is binding to the children, grandchildren and the descendants, failure of which will lead to problems and suffering within the lineage.

Spirituality has its cultural roots.

Ndi omu kubalubaale abatono abalina obusobozi n'obuyinza okutegera n'okukakanya Lubaale owenono ezenjawulo – I am one of the remaining few traditional healthcare spiritualists with powers and abilities to understand and with capacity to handle spirituality of diverse cultural origin/roots.

When harmonizing spirituality and one does not follow the cultural roots of the spirits, the harmonization process ends up being messed and frustrated.

omusingo mu Lubaale – item to stand in for, within ancestral spirits. When a family is not yet ready for the harmonisation process of Lubaale or any other demanded activity, the family or an individual may give in a sheep to stand in, until such a time when they are ready for the process.

Currently, spirituality is a very difficult process to harmonize to its original values, because; (i) harmonising spirituality is a long meticulous process that requires carefully, systematically and patiently taken and calculated steps. (ii) These days, there are very few people with the

knowledge and ability to handle the intricate of Lubaale. (iii) It is difficult these days to identify right people with quality knowledge and ability, and are willing and available to handle Lubaale. Many people pretend to be able to handle Lubaale, while others attempt and end up messing the Lubaale. (iv) Harmonizing Lubaale is a very costly process. (v) When Lubaale has been messed up during its harmonization, the process has to be repeated and corrected, this is a very frustrating and (*abantu betamwa Lubaale). (vi) yet* Lubaale is very painful when demanding for its harmonisation (*Lubaale aluma nyo nyo, era atawanya nyo nga abanja okummutereeza).*

Lubaale Kiwanuka

Lubaale Kiwanuka yasaba endiga ye mume, myufu, n'empanga ya Lujumba omumyufu - Lubaale Kiwanuka demanded for a Brown, adult Male Sheep, and an Adult Male Brown Chicken

Kiwanuka is a first acting (swifts) spirit.

Kiwanuka gwe musambwa omulaguzi omukulu mu kiggwa kino - Kiwanuka is the main diagnostic and health assessment spirit in this clan shrine. It was delegated by spirit Muwanga.

For Spirit Kiwanuka to work in healthcare properly, it must first be harmonized – *Kiwanuka okukola/okujanjaba obulungi asooka tutelezebwa*

Before Kiwanuka is harmonised, it can advise but to work properly, it must be harmonised first

To harmonise Kiwanuka, a brown adult male Chicken (lujumba omumyufu) is sacrificed.

Kiwanuka is the same spirit but work in different ways within various clans. In some clans, Kiwanuka may use herbs for healing by instructing the clients the plants to pick and drink or bathe.

Kiwanuka may in some clans work by delivering messages through dreams "*Kiwanuka aloosa*". It is through dreams that he instructs his medium or client what and how to do Kiwanuka who works through dreams normally has few words.

Spirits were utilised for different activities within various clans. The role Kiwanuka played in Mbogo clan were different from the functions Kiwanuka performed in the royal clan.

Kiwanuka akolera mu buzaale ne mu butoonzi – Kiwanuka works both with natural and ancestral forces. Kiwanuka (*mubutoonzi*) is referred to as Sselubwatuka – Kiwanuka while working using natural forces is referred to as Sselubwatuka (Thunderstorm). It is possible that Kiwanuka was a human being but acquired and worked with the powerful powers of nature

Kabaka Suuna was an arrogant and stubborn King of Buganda. One time he put in prison a healer who dared to tell him about his mistakes in the cultural norms of Baganda. The healer used the powers of spirit of Kiwanuka Sselubwatuka to send a thunderstorm that struck and shuttered king's jaw. King Suuna later understood, humbled himself, freed the healer, befriended him and appointed the healer as his special advisor.

Kiwanuka is not a healer spirit. Kiwanuka does not offer treatment to an ill and diseased person. Kiwanuka works indirectly by addressing the cause of the problems, illness or disease. However Kiwanuka may send a message through dreams to its medium or patient on what

plants to use to get better. Similary Musoke can send message through dreams to his medium or the client on that plants or rituals to do to get better.

Lubaale Mukasa

Lubaale Mukasa yansaba enume yembuzi ne ssegwanga nga byeru tuku – Lubaale Mukasa demanded both purely white adult male goat and Chicken from me

Lubaale Musoke

Lubaale Musoke yasaba embuzi ye eya luyina. Lubaale **Musok**e demanded for its goat with a patch through it middle body.

Muwanga

Nze nkongoja Muwanga omusambwa omutonde, Muwanga Ssebyoto Lukankana, Mukadde nyo nyo ddala. – I am a medium for Muwanga, a natural very old ancestral spirit called Muwanga Ssebyoto Lukankana. When he possesses his medium, he speaks very softly and slowly, using hardly audible voice of an old person. Muwanga's diagnostic tools set is referred to as *Omweso gwe ngatto za Muwanga*.

Muwanga is a Musambwa spirit with is part of Lubaale

Muwanga is a nature spirit that was created to bring light and warmth, through setting up a fire place, hence the name Muwanga Ssebyoto

What are the characteristics of Muwanga? (Requirements for harmonization)

Muwanga demanded for two (2) Brown cows; an adult male strong Bull that was slaughtered for his rituals and a female cow that was to be reared at home. *Muwanga* demanded for more things including *Omweso gwe, ogwengatto*

In fact, I used to dream of a male requesting from me his shoes. For me I thought of ordinary shoes, so I went to the shops and on many occasions bought different sets of ordinary shoes, but the dream kept persistent until I consulted our trainer - *Senkulu mu Lubaale* (*Kitaffe mu Lubaale* - *eyawanga Lubaale waffe.) who* informed me of the nature, types and nature of animal skins to use for those "shoes". So *Muwanga* spirit was called in, possessed me and talked for himself the types of animal-skins from which his diagnostic shoes are made.

Si buli Muwanga nti akozesa omweso gw'engatto - It should be noted that not every Muwanga use *omweso gw'Engatto*, the diagnostic tools.

omweso gw'Engatto za Muwanga guwangibwa mumaliba Mwenda (9) agensolo ezamanyi - The diagnostic tools set for Muwanga is made of nine (9) pieces of strong animal hides. Three (3) animals' skin pieces were got from the brown adult Bull used for his rituals. The remaining six (6) pieces of animal skins were got from Lion (*Mpologoma), Mpisi, and Entulege* wild animals.

Muwanga mwoyo ogulina obusobozi n'amanyi okwogerezeganya n'okukirizisa emyoyo emirara - **Muwanga** is a spirit with ability and power to easily communicate with and convince other Spirits

Muwanga alina Katikiro we Kawumpuli era akolera wamu n'empewo endara nga Kiwanuka z'awa emirimu nga okusinzira mubukugu bwaazo – Muwanga has his Prime Minister (Katikiro) Kiwanuka and works works closely with other spirits like Kiwanuka that he delegates work according to their respective specialities

Omusambwa Muwanga gwekenenya nyo mukulagula, okukebera, okujanjaba, n'okutangira Spirit Muwanga is very thorough in divination, diagnosis, treatment and protection.

What role does Muwanga play in healthcare management / treatment?

Nkozesa Muwanga mukujanjaba bulimulwadde na buli bulwadde - I use Muwanga spirit for most of the healthcare issues.

When Muwanga spirit establishes the root cause of the problem or illness, he identifies the most appropriate spirit to address the problem from its root cause and sets up ways to lead you to that spirit with specified instructions

Muwanga *eyaterezebwa mutendesi mulungi nyo era empewo endala zona zibukaala ntende mu ddiro lya* Muwanga -- When fully harmonised, Muwanga spirit is a good trainer, and all other ancestral spirits feel at home in Muwanga's shrine.

Muwanga *wo mu kiggwa kino yaterezebwa bulungi ate zirina obumanyirivu, kyekyo lwaki buli mpewo, nebweziba zaagana zitya wano zivaayo nezogera* – The ancestral spirit Muwanga of this shrine was completely harmonised and experienced, that is why all ancestral spirits, however stubborn, are comfortable in this shrine and always come out, possess a person and talk for themselves.

One time, there was a family that had moved to forty two (42) different shrines, but their spirits had refused to come out, however, when they reached this shrine, their spirits came out and possessed them. The spirits finally said, "Yes! this is the right place" This reached the climax when the major Phantom (Omuzimu) of their grandfather came, talked to the family and even directed and led them where his body was buried. None of the family members had ever known where their grandfather had been buried. *Omuzimu gwa Jajj'abwe azala kitaabwe gwajja negwogera. baali tebamanyi nejeyali yazikibwa. Omuzimu gwajja negubakulembera negubatwala gyeyali yazikibwa*

Misambwa

Emisambwa egimu mitonde emirala mizaale. Emisambwa nga Engo, Timba, Omugga Ssezibwa, Omugga Kasota, Omugga Mirembe. Some spirits are natural while others were delivered by human beings. Some leopards, pythons, and rivers like river Ssezibwa, river Kasota and river Mirembe were delivered by human beings.

Misambwa emitonde

Emisambwa emitonde nansangwa – Natural Misambwa existed before the human race.

The images of natural Misambwa are seen as of human beings or unique animals -

One time I had a personal experience when I was with my sisters while walking towards a water body, Ssezibwa arm, I saw a man seated on the water surface, who told us to take water

home and he then disappeared. We were very frightened and run home and hid ourselves without taking to anybody, because we knew that when you see a Musambwa, you do not talk to anybody, rest you are harmed.

Ndawula

Ndawula mulangira – Ndawula is a royal spirit and is responsible for many things including illnesses and disease that manifest and appear on the physical body. Such as Cataract, various rough skin conditions and swellings, *ensundo*.

One time in Kitale village in Kasawo, I, Bikongoolo together with some family had constructed a shrine for the Mulangira Ndawula spirit, however, the community did not like the idea of a shrine on the village, so they demolished it with support of the community people. The spirit of Ndawula possessed some people and told the community that they would pay for their sins. Jiggers and Lice invaded the whole village and community including people, domestic animals and birds until the community assembled, apologised, mobilised themselves and reconstructed the shrine that the jiggers and lice stopped in the village.

Ndawula may, at times require a black goat for his rituals

Communal meal for ancestral Misambwa (*Ekijjulo kye Misambwa emizaale)*

Other Misambwa also came and made their demands which were given and rituals done.

I remember one time, in my childhood, I experienced one *Misambwa, Mulongo Waswa Sezzibwa* which demanded me for specified rituals which were done.

When I was an adult, *Musambwa Ssezibwa exponsed its self to me, reminded me of our encounter in my childhood and* demanded for a communal meal (*Ekijjulo)* from me. Musambwa Ssezibwa instructed me to prepare a communal meal (*ekijjulo) at its river source in Kyaggwe. The communial meal was to be made of* well roasted meat and roasted banana fingers for a bigger number of people. The spirits promised to prepare the people who will eat that meal.

Assisted by the family members, we prepared the requirements as instructed, set off to the source of River Sezzibwa in Kyaggwe, and prepared plenty of roasted meat and banana fingers. There were no people at the source of river Sezzibwa as we started the roasting of the meat and banana fingers.

When everything was well prepared, we were surprised to see a fleet of vehicles with over forty (40) tourists including whites, coming to the place, they joined us, and we served them the meal. They all eat and were very happy and grateful for the meal.

Musambwa Ddungu

Musambwa Ddungu made its demands through shared dreams. Every morning we used to have time to say our dreams within the family members, or the trainees. The dreams from relating to Musambwa Ddungu is normally similar by most members. My dreams were similar to the dreams of other members. When we shared our dreams with the family members, the dreams made sense of what should be done to for Musambwa *Ddungu*

Muzimu

Muzimu gwe gunanyini Lubaale – Muzimu is the owner of Lubaale. Muzimu is not Lubaale and Muzimu is not part of Lubaale.

Muzimu ogwasoka okuwangibwako lubaale mu Kika gwe gutambulirwako ennono ye nkuliiti - The original Muzimu to perfects its Lubaale in the clan is the one whose original functional and cultural details are followed which are referred to as *"ennono ye nkuliiti*".

E Mizimu nga bwejijja gidiringana mu kutambuza Lubaale, bengi bigenda bikyuuka mu nteleeza ya Lubaala ne nnono w'ayo. – As Mizimu follow each other in harmonizing Lubaale, many cultural differences occur in the process as contrasted to the original Muzimu and its cultural process. So it is advisable to trace the original Muzimu and its Lubaale hence "*ennono ye nkuliiti".*

Bikongoolo-Tebitta-Nume gwe Muzimu omukulu gwenkongojja -Muzimu gwa Jaja wa ba Jajange. Taata wange aguyita Jaja we - Bikongoolo-Tebitta-Nume is the Major Muzimu spirit for which I am a medium, it is an old ancestral spirit of my great-great grandfather.

My father died when he was 87 years yet, my elder brothers are above 60 years, but none of them saw our great-grand-father while still alive.

Silya mere omuzimu bweguba nga gwegunalagula n'okujanjaba – I do not eat food the day the Muzimu will be on duty for health management

Omuzimu gwe gutambulirako ensonga za Lubaale yena mu lugya – Muzimu is the spirit that controls all the activities of all the ancestral spirits of the family.

Omuzimu kidundu ku muntu – Muzimu is part of human. Muzimu can

Muzimu/Mwoyo is part of our living body. Muzimu has the ability to separate from human body and stay or work on its own especially during sleep or upon death. *Okufa ekikutuko* is when the muzimu leaves the body and fails to come back to the body in time.

There are forces in nature that can control over the Muzimu or make it a captive.

Mwoyo is when the spirit is in the living body.

Muzimu is when the spirit has separated from the body, either when one is sleeping or when one is dead.

The Muzimu of a dead person may possess a living person of its choice and give guidance regarding its possessions, roles, property, and how different things or abilities were gathered, empowered, protected and how possessions they may best be utilised.

Omuzimu gutera okugoberela emirimu, obuvunanyizibwa n'enkola yaba Jjaaja bagwo - The *Muzimu* normally follows the ancestral function, roles and responsibilities.

If one's ancestry had a healthcare functional role, the Mizimu will follow suit. The Muzimu that used to offer healing services will give clear instructions regarding the materials and non-materials, to use for healing specified health and life conditions to the medium through the deities Misambwa and Mayembe

Muzimu is the one that instructs the other ancestral spirits Kiwanuka, Mukasa, Musoke, Jembe and Misanbwa what healthcare to offer. Muzimu sets the direction for healthcare function

In Buganda, *Muzimu gwa Kintu* is the most knowledgeable spirit regarding *ennono ya Baganda*

Abalongo

Nina emigogo gy'Abalongo – I have sets of twin forces. These were the second spirits to possess me after the Muzimu. The twin forces demanded to be rehabilitated and rejuvenated since their regalia had been spoilt by rats.

A woman can give birth to various type-set of twins.

A woman can give birth to twins where both are human beings (Male-Male; Male-Female; Female-Female).

A woman can give birth to twins where one is human and another is not human but a living thing like an animal, reptile or any other living entity

A woman can give birth to twins where one of the twins is a human being and the second twin as non-living thing like lake waters like Lake Nalubaale), river water like river Mayanja

A woman can give birth to twins where both twins are non-human

A woman can give birth to twins a child being born comes out of the birth canal with legs first, a twin called *Kasowole*.

A woman can give birth to twins where a new child baby comes with two umbilical codes, is a twin. All these sets of twins have specified cultural rituals that must be performed onto them at specified periods of their lives

In such cases, the human twin has special connection with the other twin entity, which twin has special energy forms, powers or abilities that can be tapped into by gifted or knowledgeable individuals. These other entity twins may transform into Misambwa and at times visit its human twin.

My grandfather knew how to cool down very powerful animals like lions by placing some medicine in the footprints where the animal has stepped.

One time, Lions invaded the villages in Kasawo and destroyed people's property and domestic animals, when my father, was called upon to help. My grandfather placed some medicine in the footsteps of the lions and soon all the lions were able to be killed at a place called Kagere.

Mayembe

Jjembe *liba mugatte gwaddagala, likumibwa munyumba empangaazi, elikuwebwa omulubaale nolitwala okukola ekyo gwe kyolitwalidde. Eddagala elikuweredwa*– Jjembe is a combination of materials, placed in a durable container, given by a spiritualist to perform the specified tasks. The durable container for the jembe is normally an animal horn.

Ejjembe liwangibwa n'eliganbibwa emirimu n'obuvunanyizibwa ebiritumiddwa okukola – The Jembe is empowered and given instructions of its roles and responsibilities

Bwemba mpanga ejjembe, ndiwandako eddusu, ndubulirira, nediwa obuvunanyizibwa n'emirimu gyeririna okukola – When I am making and empowering e Jjembe, I use my saliva to empower it, counsel it and give it instructions regarding its responsibilities and roles

Olulimi lutonzi, ejjembe kyoligamba kyelikola – The tongue has creative powers, what you tell the Jembe to do is what it will always do.

Amayembe gakolerwa emikolo omuli n'okusaddaka buli banga gere, nga buli mwaka, okugaza obujja n'okugongera amanyi munkola yago – periodical rituals involving sacrifice, usually annually are done for the Mayembe to renew and rejuvenate the powers and abilities to perform

Ejjembe lyange linywesebwa bulu mwaka, okulidabulula mumanyi n'enkola yaalyo – Annually, the powers and abilities of my Jembe are rejuvenated by the blood of animal and chicken sacrifice.

Composition of Jembe

Jembe Namuzinda lyalungibwa Munyera, Enumba, Kabaka we Nsanafu, Queen we Njuki, Queen we Nsanafu, Kabaka w'eMiti (Enzirugaze) nebirara ebyekyama bingi. Lyasalirwa embuzi lyejasabwa, ebiliwa amanyi gaalyo n'okukola emirimu jelitumibwa – Jembe Namuzinda is composed of many Kings and Queens of insects, plants, animal sacrifice any other secrets that give it powers and abilities to perform its instructed duties.

However, if one accidentally cuts himself and blood comes out, while constituting the Jembe, the whole process is terminated immediately, because human blood should not be part of the Jembe process. This is because, in case human blood gets into the Jembe, then that Jembe will always demand human blood and hence human sacrifice.

Characteristics and functions of Mayembe

Nina amayembe ana (4) nga gakola emirimu janjawulo – I have four (4) Mayembe and they different functions.

1. Jembe ***Lugabo*** - to remove any bad spirits from within the body on an individual,
2. ***Jembe Lubowa*** - to protect home from anything bad,
3. Jembe ***Namuzinda*** - to look for money.
4. Jembe ***Kilarile*** - keeping and caring for domestic animals

Acquisition of healthcare knowledge. (Sources and access)

Diversity in knowledge by the traditional healthcare spiritualist largely depend on their trainer's ability, knowledge and availability of time to explain in details to the trainees.

I believe in the powers of the dreams;

Nzirririza mububaka bwenfunira mubiroto I believe in the messages contained in my dreams,

While in a dream I was given and taught a prayer and in the morning the prayer was very clear to me. The prayer (*Saala ya mutonzi),* calls upon one's ancestral spirits, *Misambwa, Lubaale, Mayembe, Mizimu, Balongo,* for protection and indeed the spirits hear and protect

Health, Illness and Disease

Illness and disease are the same*: Ekintu ekikosa omuntu kumubirigwe*

Health

Omuntu omulamu ye muntu atalina bukosefu bwonna

water spirits Kadduwanema, Kiwanuka, Mukasa and Musoke use the word Gayira-gayira

Bweeza is a word used for good health, harmony, good luck and in all aspects that accept the situation as positive. The word Bweeza is used for and by all ancestral spirits to refer to Lubaale Mukasa. Every clan in Buganda has the spirit Mukasa.

Bweeza refers to blessings and Mukasa spirit is known and acknowledge for blessings. (*Bweeza bwa Mukasa*)

Mukisa (Good Luck)

Eddagala lyo Mukisa: Muswaki gwo Mukisa

Ekyoogo

Ekyoogo

Kyoogo is used for; Cleansing. Kyoogo is used first before most spiritual activities. The content of kyoogo depends on its purpose or the ritual to be undertaken. Timing for use of kyoogo is significant (time of the day, especially at day break, midday, evening or night). Water used for mixture is specified at times (sea, lake, river, spring, swamp, rain, *mukoka*, plant exudates, *ntonyeze*, etc.)

Problems

Ebizibu bisobola okugibwaawo n'omugatte gwemiti nga eddagala

Illness (*Olumbe*)

Disease (*Obulwadde*)

Obulwadde bw'omubiri – Diseases of the physical and biological body

Obugumba busobola okuletebwa eddogo. – Infertility may be caused by witchcraft.

Obugumba busobola okugibw'awo n'omugatte gw'amanyi gebimira - infertility can be removed by combinations of medicinal plants imbued with spiritual energy and power.

okujanjaba obugumba obuletebwa eddogo, empewo za bajjajange enkugu mukujjanjaba obugumba zindagirirwa ekyokukola nebimera byebanyambyeko okunoga n'okuyenga nebinaaza omulwadde n'okunywako nanywako. Mumyezi nga ebiri afuna olubuto lwomwana. – I manage infertility due to witchcraft by following guidance from my ancestral spirits, specialised in management of infertility, to pick medicinal plants that I mix with water into a concoction that client bathe and drink. Within two months the clients become pregnant.

Obulwadde obw'obwongo n'ebilowoozo - Mental and psychological diseases

Omuntu bwanyomola empewo omuddiringanwa, empewo zisobola omubonereza n'okukuswazaswaza nga zimukwata nezimukosesa ebyambyoone, nga okwekakatika kunsolo oba okusobya kumwana owemyaka ebiri, olwo nezimwamukako amateeka gensi negamuvunaana. Olusi empowo zimufulira ddaga omulalu. - When a person continuously abuses the spirits, the spirits may punish him by possessing him, controls his activities and take him to have *sex* with an animal or a child as young as two years after which the spirits leave him embarrassed in the public and the state laws takes over. It may instead make a person a lunatic.

Obulwadde obw'omwoyo – Spiritual diseases

Obulwadde obw'omwoyo; Okulota nga okakalukana, okalubirizibwa, oba olwanagana mebisolo ela nozuukuka nga okooye. Spiritual diseases may manifest in dreams while one is working very hard, when life is very hard or when one is fighting with animals and wakes up physically very tired.

Obulwadde bwomwoyo bujanjabisibwa ebimera obutabulamu Omwetango ne Bombo. _Spiritual disease are managed using highly spiritual plants that include *Omwetango (Chenopodium opulifolium / ugandaes) and Bombo (Momordica foetida)*

Jiggers may be a physical manifestation of a spiritual problem to an individual or a community.

Empewo ya Ndawula nga ebanja eleela evunza kumuntu oba mukika. The spirit of Ndawuna may manifest as Jiggers to individuals or in family when it is demanding rituals.

Empewo ya Ndawula esobola okubonereza ekitundu nga ebaletera envunza. The spirit of Ndawula may manifest in form of jiggers to the community as a form of punishment.

There was a time when jiggers infested Busoga region and the problem was addressed by specific rituals that involved construction of a shrine for spirit Ndawula.

Places with spiritual powers

Natural places

ebifo ebimu mubutonde bilina amanyi n'obuyinja ebyenjawulo - Some natural places have known special powers, energies or abilities

Buzilanduulu hill is one of the significant natural places

Lusozi Ddindu has the footprints impressions on the rock of Kintu, the claimed first person on earth, his dog and spears

Forests

Waters

Ssezibwa is a water twin entity

Mayanja is a twin entity that is represented in the water form as river Mayanja, Cat-animals family or reptile family such as a snake normally green in colour

Ssezibwa

Ssezibwa is a river believed to have been produce by a woman.

Shrines

Main shrine used by Muwanga, Kawumpuli, Mukasa, Musoke, Kiwanuka, Kadduwanema, Mayembe

One shrine may serve for the spirits of Muwanga, Kawumpuli, and water spirits (Lubaale we Nyanja) including Mukasa, Musoke, Kiwanuka, Kadduwanema etc.

Other Shrines for: Muzimu, Bulamu, Ndawula, Ddungu, Bamweyana, Kaliisa

The spirits Muzimu, Bulamu, Ndawula, Ddungu, Bamweyana and Kaliisa usually need their own bases or shrines

Health management

olulimi lutonzi- omulubale kyayogera kyetondeka kwoyo gwakigambye – the tongue has creative powers – the words spoken to a client by Mulubaale using his/her tongue come to be

Lubaale akola obulungi addizibwa obujja nga kumpi buli mwaka nga akolerwa emikolo omuli okusaddaka enkoko n'ebisolo - for effective health management using ancestral spirit, periodically, usually annually, the ancestral spirits are rejuvenated and their powers renewed by rituals involving sacrifice of chicken and animals.

Ntera okufuna obubaka n'ebiragiro kumulwadde atanajja, olumbe olumuluma n'ekyokumukolera – Often I get dreams about a client yet to come, his/her ailments, and how to manage.

Health issues of cultural origin are managed through traditional rituals. I have been consulted by various categories of people including prominent religious leaders on health issues of cultural origin whose issues I have effectively addressed by performing few cultural rituals.

Causes of illness and diseases

Lubaale – Ancestral spirits

Lubaale Kiwanuka asuula abantu eddalu nga tebanamuteleza: Ancestral spirit Kiwanuka causes madness in some people when not well harmonised

Musoke is unique in his ways of causing and managing illness and diseases. Musoke is responsible for illness and disease associated with dehydration, anaemia, infertility, miscarriages, abortions, menstrual disorders, loss of amnionic fluid during pregnancy. These diseases are managed by rituals that involve presentation of waters in a *nsumbi* (a brown water clay pot with a long neck. It may have one, two, three or more necks

When the ancestral spirit is demanding for acknowledgement or anything, it may demand for Nsumbi (clay pot) or any of its other symbol. It the process of demanding, it will cause illness or diseases that may be uplifted when its demands are fulfilled.

Musoke is the spirit that easily pardons. It one come in with an issue, illness or disease caused by ancestral spirit Musoke, the spiritual healer may recommend a communal meal with specifics for Musoke and when well done, Musoke may go ahead to pardon and uplift the problem, illness or disease.

In case you had a misunderstand with your parent and then your parent dies before he/she had pardoned you, it means you were cursed before your parent died and you carry that curse with you. Such curses are bad and can be responsible for one’s problems, illness or disease. When I receive such a case, I present it to spirit Musoke for management. Musoke will demand for some symbolic artifacts, rituals and a communal meal to uplift such a curse on behalf of one’s dead parent.

In case of misunderstanding in a family or in a relationship and people have separated, or when the family spirits are in disarray, Musoke will address that issue by recommending preparation of a communal meal (*ekijjulo*) and when well done things will normalize.

Musoke is not considered a healer spirit. Musoke is not involved in direct offering of health services but his role is in making necessary corrections so as for the responsible spirits to uplift the causes of the problems. Musoke does not address the problems directly.

Musoke anywa enkuba – Musoke can drain the sky of pending rains

Kinene

Kinene is a spirit and can be responsible for the manifestation of scaring dreams of dead people, night dancing *(okusera).* People who get possessed by the spirit Kinene are restricted from attending burials, or looking at a dead body of the person, otherwise s/he may feel like eating the fresh of the dead body.

Plants commonly used in Health Management

Plants used in empowering (*Mukuwanga ensawo ya Lubaale, ebbibbo bya baloongo*)

The money saved within the ancestral bag and baskets is not used for burials and related functions (kuziika, Kubagiza, kwabya lumbe), nor for humanly love affairs.

Bombo

Lweza

Mulamula

Kafugankande

Mavigamukulu

Namirembe

Lumanyo

Muwanga

Kakumirizi

Kayayaana

Lumuli lwe nkalira

Coffee beans (emwanyi, Ntabaluganda)

Others used

Matembe

Ensimbi engezi

Witchcraft

There are people who are born as witches because the ancestral spirits they possess were highly involved in witchcraft.
