# Supplementary material for: Perspectives on health, illness, disease and management approaches among Baganda traditional spiritual healers in Central Uganda
Source: PLOS Glob Public Health. 2024 Sep 6;4(9):e0002453. doi: 10.1371/journal.pgph.0002453 (PMC11379289; doi:10.1371/journal.pgph.0002453)
Supplement: S2 Data — (PDF) [file pgph.0002453.s002.pdf]

Study participant 2 transcription

Contents

Study participant 2 transcription 1
socio-demographics 3
Mulubaale 3
Problems associated with becoming a Mulubaale 3
Becoming a Mulubaale 5
Okutendeka Lubaale: 5
Lubaale we bu Kojja – Ancestral spirits from maternal *side* 6
Ssenkulu 7
Sources and Access to healthcare information 7
Sources of healthcare information 7
Referrals and Comparing Traditional with Western medicine 8
Health, Illness and disease 8
Words and phrases that describe health, illness and disease 8
Health 8
Illness 8
Disease 8
Health management 8
Health assessment and diagnosis 9
Prevention, protection, health promotion, (*Okugema, Okuganga*,) 9
Okuganga 9
okunywa eddagala 9
Causes of illness and disease 9
Lubaale (ancestral spirits) 9
Omukago (traditional covenant) 10
Witchcraft 10
Biological and physical causes 11
Health assessment and diagnostic tools 11
Symbols and symbolism 11
Songs 11
Health management 12
Animals and birds used in healthcare provision 12

Cow 12
Sheep 12
Lubaale (Ancestral Spirits) and their characteristics 12
Lubaale we Nyanja 12
Misambwa emitonde 13
Kabaka Mukasa 13
Kadduwannema 13
Mukasa 13
Kiwanuka 14
Musoke, ... 14
Lubaale wo kulukalu 14
Misambwa emitonde 14
Muwanga Ssebyoto Lukankana 14
Misambwa emizaale 15
Muwanga omuzaale 15
Kawumpuli 16
Bamweyana 16
Ddungu 17
Characteristics of Misambwa 17
Mayenbe 18
Characteristics of Mayembe 18
Jembe Lubowa 18
Mizimu 18
Balongo 19
Characteristics of ancestral spirits 20
Places of spiritual significance 20
Natural places 20
Forests 20
Water bodies 20
Mountain tops and rocky places 20
Shrines 20
Lubiri 21
Kiggwa 21
Sabo 21
Fire places 21
Ekyoto ky gombolola 22

Road junctions 22
Animals and animal parts.. 22
Challenges among Balubaale 22

socio-demographics

My name is (study participant 2). I am a married Muslim adult (77years), male Muganda of Ngali clan. My level of education is Junior 8 which is equivalent of Senior 3.

My occupation is a traditional healthcare spiritualist. My father is Aramanzan Kyeyune Bakidawo and my mother is Safina Nabatanzi.

I am in Busiro County (Saza), Wakiso district, (xxx) Sub County, (xx) parish, (x) village. I am a Mulubaale and I belong to Uganda N'eddagala Ly'ayo traditional healers association. I belong to a healers' association of Baalubale that fulfils the objectives of traditional healthcare spiritualists. I have been a practicing Mulubaale for 37 years. I become 62 years, I retired from Makerere University in May 2004 and become more active as a healthcare spiritualist.

This shrine is a sub-clan shrine, Kiggwa.

Mulubaale

Problems associated with becoming a Mulubaale

It was not an easy process for me to become a Mulubaale.. It started when I was about 10-11 years of age. In the beginning, it sent indictors, signs and symptoms that showed that in the long run I would become a spiritualist. I can group those indicators into sickness, dreams, seeing images of people or animals (*obutulume)* when all the other people cannot see visions.

I went through lots of problems before I finally agreed to harmonise Lubaale and become a Mulubaale.

When I was about 10-11 years, one evening after the last meal of the day and everybody had retired to sleep, all lights were turned off and there was darkness everywhere. Suddenly I saw a bright light in the room where I was, and saw many gigantic men, dressed in white, moving towards me threatening to eat me up. I screamed out loudly seeking for help. All the relatives got up and came for my rescue. I explained that I was seeing those men who are threatening to eat me up, but none of my relatives could see them nor understand what I was talking about amidst the bright light that I was seeing. Suddenly I saw darkness eating up the light and night set in again.

The following week, I become very weak and sickly. I could not tolerate darkness, so my parents kept on the lantern throughout the nights. During the day, I could three scaring white men dressed in white cloths doing their own businesses, but none of family people could see them. They were not bothered about me during the day, but scared eating me up at night. So, I

could scream out seeking for help at night and family members hought that the high fevers had affected my brains.

I also developed high sensitivity to sound especially from my relatives. I could hear their conversations at a distance of more than three kilometres away, beyond valleys and hills. When the relatives would come back, I would tell them exactly what they had talked, which surprised most of them. I can relate this to a situation where someone's phone rings its self to mine, I hold it and hear all their conversations without their notice. I also developed higher sensitivity to other things. For example, I could see through walls. If anyone did anything across a wall, I could be seeing whatever is being done as if it was a see-through or transparent wall.

Since my grandfather could not afford to keep the lantan lighting all night, I was forced to spend the nights outside, at the house front door for about three months in an attempt to avoid night room darkness and the threatening men. My grandfather transferred me to my mother's home. This time, in the evening, I saw a stream of animals (*obutulume),* which my mother could not see when I called her in. The whole night my mother kept awake because I kept screaming due to men intruders threatening to eat me whenever she put off the light. After explaining, she left the lights on until morning. In the morning my grandfather, my father and aunt gathered at home.

"Senga, mukulu wa taata yali yasamira" My aunt, the elder sister to my father, was an accomplished spiritualist, had fully harmonised family ancestral spirits. Out of her own experience, she easily understood my health situation and related it to ancestral spirits. The following day, the aunt came with a big luggage (*mugugu gwa Lubaale*) which she placed between me and her, opened it up, and handed me two 10 Cents coins and told me to talk saying some words after her, which I did and she told me to put the coins in the luggage. She also did the same, after which she closed the luggage and took it back to its custody. By that ritual, she had made a *kakalu* with my ancestral spirits. (*Lubaale weda yali awulira era nga akakalu akasibibwa kakola),* After that ritual, my scare experiences stopped and my health improved. I went back to school until I completed, and got employment with Makerere University.

The aunt was born during the reign if Kabaka Muteesa 1. During her adulthood, she produced nine (9) children and lost eight (8) of them through death. The one survivor child was produced after her *Kusamira*.

In 1979 I went into exile, however, I survived all the bad experiences while in exiled until I returned in 1981 and re-joined my job at Makerere University after 2 years of exile. I got money which slowly money dwindled to zero. My life become bad until one day a lady took me to a spiritualist lady (name removed) who diagnosed that my life was miserable and would continue to be so until I addressed my ancestral spirits (*nga Nsamidde).*

While at the university I lost almost everything and become very poor. I sold every household property and I was left with only a mattress and a bed. One day I convinced my wife and I sold the only mattress we had, to get three thousand shillings (3,000/=) to start on the process of Lubaale expoloration (*Okwaaza Lubaale).* I explained my situation to two of my sisters who joined me. We agreed and planned for the kwaza process, which we expected to last for three days, but the process lasted for five (5) months. During the five months I was not bathing, not washing my cloths, not cutting nails or doing any hygienic measures (as a researcher I wondered why and What is the significance, interest or intention of Lubaale by keeping the people in very unhygienic process of not brushing teeth, not bathing, and not washing clothes, not cutting down the nails?). The process ended with *Okutuuza Lubaale* (settling the ancestral

spirits). When the process was over, my life got better, I got money and everything got better. The ancestral spirits had told me that when I get money, I should first use for harmonizing Lubaale, unfortunately I diverted the money I got for into business, which business failed completely.

Becoming a Mulubaale

After many attempts and failures, I was finally directed to *Makolo*, a prominent and experienced mentor spiritualist for my *Butende*, an informal but structure training. After all the processes of kusamira Lubaale, I was subjected to some conditions. (1) I was to go back to work at Makerere University until retirement age, after which I was to work with Lubaale and do its activities. (2) I was to start working with the University but report for *butende* (apprenticeship) every Friday evening after work, without fail and without any excuse and stay until Sunday evening, to go back to the University.

Sibulimuntu inti asobola okuba omulubaale. Nze okufuuka omulubaale nayita mumitendela gya lubaale emisengeke obulungi, omuli okwaaza lubaale, okusamira lubaale, okutuuza lubaale, okusalira lubaale, okukkaliza lubaale, okunywesa lubaale, akasera ka lubaale, okusiba amaliba ga lubaale, n'okutendekebwa mu butende. - Not everyone can become Mulubaale. to become Mulubaale, I went through a systematic process of ancestral spirits exploration (*Okwaaza lubaale),* induction and training (*okusamira Lubaale*), initiation (*Okutuuza Lubaale), throat-cutting (Okusalira Lubaale), (Okukaliza Lubaale) (Okumwesa Lubaale),* examination (*Akasera Kalubaale), (Okusiba amaliba ga Lubaale),* apprenticeship (*Obutende*).

Okutendeka Lubaale:

Okutendeka Lubaale is currently an uphill task. If the original Muzimu had performed all the tasks required for Lubaale, the ones being possessed by that Lubaale may not be able to do all the needful for that Lubaale, instead they do bits and bits and by the time they die, they have not completed all the requirements for the Lubaale. When their spirits Mizimu come back and possess other individuals, the individuals also end up doing parts of the previous spirits' work. So currently there is hardly a recent Muzimu that had completed all the rituals of the Original Lubaale as per the original Muzimu, the owner of the Lubaale.

Omuzimu bwegulinya kumutwe gubuzibwa "ebibyo byonna byakuwebwa era tobanjan?" – When one gets possessed by Muzimu, the Muzimu is asked "Were you given whatever you rightfully demanded?" The answer is either yes or no, but commonly no. The muzimu can control over whatever was fully harmonised but will not take control over the spirits it did not fully harmonise. The option is to look for the Muzimu that last fully harmonised its Lubaale.

Waliwo empewo ezikolebwa newabelawo eziwebwa obuwebwa ekifundikwa. Some spirits are harmonised by rituals, offerings, sacrifice and trainings, while others are harmonised by giving them their regalia.

Lubaale bwajja, alanya eli Senkulu. Bwamala ne Ssenkulu naye nalanya eli lubaale. Ne Jjaja wa Lubaale naye nalanya. – When the ancestral spirits possess an individual for the first time in-front of its trainers, it introduces itself fully to the trainer, giving its ancestral lineage. The trainers also introduce himself in details to the ancestral spirits, Also the person present as the grandparent to the ancestral spirits does the same.

Omuzimu ne Lubaale na Mayembe bya gwo bwebyeyoleka, bitelezebwa nebitendekebwa, era bisobola okukyamizibwa ne ntendeka embi oba abatendesi ababi – When Muzimu and its Lubaale and Mayembe express themselves, they undergo training and they can be misled by bad training or bad trainers. Good and strong ancestral spirits can be misled by the trainer (Senkulu) for his own benefit, normally by the good and strong spirits working for him. At which time the spirits are changed from their ancestral rail to that tuned by the trainer.

Kikyusankanamu means (yenna lubaale omuntu gweyasamira - "musuluru moja" - amusimba mabega)

Akakalu

Lubaale weda yali awulira era nga akakalu akasibibwa kakola, naye Lubaale wa kakati tekyawulira. mwegayirira naye tawulira).

Lubaale we bu Kojja – Ancestral spirits from maternal *side*

Lubaale we bu Kojja asobola okweyoleka mukika, era sikirungi kumugobaganya - Lubaale from maternal side may appear in one's clan and it is not right to be against it.

*T*his Lubaale (maternal Lubaale) may appear in one's clan for two major reasons. When a man gets a wife and the Lubaale from the wife wants to be harmonized, but the people of the wife's side are hesitant or are not interested at all, yet the husband is willing to assist the wife to harmonise the Lubaale, Lubaale will be requested if it would allow to be accommodated at the husband's side. If Lubaale accepts to be hosted at the husband' side, the husband will finance the harmonization of that Lubaale and a separate house will be made aside to accommodate the Lubaale of the wife. The Lubaale of the wife and will acknowledge the husband as *a munywanyi.*

When the children grow and have grandchildren, this maternal Lubaale may possess any of the children and grandchildren. If at any time in the future, the family or clan of the wife gets interested in that Lubaale, they have to pay a price, determined by the husband, to the husband to get that Lubaale back into their clan. This process is spearheaded by *Lubaale Mukasa*, who represents the rest, while possessing a person. The husband gets some of the money he is given, puts it in a basket and releases to Lubaale to go, but requests the to always be mindful of the husband's family and grandchildren.

The second aspect of the *Maternal Lubaale* comes when the spiritualist lady, gets married, gets the Lubaale harmonised, get children, later dies, and gets buried at the husband's place. The Lubaale of that wife has the two options to go back to the clan of the wife, or to stay at the husband's clan. That Lubaale may possess any of the husband's children, even if the dead wife was barren and never produced any children. However, if the child possessed by that *Maternal spirit (Lubaale we bu Kojja)* get old or dies, that Lubaale may go back to its original clan, but bearing the name of the person it possessed from the husband's clan. The point to note here is that in case Lubaale comes with a name that does not belong to your clan, do not just chess it away, instead settle, and get clarity (*tuula wekanye)* on how that could have happened. That Lubaale will be willing to explain in details how it came to be that way.

The other thing to be careful about, especially by the *Senkulu*, is to know that the same *Senkulu* for the Lubaale in the clan (*Lubaale we kika)* cannot and should not be the same *Senkulu* for the *maternal Lubaale (Omukulu takulira mpya bbili, Senkulu w'Kika tatekwa at kubeera*

Senkulu wa Lubaale w'bukojja. Senkulu afunayo Senkulu mune afuuka Senkulu wa Lubaale we Bukojja. (kino kikolebwa okwewala obuko mu Lubaale).

Ssenkulu

omukazi tafuuka Ssenkulu wa Lubaale, nebwabera nga aliko Muwanga. Lubaale asobola okugana okutuzibwa muddiiro ly'omukazi era nagaana okuteleera. – A woman cannot become a Ssenkulu, the chief trainer of Lubaale, even if she is a medium for Muwanga. The ancestral spirits may refuse to be harmonised by a woman.

Senkulu and Lubaale spirits have father-son relationship.

Lubaale were human beings and therefore are counselled by their Ssenkulu. Senkulu is respected by Lubaale and Lubaale tries never to offend its Ssenkulu.

Sources and Access to healthcare information

Access health information and treatment modalities are many folds: the use of material (herbs) and use of non-material (*amanyi n'obuyinza bwempewo*).

Sources of healthcare information

Some people are gifted with powers and abilities (*abantu abamu balina ebitone byamanyi n'obuyinza).*

(abamu Lubaale abalosa buloosa buli kyakukola naye nga Lubaale talinyangako wade okwogerera ku mutwe gwooyo omujanjabi). Some people just dream and within the dreams, they are clearly given the description of the herbs to use, and how to prepare them for the treatment of some given ailments or particular individuals' health condition

Nze nina olumanyo, ndabira awo nga manyi ekyokola nengeli yokukilolamu – I have a natural ability to know, I find I just know what to do and how to do it. Some information comes intuitively just like that,

Nina amanyi, obusobozi n'obuyinza – nsobola okukozesa buli kintu okujanjaba – I am naturally gifted with some powers and abilities to use anything for healing.

Olusi nina kusamira Lubaale okumanya ekyokukola - At times, I have to be possessed by *Lubaale ancestral spirits)* that talk in order for me to know what to do. B*asamize* get to know and access healthcare information through the spirits talking to them and giving instructions on healing others.

Olusi Lubaale ayogelera mubantu abalala - Some information is got through instantaneous talks or conversations with other people, somehow sent or set by the ancestral spirits or powers).

Referrals and Comparing Traditional with Western medicine

It is worth noting that there are health conditions where Western medicine may be better and faster than traditional approaches, but also there are health conditions where traditional healthcare approaches are most suited and Western medicine completely fails.

The most important aspect for all healthcare practitioners (Western and Traditional), is to know and respect our limits and limitation, to know where and which healthcare conditions we can manage and those that we cannot manage. We need to refer cases that we feel we are not competent to manage.

Health, Illness and disease

Words and phrases that describe health, illness and disease

Health

Obulamu obulungi bwebulamu nga tewali kikunyigiriza – Go health is when the life has no difficulties nor hardships. A person who is health is one able to breath well, eat well and do daily activities without any bother.

health refers to physical health,

Illness

Disease

Obulwadde is when one gets out of the "considered normal health condition", and enters a health condition that does not provide peace to him/her.

Obulwadde obwenkukunala is sickness with obvious signs and symptoms, such as painful swellings

Okunyigirizibwa mubilowoozo; obutakkanya, amabanja, enkayana nga ezettaka, obutakkanya mumaka, abaana bo okukukyawa, okunyigirizibwa mubyenfuna. All these forms of *Okunygirizibwa* can end up manifesting in physical sickness like hypertension or ulcers. (Social aspects of disease)

Health management

Nze, omulwadde wa Lubaale simuwa ddagala kumukakanya, okujako okwogerezeganya naye okuwayo ebanga omulwadde yetereze mubyensimbi akole Lubaale. - For me, when a client

comes and I find out that the problem is related to Lubaale, I do not give any medications, instead I advise the client to go and request the Lubaale for a grace period and for ability in form of money to do the needful. This is because, in my case when Lubaale was the issue, when the spiritualist healer gave me medicine, Lubaale become even worse in the way it demanded to be done. Everything became worse. So, I learnt not to give medicines to cool down Lubaale but request and negotiate for a grace period. (With no schooling, the healers health practices put in question the wisdom of the learned)

Health assessment and diagnosis

Prevention, protection, health promotion, (*Okugema, Okuganga*,)

Okuganga

okuganga omubiri

okuganga awaka

okunywa eddagala

Infertility in women is managed by *okunywa oluzaalo,*

Causes of illness and disease

olumbe oba obuluwadde olusi bibonerezo by'okuvoola Lubaale. Some health conditions are purnishments for belittling ancestral spirits.

Lubaale (ancestral spirits)

Lubaale can be a cause for infertility

Lubaale can cause infertility in women: This may happen when such women have Lubaale which is very sensitive to urine, faeces or noise from Children. (*nga Lubaale tayagala kumufukira, kumupamira oba kumuwoganira namaloboozi g'abaana abakaaba.).* Such Lubaale may even refuse such women from having husbands.

In case of men, Lubaale can be responsible for their failure to have children in case Lubaale feels that the spiritualist may fail to give love to his non-biological children (*Abatende, abaana ba Lubaale).* This had happened to one of the most prominent healthcare spiritualists by names of *Makolo* until many ritualistic processes were performed and *obukalu* (agreements) were

made that in case he gets children, he would love as well the non-biological children (*Abaana ba Lubaale, Abatende)*

*Lubaale may p*lan many years ahead, it plans for whom it will possess at time many years before conception of the woman. That is why *Lubaale* is not transferable. a spiritualist cannot opt to change Lubaale from one person to another.

Walumbe ne Kitambo have much in common but are not the same. If one cannot handle issues of Walumbe, certainly he/she will not manage to handle Kitambo.

Kitambo is a spirit called Walumbe

Walumbe

When Walumbe spirit is demanding, one dreams of dead people.

Walumbe spirit is harmonized depending on the ancestral lineage

The respondent narrated a story relating to Walumbe. Gulu and his wife are the parents of Walumbe, Kayikuzi and Nambi

Kitambo

Kitambo is considered a twin spirit

Omukago (traditional covenant)

Omukago is a blood binding relationship between entities.

Omukago used by our ancestors used to involve two coffee bellies (*emiwula gye mwanyi)* of the same coffee bean, where each of the bellies is smeared with umbilical blood cut from one person and swallowed by the other person, with whom the friendship is being made and vice vasa.

The respondent narrated a failed *mukago* between Buganda and Busoga attempted by a King of Buganda. The king of Buganda once tried to forge o*mukago* relationship with Busoga which was not fulfilled. The Buganda King sent some people to take soil from Buganda to Busoga, but the messengers were fooled by Basoga people and instead poured the soil at one place in Buganda called Takajunge. This place actually exist up-to today

Mukago can be inherited and is very significant and binding to the grandchildren

Mikago were done to enable the privileged to help and work with the less privileged, since individuals and spirits abilities vary.

Witchcraft

Witchcraft generates fear from within the individual involving the thoughts, spirit and the body – *"eddogo lileta okutya mubilowoozo, mu mubiri ne mu mpowo zoomuntu"*

Witchcraft brings fear from the environment, its people and spirits. – *"Eddogo liletera omuntu okutya ekifo mwali, empewo n'abantu abaliwo"*

Witchraft causes problems, difficulties, illness and diseases – *Eddogo lileta ebizibu, okukalubilizibwa, enyimbe n'endwadde.*

Biological and physical causes

Health assessment and diagnostic tools

Health problems or concerns are identified through health assessment, "diagnosis", root causes and health management plan are done by spiritualists or the spirits through listening to history, physical examination, spirit possession and use of diagnostic tools (Omweso), etc.

Symbols and symbolism

Every ancestral spirit has a symbol by which it is identified.

Ancestral spirits are associated with particular symbols of colours, shapes, plants and animals of their preference by which they were originally identified.

The dream messages associated with Mayembe demanding harmonization are symbolised by soldiers chasing and beating up the one dreaming or other people

Easy familiarity with nature, Interaction between culture and science, Nature and its mysteries

Black

Black colour is associated with Walumbe spirit. If one's dream relate to black colour, the dream may be about Walumbe spirit.

Interacted with nature

Songs

Songs are used to call upon the spirits. Songs unit all spirits. Singing songs is done in the process of calling upon the spirits to come and possess people of their choices. *Lubaale asobola okujjira kubuli luyimba*. However, there are specific songs meant for specific spirits. Otherwise, any type of spirit can possess someone while singing any spiritual song if the spirit wishes so but there are specific songs for each type of spirit. Songs have meanings and entertainment.

Songs are for entertainment of the spirits and people and are used in rituals and ceremonies. The songs have messages (*obubaka*) embedded in them which are sometime directed to specific spirits. Therefore, spiritual songs are for, entertainment, carry messages, counselling. Spirits are counselled as well as the spiritualist using songs.

Health management

There are situations when you do what the healer tells you to do, the situation gets worse. What then is the problem?

Animals and birds used in healthcare provision

Easy familiarity with nature

Cow

Sheep

Mu nnono ya Buganda, endiga ensajja tesomoka mazzi kuva kulukalu kutwalibwa Sese - In Buganda culture, a male sheep does not cross over waters to be taken from the mainland to Sese islands.

In Buganda culture females never used to eat sheep, so spirit does not take blood (okunywa omugerengejjo) but *abuka omugerengejjo* and does not eat the liver.

Lubaale (Ancestral Spirits) and their characteristics

It is currently difficult to harmonise *Lubaale (enakuzino kizibu okukola Lubaale nateleera)* because *Lubaale ya laluka, Lubaale takyawulira*, religion intercepted the Lubaale process and demonised it, monthly and annual cultural and spiritual rituals from top *(Kabaka)*, through royals *(Abalangira)* to the common person, are no longer being performed. All ritualistic and highly energised natural places have been adulterated. Such places include; *Nsozi Mwenda, Tanda, Walusi, Nyiize, Bukasa, Lusozi Mirembe n'ekyoto kyaakwo, Ekyoto mu Libiri e'Mengo kyafulumizibwa, Amasiro ge'Kasubi okuyitamu omuliro,*

Lubaale is the class or category of spirits that possess a person and talk (*Lubaale zemandwa ezikwata kumutwe nezogera).* Lubaale is further divided into two major groups, the dryland and water related spirits – *Waliwo Lubaale w'okulukalu ne Lubaale we Nyanja*

Lubaale we Nyanja

The water/lake spirits include; Mukasa, Kiwanuka, Musoke, Wanema, Musisi, etc.

Emikolo gya Lubaale we Nyanja gikolerwa ku Ssabo – The rituals for harmonizing wetland ancestral spirits are performed at the shrine.

It is common for Lubaale wenyanja to be symbolised by men dressed in white coloured cloths

Lubaale wenyanja anywa omugerengejjo

Lubaale wenyanja abuuka omugerengejjo –

Sibuli mandwa nti anyway oba ebuuka omugerengejjo!

Lubaale w'enyanja asalirwa kumakya nga busasaana – Rituals for water related spirits are performed at dawn – just before day break. This is done at that time because when Kabaka Mukasa was coming from Sese Islands, he arrived at the showers at a place call Bakasa, at dawn before day break. This draws upon the *kalombolombo* of when Mukasa and his team arrived at the dryland at Bukasa while from Sese across the waters.

Lubaale w'omukyala: if a husband assisted his wife to harmonise her Lubaale and built it a house at the husband's place, when the wife finally dies, and the clan of the late wife want to get the Lubaale back to their clan, the people of the clan of the wife have to pay for the Lubaale. The bring *katamukago (*coffee beans), local brew *(ekita kyomwenge),* sing twin songs (*enyimba zabalongo) and then* request to take the Lubaale and pick some soil from the shrine.

Lubaale abanja bwaterezebwa buli kintu kilongooka - Once the needful is done for *Lubaale* everything gets on very well, health is okay, life is good, and have the children, money and easy life.

Misambwa emitonde

Kabaka Mukasa

Kabaka Mukasa tatendeka - <u>Spirit Mukasa does not train</u>

Kadduwannema

Kadduwannema is the son of Kabaka Mukasa. Kadduwannema had three adult male sons, Mukasa, Kiwanuka and Musoke.

Most Balubaale harmonised Lubaale Mukasa, Kiwanuka and Musoke since their process is done together as a set. Mukasa and Kiwanuka are done in the same sitting the Musoke done the following day or at a later date.

Mukasa

Empewo ya Mukasa yemu kuzasoka okunhogererako - Mukasa was among the first ancestral spirits to appear and possessed me.

Mukasa omuzaale mutabani wa Kadduwanema, muzukulu wa Kabaka Mukasa omutonde – Mukasa, the son of Kadduwanema is a grandson of Mukasa the King. King Mukasa had two children Kadduwanema and Najjemba. King Mukasa was originally the King of the wetland and when he became the King on the dryland, he left his sister Najjemba in his chair on the wetland (Kulubisi), and his role on the wetland was then forgotten. However, his son Kadduwanema produced children and named one of his sons Mukasa who become very popular as Mukasa omuzaale, a grandson of Kabaka Mukasa omutonde.

Lubaale Mukasa is associated with white colours, and is harmonized by a white male goat, white male chicken.

enkanamu ya Lubaale bagisibira mumaaso so simabega nga kisenso – The skin-hide for the sacrificed animal is tied in the front of its medium while standing, but not behind. The following day at the same time at 5.00 am in the morning, the skin-hide from the goat sacrificed for Lubaale Musoke is also tied on the same human medium while possessed by Musoke spirit.

However, when the second skin-hide for the next animal is being tied round the human medium, the previous skin-tide of |Mukasa is turned to face the opposite side, a process referred to as *kikyusa nkanamu.*

Kiwanuka

waliwo emeeza nga etwalibwa muwanika newankubadde nga emeeza teli mumatwaale ge. "emeza ya Kiwanuka eli mu nyanja" - The treasurer was responsible for the table even-though the table was not in his area of jurisdiction. The table for Kiwanuka is in the lake (Use of words in spirituality is not a cram work but words are used and understood in context, other-words many mistakes are made in the process

Musoke,

Lubaale Musoke is a male spirit, a son of Kadduwannema and a grandson of Kabaka Mukasa.

Musoke is a male spirit but unlike for Mukasa and Kiwanuka, Musoke is hermonised by use of a female goat with a patch around its belly.

Since Lubaale Musoke is the youngest of the sons, he presented the feminine is given a female goat to symbolise the need to produce females in the family. That is why when the women get menstruation periods they say "*agenze busoke*".

The skin-hide of the sheep used for the process of Kiwanuka spirit is used as a *Kiwu* to seat on by all the Lubaale spirits of Mukasa, Musoke and Kiwanuka.

Lubaale wo kulukalu

Dryland spirits include Kawumpuli, Muwanga, Bamweyana, Magobwe, Serwanga, Nabuzaana, Wanema, Walumbe etc.

Misambwa emitonde

Muwanga Ssebyoto Lukankana

Muwanga alagula, agaba eddagala ayambulula – Muwanga divines, gives medicines and performs cleansing rituals

Who is Muwanga?

Muwanga omusambwa omutonde gwava mu ggulu kuntandikwa y'ensi, era neguwebwa amanyi n'obuyinza ku bitonde, empewo endala zonna namatwale gaazo gonna, okuziwanga n'okuziwangulula, okuzisobozesa okutukiriza obuvunanyizibwe bwaazo. Muwanga is a natural spirit that descended from the sky at the beginning of the world and was given powers over all the creation, the spirits and their fields of influence to empower or disempower all the spirits so as to enable them fulfil their inherent responsibilities.

Muwanga is Lubaale. When a person dies his spirit becomes a Muzimu which might later become a Musambwa and Lubaale depending on the time that passes by and the spiritual powers of the spirit.

Characteristics of Muwanga

Muwanga alagula, agaba eddagala ayambulula – Muwanga divines, gives medicines and performs cleansing rituals

Muwanga awanga empewo – Muwanga empowers all other spirits

Muwanga awanga ebiggwa – Muwanga empowers family shrines

Misambwa emizaale

Empowo ezokuttale mulimu emisambwa Mayanja/Sserwanga, Dungu.

Entekateka yokuteleza emisambwa gy'okuttale, emismbwa gikomezebwawo awaka nezikkalizibwe nezitabaganyizibwa n'ezawaka, ezenyanja nez'okulukalu nga Bamweyana, Muwanga ne Kawumpuli,

Muwanga omuzaale

Muwanga omusambwa omuzaale ava mu Kintu (Muwanga is an ancestral spirit from Kintu)

Muwanga omusambwa omuzaale was a Katikiro of one of the Kings of Buganda.

A story of Muwanga was narrated by the respondent. Muwanga as a Katikiro of Kabaka was left to administer the Kingdom on behalf of the Kabaka when the Kabaka went for a long trip out of the kingdom. By the time the Kabaka returned, the Katikiro Muwanga had gone to check on his family, so he was not around to welcome the Kabaka. This was very abnormal to the Kabaka for the Katikiro not to be present to welcome him back.

Incidentally the pregnancy of the the king's wife was visible and the Kabaka suspected the Katikiro to be responsible and that being the reason for the Katikiro Muwanga being out of the Kingdom for fear of the wrath of the Kabaka.

When Katikiro Muwanga was informed of the return of the king. He came back to the Kingdom to see the Kabaka. However, the furious Kabaka because of his formed opinion regarding his pregnant wife, threw a spear at Katikiro Muwanga and hurt his leg. Katikiro Muwanga was forced to run away and exiled himself in a thicket at a place that later came to be called Munseke.

After some time and after taking closely with his wife, the King realised that actually the pregnancy was his. So, he felt apologetic to his Katikiro Muwanga. The Kabaka also felt very

lonely without his long-time friend Katikiro Muwanga so the Kabaka sent a team of his men to go and invite Muwanga back to the Kingdom. When the messenger team of men reached Katikiro Muwanga at the thicket where he was and they informed him that the King had sent them to invite him back to the Kingdom, Muwanga formed an opinion that the King was now planning to completely kill him instead. So Muwanga made a plan to run away from this messenger team. Muwanga brought local brew to the team without the Nseke drinking pipes, he told the team that was getting for then the nseke from nearby. In the process he escaped from the team and went to another thicket later called Kaligwa at his maternal side to hide from the Kabaka and his team. Muwanga expected the worst to follow so he told his maternal people that *akaligwa eno tekaliba kalungi,* thus the name Kaligwa. That is why Muwanga is referred to as of two places *Muwanga we munseke and Muwanga we Kaligwa.* However, Muwanga is the same.

Muwanga is the same spirit but with variable ways of working which are determined within the clan, the trainer (*Senkulu),* the training process and experience.

Muwanga we Nseke

Muwanga alagula, agaba eddagala ayambulula – Muwanga divines, gives medicines and performs cleansing rituals.

Muwanga we Kaliggwa

Muwanga alagula, agaba eddagala ayambulula – Muwanga divines, gives medicines and performs cleansing rituals

Kawumpuli

Kawumpuli alagula, agaba eddagala ayambulula – Kawumpuli divines, gives medicines and performs cleansing rituals

Kawumpuli asobola okukola lubaale, okutendeka, (singa Muwanga aba tanatelezebwa) - Kawumpuli can perfom the rituals to harmonize and train ancestral spirits in case Muwanga is not yet harmonized.

(Kawumpuli ya wanga n'okutereza Muwanga kumutwe, okuwanga n,okumukwasa Ddamula, n'omweso. It is spirit Kawumpuli with powers and abilities to harmonise, empower and hand over the stick of authority and the diving or diagnostic tools set.

Omuzimu is superior to Kawumpuli but Kawumpuli empowers and advises the Muzimu.

Bamweyana

Bamweyana, alagula, agaba eddagala ayambulula – Bamweyana divines, gives medicines and performs cleansing rituals

Bamweyana mulaguzi mulungi nyo, - Bamwayana is very good in health assessment and diagnosis

Ddungu

Ddungu Musambwa gwa kuttale

Emikolo gye Misambwa jokuttale, nga Ddungu, gikolebwa Kuttale - The process of harmonizing the ancestral spirits of the wilderness, like ancestral spirit Ddungu are performed in the wilderness

Ensolo ya Ddungu jeyasaba emulagibwa nga alikumutwe natwalibwa mu ttale okujiyigga ne ffumu n'olutuula – During the harmonization process for spirit Ddungu, the animal he demanded is shown to him and he directed to the wilderness to hunts for it using all the hunting gear like spear, nets etc.

Nga emikolo gye misambwa gye ttale giwedde, e Misambwa gye ttale giletebwa mussaboe okutabaganyizibwa ne Lubaale we kika - After the harmonization rituals and ceremonies for the spirits of the wilderness, these spirits are brought to the shrine in order to harmonize them with the other ancestral spirits of the clan.

Characteristics of Misambwa

The ways of working of Misambwa are different from other types of spirit.

Emisambwa jokuttale enkola y'ajo elimu akettale, n'emikolo jaajo jona gikolebwa ku ttale – The working system of ancestral spirits based in the wild has wild aspects and all their rituals are done while in the wild. Example (1) *Ekibuumba kye kibaako akanyama akasooka okusalako akawebwe ettale nga kabisi.* – When an animal is slaughtered for the Misambwa emizaale, there is a small piece of fresh meat, next to the liver, that is cut as thrown to the bush. Example (2) *Amatoke gakwo gokyebwa bwokyebwa.* – The bananas are just roasated.

Muwanga, Kawumpuli, and Bamweyana are diagnostic and predictive spirits, and can prescribe medications and perform ritualistic removal of bad spirits by performing ritualistic cleanings baths).

The rest of the spirits work in their own ways to maintain the health of the individual clients and guide them in their activities (*kubawa, kubaloosa, kubakuuma)*

Jjaja Muwanga tasala nsolo nga alikumutwe, naye kusawa yokusalira, Jaja Muwanga agezesa ekyambekye kubulago bwensolo emirundi musanvu (7) kulubaale we Nyanja ate emirundi Mwenda (9) kulubaale wo lukalu. - Ancestral spirit Muwanga never slaughters any animal but uses this knife to simulate throat-cutting seven (7) times for water related spirits and nine (9) times for dryland spirits

For Lubaale w'enyanja, most activities rhyme with (x 7); that is *okugeesa x 7, okwetolora x 7* reflecting parts of the human being [2 legs, 2 hands, 1 abdomen, 1 Chest and 1 head) = 7]

Lubaale w'olukalu, the ritualistic activities of Lubaale w'olukalu rhymes with (x 9), reflecting the orifices (openings) on the human body, that is 2 eyes, 2 nostrils, 2 ears, 1 mouth, 1 anus, and 1 urine/vagina outlet = 9). All this is done this way to follow the originality of the issues. (*bino byona bikolebwa okujayo obulombolombo nenono y'abo abasooka nga bwebakola. Lubaale alina ennono ye*.

Mayenbe

Mayembe are soldiers (protective) spirits for Lubaale and its people.

Nkola nentekateka amayembe. I make and prepare Mayembe spirits

Ejjembe nditekamu eddagala lyo Mukago lisobole obutabanja nga litta bamukika – When I am making Mayembe, I put in materials for covenant so that when the Jembe is demanding for harmonization, it does not kill clan members. Mayembe become unruly when not well prepared nor counselled.

Every spiritualist has his/her own main Jembe, Normally the Jembe that expresses its self during the process of Kwaza Lubaale is the main Jembe for that spiritualist.

Mayembe are spirits made of some materials, put in homes or work places for various reasons.

The materials are housed in a durable container such as a horn of any animal and the spirit agrees by spiting twice into the container of the medicine.

Characteristics of Mayembe

Mayembe galagula, gagaba eddagala gayambulula – Mayembe divine, give medicines and perform cleansing rituals

Amayembe gakola emirimo ejagalesa mu kika - Mayembe serve the purpose for which they were brought into the clan. For instance, business, family issues, war, reproduction.

Ejembe liwangibwa mubuli jembe lyensolo yonna naddala ejembe lyensolo eyo eyasooka okuliwanga gyeyakozesa. Ekyo kyekyafuuka enono ye Jembe elyo. Ela ejembe bweliba liwangibwa linyonyola enjumba yalyo mwebaliwanga. However, with the scarcity of the wild animals which are highly protected and their products highly restricted, time has come when we shall request the Mayembe spirits to use the available or domestic animal horns.

When the Mayembe were being brought in the clan for the first time, they were given proper instructions, guidance and counselling. However, these days, people are given Mayembe without proper instructions, guidance and counselling which causes problems for the generations that follow especially when the Mayembe become unruly.

Jembe Lubowa

Jembe Lubowa is the head of all Mayembe spirits in Buganda.

Mizimu

Omuzimu (*Kantunsimbi)* was of my grandfather who died in the early 1940's.

Buli Muzimu ne Lubaale wagwo gwegwasamilanga – Each Muzimu has its ancestral spirits it harmonized.

Omuzimu guba nobusinziiro ate namakanda. Muzimu has its origin and its base. The base may be the same place or different from the origin. The origin of the Muzimu is usually the ancestral base where the original Muzimu that owned the current Lubaale was laid to rest (the grave). While the Base of Muzimu is the place where the current Muzimu that carries the ancestral spirit has his work base, where Muzimu shrine is built.

Muzimu wants to be very clear in its deliberations and tries to insist that it is well understood. It is not bothered by as many questions as possible provided it is towards understanding.

Muzimu is always available when called upon on issues of substance and on track, however, when Muzimu is called upon and it fails to come, it should be immediately noted that there is a problem, either issues are not of substance or are off the track or the environmental circumstance is not appropriate.

Omuzimu gutambula ne nnono yaagwo – Muzimu moved with all its spiritual package

Characteristics of Muzimu

Omuzimu gulina obuyinza bungi nyo mu mpewo - Omuzimu has a lot of authority among other spirits.

Omuzimu ogwasembayo okutukiriza Lubaale wagwo gwe gunanyi lubaale. – The last Muzimu to perfect its Lubaale rituals is the owner of all the ancestral spirits.

Omuzimu tegukulira lubaale gwe gutasamira – Omuzimu does not head the other spirits it did not harmonize

Omuzimu gunanyini Lubaale gusobola okuwa olukusa omuzimu omulala okutambuza lubaale wagwo – The muzimu which owns its other spirits can authorise another muzimu to cary on with its roles regarding the spirits it owns.

Omuzimu gulagula - Muzimu can divine

Omuzimu gwambulula – Muzimu can do cleansing rituals

Omuzimu gulagula, gugaba eddagala gwambulula – Muzimu divines, gives medicines and performs cleansing rituals

Muzimu is diagnostic and predictive spirit, and can prescribe medications and perform ritualistic removal of bad spirits by performing ritualistic cleanings baths

Balongo

Twins are in many forms or categories.

Some twins are born at the same time by the same mother; Waswa, Kato, Babirye Nakato.

The children that came before and after the twins are also considered twins.

Kasowele twin is the one twin who come out of the birth canal with legs coming first.

Some twins are considered Misambwa, but were entities like animals or waters, produced by a woman coming out of the birth canal before or after a human being. Examples of such entities include Mayanja and Namayanja water body twins (*balongo*) birthed by a woman, that transformed into Misambwa. In case the woman produces a lot of water and then produces a male child the child is a twin Kato while the twin water is called Mayanja if male or Namayanja if female.

Ebisolo oba ebyewalula nga engo ne timba ebizalibwa omukyala biba Balongo - Animals such as leopard or python birthed by a woman, are twins with or without a human child coming before or after the animal.

Emikole jabaloongo mulimu; okusiba abalongo, okwalula abalongo,

Characteristics of ancestral spirits

Empowo zekweka nyo - Many spirits hide their identity. Spirits are secretive and hide their identity because there was time when they were highly demanded to be destroyed. Such time exist today.

Places of spiritual significance

Spirits Misambwa, Mizimu and Mayembe are particular with the places the stay in and work from. Such places are designed naturally or by man to be appropriate for the particular spirits. The places may natural like forests, water bodies, mountain tops or rocks especially for Misambwa spirits while in the wild. However, such places could be well designed and empowered shrines in form of Lubiri, Kiggwa or sabo.

Natural places

The nature places and the power contained their-in are able to identify spiritualists, their powers and abilities, and thus offer respect to individuals accordingly

Forests

Water bodies

Mountain tops and rocky places

Shrines

The process of making a covenant (*okutta omukago)* is involved when empowering a shrine.

Ancestral shrines are headed by a Muzimu which literally owns all the ancestral spirits operating therein.

Shrines are cleaned of any evil spirits, any un-appropriate previous undertakings at the shrines through multiple cleansing rituals (kwambulula) that involve use of particularly identified and

gifted people, mainly from other clans, sacrifice of animals and birds of particular sex and colour, and use of specified plants. The process normally involves family members, especially those connected with spirits as mediums

Shrine location, construction, setting and content is guided by the major spirits of the shrine.

In my case, the cleaning rituals for rejuvenating our ancestral shrine involved all my family members. She spirits helped us to identify gifted people from various clans, more significantly Mbogo clan, Mutima clan, Nkima clan, Nsenene clan, Royal clan, who played specific roles based on their capacities and dressed in their respective regalia.

All participants are dedicated to their roles with a hope that they will be rewarded by the spirits.

The whole process was led by a person possessed by Muwanga spirit.

Lubiri

Kiggwa

Ekiggwa kiwangibwa kumutwe gwamusajja so si mukazi - The kiggwa shrine is built on a man as its leader but not a woman. This type of shrine has its leader as a man who might have ancestral spirits or one without ancestral spirits but if he has inherited three of his grandparents i.e. if he has inherited his father who had inherited his grandfather who had inherited his great grandfather. Its leader can also be its leader, if he is possessed by a muzimu spirit which gives him power to be the head of the shrine. This type of shrine belongs to a family lineage or clan. This type of shrine can be headed by a man who is not possessed by spirits by chosen by the family members in case he is more responsible and accessible than the one who is possessed by spirits.

Sabo

Fire places

Ancient residential houses contained ebyooto (fire places) that served to provide light, warmth and repelled mosquitoes

Traditional kitchen contained ekyoto (fire place) above which was ekibanyi on top of which were placed *amabidde* bananas for making local brew

Shrines contain *ekyooto*. Unlike the fire places in the house and kitchen, the kyooto in the shrine serves additional functions of providing warmth to the spirits Mizimu, Mayembe, Misambwa and Lubaale *to kwoota*. The spirits demand for the kyooto in their shrines, which kyooto the spirits know how best it serves them. Examples of ebyooto in the shrine include; *ekyooto kya Muwanga*, which kyooto is used by many other spirits including Misambwa, and Lubaale.

There are fire places (ebyooto) outside the shrines for particular lead spirits that demand for them. Such spirits and fire places include; Ddungu ne *kyooto kya Ddungu*,

Ekyoto ky gombolola

In a King's Palace, there used to be ekyooto (fire place) in a shrine where the royals used to carry out spiritual rituals and ceremonies which would lead to a peaceful kingdom. A fire place is associated with peace. The Kyoto in the King's palace was called Gombolola and was started on the reign of Sekabaka Kintu at Namirembe Hill that belonged to Nakinsige Clan. The Kyoto Gombolola is where the King used to meet with is subjects. When the king dies, the kyooto Gombolola would stop being lit until a new king is installed for it to light again. This can be likened to the Balubaale. When a mulubaale dies, all his/her powers, Lubaale, Mayembe, Misambwa will stop active working until a new person is possessed by a Muzimu and rituals performed for its powers to work again.

Road junctions

3-junction road (what is the significance?)

Animals and animal parts

What is the significance of an animal Liver in spirituality?

Challenges among Balubaale

Namujinga ayelimbise mubalubaale be bafere nga sibatendeke, sibamanyi, sibabitone, naye nga barina ebiwangiko ebibogerako mumateeka – the challenge among healthcare spiritualists using ancestral powers is the fake, untrained, unknowledgeable and non-gifted imposters yet possessing all necessary legal documents.

Most of our clients are poor peasants in our rural settings although we provide services to all humanity especially the neglected and suppressed.
