# Supplementary material for: Perspectives on health, illness, disease and management approaches among Baganda traditional spiritual healers in Central Uganda
Source: PLOS Glob Public Health. 2024 Sep 6;4(9):e0002453. doi: 10.1371/journal.pgph.0002453 (PMC11379289; doi:10.1371/journal.pgph.0002453)
Supplement: S3 Data — (PDF) [file pgph.0002453.s003.pdf]

Study participant 3 transcriptions

Contents

Study participant 3 transcriptions 1
Socio-demographics 2
Baluntansozi and Balubaale 3
Baluntansozi 3
Mulubaale 3
Mulubaale defined 3
My process of becoming a Mulubaale and the associated problems 4
Becoming a Mulubaale 6
Health, Illness and Disease 7
Health: 7
Bulamu bulungi 7
Embera enungi 7
Omulamu obweyagala (Graceful life) 7
Obulamu obutebenkedde (Harmony 7
Mirembe (Peace) 7
Embeera enungi; 7
Olumbe Illness: 7
Ennaku, / Enyiike; 8
Kubonabona 8
okujanjaba olumbe 8
Obulwadde 8
Causes of problems, illness and diseases 8
Spiritual causes 8
Spirits 9
Natural spirits 9
Characteristics of spirits 9
Ancestral spirits 10
Lubaale 10
Lubaale we Bukojja 12
Lubaale Mukasa 12
Lubaale Musoke 12

Lubaale Kiwanuka 12
Muwanga 13
Muzimu 13
Obuko mu Lubaale 13
Nakayima 13
Kalisa 14
Bamweyana 14
Mayembe 14
Characteristics of Mayembe 14
Okuwanga Ejjembe 15
Jembe Katabazi 15
Namuzinda 15
Jembe Kiwanuka 16
Misambwa 16
Ndawula 16
Balongo (Twin forces) 17
Kitambo 17
Healing 17
Healthcare management 17
Access to healthcare information 18
Diagnosis 18
Ekijjulo – Communal meal 18
Music, songs, and dance 18
Offering treatment and protection, ... 18
Natural places 19
Other research issues raised 19

Socio-demographics

My name is (Study participant 3). I am 61 years old, female, Muganda. I am *omwana womukago*, in that way belong to both Fumbe Clan and Nyonyi Clan. My father in Nyonyi clan is Zakaria and my father in Fumbe clan is Sengendo from Mawokota County, Mpigi district, Kiringente sub-county, Katende parish, Kikondo village. Mother is Najjuma, of Lugave clan. This is a bit confusing, let me explain better.

My original names are (withdrawn) and belong to Nyonyi clan, but now my names is (withdrawn) and belong to Fumbe clan. This is a bit confusing but let me explain better. My mother is (name withdrawn) of Lugave clan and Sengendo is my adapted father. My original

father is (name withdrawn). Mawokota County, (xxx) Sub County, (xx) parish, (x) village. Surprisingly, the ancestral spirits of my adapted father preferred to possess me, even though my adapted father had many other children. It was the spirits that gave me the new name of (name withdrawn) since my adapted father is (name withdrawn). That is how I come to belong to the Fumbe clan. (Omukago is that strong)

I belong to traditional beliefs by religion and I studied up to senior two (S2). I am a Mulubaale, married, with two places of work, one in Kawempe, Kampala and another one in Ddwaniro in Kiboga district, Singo County. My shrine is Sabo. I am a spiritualist healer and a subsistence farmer to feed my family and the workers.

I do not belong to any traditional healers' association. I used to belong to nearly all the traditional healers' associations. However, their aims and objectives, though good on paper were never put to practice. I was not satisfied with the practical reality of the role of the associations. Yet, there was need to belong somewhere in relation to the law. In case anything legal issue comes up, for or against me, belonging to an association would become very relevant. Belonging to an association protects it members and helps them to develop. So, I formed my own association by name of Manyi Gamiti traditional healers' association

The Muzimu which possess me worked in Buganda kingdom palace, however I am nor royal and my spirits are not royal spirits either.

I have Muzimu, Lubaale, Mayembe, Misambwa which make me a traditional healthcare spiritualist. I have many male spirits so I am referred to as *muka basajja* – a lady of many men

There are those who call themselves traditional health practitioners because they heard, copied when in the actual sense they are not traditional spiritual healers.

Baluntansozi and Balubaale

Baluntansozi

Enkola ya Baluntansozi yanjawulo, bakozesa manyi ga butonde, nakyetondeka – The ways of working for Baluntansozi is different, they use spiritual powers of nature contained in mountains, stones, rivers, reptiles and such other creatures.

Mulubaale

Nze ndi mulubaale, nkozesa mpewo zabuzaale - For me I am a Mulubaale, I work using ancestral spirits.

Mulubaale defined

Omulubaale ye mujanjabi akongoja empowo zobujjajjawe – A Mulubaale is healer medium is the person who is possessed by Lubaale ancestral sprits and these includes Lubaale Mukasa, Lubaale Muwanga, Lubaale Musoke, Lubaale Kiwanuka etc Balubaale are the people possessed by *Lubaale w'enyanja*.

My process of becoming a Mulubaale and the associated problems

In many instances I have tried to run away from this spiritual wok but the spirits have brought me back. My ancestral spirits exposed me to America. I went to Boston, Texas, Washington and later came back home to do spiritual work. However, my intention of going to America was to stay in America, although the ancestral spirits had assured me that I could go anywhere but my place of stay and place of work was based in Uganda.

I suffered a lot and my suffering was associated with Lubaale. In fact, I can say most of those practicing *Lubaale* went through some form of suffering before or in the process of acquiring or harmonizing *Lubaale*. There is a song associated with my saying so. **Song:** "*leka abonanone, nafira kumawanga, lek'abonabone, n'asula ejembe lya kitawe" meaning,* let him/her suffer, will die suffering for having thrown away the ancestral protective spirit.

I experienced many problems which were not of my direct making. One time, I visited my friends, who had issues with Police and I was not aware. As I was there, Police came at my friend's place and pick all the people who were at home, including me. All the people were gradually released from the Police except me. The police officers had got interested in me, because of my beauty. I was detained in prison, sexually abused by a Police Officer, and I become pregnant while in prison, over no known case. When I was finally released from the prison, I went to look for a traditional healer to terminate (abort) the unplanned, rather raped pregnancy. I was directed to a good traditional healer who would help me to abort, but when I reached the place, I was told the traditional healer had died the previous week. I then looked for another healer and I was directed to a lady in Mityana. I also found the lady had died.

In the process, the pregnancy stayed and was advancing and becoming obvious. I went to the healer who had given me the medicine to become pregnant and have children. When I reached this male healer, he sat me down in his shrine and counselled me to let the pregnancy stay, since I had asked him for medicine to get pregnant, otherwise I would die in the process. I felt a bit comfortable at this man's shrine and I opted to stay at his shrine until I deliver the child. I feared to go home because I was embarrassed to even mention the person who had sexually abused me to become pregnant. So, I had to swallow everything to myself. In the process while I was at the traditional healer's place and during training of other healers, drumming and singing were taking place, I was surprised that spirits possessed me and instructed me to start my *Kusamira* process at this healer's place as a healthcare spiritualist trainee (*Omutende)*. Finally, I delivered my first child while as a *mutende* at the traditional healer's place where I remained with my child, as a *mutende* for more time.

After some time, while still at a traditional healer's place as a spiritualist trainee *omutende*, I fell in love with an army officer who had come for treatment from the traditional healer's place. Few months later, I realised I was pregnant again. This time I had not completed the training *obutende* so I stayed at the healer's place for time, both training, breastfeeding the first child and carrying the second pregnancy. Finally, I delivered my second child.

I spend six (6) years in *butende* at the traditional healer's place as a trainee *omutende.* I had two (2) children from Security Officers during that period. Remember I originally went to a healer's place to look of pregnancy, she ended up in 6 years training in healthcare spirituality but also got two children within that period 1983.

I harmonised the Lubaale and its spirits and I was finally was released from the traditional healer's place to start working on my own as an independent healthcare spiritualist.

I become very popular in African spirituality. I want you to note that I had two children from two security officers, (Police officer and an Army officer). Both officers were each interested in his child yet none of them could blame me for the children. So, I ended up as a traditional healthcare spiritualist, having two (2) security officers, known to each other, in my house who could come and go without any quarrel or problem.

That was translated into my being a very good spiritualist who could control two Security Officers to stay in one house controlled by me a woman. In that way, many women come to me for such abilities to control men. I also took advantage of these two security officers to handle any Police and Security cases anywhere in Uganda. Many people with any Police or Court Cases, came to me for a solution. This worked very well for me. Any case would be very well investigated, if it required the army force, it would be available, on addition to the spiritual influence. Both husbands, would financially benefit in this process so they were fine with me. Much of their interests were their children but they were also getting money in the cases I would forward to them. All this was done in Kampala so I specialised in and was popularly known for Family and Court issues.

I become known as a spiritualist on account of what I was able to do. (1) The people I worked upon are the ones who made me a popular spiritualist in the eyes of the public basing on what they talked about me in front of others. (2) My spirits could possess me, talk for themselves and offer healthcare services that worked successfully. "*nga ndiko amanyi gobujjajja agajanjaba obulungi"*

For one to become a healthcare spiritualist, that person has to be with the qualities of interest to ancestral spirits _Lubaale. (*Lubaale no Muzimu tebabigula).*

The participant advised me to be very careful during my research, of the people who impersonate and claim to be spiritual healthcare spiritualists. Some are only imitators, they copy what they have seen being done, and they try to do it and claim to be spiritual healthcare spiritualists. A spiritual healthcare spiritualist should is selected by the spirits, taken through various forms of trainings, passed out and then assisted by the spirits to manage the clients. The spirits are normally nearby to assist or even take over and control the process or even invisibly participate in the discussion or explanations.

We, the healthcare spiritualists are knowledgeable and our healthcare activities are guided by ancestral spirit. There is diversity in tribal and cultural spirits that should be taken not of.

Some of my spirits here only need to hear what I want to be done, and they will lay out the plans to put into reality what I say or think.

Even when a client talks to me on phone, the spirit will hear our conversation and do the needful to the satisfaction of the clients. That is the beauty when the spirits are all well harmonised.

When a client comes to me, I call upon my spirits to come and guide me in the process. So do not be surprised when my spirits possess me to address most of your questions and concerns.

It is important to take care of the welfare of the spiritualist.

In my practice I use various methods to access healthcare information. At times I need to drum and dance especially for newly expressed spirits. I also use local brew for the spirits that need local brew. Some spirits need fire and for some I roast meat to access the information while

other spirits are comfortable with consultation or commitment fee (*ekigali)* for the information to flow. On addition I use the knowledge already acquired.

(we reflected upon; what is the difference between ***Ekigali and Amakula***?) (How are spirits controlled? Who do spirits account to? How are spirits accountable?)

In the continued talk she reflected that Spirits are self-regulated, although the spirituality of many people is in captivity. (*Lubaale wabantu bangi ali mu buwambe)*

Becoming a Mulubaale

Like most spiritualists I was involuntarily forced into spirituality and healing. I started my healthcare practice in 1987 which is 33 years of practice now.

By basic standards, I was a beautiful, attractive and rich young woman, but I failed to get a child or even to get pregnant. I tried all the Western medical interventions and good medical doctors but all attempts failed, so I tried traditional healers finally I got one healer who gave me the medicine and assured me that I would get pregnant and have children.

Buli asamira agenda mu sabo - everybody undergoing Kusamira process should go to a shrine because the shrines are made in ways that empowered them (*Obuwange bwe sabo)* to accommodate, cool down and tame the spirits in an attempt to harmonise them. When the spirits are called and they come and possess a person, there are those other spirits that must be present to inform, educate and discipline these newly expressed spirits. Such spirits are already harmonised within the shrine. Lubaale has a suitable place and is enshrined in the shrine, but not is a human residential house or home.

Some of my spirits had a significant role in the King's Palace. Like which spirits? By name. It is important to note that the spirits of the People who are Royals (*abambejja n'abalangira)* and the people who worked in the Royal places (*abakolanga mumbiri)* are different and play significantly different roles. There is a saying (*Okutambula n'abalangira, tekikufuula Mulangira)* one does not become a Royal by associating with Royals.

Spirits have me men three times until now when they have taken me from all those men. I now belong to the spirits, and they are the ones looking after me.

Spirits such as the Mizimu, Lubaale, Mayembe and Misambwa in the end make up a traditional healthcare spiritualist.

In preparing me for this work, the spirit made me go through tough moments especially in the family and court fields where I finally specialised. I went through difficult family life in her childhood and young adulthood, and this prepared me very well for handling and settling family life. I was also taken to prison three-four times, but all those times, the spirits made wonders in the process of getting me out of court cases. In process I was prepared very well. The best way to prepare a human being is to take them through a similar situation and then get them out of such in unbelievingly surprising ways.

What I learn from all this is that the special healthcare knowledge and skills we acquire may change while being transmitted from our mentors and trainers to the trainees within generations but the essential principles remain the same.

Health, Illness and Disease

Health:

Bulamu bulungi

The one who breathes in and out is the one who is alive. (*Omuntu assa yaba omulamu)* - *Omuntu bwataba mulamu aba mufu.*

Embera enungi

If you happy, joyful can eat, can breathe

Omulamu obweyagala (Graceful life)

Obulamu obutebenkedde (Harmony

Mirembe (Peace)

Embeera enungi;

Olumbe Illness:

Obulamu obutali bulungi,

Olimbe lukwambala bwambazi

Olumbe luyingira omubiri naye telutula munyama yomubiri

Olumbe bwelulwa mumubiri, lutandika okwonona enyama yebitundu byomubiri nebitandika okulwala – olwo ebimu kubitundi byomubiri gulwaala negufuna obulwadde

Kitambo can be able to cause *olumbe.* Every family has *ekitambo.*

Seyagala,

Sewulira bulungi

Ennaku, / Enyiike;

abaana bange banfuddeko,

Obulamu busigala bulamu. Jaja bulamu ali omu- Bulamu in one and there is a spirit called Jaja Bulamu.

Everyone is healthy until he/she declares otherwise.

Somebody is the one who makes his life ill.

Kubonabona

okujanjaba olumbe

okujanjaba olumbe by spirits varies with the specific spirits involved. There are specific spirits known for addressing infertility, luck, richness, stabilizing homes, dealing with court cases.

It is not true that a traditional healthcare spiritualist will deal with everything, no. However, I am multiple functioning especially that I have many spirits that are responsible for the various tasks and duties.

Obulwadde

A person is the one who brings obulwadde, because he is the one who says "ndi mulwadde" then one asks "kiki ekikuluma" Life starts getting a problem. Some one can say "*setegera bulungi*" when on the looks he is not sick but at the end he dies.

Obulwadde bukwata ebitundu bye nyama yomubiri nga omutwe, amaso, enyindo, amanyo, omusaayi, ekibumba, ensigo, enda, mutima, mawuggwe, magulu, mikono nebirara bwebityo. - Disease affect the body parts or organs like head, eyes, nose, teeth, blood, liver, kidneys, intestines, heart, lungs, legs, arms, etc.

"Obulwadde affects an individual body even when it affects many people. An example of obulwadde include HIV/AIDS, obulwadde bwa Kawumpuli (smallpox), Measles (obulwadde bwa Mulangira)

Causes of problems, illness and diseases

Spiritual causes

Songs used to bring out meanings and explain certain situations. *"Leka obonabone newerabira ejjembe lya kitaawe leka obonebone"* meaning let him suffer for having neglected his father’s Jembe, let him suffer. Such songs are, at times, directed to the spirits which hear, get the meaning and respond appropriately.

Spirits

Spirits are not confined to a shrine. There are spirits associated with swamps, mountains and many other places.

Spirits collaborate and cooperate just as human collaborate and cooperate. Spirits through their medium may talk to a client and refer to a client by a name of a spirit, in which case the spirit of the medium is calling for the attentive presence of the spirit within the client, and being addressed or informed.

Spirits use words that are known and familiar to specific spirits so as to attract presence of those particular spirits or for certain spirits to perform specified tasks. For example, the spirit medium says Gayira-gayira, all water spirits, such as Mukasa, Kiwanuka, Kadduwanema or Musoke, will be attracted near and become attentive to the words or the conversation that proceeds. If the water spirits were responsible for the illness within the client, and are attracted by the words said by the spirit medium, express their concerns to the spirit of the medium. The water spirits may be persuaded to lessen their pressure or even leave the client for him/her to get better, just through the use of words

Spirits vary in their abilities and methods of work. Some spirits are natural while others are ancestral.

A person with spirits gets extra protection when the presence and expression of his/her spirits are attracted.

The presence of the spirits provides extra abilities to know and understand to spirit medium or host. Spirits use words that need extra abilities to understand and follow the conversation which only people with spirits may be able to follow. For example, the spirit may tell a story to its clients but the story will be understood only by those who have their spirits nearby. The content and context of the story will be missed by the rest of the people without spirits.

Natural spirits

Muwanga Ssebyoto is a natural spirit and is without a Muzimu

Natural spirits do not have a Muzimu in their formation

After the formation of natural spirits, there followed the spirits that were produced as human and the subsequent reproductions

Characteristics of spirits

Every spirit has its own place where it dwells. When human beings change their ways, the spirits also changed their ways. As a saying goes; *enswa bwekyusa amaso nga nawe okyusa envubo.* The spirits have changed their ways to fit with the changing world.

Even if the spirits cannot read English, they employ those who can read and they listen. The spirits have the elite in the present generation who can read and interpret to the spirits and they listen and know what is transpiring. After understanding what is taking place the spirits also converge and decide on the way forward regarding that particular issue. Spirits are instructed

to look for those human beings who can be instrumental in a particular issue. You might be sleeping and you dream to go somewhere where you didn't intend to go. An individual person is forced to do what the spirits want due to the powers of the spirits.

Spirits work for all the people. A healthcare spiritualist work for all the people. Spirits and the healthcare spiritualist are not, and should not be selfish the way current people are who work for their families alone. When the spirits possess a spiritualist, they want the spiritualist to work for people (referred to as grandchildren). So, the spiritualist do what the spirits want.

The spirits choose their own mediums and the work very hard to protect them.

Some spirits dwell in natural places within the environment. When the environment is destroyed, the spirits, like spirit Mayanja, get displaced and get angry. Spirits multiply and become many. The off-springs of the spirits no longer have space in the encroached environment so currently the move haphazardly and collide with people, leading to accidents. This leads to many things to happen that human beings cannot explain, but can be attributed to distorted culture, cultural sites and the embodied powers. However, the spirits will ensure their existence in the future generations. The spirits will fight for their survival up to the end.

Some spirits are associated with specific sites in Buganda. Spirit Walumbe is found Tanda a Cultural/spiritual site.

Each spirit has its own specialities.

Ancestral spirits

spirits are particular about their characteristics such as dressing code, eating modalities and behaviour (*eneyisa).*

Lubaale

Munono ya Baganda, Lubaale owokunono alimu; Abalangira, Emisambwa, Amayembe, n'abaana/abalongo. – Lubaale constitutes Abalangira (Royal spirits), Misambwa (Spirits with natural forces), Mayembe (Support and protective spirits) and the twin spirits. (note; Muzimu is not part of Lubaale)

Lubaale ow'enkuliiti ye Lubaale owensikirano – Lubaale wenkuliiti is the ancestral Lubaale over generations.

Abalangira (Royal spirits) include; Ndawula, Kawumpuli, Bamweyana,

Misambwa emizaale (ancestral misambwa) include; Mukasa, Kiwanuka, Musoke,

Lubaale belongs to a clan and not easily transferable between clans.

Buli Lubaale alina abaselikale - Every Lubaale has its own security.

Lubaale yegenderezanyo - Lubaale is very careful in all its undertakings

Lubaale asibirwa enkanamu -

Lubaale tebakolamukole, lubaale ye waali yebangira dda. Lubaale is not just fabricated, Lubaale existed by its own long time ago. *Lubaale basiba musibe enkanamu ze.*

Song: *"mwanguwe enkuba tebakuba, mwanguwe 'abaana ba Lubaale Muwanga'"* meaning that all Lubaale, including Mukasa, Musoke, Kiwanuka are children of Lubaale Muwanga. This means that Muwanga is Lubaale.

The ways Lubaale works cannot be explained fully by a human being even if that person can be possessed by Lubaale. When Lubaale possesses a person, it mentions its name, talks it purpose for coming and gives instructions for what should be done, how and why.

The spirits have many intentions to work for their grandchildren (*abazukulu*). Every human being on earth is the spirit's grandchild. (*bulimuntu muzukulu w'empewo).*

Lubaale is engaged in fighting wars at the instructions of their Muzimu and God. Lubaale advises its people how to overcome their challenges. Lubaale controls one's life.

Buli muntu assa alina Lubaale we, nga bwalina abazadde – Every living person has Lubaale, just as every person has parents.

Lubaale chooses the person to be its *mukongozzi* medium after careful considerations. Lubaale may attempt many people before finally settling on any one person to act as its medium.

Lubaale works to feed, protect and dress its *mukongozzi* medium and in the process the *mukongozzi* medium see the value of Lubaale and work for it.

Lubaale already exists and it is only harmonised. *"Lubaale basiba musibe enkanamu ze kubanga ye wali. Tebakola mukole ye jjali. N'amanya jegali ga nanziri, namukasa, nalwewuba, ago gonna manya gabalubaale bakazi ba Kabaka Mukasa."*

Lubaale possesses a human being (*Lubaale akwata muntu*) without which Lubaale will not exist. When a person gets possessed by Lubaale, Lubaale takes over the control of the mind of that person. Lubaale selects by itself the one who will be possessed and be its custodian (*Lubaale alonda anaaba mudiiro).* Each Lubaale has its own way of working, it assigns duties to different deities as it wills. Each Lubaale has different working spirits that include, Mayembe, Misambwa and other spirits. Lubaale is always with its *Mukongozzi (Lubaale tavaawo, otambula nomukongazzi*). So when talking with the spiritualist, the Lubaale is by the side listening and at times as the spiritualist is talking, the Lubaale may take over the conversation and directly respond to you with or without your knowledge. In that way, the Lubaale may tell you the truth or lie to you in case your intentions were bad or to protect its secrets.

Lubaale omukulu tagwa, lubaale omuto y'agwa. why? This is because when Lubaale is just manifesting on an individual, the individual is hesitant and is not yet set to receive and accommodate Lubaale and the Lubaale has not anchored properly on its host. There is a fight between Lubaale and the person being possessed, who falls down in the process.

Lubaale, like lakes and water, is one. Therefore, all spirits from all walks of life are the same. *Lubaale ali omu, nenyanja eri emu amazi gali gamu*. The trees are similar, the grasses are the same but because of different ecological situations they differ in names. Lubaale has existed many centuries., Lubaale was not created or made. Lubaale is very slow in its way of working

Lubaale we Bukojja

When *Lubaale we Bukojja* possess a person, the possession is short lived, and goes off and stays a side.

"Nyoko abanga omunyoro nakuzala kukika" meaning that it is better for you if your inferior mother had your father from a superior clan. Many people who were brought to Buganda as inferior slaves (*baddu)* produced their children with 'superior' Baganda and so their children and grandchildren were Baganda.

That lubaale is not the owner of the head *(lubaale we Bukojja si yenanyini Joba*)

Lubaale Mukasa

Lubaale Mukasa is the king of Lubaale (*Kabaka Mukasa ye mukama wa Lubaale yena), Lubaale Mukas* has many (9) wives. His main base is in Bukasa Sese. *wano mu diiro Lubaale Mukasa yasinga okubera nga akebera* - Here at my shrine, Lubaale Mukasa is the main spirit that carries out diagnosis. Lubaale Mukasa provides luck (*mukisa/mikisa).* There are two types of *Mukisa; omukisa omuzaale n'omukisa omunywere eddagala).*

Lubaale Mukasa is a water spirit, originally from Sese Island, at place called Bukasa. Lubaale Mukasa is not usually involved in causing sickness nor healing. It normally sends other spirits. in my case Lubaale Mukasa sends Mayembe, Kiralire, Kagorokakyomya and Misambwa to effect healing.

Lubaale Mukasa does diagnosis (*okebera*) and is very thorough in explaining. Can give detailed explanations for diagnosed health conditions.

Lubaale Mukasa provides, improves, clears one's luck, improves one's ability to get and it gives luck. *Lubaale Mukasa agaba Omukisa, no kufuna.*

Lubaale Musoke

Lubaale Musoke *asobola okwonoona*

Lubaale Musoke is concerned with ladies health conditions (can improve or worsen them). Lubaale Musoke may be responsible for threatened abortions or even for stopping any abortions.

Lubaale Kiwanuka

Lubaale Kiwanuka ow'enyondo (is responsible for thunder and lightning)

Lubaale Kiwanuka akozesa omuliro (fire) era akomba ku Nkanamu.

Muwanga

Muwanga has two (2) types namely; Muwanga womunseke ne Muwanga wa Kaliggwa: (Enissoso gya buli kika kya Muwanga gyanjawulo; Ensiba, enkola, endagula). The main job for *Muwanga* is to *Kuwanga n'okuwangulula.*

"mwanguwe enkuba tebakuba mwanguwe, abaana ba Lubaale Muwanga mwanguwe" This song indicates that Muwanga is a superior Lubaale and is the one who instils power in other spirits.

Muwanga we Nseke is the main/major spirit for my shrine set up. My Muwanga works best under the influence of alcohol. We Muwanga appears, he asks for his alcohol and after drinking alcohol, them business begins. Muwanga we Nseke likes working companion spirits that drink alcohol like Bamweyana. Muwanga we Nseke is very articulate and serious after an alcohol drink. I personally enjoy alcohol, so I am happy working with my Muwanga who drinks alcohol. Muwange we Nseke, is not rigid and easily adapts to the changing circumstances.

The role of Muwanga in any set up, *Sabo, Kiggwa or Lubiri* depend on his role in the clan. For different clans, Muwanga together with other major spirits like Mukasa, Kawagga, etc., will assign a spirit that will be taken as the major spirit for most of the shrine activities

Muzimu

The word Muzimu stems from the word Muzzi which means "Just Comes".

It is the Muzimu that knows its owner "Muzimu gwe gumanyi nyinigwo".

The Muzimu knows the weakness of its owner and knows when to request for assistance

Luyimbazi is a male *Muzimu* spirit that possesses me. Muzimu Luyimbazi is the main spirit that owns our Lubaale and is the one that assigns duties to other spirits. Lubaale is a property of our family and is part of the Clan Lubaale (Lubaale we kika)

Muzimu works closely with other spirits *abatabaazi, abasambaganyi, abamanyi abakozi b'emirimu.*

The Muzimu that possess me used to work in the King's Palace in the security section responsible for controlling and sieving whoever goes through the gate of the palace.

Muzimu is originally the owner of Lubaale. It is the Muzimu that possess the lubaale.

Omuzimu tegulya mere - *t*he Phantom do not eat food.

Obuko mu Lubaale:

Lubaale asobola okufuna obuko. Kyekyo lwaki lubaale webukojja bamwawula nebamuzimbira enyumba ze wabbali nebamwawula ku Lubaale w'ekika.

Nakayima

Nakayima is a female spirit.

Kalisa

Kalisa is a male spirit.

Bamweyana

Bamweyana is *mutamizu, mwenzi, ayagala nyo okulwaana, mulalu. Bamweyana mukozi* and knows medicines for many ailments, issues and cases.

There was a misunderstanding between the royals whereby the female royals (Bambejja) demanded for equality to contest with their male royals (Balangira) for the seat of Kings. The situation become so hot that the matter was referred to Kiwanuka for a solution. Kiwanuka appointed Bamweyana as acting but with stun and harsh instructions to behave in madman ways which he perfectly did. Kiwanuka and Bamweyana had agreed that Bamweyana would not be held liable for whatever would go wrong nor would he be blamed. However, whoever would follow they ways of Bamweyana would be held responsible, bear the blame in their own individual rights and be implicated individually.

Mayembe

Mayembe are of various types; "amayembe gobutonzi" Natural Mayembe, (amayembe gekika) ancestral Mayembe spirits, and "amayembe *amagule".*the Mayembe acquired by an individual for a particular purpose

M*ayembe* spirits are mainly individualised and can be acquired by an individual who can transfer, lend, loan out or give part of his/her Mayembe spirit to any other person anywhere and at any agreed terms.

When the individuals completed Lubaale harmonization, they contemplated upon how they were to protect their Lubaale. Then indivudauls were to Walusi hills in Luwero district and derived (*okugya*) the Mayembe mainly to; protect Lubaale,

Teli Jembe liwongerera Lubaale (lyambaza Lubaale bifundikwa) – Jembe does not knot and dress Lubaale its knotted backcloth, because Jumbe is only brought to protect Lubaale.

Muwanga and Kawumpuli boka bebawongerela Lubaale owe nkuliiti -It is only Muwanga and Kawumpuli with the authority and capacity to knot Lubaale and Muzimu.

Any current spirit medium may at any time acquire his own Mayembe for any particular given purpose. – *Omukongozi wa Lubaale asobola okufuna (okugya) a Mayembe esawa yonna.*

Characteristics of Mayembe

Amayembe gawangibwa era gawangululwa

Amayembe *gapapa nyo*

Amayembe Luyizz, Kayizzu,and Muyizzi are different and may serve different functions

Mayembe are originally owned by Muzimu.

Mayembe are fabricated by a combination of pieces of plant and animal materials in order to serve specified functions, like; to work (*kukola*), providing for bearing of children in the family (*Kuzaala*), looking after and upbringing of children in the family (*Kulera baana*). Protection/Security (*Kukuuma*).

Some Mayembe spirits are thieves, like Jembe Kisomolo. Jembe **Zizinga** is known for causing conflicts. When such Mayembe spirits, like Jembe Kisomolo and Jembe Zizinga possess people, such people may behave as thieves and cause conflicts among people. Such people need to be helped by calming down such spirits.

"Amayembe, magezi, makujukuju ate massi" meaning Mayembe spirits are very clever, wise, and can kill.

Mayembe are helper spirits. Amayembe m*atabaazi, masambaganyi, gamanyi, ela makozi g'amirimu*. Mayembe spirits are mainly individualised and can be acquired by an individual. The individual owner of the Mayembe can transfer, lend, loan out or give part of his/her Mayembe spirit to any other person anywhere and at any agreed terms.

Mayembe are very fast in their actions and easily make friends with other spirits especially fellow Mayembe spirits. One may task one Jembe spirit, but in a short while, that one Jembe gets many others to help it in the given tasks.

Mayembe are free and careless in their talking. They can talk anything without respect, fear or favour

Okuwanga Ejjembe*:*

Amayembe bagawanga and bagawangulula (okugapangulula)

Jembe Katabazi

My Jembe Katabaazi is more instrumental in court cases

Namuzinda

Namuzinda is a Jembe with capacity to handle very difficult cleansing rituals – *Omulimu gwa Namuzinda kugogola.*

Namuzinda spirit is a good counsellor and trainer to both the spirit medium and other spirits. to sustain ancestral spirituality for many years is to keep it in its original forms. Please do not make any additional spirits to your ancestral spirituality, Namuzinda advised the spirit medium.

It is possible to do your ancestral work and continue very well with your earthly work including academics and employments if both are given concentrated time.

In my shrine, Namuzinda spirit is the chair-spirit of the gatherings involving spirits and humans. So as a chair-spirit, Namuzinda has more presiding powers during the gatherings.

Amayembe go mulubaale ga kumutwe, era ga nnono. - The Mayembe spirits of a Mulubaale following traditional cultures use a human spirit medium to verbalise.

Amayembe gatambulira wamu ne Lubaale. Mayembe move together with Lubaale.

Omuzimu gukozesa olukaayi okujanjaba – Muzimu may use *olukaayi* when offering treatment.

Okukaayi is composed of Yirizi ye Mpologoma, endege za Mukasa, Kiwanuka, Musoke, Bulamu, ekide kya Ddungu ne Misambwa.

Mayembe in their traditions do not require a special shrine, instead they work together with Lubaale. *Amayembe genono tegasaba nyumba yaago.*

Amayembe gakola ne Muwanga, Kawumpuli, Lubaale we Nyanja, abalangira ne Misambwa – Mayembe work with all the other spirits including Muwanga, Kawumpuli, water spirits, the royal spirits and Misambwa

Jembe Kiwanuka

There is a spirit called Kiwanuka which is *e Jembe*

Misambwa

Misambwa are mostly found in mountains and rivers. Some people only work with Misambwa from mountains are called *Baluntansozi* and their ways of working is different from *Balubaale*. People with Lubaale are called *Balubaale*. The *balubaale* work with Misambwa on addition to their Lubaale.

The origin of some Misambwa is related to human beings who had mysterious disappearances, but left persistent impression in the communities.

I have several male Misambwa spirits. I believe that my male spirits are the ones who selected for me the men who fathered my children. I have had my children with three men until now that the spirits are in full control of me. My spirits have taken me from all men.

Misambwa stems from Misangwa.

Musambwa connects the human soul to other spiritual entities

Misambwa are protected by Mayembe

Ndawula

Ndawula as a King Spirit, does not get involved in making diagnosis. (*Ndawula Kabaka, talagula). Ndawula spirit* Is normally available only on call. Has no specified routine role, but always aside and available when there is need for him to be consulted for some guidance. The king does not work, instead people work for the King (*Kabaka takola, bakolera bukolere).*

The shrine, fireplace or the environment for Ndawula contains fresh Banana |Juice (Mubisi)

The spear of Ndawula is smeared with cow-gee (Omuzigo).

Balongo (Twin forces)

Abalongo benina abazaale mukika. The twin forces I have are ancestral in my clan.

Abalongo are either born by a woman (*bazaale*) or they are natural *batonde*. (*Abalongo babeela bazaale oba batonde*). *Abalongo abatonde* are mostly found in kingdoms (*mubwakabaka*).

The balongo also belong to the Muzimu.

In Baganda, an albino is a twin. When the Africans produce an **albino** he is quickly soaked in alcohol so that he does not resemble the whites so much. When that is not done, the albino is so pale and the houseflies follow him so much in that way becoming an outcast.

Ancestral twins are protected by Mayembe.

Most rituals are started by calling upon the presence of the twins and twin forces.

During *Kusumikira*, twins and twin forces are first called in to be present.

At the inception of twin basket (*ekibo kya balongo*) that I witnessed, various items were placed into it by Kawumpuli spirit while saying words of meaning (*kulamiliza*) that included; coffee beans (*Katamukago*) and said "*kano kekabonero akalaga nti muliwamu n'omuzukulu wamwe* (name)" – translated as "this is symbolism that you are together with your grandson (name); Matembe (English name) *okumutembeseleza ebilungi okuva olwalero* - to signify upholding the best for him and his family, all the time, beginning now onwards, including good life, riches, being loved and preferred, leadership roles, and having a good tongue towards his listeners. She placed in cowrie shell (*ensimbi*) saying this is a symbol of riches your rich ancestors and she requested the riches to stretch to the children and grand-children. , ;

Kitambo

Kitambo is a spirit found in every household. Kitambo spirit is not as bad as is commonly considered. When not well harmonised, Kitambo can turn the family into a nuisance as *basezi (*night dancers). Kitambo is associated with walking naked at night and eating human fresh. However, if well harmonised, Kitambo is very good for the family. It is the source of riches that can last for generations.

Healing

Beliefs are the basis of healing, Once I strongly believe in something, what I believe in contributes a lot to the healing I offer.

In my traditional healing practice, I am aware that the client's belief contributes to his/her healing. So, I take effort to understand what my clients' belief in.

Healthcare management

In Lubaale, healthcare management is associated with powers of the lakes, rivers, swamps. The rivers, lakes and swamps have their own powers that are tapped upon in healthcare. Some rivers

are naturally created while others are delivered by female human beings such as river Mayanja, and river Sezibwa. Mountains have their spiritual powers and Lubaale has its own powers

It is not that every spirit will do everything, different spirits are good in different aspects. Lubaale may be potentiated in its functions by use of things the spirits are interested in such as alcohol, smoking pipe and its ingredients like tobacco, milk or use of ghee. Some spirits do heal, while others do protect the shrine work place.

Access to healthcare information

When Lubaale is harmonised, it helps in getting its people informed through dreams. Through dreams, one is informed of what has happened and what will happen. Trainings of traditional healthcare spiritualist is gradual through dreams, letting things happen as one dreams them. If Lubaale is not harmonised, one may fail to receive information through dreams.

Our healing knowledge is inherited and intangible heritage which is framed according to the practitioner and his or her trainers. This means that this is not cram work. Information that flows is for particular individual clients and no similar information or manifestation are identical.

Diagnosis

The diagnostic process of Lubaale is very variable. Some Lubaale may make diagnosis based on use of a common diagnostic tool (*omweeso).* Other Lubaale may diagnose using water, palms, coffee beans, etc. It is worth noting that the diagnostic process uses the tools, the powers embedded in the individual (*amanyi n'obuyinza)* and the experience got overtime.

Ekijjulo – Communal meal

Ekijjulo is a communal meal. Ekijjulo is a ritualistic activity that brings together different kinds of spirits.

Music, songs, and dance

Songs form a significant part in education and informing spirits. When training, the spirits informed, corrected, disciplined by the Senkulu through actions where the Senkulu brings in a relevant song to which the spirits listen, click to the content of the information and corrects itself appropriately.

Offering treatment and protection,

When human beings transform into spirits, their spirits can make things happen. The transformed spirits of human beings may move at night and treat a person through scarification on the person's body, on waking up in the morning the person will notice that he/she has scarification marks on his/her body, which were done by the spirits at night when she/he was sleeping.

At times spirits move through reptiles, like the snake, to inoculate some antidote into a person's body to prevent some illness or witchcraft. So,rrrrdddddz a snake will bite a person, but will not cause any serious effects upon the body, apart from panic. Some spirits move within the fierce animals to use them to effect the spirits plans

Muzimu is a spirit. I believe in the spirits which enable me treatment and cure my clients. When a spirit instructs me to do something that is what I will do. I will not accept another person’s instructions, otherwise I would be in trouble. Similarly, when my cleints hear the instruction from my spirits, they will not accept to divert from those instruction because of the beliefs my clients have in my spirits which have a good history of effecting cures.

Natural places

Each natural place, such as mountain, rocks, rivers, etc has its own unique energy or powers that can be tapped into for healing.

Each of such places have their own names and if followed well such names have roots of meanings.

Some of the water bodies like rivers, are nature yet others were delivered by women, such as rivers Mayanja and Sessibwa in Buganda.

Walumbe spirit has its origin found in Tanda cultural/spiritual site.

Other research issues raised

At times as we, the researchers, were talking to some respondents, their spirits would come and take over the conversion and give us more details. The questions we had for ourselves were how convincing will it be to the scientific community that we talked with the spirits on the human being instead of the human being himself or herself? As I was still reflecting over that, the spirit continued. If I may talk on behalf of other spirits, we the spirits can talk for ourselves about ourselves. Now that you have come to know about us, then I found it more appropriate for me the spirit, to come and explain about myself and make you understand myself. (Is talking to the spirits permissible in research?)

The spirit while possessing her (name withdrawn)) told us that; “when you request for the spiritual information from the people who get possessed by spirits, there is a big assumption that they know about us "the spirits". At times that assumption is not always true. Some do not know about us the spirits and I would suggest that you get the opportunity to request to talk to the spirits when the spirits are possessing the individual. You will then get good and first-hand information. For example this (name withdrawn) (The one we possess now) does not know, it is us who possess her that know why and how we possessed her. She actually follows my instructions, whether she likes it or not. I, the spirit, have an agenda to accomplish, and I will do it while possessing her (Justification why talking to the spirits is important and scientific)
