# Supplementary material for: Perspectives on health, illness, disease and management approaches among Baganda traditional spiritual healers in Central Uganda
Source: PLOS Glob Public Health. 2024 Sep 6;4(9):e0002453. doi: 10.1371/journal.pgph.0002453 (PMC11379289; doi:10.1371/journal.pgph.0002453)
Supplement: S4 Data — (PDF) [file pgph.0002453.s004.pdf]

Study participant 4

Contents

Study participant 4 1
Consent form 3
Socio-demographics 3
Acceptance rituals 4
Buganda and Bunyoro ties 4
Becoming a traditional healthcare spiritualist (both a Mulutansozi and Mulubaale) 4
Training 5
Final examination and pass-out 6
Shrines 6
Muwanga's shrine (Essabo lya Muwanga / Ekiggwa kya Muwanga 6
Sacrifices and offerings 6
Animals and birds' sacrifices 6
Offerings 7
fruits offerings 7
animals and birds' offerings 7
Sources and access to healthcare information 7
Sources of healthcare information 8
Access to healthcare information 8
Words and phrases that describe health, illness and disease 8
Words and phrases that describe health 8
Words and phrases that describe illness 9
Ekisirani; 10
Ebbanja mu mpewo 10
Words and phrases that describe disease 10
Healthcare using ancestral spirits *Okujanjaba kwo Mulubaale* - 10
health management using Misambwa - *Enzijanjaaba ye Misambwo* - 11
Health management using words 11
health management using herbal materials 13
Health management using other sources of healing powers 13
The Sun, moon and stars 13
Health management (prevention, protection, health promotion) 13
Kuwanga 13

Scheduled communal rituals for health management 13
Spiritual cleansing (okwambulula) 14
Causes of problems, illness and disease 16
spiritual causes 16
causes as punishment 16
Management of illness due to spirits 17
Payments for healthcare services (Fees/Costs) 17
Spirits (myooyo) 17
Lubaale 18
Kadduwanema 19
Mukasa 19
Kiwanuka 19
Musoke 20
Muwanga 20
Muzimu 20
Misambwa 21
Ndawula 21
Bamweyana 22
Kawumpuli 23
Nakayima 23
Kaliisa 24
Mayembe 24
Kanoonya 24
Katabaazi 24
Kasajja 24
Balongo 25
Walumbe (Kabaka Bulamu) 25
Royal spirits 25
Shrines 26
Amasiro: 26
Lubiri 26
Kiggwa 27
Ssabo 27
Natural places 27
Nyiize 27
Walusi 27

Sexuality in spirituality 27

Regalia 28

Backcloth 28

Witchcraft 28

Consent form

I presented the consent form and read it out loudly to the respondent. The study spiritualist held the consent form, placed it in the basket (*kibbo*) over-night for the spirits. We all spent the night in the shrine. The following day, the responding spiritualist told us that the response was positive and he had the permission to look at and carefully read for himself and understand the consent form thereafter he would sign. However, he advised us to continue with our work even before he signs. On another day, he come with the consent form and signed it in our present. The responding spiritualist told us that his spirits would be around throughout the research study period to give us the needed information

On the third day we were taken through an acceptance and ritualistic orientation, that involved an overnight stay in a forest, a white cow, a black calf, Pressure lump and Cereals. We were taken through detailed ritualistic prayers after which the respondent declared to us that he was permitted and free to talk to us to any details.

Socio-demographics

My name is (name withdrawn). I was born Muslim but practice traditional religion. I am a male adult, 63 years old and belong to Mbogo Clan. I completed A' level (Senior 6), and studied Mechanical Engineering. I have 30 years of experience as a traditional healthcare practitioner and I am a substance farmer. I am married with many children

I have other names given to me (removed) given to me by virtue of my position and the powers embedded in me.

My tribe is Muganda (Ganda) and my parents are both Baganda. It is important to specify my tribe because it identifies me from other tribes. We have many tribes and "*Buli gwanga n'ebyaalyo*". The role of the Mbogo clan in the Buganda Kingdom is *Bakongozzi ba Bakabaka:*

I am in Busiro county (Saza), Wakiso district, (xxx) sub-county, (xx) Parish, (x) village, in Buganda (Central Uganda). My father is (name withdrawn) of Mbogo Clan and his mother is late (name withdrawn) originating from King Kagulu Tebuchereke in Busiro County. His maternal grandparent is (name withdrawn) used to keep the gate to the Kingdom Palace.

I belong to Uganda N'eddagala N'obuwangwa Bwaffe Traditional Healers Association in order to have a belonging and to advocate for traditional healing as one voice with others

I have many shrines here, the main one is called Muzanganda

Acceptance rituals

At about 10.00 pm, we were taken to sacred place, thicket, down the valley about 100 meters from the Major Shrine. The thicket is comprised of a decomposing old big tree trump covered with pieces of cloths. Under the thicket are *Ebyanzi* (vessels for milk), fruits, and eggs. In the upper and left vicinity are four fire places that belong of the spirits of Kalisa, Ddungu, Nakayima and Bamweyana. On the right lower side is a water well beneath the thicket. On the left lower part is the relatively small shrine belonging to Kalisa. We performed ritualistic processes until 2.00am that same night of 19th July 2019.

Buganda and Bunyoro ties

The respondent narrated to us the story on how the Mbogo clan came to be *abakongozzi ba* Kabaka**. Story):** It follows war period where the Royal Buganda Kingdom got wiped out and many clans run to Bunyoro. The clans that remained were of Lugave, and the then Prime Minister (Katikiro) stayed in control of the Kingdom on behalf of the King. There is a popular saying that the current Kingdom of Buganda re-emerged from Bunyoro Kingdom. Two children (Twins) were born in Bunyoro Kingdom by the wife of King Kabalega, one of the children, called Kimera, was hidden in a cave. Time came the Baganda people returned from Bunyoro to Buganda. The Baganda were informed that there was a royal child born in Bunyoro Kingdom that was left behind. Buganda sent a team that included two people, one from the Nkima Clan and the other from Mbogo Clan. When the two reached Bunyoro, the royal demanded, Kimera and his wife Namagembe to be lifted on the shoulders (*Kukongojebwa*) of/by the person of Mbogo Clan to return him from Mazigita to Buganda as a royal of Buganda. As they were moving, they lost a bell (*ekide kyagwa*). One Kayira informed one of his subjects to look for the bell in the nearby bush (*Samba samba ensiko eyo*), - hence the clan name Kasamba}. They failed to get the bell. So they ended up burning the bush in order to get the bell (nebookya a Kayiira – hence the name Kayiira – was given to Mr. Gajuule who burned the bush)

Nkongojja empewo za Bakabaka, eyo mulangira Kalemera, mutabani wa Kabaka Kalemera okuva mu Saza lye Busiro. - I am a medium for royal Spirits of Prince Kalamera, a son of King Kalemera whose spirit is from Busiro County.

Becoming a traditional healthcare spiritualist (both a Mulutansozi and Mulubaale)

Ebizibu byankaka okufuuka omulubaale, seyagalira - I was forced into healing spirituality. I went through a lot of sufferings before I become a traditional healthcare spiritualist. I was referred to as stupid and mentally disturbed. - (Researcher reflection: Why should the process of becoming a healthcare spiritualist be associated with sufferings?).

During my primary and secondary school years, I was taken to Muslim Founded schools and I studies reading the Qur'an very well. I had a job as professional mechanical engineer at Makerere University for 5 years. I worked in the department with my elder brother call Felesiko

Lwanyaga and with Professors Luwumba and Sengooba. When I left Makarere University after 5 years, I become self-employed at Bwaise and I could read the Qur'an against "evil" spirits.

When ancestral spirits selected and possessed me to serve, I resisted very much. I could read Arabic prayers *Dduwa* against the spirits while in the house and actually the ancestral sprits would get out of the house. However, the ancestral spirits would wait for me outside the house and possess me fully.

Emisambwa emitonde tegirinya kumutwe, emisambwa emizaale gyegirinya ku mutwe negyogera. Natural spirits do not possess a person, but it is the ancestral spirits that possess a person and talk. (This contradicts the expression by the spirits that say they are natural spirits (*tuli misambwa mitonde*)) – (*Emisambwa emitonde mu Baluntansozi gibawa bubaka mubiroto so nga Abalubaale gibogerelako kumutwe.* (The natural spirits give messages to Balutansozi while for the Balubaale they possess them and talk verbally)

I was involuntarily forced, as a mad man, to move from one place to another and finally taken in middle of a bush/forest where I stayed for more than two years as a mentally disturbed person (within the eyes of the public). This is my present location, a place I finally bought officially from the owners and constructed the current buildings that include a permanent home, multiple shrines and a Palace (Lubiri) for Kabaka Ndawula.

His workplace has two sections a Butonde and Buzaale sections. The Butonde section of traditional healing is where natural spirits are dominant. to him natural spirits are those whose origin is natural and do not originate from family lineage. *Emisambwa emitonde gya mubutonzi so si kika, ero gikwata omuntu yena gwegiba gisiimye ne gilonda okuva mubuli kika, gwanga, oba obutonde bwe, musajja oba mukazi.* The kind of spiritual powers and abilities in Butonde are natural and can be possessed by anybody chosen or liked by the spirits themselves regardless of tribe, sex, or age. This is supernatural power/ability (*buno buyinza*). People with such *amanyi n'obuyinza (*abilities or power), just their words have the potential ability to heal (*ekigambo obugambo kiwonya*). Such healing power/ability does not need to use herbal medicine to address the ailments. In the Butonde section of healing, offerings such as fruits, milk, cereals are done.

Obutonde tebuliko kika Natural places with natural forces or Kingly Spirits are not owned by any single clan or tribe. Such forces just possess someone who then serves their interests. (*Buno Buyinza.)* In such natural places with *amanyi n'obuyinza (*natural forces or abilities), there is no need for cooked food for humans. However such forces may possess a person who is already or will serve the humanly ancestral spirits (*Mulubaale).* In these natural places with natural abilities *(Mubutonde*) and powers, animal sacrifice, by slaughter or pouring blood is not acceptable. Offers are only natural products like Fruits, Milk and Ghee.

Training

Entendeka ya Lubaale yabikolwa okusinga ebigambo - Trainings by the spirits are more practical than theoretical. (It was relevant for me as a researcher to be present during some practical sessions and some questions I asked related to what I actually had seen happening).

Spiritual work should be done by the right people with the right spiritual powers and at the right place and time. The challenge is to identify the right people with the right spiritual powers, for the right work within the right time and place, under the specified and prescribed conditions.

Final examination and pass-out

Nze akasera kemisambwa ne Lubaale nakakolera Buddo wakati wa ba Senkulu okwali Kimbowa e Buddo, Omukongozi wa Kawumpuli e Buyego, Kyasanku e Bombo, Makolo e Nangabo. I did my final examination in ancestral spirituality at Buddo in presence of prominent spiritualists like Kimbowa of Buddo, Main spirit medium for Kawumpuli at Buyego, Kyasanku of Bombo, and Makolo of Nangabo.

Shrines

introduction

Muwanga's shrine (Essabo lya Muwanga / Ekiggwa kya Muwanga

Muwanga's Shrine is an ancestral shrine.

Mu sabo lya Muwanga, empewo zikozesa butende nga ebimira, ebisolo, ebinyonyi n'obutonde obulala n'amanyi g'abwo mukujanjaba - At Muwanga's Shrine, the spirits give clients natural medicines and spiritual powers embedded in plants, animals, birds and mineral products to manage or solve health challenges.

Lubaale mu bazaale akozesa obutonde omuli bwebimera, ebisolo nga eddagala mukujanjaba. ancestral spirits use natural materials in form of herbal medicine, such as plants and animals to manage health conditions.

Sacrifices and offerings

Some people are sent here to me by spirits or through dreams for thanks giving.

Animals and birds' sacrifices

During communal rituals, animals and birds are sacrificed and fruits and local foods are offered, mostly guided by ancestral spirits. The ancestral spirits are welcomed with anticipation by the participants, "*Tusanyuse okulaba jjajja*"

Saddaka ziwebwayo nokulamiriza ku lwo bulamu obulungi n'obukuumi – Sacrifices and supplication are offered for good health and protection

Mulubaale, ebisolo ebisaddakibwa kulwobulwadde oba eddagala enyama yabyo eliibwa abantu - In ancestral spirituality, the meat of the animals and birds sacrificed for treatment or medication is edible by humans.

The traditional healthcare spiritualist said that it is very important to keep animals, especially the sheep at home or at the shrine. This is important because in case the spirits are angered by the human being, they may unleash their anger in the animal and spare the human being.

Offerings

Offerings were milk offered in (*Byanzi*) is tested upon by the person offering to ensure that the milk offered is not poison. *(Does this mean that even the spirits [spirits] offered are in a state that can be poisoned? And if the spirits are poisoned what would happen?*

fruits offerings

Offerings: Fruits, Cereals, Milk, cow-Ghee, Fruits Sugarcane (Gowa type), Pineapples, Pumpkin, Water Mellon, Yellow bananas, Cereals; Millet (Obulo), Simsim (Entungo), Sorghum (Omuwemba) were offered and placed in a particular space around one of the four pillars.

All the offered cereals were placed in one big basket (*ekibbo*) and mixed thoroughly well.

The significance of materials used in spiritual work was elaborated. Seeds: (*ensigo zino zigira mu birimba*) The seeds are in bulk/plenty together on a stalk. So whatever you request for should be as much as the content of the seeds on a stalk. Simsim seeds (*entungo*) - (*kutungirira*) to put neatly together. Millet seeds (*ensigo z'obulo*) - *Kumerusa*, to enable spouting of new seedlings. Sorghum (*Omuwemba*) – *kuwembejja.*

The researcher was instructed to submit the offerings while the other people touched him. While submitting the offerings the following prayer was made:

"Maama Nalongo Nakayima, ne kabaka Ndawula omusambwa omusangwa omutonde, wamu n'omuzaana wo nakahema n'omulongo wamwe byamukama, nga tufukamidde wanno mu mbugga yamwe eyekitibwa eyobutonde nga nzize nentabaluganda eno (Emwanyi) okutabaganira wamu namwe, era nga nzize ne tabba ono tabamiruka okutabagana awamu namwe. Tabba onno gwendese, nsaba muntawulirize munsonda yonna".

animals and birds' offerings

eddagala ly'obutonde bwe busalirwa lifuna amanyi n'obuyinza byanjawulo okusingawo. When a sacrifice is made for herbal materials, the resultant herbal medicine has transformed energy and abilities to manage health conditions.

Most animals and birds meat used for sacrifice is edible unless in specified health conditions.

Sources and access to healthcare information

When asked about how ancestral spirits work, the response was. Lubaale yeyogerera lwaki tomwebuliza? nze *nga omukongozi silina nyo bumanyi Lubaale nenkola ye* – Ancestral spirits can talk for themselves about themselves, why not ask and engage them directly? for me as a spirit medium, I do not claim to have more knowledge about the ancestral spirits and their ways of working.

Communication with the spirits is associated with mutually agreed upon password or specified symbols understood by both the spirit and the spiritualists.

General songs relate to and associate with; Balongo (Twins), Mizimu, Lubaale, Misambwa, and Mayembe. Example of such songs: *“Mpunga mpunga, wolabira akwagala wolabira mpunga muyaga. Emisambwa gyenonno, wolabira akwagala wolabira mpuga muyaga …” "Zirindaba olwanga, tawa musibe.*

Sources of healthcare information

When one person gets possessed by a spirit, (becomes a Medium) all the people get close to the possessed person to share the blessings of the spirits.

When the spirits are in immediate accessible vicinity, the responded makes belches that sound like a roaring lion, which are regulated by use of a smoking pipe and tobacco.

I get dreams, but when I misunderstand or misinterpret the dreams, I suffer instead.

Access to healthcare information

emirundi egisinga nungamizibwa maloboozi na bifananyi byempulira no kulaba abalala abaninanye byebatawulira yadde okulaba - Many times, I get guidance through hearing voices and seeing things which other people around me do not experience.

bwempumuza omutwe wansi nenzibiriza, mba nga aloota nendaba abantu abambulira enkyokukolera omulwadde gwenina oba anajja. - "When I put my head down and close my eyes, I kind of dream and access information through kind of dreaming seeing people talking to me and giving me instructions on what to do either to the patient that I have or to the client yet to come".

Some information flow is associated with natural flow of things; One can be directed to do some activity in a place he/she has never heard of, but in the natural follow, directions will be unveiled, means of traveling and ease of work. The information may flow in symbolic manner

The respondent said that his communications with the spirits, through visions, dreams and voices is potentiated/enhanced by smoking the specific pipe of Kanoonya with dry leaves from Kisumuluzo plant.

Words and phrases that describe health, illness and disease

Words and phrases that describe health

Obulamu obulungi is good health

Obulamu obulungi is a situation where someone is capable of proving oneself healthcare even if he/she is not rich and can be able to attend to basic family issues and concerns.(social aspect of health)

Obulamu obulungi kitwaliramu omubiri, empewo, obwongo, empisa n’omwoyo. – Good health includes aspects of the body, spirit, mind, moral, soul

Obulamu obulungi obwemirembe relates to the peaceful physical body, spiritual, mental, moral, and soul components of human existence.(physical, biological, social, spiritual and moral health)

Obulamu obulungi relates to one's individual feelings as self-evaluated

Bweeza kyekigambo ekiwumbawumba embera yobulamu ennungi – *Bweeza* is a summary word for good health.

Mulaala means everything is okey

Embeera enungi is where one is able to get what s/he needs timely.

Obulamu obutali bulungi relates to health which is not good

Words and phrases that describe illness

Olumbe is a condition that will take you to a traditional healthcare spiritualist's shrine for consultations.

olumbe is a general term and can relate to all aspects of a person; the physical, mental, spiritual, cultural, moral, and soul. – *olumbe kigambo kigazi era kikwata kubuli kimu kumubiri gw'omunti*

Other words that can be used by a person with *olumbe* include; *Embeera yenyamiza, Muyi, Agayibwa, Akomuntamu kaganye, Lumubala embiriizi,Atweganyi*

Olumbe bwebukosefu nga tebunatumibwa linya, nga tebunategelekeka. – *olumbe* is illness before it is identified and given a name.

Olumbe yembeera evaamu obulwadde – Illness condition results into disease

Olumbe lutta naye obulwadde tebutta. Olumbe is the one that kills but *obulwadde* does not kills.

olumbe teluwona kuba nokulumanya telumanyidwa - *Olumbe* cannot be cured since it is not known.

Munono ya Baganda, olumbe litta, lwabizibwa ne lufulumizibwa okuva munyumba. In Baganda culture, *olumbe* that kills, is not known, the last funeral rites are done and the illness removed from the house and taken outside.

Kubonabona kwekuba n'ebizibu ebyenjawulo olusi ebitalabika n'amaaso, - nga bisobola okumwambululwaako. – Kubonabona is when a person experiences various problems some of which are invisible, but can be taken away through cleansing rituals

Olumbe bwelukwata omuntu ne lumugalanjula, kitege nti ekitundu ekisinga kubulwadde bwe bwa Kinene / (nga alina mumubiri obuyinza bwa Kinene). The sick person, where the cause is related to Kinene, can be managed by preparing to transfer the sickness from the patient into

the Kinene (Python snake). Olumbe lwa Kinene bukwaata omuntu yenna gwebuba bwagadde okukwata, naye bwebakola ensonga za Kinene nezitereela, olumbe luwona. However, the healer must be knowledgeble and endowered with authority (*omusawo atekwa kuba mumanyi and nga aliko obuyinza)*

Ekisirani;

Ebbanja mu mpewo

Ebbanja; (empewo oba obuwangwa nga bibanja)

Omulalu alya kukisasiro nga talina kimuluma taba mulwadde?

Singa omulalu gwebaleese basibye emigwa ajjibwako ekizibu ekimubaddeko nafuluma mussabo nga atteledde aba abadde mulwadde?

Words and phrases that describe disease

Obulwadde yembeera nga mbi (sinungi), mumubiri, mubirowoozo, - Bulwadde is a non-conducive condition in terms of the physical body and the mind. (Biological aspect)

obwavu bulwadde – poverty is a disease. *(*economic aspect)

There seems to be lack of a proper description of traditional word to describe disease in a traditional sense. Disease is one of the terms.

obulwadde simbeera mbi; Tebwawula mwavu na muggagga – Disease is not about poor social health, it included the rich and the poor

Normally *olumbe n'obulwadde* are associated and move together. For example, I normally receive clients whose problem in inability to retain money, when the ancestral spirit Muwanga examines them and informed that their problems were related to the need to harmonise their ancestral spirits (Lubaale).

Disease - oba oyogera ku kawuka (it relates to macro and micro-organisms) so the doctor is interested in finding out the particular organisms responsible for the disease.

Obulwadde *bwebo obujjanjabwa*. Obulwadde are ailments or concerns that can be treated.

bulwadde obwolukonvuba: refers to ailments of chronic nature

Healthcare using ancestral spirits *Okujanjaba kwo Mulubaale -*

Ffe mu Balubaale, Lubaale yajanjaba so si omukongozi. For us Balubaale, the ancestral spirits are responsible for the healing but not the human medium.

obusobozi bwomulubaale businzira ku busobozi bw'empewo zakongojja, obusobozibwe obuzaalinanwa, entendekebwa, n'ebyo byeyakakolako - Individual medium (*Mulubaale)* will experience powers and abilities according to his/her ancestral powers, background abilities, trainings, exposures and practical experience.

Omulubaale okilwako amanyi n'obuyinza bwa Lubaale ebijjanjaba, olusi n'okusinga obusobozi bwomuntu. Powers of authority and ability descend on a *Mulubaale* (healer spiritualist) that are responsible for healing, which powers are beyond humanly abilities and authority.

Some spiritualists, when possessed, are completely cut off (100%) from the worldly connections. s/he cannot understand anything when fully controlled. s/he is only told what transpired at the end of the session. Other spiritualists, the percentage (%) cut off varies from less than 100% to more than 0%.

Omulubaale asinga kujanjabisa mikolo na ddagala. Mulubaale mostly utilises rituals and herbs for treatment and health management. The health benefits of these rituals are to the client dependant on the state of the health condition by the time of presentation, the time of the day and the changes experienced by the client during the health management process.

I improve the health conditions of the people by consulting and getting guided by the spirits.

Spiritual power can be expressed by the stretch of the hand especially so where one feels filled up and engulfed by the spiritual powers.

health management using Misambwa - *Enzijanjaaba ye Misambwo* -

Enzijanjaba ye Misambwa yanjawulo. emisambwa jijawo ekileta olumbe n'obulwadde okusinga akutunulira obubonero bw'olumbe n'bulwadde) Misambwa have their own treatment modalities, they strive to eliminate the root-cause of the illness and disease than addressing their symptoms. The treatment that addresses the symptoms is only to give temporary relief but not to give a lasting solution.

Emisambwa gikolera mu byaama obutanafuya buyinza bwaajo. Generally, the Misambwa spirits are secretive in their treatment modalities so as to maintain their healing powers and abilities over many generations.

Kujanjabwa kwe Misambwa kwabikolwa so si ddagala - The treatment and health management by Misambwa is by conduction of rituals but not use of herbal medicine materials. At times the problem that manifest in the client is rooted in his/her ancestry, so during treatment you address the root cause of the ancestral condition for the client to get well.

Health management using words

Prayers physically untie any knots. Individual prayers are made to spirits (Balongo and Misambwa) when asking something from the spirits. (The physical act to untie is symbolic of untying problems).

This is an example of a communal prayers: *"Maama Nalongo Nakayima Ne Ndawula emisambwa emisangwa emitonde nga tuzze ne byanzi byamata. Nga esonga zetubanyonyodde ngatetubalagira naye nga tubasaba busabi, kubanga ekyamwe kibba kigambo bugambo. N'ekibera nga wewawo ngawewawo, nga wewawo ngawewawo. Tunatenderezanga mu niimi zoona, n'emu mawanga goona, n'emundyo zoona, n'emubiika byonna. Nga tusaaba okutwerusiza ensonga zoona zetunyonyodde nga amaata ganno. Bwetuba tulina kyetusobeeza, tusaaba okutuwa okutusonyiwa, okutuwa okumanya n'okutegeera wamu n'okutusiiza obubaka obulambukuffu. Nga amaata ganno getulesse nga tetulesse butwa ngaganywebwa."*

Thanks giving was always led by healthcare spiritualist and responded to by the clients (*Tweyanziza, Tweyanzege) x5, olwakaana n'olwakataano, bweza, bweza bwa mukasa)*

Prayer while at the forest *"(Otenderezebwenga, oguluzibwenga kulwa tonda, kulwa kubo kulwa munyenye, kulwa walaba, kulwa semwezi, kulwa namwezi kulwa lukuba, kulwa njuba ne kulwa mwezi, Waberenga wewawo x3.) x 9. Tunatenderezanga munimi zonna ne mumawanga gonna ne mundya zonna, ne mu biika byonna, ku lwo buyinza obuli wakati we gulu n'ensi wamu n'obutangavu obuli w'akati we njuba wamu n'omwezi n'emunyenye, ekisa kibere gyetuli n'okusasira okunji enyo. Tweyenziza tweyanzege x7 ngatusaba ebyetikibwa n'ebiretebwa mu mbuga zamwe omuli amata gatukuze ensonga, omuli obulo tumeruse ensonga, omuli entungo etutungirire birungi byerere omuli omuwemba gutuwembejje, omuli enanansi etwanjulirize birungi byerere, okuva mu bwakiragala okudda mu bwakyenvu mubuwomerevu, ensonga zaffe zonna zetuba tuyisiza mu nimmi zaffe, tusaba n'okusasira zibere nga mpomerevu nekyo kyetuba tusobeza, tusaba kisonyiwo era mutulungamye. Ebirala ebiretebwa nga mwemuli omuzigo mutugondeze ensonga zonna. Gusanuke nga bwegusanukira mu ntamu tusobole okufuna obulamu n'obuwangazi n'obugaga. Nga tuzze n'amakula wamu ne'bitone tusaba mwebyo nga ebyawebwayo mu lubereberye nga sadaka naffe tusaba omukisa okuva gyemuli nga tuzze n'ensujju nga lwelulyo olwa serulanda olwa ndawula olwasobola okusitula lukanga okugenda okulwana olutalo n'aluwangula mu Abyssinia. Bwekutyo nno naffe bwetusaba enimi zonna ezibadde eziba zimeruse ku nsujju enno zibere nga gemakubbo gaffe agasobola okutambula okuva ebuvanjuba n'ebugwa njuba, ebukikka dyo n'ebukiika kono. Tweyanziza tweyazenge x7 olwakana n'olwakatano. Kubadde kusasira kwamwe era n'akusiima kwamwe okubera nga tuli wanno mukitebbe kyamwe ekyekitibwa. Nga bwemukiriza era kwabadde kusiima kwamwe nemukulembera omukongozi wa Nakahema naye nasobola okubera wanno mu budde bwonna era n'abazukulu abali wanno bonna bemwalese okusoma, bemwalese okunonyereza bemwalese okusitula obuyinza bwamwe okubuza obugya mukiffo kino tetuyinza butakyogerako era nga betisse amakula ganno agaletedwa nga gakulembeddwa muzukulu munaffe Yahaya Sekagya bwetutyo nno bakama baffe tusima nyo kuba ekyamwe kiba kigambo bugambo era tusaba buli kyetugenda okusaba wanno buli kinnomu musime bakama baffe ffe tetulina buyinza bubalagira .*

A Thank you prayer was made *"Mukama atakaliza Nyanja ensawo ye tekalira. Akuwe obulamu, obuwangazi, obuwanguzi n'obugagga obwensibo, ibutevunya nga obuvunyu", "Oli kigonge kyakukubo, oli mweramanyo teguwalampibwa".*

health management using herbal materials

The words used by an empowered traditional healthcare spiritualist have the ability to heal with the additional use of herbal material.

Health management using other sources of healing powers

I have a *mutende* who is accessed by the powers of the sun and the moon (*akirwaako amanyi g'enjuba n'omwezi - Ssemwezi).* When that power is coming, its light is visible to all people around but its voices are only audible to the *mutende*.

The Sun, moon and stars

The respondent said "*Nzikkirwako nenebulungulurwa amanyi g'enjuba n'emunyeenye*" (the powers of the Sun and Stars connect with me and I am able to utilize the abilities of the Sun and Stars in the healing process.

Health management (prevention, protection, health promotion)

Kuwanga

There is an associated belief that the ground place where the old banana leaves have swept very clean and grass free, is very significant in terms of meaning and power, as used by some spirits especially spirit Muwanga for imbuing powers to specified items (*okuwanga*), like the Pipe *(okuwanga emindi). "Ekifo essanja weliyeze, ettaka ly'awo likozesebwa mu kuwanga okuwa amanyi n'obuyinza ekintu ekiwangibwa*". Example; this ability is used to imbue power into the pipe (*emindi*) by the spirit (Muwanga) to 'Kuwanga' for the pipe to gain power used in offering treatment.

The major spirits with the ability to empower (*empewo ezirina obuyinza okuwanga*) are two, Muwanga and Kawumpuli

Scheduled communal rituals for health management

Wano mu Lubiri, omusambwa gwa Kabaka Ndawula guvunuka buli 18 ezabuli mwezi, kujjoba lyo Mukongozzi negujanjaba okutandika saawa Mwenda ogwekiro paka kejenge – At this Lubiri, Musambwa of Kabaka Ndawula possesses it medium and expresses itself to its subjects on 18th of every month for health management and treatment starting at 3,00 am till dawn.

The spirit of Ndawula work closely with and in presence of its spiritual wife the female spirit of Nakayima, (the mediums behave intimately as husband and wife).

Treatment Process includes materials such as Cow-ghee, Cereals and fruits associated with chants, prayers and other ritualistic activities.

I witnessed one 18th day of the month. The whole day was spent preparing, as various people with multiple complains arrive at the place ready to spend the night in the big shrine (Lubiri).

Most people come with offering that include fruits, cereals, milk, coffee beans, Sackets (*amatu g'emwanyi)*, millet seeds, (obulo), simsim seeds (entungo), soghurm (omuwenba), Sugarcare (Ekikajjo kyagoowa), pineapples, pumpkin and cow-ghee. The treatment ritual process started as a ceremony on the 19th day at 3.00 am and end before day-break

The Presence of the various sets of twins was acknowledged before the process continues. At 3.00 am while singing the songs of different spirits (Balongo, Mizimu, Lubaale, Misambwa, and Mayembe), the spirit medium appeared form behind (*Entindi*) possessed by Kabaka Ndawula (*Kabaka Ndawula Yamuvunukirako*). The ceremony was then officially opened with recitation of specified words by the spirit medium, seated at *Namulondo* (specific chair). Besides him was seated a brown lady possessed by spirit *Nakayima*, who was introduced to the *lukiiko* (gathering participants). The gathering participants introduced themselves in detail (*Okulanya*) mentioning their Names, Names of the parents, their paternal grand-parents, and their great-grands, relating to their respective clans. All the offerings were then made to Spirit medium *Ndawula* through a soft spoken *Nakayima* spirit medium (*Maama Nakayima*).

The treatment session started with the most-sick patients who were brought before the spirit Ndawula. I assisted the sickest patient to remove his shirt, and spirit Kabaka Ndawula smeared the body of the patient with ghee, while pressing the body from top to bottom, and hitting the side walls, as if removing something from the body and transferring the sickness into the walls of the *Ntindi* in the shrine. The medium of Ndawula picked some of the mixed cereals, gave some to the patient to eat while some to keep. He also mixed some cereals with ghee and smeared the whole body with the mixture of ghee and cereals. This process was repeated for all the individuals present and each was given some ghee and mixed cereals to take and apply from their respective homes. Meanwhile, everyone would be praying for what he/she wanted and the spirit Ndawula would periodically say *MBATADDE* (I have released/relieved you) while the spirit Nakayima would preceded saying *NBEYIMIRIDDE* (standing in as surety). All the participants had an opportunity to interact with the spirits. The process ended by 5.00 am on 19th July 2019.

Ndawula is a Musambwa that uses words and smears ghee to heal – (any other ghee that he has not touched will not serve his intentions). The ghee he touches is imbued with healing powers and to remove/untie the heaviness/problem/sickness from the body of the client and place it though the walls or a standing tree by passing the palms of both hands to the body of the client and pass it on the walls or tree or any other sold substance. A client gets some of that ghee at any time and call upon the spirit of Kabaka Ndahuma as he/she smears on the body and call for assistance as he/she mentions in details all the issues that need to be addressed.

Spiritual cleansing (okwambulula)

Cleansing of spiritual places: Olubiri lwa Kabaka Ndawula; I witnessed the spiritual cleansing of the ritualistic workplace of the respondent, including the Lubiri lwa Kabaka Ndawula. On the set date 22nd July 2019, in presence of about 12 people, including the owner of the home, the respondent, two researchers two (2) Nakayima mediums the newer and older, Muzaana, (the wife of the respondent), Bamweyana medium and others both male and female. The ritual was led by Bamweyana medium. The spirits thanked the participants and assured us success in our endeavours. The ritualistic activity started by giving a spiritual bathe to all people present,

the "*okuzza abalongo obujja*" revitalizing and rejuvenating the powers /abilities of the twin forces, followed by cleansing Nakayima and Mayanja spiritual ligaria and houses, and ended with cleansing the whole place both inside and outside the shrines, the multiple fire places, the whole palace (*olubiri lwona*).

Some of the plants/materials used in the ritual bathe water included *olweza, omulamula, akakumirizi,* and milk. The place had two people mediums of Nakayima, the one who has been there for a long time. The new medium had been sent by the spirits as a new client and this was her first time to come to the place. When Ndawula spirit possessed the respondent, the client also got possessed, the communication that followed expressed that the client was the New Nakayima of the place because the old person as a medium has made mistakes as a medium of Nakayima. So spiritual arrangements had been done to replace her with a new one. (to my surprise, The new Nakayima was a stranger to both the old Nakayima medium and Ndawula mediums. Yet the Lubiri has to have one Nakayima medium. Therefore the spirit medium Bamweyana, while drinking strong alcohol, (Uganda Walagi) had to establish who of the two Nakayima mediums was to stay in the Lubiri.

The Lubiri cannot have two Nakayima mediums (*abakongozi ba Nakayima*), at the same time. Bamweyana medium requested the spirit of Nakayima to come and it immediately passed the new medium. The spirits while on the mediums greeted each other. The Nakayima spirit introduced itself as the original Nakayima from Bigo byamugeni (*Nyowe ndi Nakayima kuruga omubigo Byamugenyi*). Bamweyana spirit, introduced itself and its role to the medium of Nakayima spirit and asked the Nakayima spirit if it was in agreement with the Bamweyana spirit and its role in this process. The Nakayima spirit was in affirmative and added that that was the specific purpose of her coming. The whole place was abused and adulterated by the meduims of Ndawula and that of Nakayima having sex as humans within the places housing the spirits especially the twin spirits (abalongo tebakunamirwa). According to the spirits, sex was unacceptable and adulterate. The Bamweyana spirit asked for reassurance that if the place is cleansed, would it not be adultereted again? The spirit Nakayima while on the new medium, gave instructions that no person should reside in their shrine house and the old medium should vacate immediately, which she did. Spirit Nakayima expressed visible anger and grieve. Its central pole was adulterated by the sexual acts in the shrine and the spirit cannot use it appropriately (*empagi yange ekasisikara*).

Spirit Bamweyana asked spirit Nakayima what should be done on the Central Pole, to replace or cleanse it? Nakayima spirit replied that cleansing would suffice (be enough). The cleansing included use of nine (9) green fallen leaves of Kiwanuka's tree (*Omukokowe*). This *Mukokowe* is also referred to as *Omutuza Misambwa* because if used in the kyoogo, the cleansing water, it enable the spirits to settle as they used to (*emisambwa gitule nga bwegyatulanga*). All this was done in a free atmosphere and anybody could freely ask any questions and all the questions were addressed by the spirits. Spirit Bamweyana asked many questions regarding how the cleansing was to be done. All the questions were answered. The cleansing included the regalia, the shrine and the thickets surrounded by clothes (*akabira ka Balongo*).

Importance of spiritual cleansing of the spiritual place were to empower the place such that any prayers made by any person come to materialise.

Kaseegu: is associated with Immorality especially in talking

Causes of problems, illness and disease

spiritual causes

Spirits can cause, or be responsible for the cause of illness and disease - empewo zileta *olumbe and obulwadde.*

Bamweyana Spirit can be responsible for madness in people

Kinene - *(Walumbe e Tanda)* spirit is believed to be responsible to cause *olumbe*

The spirit "Ttembo" is associated with the mad person who is interested in picking, compiling and eating from rubbish (Kasasiro), while the spirit "Byuma" is associated with the mad person interested in metallic substances which he/she picks, ties them round the body, moves with them but may also keep a pile of old metallic substances.

In Kampala, the spirit "Byuma" is found in Katwe. It is also claimed that Katwe is a town associated with fabrication of metallic substances due to the positive association of the spirit "Byuma". This is also connected to the movement of the mad people towards the cities, towns and urban centres.

Spirits can cause witchcraft (*eddogo*) or enable/facilitate witchdoctor's effort. There are many reasons why spirits may be responsible for the experience of *olumbe, obulwadde, ebizibu, and ebisirani.* Spirits can make a person experience *olumbe, obulwadde, ebizibu, and ebisirani* when spirits have recognised you to serve them. Spirits can make a person experience *olumbe, obulwadde, ebizibu, and ebisirani* when spirits want a person to recognise them. Spirits can make a person experience *olumbe, obulwadde, ebizibu, and ebisirani* when spirits are taking a disciplinary action on the person. Spirits can make a person experience *olumbe, obulwadde, ebizibu, and ebisirani* when spirits are giving directions for a person to follow but the person is resisting.

causes as punishment

Kubonerezebwa - Kibonerezo; (kisobola okukwata kubulamu oba kumbeera) – A spiritual process of punishment .

okuziyiza obutabonerezebwa. – as a spiritual lesson to prevent being punished

when one misuses or is ignorant of his/her spiritual protection and guidance

Amanyi agakukamu nga tosanide oba tewetesetese kimala gakuletera obulwadde.- when one is not fit or is not prepared enough for the spiritual protection he is receiving

Olusi tukola obweyamo bungi eli empewo obutulema okutukiriza nebutuvirako olumbe n'obulwadde – At times we as humans, make many commitments that, we fail to fulfil and, in

the process, we get illness and diseases (*olumbe* and *obulwadde).* For example, while singing for the spirits we may say "*Bwemuliba muzze tulibawunda, bawunda bawunde"*, meaning that in case the spirits finally expressed themselves, we commit ourselves to decorate them to our best. yet when the spirits come and express themselves, we completely forget the content of the song and the commitments we promised to do. In that way, the spirits cause *olumbe* to us until we fulfil the promises we made. In my case, the ancestral spirit, *Kaduwanema,* omusambwa gwekika, *was* decorated as promised and it prevails during our spiritual gatherings.

Management of illness due to spirits

In spiritually oriented ailments, the use of herbs may not help, without the use *of actions, rituals and words – olumbe lujibwawo na emikolo bikolwa, n'ebigambo okusinga okukozesa eddagala"*

At times the unfavourable health condition is removed with rituals but not medicine.- *embera eteyagaza esobola okujibwaawo nakukola mikolo so sikunywa ddagala*

One time I received clients with spiritual problems that involved bringing together their spirituality from specified places at particular times of the night, under definite conditions and directions. *(lumu nafuna abalwadde nga balina kuyambibwako okukunganya emisambwa gy'abwe okuva mubifo ebyenjawulo, kundagirilo yobudde bwekiro nembeera enambike obulungi*)

Okujanjaba olumbe oluletebwa Kinene - Walumbe e Ttanda, kyetagisa ente eyamabala abiri. - The treatment of *olumbe* caused by spirit Kinene requires rituals that are led by a spirit-medium possessing appropriate powers and involve *sacrifice of a live two-colours cow [amabala abiri] (Black and White).* The Parts of the cow used are: *Akagula*, liver, Heart, and Kidney), which are burnt to ashes in the peaceful fire place *(Ekyoto kyemirembe).* Other requirements include; Two meters of white cloth, *ensaka (black in colour) for Amalwa (*local brew*), Endeku for Omwenge gwo Tonto (local brew)*, spears for the 3 spirits*; Bulamu, Kinene, and Kayikuzi, a black bull, two goats one black Male and Female eyaluyina* different colours on the head and behind with white patch in the middle).

Payments for healthcare services (Fees/Costs)

Some spirits determine the amount of money for the clients to pay and if the spiritualists decide otherwise, the spiritualists are punished.

Spirits (myooyo)

Ancestral spirituality can be adulterated, wronged, spoiled or lost.

Spirits were created to offer services on earth depending on the presenting situations.

Spirits do not want to be given empty promises.

Spirits give strict instructions to be adhered to, however one needs to apologise in case of deviations within limits, otherwise one is punished by the spirits.

Traditional healthcare spiritualist posses some supernatural powers, however, if misused or contaminated by human innovations, such powers can disappear from an individual and be lost. (Obuyinza Okumuvaako n'okumwesulubabba).

Emisambwa Emitonde muntandikwa (Created as Misambwa from the Beginning). These are created soul entities that lie in places like sacred mountains, rivers, forests, and isolated places.

Both Spirits "Ttembo" and Spirit "Byuma" are Misambwa by creation.

(*Amanyi/Obuyinza bwenkozesa mu buzaale bwesigama nyo kululyo olulangira*) The ancestral spirituality that I use related more with the Kingdom Royalty.

Obuzaale buli wansi wa Butonzi.

The word "Jajja" means that the spirit (Mizimu) come to us *"Emizimu jjo gyejjajja jetuli. Emizimu mizzi")*. This is also reflected in the song *"Emizimu mizadde gyajja gibwatula endagala aha jjajja jjinoonya jinaagyo".* Meaning - These spirits (Mizimu) came looking for the live human being that is where the word Jjajja originated.

Each spirit is endowed with unique powers and abilities which other spirits may not have "Buli mpewo Elina obusobozi n'obukugu bwaayo obwajitonderwa"

Particular spirits are assigned specified duties and related activities. – it is like children of the same parents but with different nature, talents, abilities, responsibilities, likes, dislikes and preferences.

When a spirit is given responsibilities, it may need assistants because the responsibilities may be too huge or require various expertise

The main natural spirits are two Muwanga and Mukasa "emisambwa emitonde emikulu giri ebiri, Muwanga ne Mukasa"

The main natural Jembe spirit is Lubowa.

Lubaale

Lubaale tasobola kujja nga taliko Muzimu. When Lubaale comes on the head of a person, it is asked "We are told that this luggage is yours, we would like you to tell us its content" Lubaale will then respond yes or no. if yes, then it will give all the details of its content. However, if Lubaale says No. it will add "This luggage is not mine, it is for so and so", until the actual owner of Lubaale comes, who would be willing give all the details of the content of the Luggage, regarding; abalongo, amayembe, emisambwa, in a process called *Kwaza Lubaale.*

"*Atalina ndege tagenda bulubaale*" This means that anyone who did not tie (*endege*) on the legs has shunned Lubaale. (*Atesibanga ndege yabanga yesambye Lubaale)*

Emisambwa emikulu ejjijanjaba mu Lubaale giri esatu Muwanga, Mukasa and Kadduwanema. – The three major ancestral spirits for health management are three, namely Muwanga, Mukasa and Kadduwanemal

Kadduwanema

Kadduwanema is one of the three major ancestral spirits in health management.

Mukasa

Mukasa produced two children, Musoke and Kiwanuka. The three, Mukasa, Musoke and Kiwanuka formed the original basis of Lubaale *abantu gwebasamira***.**

Lubaale Mukasa is responsible for "*kusiba amaliba n'enkanammu*"

Lubaale Mukasa is associated with *ekyoto, ekyoto kya Mukasa*. Selecting the location of *ekyoto kya Mukasa* is done through *bubaka n'obubonero*, where a spiritual message comes followed by physical signs. For example, in my case I received a spiritual message that someone will come and lie down at a particular spot, where that person will say word like "I need a fire place here for Mukasa". Actually, a stranger came and fell physically at a particular place within the spiritualist premises and demanded for ekyoto kya Mukasa that combines the spiritual energies of the land and waters (*Olukalu n'enyanja*), is the where the spiritualist put the Kyoto.

Ebiwebwa Lubaale Mukasa si bya kuyiwa musaayi. The offerings given to Lubaale Mukasa does not involve pouring blood.

When I prepare to attract the presence of Lubaale Mukasa, I dress in white, prepare the immediate environment with water, especially water from the lake, and remove any form of alcohol.

Kiwanuka

Kiwanuka is a name given to a child who was conceived but the mother continued getting her menstrual periods (*Omwana nga nyina yamulwalirako nga ali lubuto lwe)*

Kiwanuka mutudonzi, ye Kiwanuka *mulungamya nyondo*; *Obukkiro bwa Kiwanuka mulungamya nyondo buli; Kakiri, e* Banda - Kireka, ne Luweero. *Enyodo zino zikwatibwako mberera yo muwala oba omulenzi boka.*

Kiwanuka is a very serious spirit. It does not make jokes nor engage in smiles and laughter.

When I want to use the powers and authority of spirit Kiwanuka, I sit on the skin of animal which was sacrificed for him and I smoke his pipe, and repeatedly say my intentioned words - *Bwenjagala okukozesa obuyinza n'amanyi ga Kiwanuka ntuula ku Kiwu kye, nfuweeta emindi ye, nenamiriza*

Musoke

Lubaale Musoke is associated with *Busoke Busokelwa* the beginning, and is related with menstruation by women as a sign of maturity to bear children.

Lubaale Musoke's symbols are rings and bungles of golden in colour,

Muwanga

Muwanga Kiwotonono was originally from a Thicket in Mpambire.

Muwanga is not part of Lubaale.

Muwanga is not a Lubaale.

Muwanga is a spirit with abilities to imbue specified powers to ancestral spirits, Kings (Kabaka), royals, plants, animals and anything in nature. *"Muwanga awanga Lubaale, Bakabaka, n'obutonde.*

Muwanga was graced with natural ability/powers to imbue spiritual powers and abilities in objects (*okuwanga*) according specified duties and abilities that can be stimulated, tapped upon or directed with stated words, detailed out procedure or listed items.

Muwanga is a natural spirit (Musambwa mutonde). However, it also transformed itself and serves as an ancestral spirit Ndawula.

Muwanga descended on earth with various responsibilities in all clans of Baganda.

Muzimu

It is not usual for someone to be possess by the spirit (Muzimu) of his parents, (*omuntu tatela kusamira lubaale wa taata we*). *Tosobola kusamira lubaale wa taata wo nay otandikira ku lubaale wa Jjajjaawo".*

Song: Emizimu mizadde gyajja gibwatula endagala aha jjaja jjinoonya ginaagyo" this song means and illustrates that the ancestral spirits Spirits (emizimu) are associated with banana plantations and are responsible for the physical breaking of the banana leaves (*endagala okukonoka*). In an event that one sees a banana leaf physically breaking the, (*kulaba olulagala nga lukonoka*), that spirit (Muzimu) is an ancestral spirit in one's ancestral linage. The associated myth is that when one sees the banana leaf physically breaking, (*Okukonoka*), and try to physically touch that same banana leaf, you may be struck unconscious. In the same vein, in Buganda the leaves that have broken off (*endagala ezikonose*) are not used for preparing food. (*endagala ezikonose tezikozesebwa mu kusaanika mere, zibeera za mizimu era bwezikaddiwa zifuuka sanja*".

The other associated myth is that, in Buganda "Children are never allowed to go to Banana Plantations during time when the sun is overhead, midday, noon, (*Kalasamayanzi*) because it is believed that that is the time when the phantoms (Mizimu) are in the plantations.

The spirit (Mizimu) dress in backcloth (Lubugo).

There are some royal spirits that offer healthcare services

When a person dies, his spirit may express itself to the subsequent generations as a stransformed Musambwa or may remain in the Muzimu and continue to offer healthcare services.

Misambwa

In Buganda every clan has its own Misambwa, yet there are some Misambwa that are not based on clan structures, but natural forces that may possess a person on behalf of the natural world

Obutonde tebusamizibwa, omuntu bumukwata bukwaasi nebumulaga ekyokukola

Obuyinza bwemisambwa bwemalirila, bwobugatamu ebilara nga te bukirizza, busobola okukwabulira

Emisambwa Emizaale nga jaali Mizimu are when a person dies and takes a very long time, his spirit (omuzimu) may transform into Omusambwa. This is the origin of 'Emisambwa emizaale with ancestral linage.

Misambwa dress in cloths.

Emisambwa gigezesa abakongozi b'ajo: The process I went through to get the "*Ddamula" (Amanyi/Obuyinza)* that I hold was a long process, full of trials and scaring moments, but with a clear setting and arrangement that we came to fulfil. Everyone had a unique role to play and those without were always left out unconsciously.

Misambwa are many and carry out various functions. The Misambwa I am engaged with offer healthcare services.

The royal spirits of Kawumpuli, Bamweyana and Ndawula are in every clan of Baganda and offer healthcare services.

Ndawula

Ndawula musambwa

Ndawula mulangira

Ndawula spirit is not found in all clans of Baganda.

Ndawula is one of the most dangerous spirits when demanding its harmonization rituals

Spirit Ndawula is associated a male, uncastrated white Lamb.

In Buganda Mulangira/Kabaka Ndawula has many names; Ndawula Segamwenge; Ndawula Kikyonkyo; Ndawula Byuma; Ndawula Gogombe; Ndawula Kyalubimba; Ndawula Kisaka; Ndawula Majegere;

Ndawula is associated with green and yellow colours; symbolised by green and yellow cloths.

Ndawula make his communications through dreams related to green and yellow colours

Ndawula is an ancestral royal spirit found in all clans of Baganda and is referred to by many names.

The dressing regalia for Ndawula has many restrictions. One is not allowed to go anywhere while putting on Ndawula regalia dress.

A spirit medium does not go to the pit-latrine or toilet while dressed in Ndawula's regalia

All twins are referred to as children of Ndawula. Some children of Ndawula are expressed as snakes

The kind of work Ndawula does is unique and is not for all spirits. Ndawula only appears when there is cause, but its way of demanding is bad. (*Ndawula abanja bubi*). Ndawula may cause any form of disease on the body when it is expressing its demands.

Sign and symptoms of Ndawula relate to pricking pains in the body.

Regalia for Ndawula include a smoking pipe (photo), walking stick (Photo), endeku (photo),

Ndawula *tebamulamiriza*.

Ndawula *tebamukozesa nsobi*

Ndawula is not engaged in witchcraft and a healer cannot use the powers of Ndawula in witchcraft. Otherwise Ndawula is known for punishing any healer misusing its powers.

Ndawula musambwa muzibu nyo, naye ate Ndawula musambwa mugabi nyo bweguba nga gutelede. Ndawula is very difficult Musambwa but Ndawula is a very giving Musambwa when well harmonised.

Bamweyana

We were advised by Bamweyana spirit that was safer and more rewarding to act stupid/foolish than to act wise. In this world, it is advisable to have both wisdom and stupidity. However, stupidity should be more than wisdom because, when you act stupid, you will be successful in most of your endeavours, but when you think you are wise, you end up not being successful. The spirit related the advice to songs by singing; "*Abantu bekika bantama lwakugeyangana", "Lwembawo lwemba munamwe lwesibaawo…", "Buno bwebuyumba obutono kasangwawo X 2"*

"Bamweyana Omulalu" is a spirit Bamweyana associated with fire, and is believed to move with two other spirits called "Ttembo and Byuma". These three spirits, Bamweyana, Tembo and Byuma are the most responsible causes of madness seen in most people.

Spear for Bamweyana: Shape? Make? Significance?

Stories of the origin of Musambwa Bamweyana and how he came to be burned at Katerekke.

Kawumpuli

Spirit Kawumpuli e Buyego is associated with a song. *"Akabira kano kalimu ekyama" - aka Kawumpuli e Bajjo – e Buyego*

History and background of Kawumpuli

The potentials of Kawumpuli and his works

Example of Kawumpulii empowering

Omuzimu

Ekibbo kyabalongo

Ekibbo kya Lubaale

Ensawo

Nakayima

Nakayima is a female Musambwa, Originally Nakayima was not a Musambwa of Buganda, but now it is considered a very important Musambwa in Buganda.

Nakayima tajanjaba – Nakayima spirit does not offer healthcare services.

Spirit Nakayima is associated with a female white Cow-female.

Nakayima's symbol is in form of a snake. Nakayima is associated with twins in various forms such as snakes. Nakayima is prepared (*Okutekebwatekebwa*) and Beautified (*Okuwundibwa*).

Functions of Nakayima is to stands in for people (*Yeyimirira abantu*) / Nakayima spirit stands in for people (Surety) [Muyima].

The offering for Nakayima are Milk - placed in *Ebyanzi* (guards) and Cow-Ghee.

Nakayima communicates through dreams and visions, signs and predicted happenings (obubaka n'obubonero), at times clearly described in minute details.

The spirit Nakayima presented itself as a snake which was killed by one of the respondent's sons who become unconscious, and then apologised when the image of a killed snake requested to know what it had done to deserve to be killed. The respondent and his family recognised the dead snake as the predicted Nakayima spirit, apologised and made all arrangements to prepare the spirit as earlier on prescribed. (*Omukongozi akutegedde era ajja kukuteekateeka n'okuwunda nga bwewamutegeeza mububaka, nga bwewalanga*). The snake spirit had the identifiable markings as prophesied before and during its preparation. Two (2) live snakes appeared to come out of the cloths where the respondent was seated. These two snakes were latter recognised as the twin snakes for Nakayima.

Kaliisa

Kaliisa, Musambwa tegujanjaba – Kalisa is a Musambwa, but does not offer healthcare services.

Kaliisa mulunzi – Kaliisa looks after cattle, is a herdsman. So, if issues of health management rituals refer to the need of animals or animals parts, then the issues may be referred to Kaliisa spirit for those aspects of the required harmonization or management rituals

Health management of animals is referred to Kaliisa as the main spirit

Kaliisa is the main spirit in animal health management

Mayembe

Jembe Lubowa is the assistant and contact spirit for all natural spirits

Mayembe work through forming partnerships and alliances

Kanoonya

Kanoonya is a spirit responsible for all investigations required during my healthcare services delivery –"Jembe Kanoonya lye jembe ekulu mumirimu ejirimu okunonyereza"

Kanoonya spirit has a specific smoking pipe. (Why the difference in the structure of the pipes? are the contents also smoked different?). Kanoonya and Nakayima spirits were the spirits that looked for and destabilised the respondent and forced him out of his comfortable place at Makerere to a forest in Watuba where he is upto now.

Katabaazi

Katabaazi jembe. – Jembe Katabaazi does all works given to it

Kasajja

Kasajja jembe kozi lya mirimu – Kasajja is a Jembe that does all works given to it

Balongo

Abalongo have many types; *abazaale, abatonde, abasibe, abafugike, bakasowole, abensozi, abemponpogoma, abomubiwonvu, abomubiko, abayitira mubyewalura, abayitira munsolo enkambwe, abomumigga, abomuntobazzi, abomunyanja, magobwe, kitinda, mayanja, seruggulamilyango, ssettimba, nattimba, : abali muntindi n'abali kumyaliro*;

Abalongo tebakunamirwa - meaning that the house where the twin forces are kept should not be used for sex purposes.

Twin forces link the human forces to the forces of nature and the respective communications between the rest of nature and humans.

Walumbe (Kabaka Bulamu)

Walumbe spirit transformed itself into ***Kabaka Bulamu.*** That is why a Palace was built for him at Kasagati. This ***Walumbe/Bulamu (Omulongo wenkanamu)*** is the owner or one responsible for ***Biteega, Bitambo, Bisinya, Bitikatika, Ebidandi, Ebyokoola,*** who are considered children of Walumbe/Bulamu ***(Song;*** *"Kinene akuuna, muleke akuune, kinene akuuna bwagwa wansi yegalajula; akuuna munda yanyabe, bwagwa wansi yegalanjula") omusota gwegalanjula. kyamagero omwana okumuzaala neyegalanjula.* I have been talking in parables, the fact is that I am a Salongo. I delivered twins and one of the twins was a Python snake. Of the twins, the first one to come out was a human being *Waswa*, who was followed by a *Python snake.* The respondent showed us his second twin child (*Kato*), a python snake, which was well wrapped and decorated.

Ebiwebwa Kabaka Bulamu tebiyiwa musaayi

Royal spirits

A Kabaka (King) is a royal upon whom some rituals have been performed *"Osoka kubeera mulangira n'olyoka ofuka Kabaka oluvanyuma lwokukolebwako emikolo"*

Kabaka is originally and symbolically associated with "Akaba" (the lower Jaw). In Buganda Culture, the King does not die ("*Kabaka taffa" aserera oba azaama buzaami*). in Buganda culture, Kabaka (The King) is the origin of culture and its norms."*Ennono n'obuwangwa biva iri Kabaka era buli gwanga lirina obuwangwa ne nnono zalyo"*.

In Buganda Culture, when the King dies (*nga Kabaka aseredde*), his lower jaw (Akaba) is removed from the body and retained in his Palace, while his body is taken for burial in "*Masiro*", a place where the King's body is placed. (*Why is the lower jaw of the King removed when he dies? What is the significance of the jaw in relation to power and abilities to rule?*) = The King remains in control of his Kingdom and the powers (*amanyi/obuyinza*) and some of which are passed to another to inherit the kingdom as the King. The spirit of the King (*Omuzimu gwa Kabaka*) can possess a selected person (*Kabaka avunukira ku mukongozi gw'aba yesimidde*) and exercise its authority.

Akasera mwabo abasamira omusambwa gw'obakabaka: These are quite many difficult tests undertaken to prove that the claimed Kingly spirit is actually the one possessing the claimed person. In my case for the Kingly spirit of *Kabaka Ndawula.* "in my case, at around 10.00 pm, I was placed in the middle of experts in Busiro, and the king's decendants, at *Kayima's* home in *Nabulagala* for them to prove if actually the spirit of their long gone Ancestral King *Kabaka Ndawula* possess me, and if not, I stood to be speared dead. a steam of very strong light (*Kibonoomu),* brew from one side of where we all were, to the other side, then after a few minutes it strongly brew back and a strong source of light, (*nga empumumpu)* stood on my head with light rays radiating in the sky.

This surprised and scared my people and the following day one of the Princess (Omumbejja) kind of scorned me publically, and she immediately developed a very bad generalised scally skin rash. I only came to her rescue after some days, by use of words in (kwegayirira). It was a very big lesson for all. Then one of them asked, if indeed the spirit of King Ndawula, let rain come and pour. Soon after his request, very strong rains come and heavy water poured and all the tents were shacken and socked wet. In fact, since then, where I am settled with his spirit, in most cases it rains. Also, if heavy rains are coming, I have the capacity to use words to distribute it into two which parts take different sides. *(Obuyinza bukolebwa Kiganbo/Word).*

Traditional Healers Authenticity can be tested / evaluated one King pretended to be very sick and made tricks to evaluate the authenticity of traditional healers by placing "*entengotengo*" in the chicks of mouth such that saliva started oozing out of the mouth. His subjects got concerned and took him to a traditional healer's place who after using his diagnostic tool regalia/*omweso*) identified and told the King that, the King was not sick, but needed to remove the "*entengotengo"* from his mouth and that the King would be okay, Since then Traditional healing practice was officially allowed to continue to be practiced in his kingdom. So a song was developed "*Bantadde x 3 gwe mussota amafumu n'enyaggo*". Since then it meant that fighting traditional healers is not easy.

Shrines

Amasiro:

The places occupied by the kings (Basekabaka). It is claimed that this practice started with King Kayemba (Ssekabaka Kayemba). Examples of Amasiro: *(1) amasiro ga Ssuna e Wamale, (2) Amasiro ga Ssemakokiro e Kibumbi, (3) Amasiro ga Kalema e Mende, (4) Amasiro ga Kyabaggu e Kyebando, (5) Amasiro ga Kimbugwe e ...., (6) Amasiro ga Jjuko e Bujjuuko, (7) amasiro ga Mutesa I and II e Kasubi*

Lubiri

Lubiri belongs to "Emisambwa emitonzi". Examples of Lubiri in Buganda*; Olubiri lwa Ndawula e Watuba, Olubiri lwa Kawumpuli e Buyego, Olubiri lwa Nazigo e Nazigo, Olubiri lwa Bulamu e Bulamu.*

At the respondents settings, *olubiri lwa Ndawula* (the Shrine of Ndawura) is a very big , compared to other shrines, round shaped and located toward the end of a the designated spiritual place. It is originally roofed using thatch grass, but covered with grey iron sheets. Ndawula shrine has four wooden pillars squarely placed in the middle of the shrine stretching from the ground to the top of the roof. There are multiple traditional regalia, including many short small spears surrounding the four pillars. The floor is covered with grass (*Etteette*) on top of which are places mats made of both palm-leaves (*ensansa*) and recycled plastic materials. There is a fire place on the left side as you enter the shrine, while the spiritualist gets seated on a mat facing the entrance. While inside the, the female and male clients seat on the left / right side of the spiritualist respectively whom they face. The specific places and positions of most content in the Shrines were determined by the respective spirits. Example is the position of the fire place with the shrine.

Kiggwa

Ekiggwa is the shrine that belongs to a Clan in Buganda (Ekika)

Ssabo

Essabo belongs to a traditional healer's home

Natural places

Emisambwa gisobola okukusibira mukifo

Nyiize

Nnyiize kifo kyabutonde, etwalibwayo Matoke, Ssegwanga, a goat, cow meat.

Walusi

Walusi is a natural sacred mountainous place with many spiritual places of specificities

Walusi natural rocks place has lots of spiritual powers (*amaanyi*) but a lay person who has no connections with the spirits cannot experience the power

Sexuality in spirituality

Issues of sexuality are very sensitively dealt with within spirituality. It is safer to say sex is a taboo in spirituality, said by jjajaBamweyana@Wassajja.Musambwa.

Spirituality gives very tough tests and a successful person wins lots of spiritual powers, abilities and favours. For example, the Medium of Ndawula to smear cow-ghee to the whole of the naked body of the Medium of Nakayima at about 3.00 am every night for twenty-seven (27) consecutive nights (emyenda esatu). This demonstrates that although the spirits of Ndawula

and Nakayima are husband and wife, their Mediums are prohibited from any sexual interaction they are meant to act as brother and sister.

Regalia

Backcloth

Backcloth (Lubugo) is the original dress of humans in Buganda. Backcloth (Lubugo) has been currently demonized and associated with witchcraft.

Witchcraft

So *eddogo* (witchcraft) has both the negative and positive aspects.

During spiritual rituals, the initial steps call for the presence of, and attract the various relevant spirits through preparations, cleansing rituals, setting and lighting of fire places, use of incense, singing and chanting particular words while clapping and drumming or use of materials, artefacts or signs known and attractive to particular spirits. For example, if I would like to use the negatively or positively the strong forces of Kiwanuka, I will prepare by getting cleansing rituals myself, put on my long red dress (Kanzu), set fire in his fire place and light his spoke pipe and I will feel his (spirit Kiwanuka) presence. However, if I do not feel his full presence, then I will sing his much-loved songs to attract him more. Them I will say the intended words for good or for bad, and finally request my desires to be implemented.

Words can be used to create a negative or positive situation. *Kyenamiriza nga nkola emikolo jempewo, empewo kye ziwulira era kye ziteka munkola, kibi oba kirungi*. – the words that I repeatedly say during the spiritual rituals, are the words the spirits present hear and implement, whether the words are bad or good.
