# Supplementary material for: Perspectives on health, illness, disease and management approaches among Baganda traditional spiritual healers in Central Uganda
Source: PLOS Glob Public Health. 2024 Sep 6;4(9):e0002453. doi: 10.1371/journal.pgph.0002453 (PMC11379289; doi:10.1371/journal.pgph.0002453)
Supplement: S5 Data — (PDF) [file pgph.0002453.s005.pdf]

Study participant 5 transcriptions

Contents

Study participant 5 transcriptions 1
Social-demographics 2
Types of traditional healthcare spiritualists 2
Abaluntansozi 3
Abanozi be ddagala 3
Kusamira 3
A *Mulubaale* in a community 3
Becoming a *Mulubaale* 3
Sources and access to healthcare information 4
Sources of healthcare information 5
Access to healthcare information 5
Access through signs 5
Access through vision, dreams or thoughts 5
Access to information through flowing thoughts 5
Words and phrases that describe Health, Illness and Disease 6
Words that describe health 6
Words that describe illness 6
Words that describe disease 7
Health management 7
causes of illness and diseases 7
Causes of *lumbe* (illness) 8
Causes of *bulwadde* (disease) 8
Witchcraft (ddogo) 8
Kisirani 8
health assessment and diagnosis 9
Kulagula 10
Kwaaza 10
Omweso (diagnostic tools) 10
Prevention, protection and Treatment 10
Kuganga 10
Kwambulula 11
Yirizi 11

Prayers ... 11
health promotion ... 11
Spirits ... 11
Katonda ... 11
Ancestral spirits (Lubaale) ... 12
Misambwa ... 13
Muwanga ... 13
Kawumpuli ... 14
Ndawula ... 15
Mukasa ... 15
Kadduwanema ... 16
Kiwanuka ... 16
Musoke ... 17
Muzimu ... 17
Mayembe ... 18
Balongo ... 18
Ddungu ... 18
Mayembe ... 18
Kinene – Bulamu ... 19
Natural places ... 19
Symbols and symbolism ... 19
Regalia ... 19
Spears ... 20
Ekiwu ... 20

Social-demographics

My name is (name withdrawn), and married. I am a 64-years old male Muganda of Nvuma clan. My religion is traditional religion. I was once a Muslim, then a protestant but now a traditionalist. I stopped in Senior Two (S.2). I am a Mulubaale and a farmer. I belong to Uganda N'eddagala N'obuwangwa Bwafe traditional healers association in order to have unity with other traditional healers in the whole country. My shrine is a *Kiggwa* and this is (x) village, (xx) parish, Bulemezi County (Saza), Nakaseke district.

I was among the few people who participated in the rituals of crowing the King of Buganda Kabaka Muwenda Mutebi II at Naggalabi Buddo

Types of traditional healthcare spiritualists

The many types of traditional healers include abaluntasozi, abanozi be ddagala, abalubaale,

Abaluntansozi

Abaluntansozi tebatembebwa mpewo ku mutwe, naye empowo zibalungamya mubirooto. - Baluntansozi are spiritualists never possessed by natural spirits but guided through dreams.

Abanozi be ddagala

Abanozi be ddagala tebatela kulinyibwako mpewo naye zibakulembera n'okubalagirira nga banoga eddagala. Abanozi be ddagala are traditional healers who are not possessed by ancestral spirits but led and guided by spirits during their harvesting of medicinal plants.

Kusamira

What is Kusamira?

Kusamira kwe kutendereza – Kusamira is a process of giving praises – Kusamira is a process of praising

Okusamira is a word used to describe promotion of health

I started the *Kusamira* process in 1980

A *Mulubaale* in a community

I have been in this community for twenty (20) years. I am proud of this community and the community is proud of me. Most of the community members are my friendly clients because I address their health, cultural and spiritual issues. Most of the youth are my grand-children (*Bazukulu bange*). I helped some of their parents to conceive when they had failed to conceive, stabilised their early pregnancies during threatened abortions, provided traditional antenatal and postnatal care and conducted most of their child deliveries. They consult me for business related issues and unstable family relationships where I offer counselling, guidance and medicines.

Becoming a *Mulubaale*

"*Bwetwafuna ebizibu mu kika twagenda kundagu ne batulagula nti Lubaale abanja kuterezebwa" Twetegeka, netulima ebijja, netwabya enyimbe, netutekateka abalongo, netulyoka tugenda okwaza Lubaale.* When we got problems in our clan, we consulted traditional healers who divined that our ancestral spirits needed to be harmonised. We prepared ourselves for the *"kwaza Lubaale"* by properly cleaning the grave yards, doing the last funeral rights for the dead, and harmonised rituals for the twin sets.

Bwetwamala okwaza Lubaale wafe mukika, n'awebwa ebibye byeyasaba, n'asalirwa ensoloze, olwo Lubaale yajibwa mu diiro, natwalibwa mu kiggwa, nayanjulibwa eli abekika, yayawulizibwa nayingizibwa mu kiggwa natelekebwa olwo nentandika okutendekebwa kulwokuba lubaale wange yali mujanjabi. After exploration of ancestral spirits with the family members, it was given whatever it demanded, including sacrificing animals, Lubaale was separated, introduced to the family members and secluded securely kept in the family shrine (ekiggwa). Then I started my training and apprenticeship since my ancestral spirits had a healthcare role. The training process involved me as an individual trainee and the ancestral spirits guided by the trainer and his ancestral spirits. The process enabled me acquire much knowledge.

“Ndi mutendeke” I was trained. “Lubaale wafe twamwaaze nga sinatendekebwa”

Let us encourage our children to go to school. If the spirits possess the educated people we may get more quality spiritualists.

Let us show and expose our children and grandchildren our culture and the cultural values

Okutendeka omulubaale (batendeka Lubaale n’oMulubaale?)

Okutendeka is when Lubaale appears and says what it is demanding for and they are given to him.

Kunze Muzimu gwegwayasa ejjoba, negulagira okukola ekijjulo era anakulembera emikolo gye kijjulo yeyali ajja okuba Senkulu wa Lubaale n’okuntendeka. In my case, the Muzimu spirit appeared and possessed me first, gave instructions for a communal meal and the leader of the associated rituals would be the *Ssenkulu* (lead and chief trainer) for my training and apprenticeship. The apprenticeship involved many rituals that empowered Lubaale for its healthcare roles and activities.

In the case where the Lubaale did not have healthcare role or function towards the community/public, when the rituals are performed and given all that it demanded for, it is then safely stored in the clan shrine (kiggwa) for the family members to come, pay tribute, pray and go. This type of Lubaale is referred to as *Lubaale w’esanduke*. Family members prepare annual communal meal (ekijjulo) for this Lubaale. However, it is advisable for a knowledgeable person (Senkulu or jjajja w’abaana) to lead the process and advise on the rituals associated with the preparations of the various meals and how to serve out these meals

Lubaale ng’amaze okutendekebwa, Ssenkulu ne Jajja balubaale babuuza Lubaale okunyonyola entekatekaye eli abe kika. Empowo okwali Omuzimu, Lubaale Mukasa, Muwanga, Kadduwanema, Kawumpuli, Amayembe n’empowo endala, banyonyola entekateka namutayika n’engabanya yemilimu gyabwe ejokujjanjaba ne balonda ne Katikiro we Ssabo.

Towards the end, after the harmonization of Lubaale, the *Ssenkulu* and *jjaja wa Lubaale* asked the spirits to explain to the family members how they were going to carry out their healthcare services. The Spirits provide their working program majorly the working relationships involving the major healing spirits Omuzimu, Kabaka Mukasa, Muwanga, Kadduwanema, Kawumpuli, Amayembe and other spirits. Within themselves, they even divide up roles and responsibilities and who will be the *Katikiro* spirit for the shrine.

Those spirits which do not carry out public healthcare service among the community (*ez’omusanduko*) also agree on who and how to carry out their services to the family members especially those that come, pay tribute and pray to them.

Sources and access to healthcare information

introduction

nfuna obubaka mumitendera mingi; natandikira mubirooto, mpewo nezininya, npulira amaloboozi nga omuntu ambulira, mbonekelelwa; I get information through various ways; I started by getting dreams, then through possession by ancestral spirits, I hear voices as if someone next to me is giving me instructions and at times I get real visions.

I work with Muzimu, Lubaale, Mayembe, Misambwa and Balongo.

Sources of healthcare information

Obubaka buva mu mpewo za ba jjajange – The information comes from my ancestral spirits

Access to healthcare information

Bwenjagala okufuna obubaka nkuma ekyoto, nenfukamira nensaba oba nenegayirira – When I want to get information, I make a fire, kneel and humbly and sincerely pray

Olusi okufuna obubaka obwenjawulo, nsoka kufuna kyogo nga mulimu olumanyo, omulamula, Rweza, ne Bombo – Sometimes, in order to access some classified information, I take a ritual bathe using medicinal plants including *olumanyo, omulamula, Rweza, and Bombo* etc.

Ntera okunywa emindi okusobola okukowoola empewo jemba ngenda okukola nayo mubukugu bwayo - To access healthcare information, I frequently smoke a smoke pipe (*emindi*) of particular ancestral spirits to invite them for their specialised activities.

Access through signs

I can access information through signs. For instance, *kunze akabonero ko musota gwa Nawandagala kategeza Balongo, ate nga omusota gwa Namagoye gutegeza ku nsonga za mpewo ya Bulamu.* For me when I see a green snake Nawandagala, then the issues at hand relate to twin spirits, while the snake *Namagoye* relate to the spirit of *Bulamu*.

Access through vision, dreams or thoughts

Spiritual information can access the spiritualist through dreams, thoughts, or human being.

Ntera okufuna obubaka nga nsiziira mu birooto, okubonekerwa oba mubirowoozo ebyenjikirano. Nsobola okuba nga nfumitiriza ekyokukola, empowo zabajjajja nezindetera omuyikirano gwebirowoozo kwebyo byensanidde okuteeka munkola, ela nembikola nebikola. I often access information through dreams, visions, or flowing thoughts. I might be contemplating what to do, then the ancestral spirits feed me with a flow of thoughts on what I should do and when I do follow the flow of thoughts, things work out well.

Access to information through flowing thoughts

One time, I had an issue with a religious pastor who rented a house in my neighbourhood, turned it into a church and placed high volume loud speakers. All his preaching was about me, and my devil ancestral spirits. His preaching embarrassed me and my services to the community. As I was considering what to do, I got the thoughts flowing on the rituals I should do and when I did those rituals, the religious paster relocated within the three weeks.

Olulala, abantu nga bakulembedwa omukyala asitude omwana eyali abukiddwa eyaabwe nga alowozebwa okuba nga affudde. Olwatunula ku mwana ebilowozo nebinjikira okukola omukolo n'okunaga ebikolo byeddala. eddagala nalinoga mubwangu nendiyemga nenaaza omwana omubiri gwona, Bweyatandika okwenyenya nga alwaana, nenfuna ebilowozo okumunyeseko katonokatono. Omwana yafuna otulo okumala essawa nga satu. Yazukuka nga ali bulungi, maama we namuyonsa. nebadda eka. When I asked what was the health condition that he managed. He responded. *Nakati simanyi kyenajjamjaba*. Up to now I do not know what I managed.

Another time, I received a group of people led by a woman carrying a child who had got episodes of febrile convulsions and was feared dead. When I looked at the child, I got thoughts to do a ritual and to pick particular plants and make a concoction which I used to bath the child. When the child started moving the body and fighting, I got other thoughts to make the child drink some of the concoction slowly. The child got into deep sleep for about three hours. The child woke us in fair condition. Its mother breastfed it and they went home. When I asked what was the health condition that he managed. He responded, "up to now I do not know what I managed".

Nkola ejo bulubaale buli wemba ndi - I can perform any healthcare function roles wherever I am, within or outside my shrine

Words and phrases that describe Health, Illness and Disease

it is very important to first understand why people come to us Balubaale.

The most common reasons why people come to us Balubaale are consultations *(Kwebuuza,* To prepare (*Kwetegeka*) and to participate (*Kukiika*)

Words that describe health

Bulamu - Ndi bulungi, silina wanuma, Obulamu obulungi / obulamu obweyagaza - health is the life one has, and it is what describes a live person.

Omuntu ali mubulamu obulungi oba obulamu obubi A person has either good or bad health

obulamu obulungi kwekuba n'emirembe kin'omu nabomumakago, Good health is when there is peace as an individual and one's immediate family

Lubaale akozesa ebigabo byanjawulo okutegeza obulamu oba embera enungi - Ancestral spirits use particular words and phrases to express good healthy (situations)

1.	Spirit Kawumpuli	use	Ndala
2.	Spirit Muwanga	use	Kalembe
3.	Spirit Mukasa	use	Davu Sese Davu, Gayira gayira
4.	Balangira	use	Gusinze
5.	Misambwa	use	Gusinze
6.	Omuzimu	use	Bwera bwera bwerage

These words used by specific Spirits refer to presence of (mirembe) peace; Ndala by Kawumpuli, Kalembe by Muwanga, Davu Sese Davu, Gayira gayira by Mukasa, Gusinze by Balangira and Musambwa and Bwera bwera bwerage by Muzimu.

In daily life, one may say "Ndimulamu naye siteredde" I am healthy physically but I am not settled. It may also mean that I am healthy but I have a situation which is not good, financially, or I am not getting what I want.

Words that describe illness

ddogo lumbe – witchcraft is illness

Olumbe bulwadde obutawona – an incurable disease is an illness

obulwadde obutawona lufuuka lumbe, okugeza ekiwundu ekifuka kokolo - a persistent disease becomes an illness, for example, a wound that becomes cancerous.

olumbe lutandika nga bulwadde – an illness starts as a disease

Olumbe is a bad health condition that fails to be treated or cured.

Olumbe teluwona – Olumbe cannot be treated

Olumbe lutta, - Olumbe kills

Olumbe omuntu affa nalwo - olumbe, one dies with it

Olumbe lulabika oba obutalabika – Olumbe may or may not be visible with physical signs and symptoms

Words that describe disease

Obulwadde nga omutwe okukuluma nga gulina kyeguvunaana oba kyegulanga. – obulwadde is when you have a headache as a symptom for an issue that needs to be addressed or an information for what likely to happen in the future.

*Bulwadde omutwe gumbobba, (*throbbing headache), *Kamunguluze (dizziness); meeme ensidukirira, (nausea)*

Obulwadde bujjanjabibwa nebuwona – obulwadde can be treated and cured

Obulwadde bwe buvaamu olumbe; Obulwadde results into Olumbe

Obulwadde bufuuka olumbe – a disease may turn into an illness

Obulwadde are things like Malaria, flue, which can be treated.

Health management

Kusawula is to understand the health problem, its root causes and treatment or management

Our clients are referred to as *Bazukulu*.

These spirits (empewo) have different ways of carrying out their healthcare services. I believe that these spirits sit among themselves and reach a consensus and explain to the clan members (abekika) or the trainer (Senkulu) on the way they will carry out their healthcare practices

Emyoyo are the spirits like Misambwa, Mayembe, Lubaale, Balongo etc. I believe that those spirits are the ones which carry out healthcare services on behalf of God. I use plants, water, minerals, birds, animals in healthcare services.

The way these spirits work may not be able to be understood by a lay person or human being (balyamere). The Spirits decide on how to carry out activities depending on the gravity of the issue

All the spirits have a role in healthcare practice though they have different specialties.

Spirits can be used to protect, prevent, treat, maintain health and promote health

causes of illness and diseases

Ebbanja lileta olumbe n'obulwadde – A debt may be the cause of illness and disease

ebbanja lisobola okuba mubujajja, mumpewo, mubintu byabulujo, lyansimbi oba mumyoyo. - The debt may be ancestral, spiritual, material, financial, or moral

ddogo (witchcraft)

ebbanja mu lubaale – Lubaale nga abanja – Ancestral spirits demands.

Lubaale abanja bubi nyo ensanji zino – ancestral demands are very serious these days.

Obweyamo obwakolebwa abatusokawo bufuuka ebizibu – The promises or covenants made by our ancestors become binding to use and sources of problems

Okunywa oluzaalo – omwana nelumulondoola – The promises made when addressing infertility may follow and become a problem to the child when not fulfilled

Lubaale nga tateledde bulwadde – unsettled ancestral spirits are a cause of health problems

Causes of *lumbe* (illness)

Omwaana nga akayanirwa asobola okubeera nolumbe obutawona mpaka nga azibwa mu kika kye ekituufu – A child misplaced in a clan may have an illness, non-responsive to treatment, until the child is returned to the demanding rightful clan

Causes of *bulwadde* (disease)

Ebbanja bulwadde naddala obulwadde obuletebwa ebbanja mu Lubaale

Witchcraft (ddogo)

Witchcraft may cause illness and disease

Ebiteega is part of witchcraft. – Biteega are Barundi spirits that can be sent to physically, spiritually and mentally torment another through illness, bad habits like theft, madness or doing evil things. The witchcraft may extend to animals

Ddogo (Witchcraft) is a word that describes both illness and disease that affect the physical, mental, spiritual, social and economic aspects of person or family

Ddogo is invisible to the naked eye but its effects are visible

Ddoga can be managed or removed through traditional methods including animal sacrifice and ritual cleansing (*Kwambulula*).

Dogo (witchcraft) can be invisible, spiritual, physical and mental.

Kisirani

Kisirani is associated with bad luck, whatever is done does not work out well.

Kisirani is associated with misfortunes and failures in employment, marriage, family, business etc.

Ekisirani nkikolako, nsoka kumanya nsibuko yakyo nga nkozesa omweso, nensizira awo okukijjawo – I handle *kisirani*, I use *mweso* to establish its root cause first, then manage accordingly

Kisirani tekirabika kikwata myoyo – *Kisirani* is not visible it is spiritual

Some bad habits are brought about by spirits for example *Omuze gwa Kaseegu guletebwa emandwa ya* ***Kaseegu***

health assessment and diagnosis

client health assessment by spiritualists may or may not engaged ancestral spirits possession, and use of diagnostic set of tools (*mweso*)

olumbe n'obulwadde birina obubonero n'ebikolwa - health problems are associated with signs and symptoms

people consult with us because they suspect their health problems are spiritual or have failed to respond to biomedical approaches

Nzijanjaba ebizibu, olumbe n'obulwadde ebirabika nebitalabika kuba nsobola okumanya ekilese omulwadde nokukolako nga sogereganyiza naye. - I manage clients' problems, illness and diseases, both visible and invisible, because I have some power and ability to know about the client, the reasons for coming, the problems they have, and how to solve their problems without physically engaging them

omulwadde asobola okuja nga namufunyeko obubaka - A client might enter my shrine when I already have got information about him or her.

Nkimanyi nti empewo zirina obusobozi okulaba n'okujanjaba – I believe the ancestral spirits have the ability to diagnose and treat

The traditional healthcare spiritualists become compatible with their *mweso*

manya olumbe n'obulwadde okusinzira kunyinyonyola yomulwadde, ebyafayobye, ennono yobuzaale bwe, n'obubonero - I can know the health situation of the client from the explanations of his/her problems, the ancestral history and the sign and symptoms. *bwembusabusamu nga nkozesa omweso* - When I am in any doubt, I use *omweso* (diagnostic tools).

nfuna ebiroto nenonyereza amakulu gabyo okumanya ekyokolera omulwadde - I get dreams, look for meaning or interpretation in order to understand what I need to do for the client.

olusi omuzukulu tekimwetagisa kunyonyola kimulese, omweso gumbulira - At times the client does not need to explain his/her problem, the *mweso*, the diagnostic tool, informs me.

nesiga nyo omweso okusinga omuzukulu byangamba – I trust more the *mweso* (diagnostic tool) than what the client tells me.

I prefer using *omweso* for understanding the problem and its cause than what the client thinks. In many instances what the client thinks is contradicted by the findings and revelations of *omweso*.

Reading, understanding and diagnosing by use of *mweso* is the responsibility of the spirits possessing me during that time.

Amaaso gasobola okulaga obulwadde bwa Wamala oba Kawumpuli)

Kulagula

Okulagula is when a client seeks and consultation for understanding and elaboration

Kwaaza

Okwaza is a diagnostic process to establish the cause of a situation

Omweso (diagnostic tools)

Omweso is a diagnostic set of tools used by ancestral spirits for health assessment, divination and diagnosis.

Omweso is a diagnostic tool requested for by the ancestral spirit that uses it. When the ancestral spirit requests for its *mweso,* the Ssenkulu to know its constituents and the rituals required to empower it for perfection.

Omweso ngukozese mu kukebera n'okukagula – I use *omweso* (Traditional diagnostic tools set) for health assessment and divination.

Omweso gwange nkozesa Engatto za Muwanga, (gwa amaliba gensolo Mwenda (9) okuli Nbogo, Mpologoma, Nsama, Ngo) – Omweso gwa Muwanga is my assessment and divining tools set. it is composed of nine (9) pieces of hides of strong wild animals including Buffaro, Lion, Nsama and Leopard.

The use of the diagnostic tools is a process that can take time to learn and understand, and be able to interpret to make sense out of the way they elements position themselves and relate to each other within particular environment and specific words used

Omweso gukozesebwa mu kulagula n'okulebera - Diagnostic tool (*omweso*) is used in divination and diagnosis

Okukozesa omweso si bikwaate naye okusoma ebirambikidwa musengeka yo mweso – the use of a diagnostic tool is not cram-work, but a reading of what is revealed by the layout of the displayed diagnostic tools.

Prevention, protection and Treatment

kusalula banja – payment of the debt

A *mutugwa* is indigenous in Arusha and Moshi which is mostly carried by VIPs as hand sticks are protective measure. There are also others like mutatembwa, ntalogwa, which are used for protective purposes against illnesses, diseases and other situations

Kuganga

Okuganga and kusandaga are used to protect.

Kwambulula

Okwambulula (ritual cleansing) is done by use of natural materials such as birds, animals, and plants, for health to manage and improvement of an individual or family

Yirizi

Yirizi is used to prevent and protect.

Prayers

Prayer is used to confirm (kukatiriza) what I want, but the spirits already know what I want. I believe that whatever I do and what happens the spirits already know. Prayer is when you speak out with your mouth to the spirits what you want but the spirits already know what you want.

You pray to confirm to the spirits that you believe in them. Prayers also assure the spirits that you believe in them their abilities and power. The spirits can give you whatever you request them. I believe that the spirits know whatever I need. I pray to the spirits in order to confirm to the spirits that I believe in them and respect them.

Communal prayers are more powerful than prayers by a single individual as the saying goes that "*agali awamu ge galuma enyama" and "endege ziba nyingi nezivuga"* . Each personal word has different impact (*buli kigambo kyomuntu kizitowa lwakyo*) so when combined they have a stronger impact.

health promotion

Okwetereza is a word used to promote health. Okwetereza sometimes comes after some problematic situations. (omala kuggwa n'omanya ekikusudde).

Spirits

I believe that each human being has personal spirits. The sum of the spirits of different people means power. (*omugate gwe myoyo gemanyi*).

Spirits are specialised.

Spirits collaborate with other spirits

When one spirit is given a task, it collaborates with other spirits to accomplish the given task.

Katonda

"Katonda *ali wagulu wa byona, mukumanya n'okutegera, yeyatonda emyoyo, kwakkira kunsi, mwakolera era mwayitira okuwonya. Katonda ali mubuli kyeyatonda era y'awa obulamu"* - Katonda is supreme of all, He created the spirits, which spirits He uses to descend on earth, to carry out his duties including healing. Katonda is in everything He created and is the giver of life.

For instance, the spirits may predict or prophesy something and it comes to happen. A client may come with a problem of infertility and the spirits tell the cause, spit on you and later you produce children. So, I believe that the spirits do exist and they descend onto us and make possible for us (traditional healthcare spiritualists) to carry out healthcare services.

Spiritual power is imbued into herbal medicine

Spiritual powers are imbued into the regalia such as spears

Abantu bwe basobya Katonda, Katonda yabasindikira obubaka nga bwa lumbe lwa Kawumpuli era nasindika nomuntu nga mukweeke (kiwuduwudu) eyalina obubonero n'obuyinza okuwonya obulwadde obwo. abantu bangi basindikibwanga mukifo omwali omuntu oyo okuwonyezebwa obwo. kwekwaava elinya kya Kawumpuli otutumibwa obulwadde, omuntu ayabujjanjaba n'empewo ezakwatibwangako. When Katonda was wronged by the people, He sent a message through a mysterious illness. He also sent a person, in a camouflaged image (kiwuduwudu) who could treat that illness. many people were taken to that person for treatment, Both the illness and the person gifted to treat it and the spirits responsible were all given the name Kawumpuli

The powers and authority of spirits is given by God

Above everything, there is Tonda (God)

Ancestral spirits (Lubaale)

Ancestral Spirits are specialised in their health management functions and responsibilities and they refer health cases within themselves, by calling upon specific spirits for particular health conditions.

Empewo eziri mubutoozi tezeganya mulimu gwa kujanjaba – Ancestral spirits with natural powers are willing to be given any health care work

The Lubaale of the 1980's when I did my Kusamira, and the Lubaale of today have differences in their responses to the promises by human beings.

The Lubaale of 1980's I needed fewer things for harmonization than the Lubaale of these days.

The Lubaale of 1980's could depend on promises for it to act positively which is not the case for the Lubaale of these days. You could *Okumusimbira ekitooke "Salongo, nsimbye ekitooke ekyo bwekirikula netuyiisa omwenge netufuna sente tugenda kubakola muteleere" Basibanga ebitoogo nti y'endiga (sheep), Basibaanga empumumpu, Basibaanga akakalu ka 'wafu emu'* -- *"Mukirize omuzukulu asome, bwaliba akuze nategeela alibakola nemuteleera"* - *Kaakati obukalu tebukyasobola kukola*, which is not the case these days.
Lubaale could even respond positively upon pure lies, but not these days. *Kati Lubaale takyalimbibwa. Bamulimbalimba dda. Neba jjajja ffe bamulimbalimba dda 'yak*oowa'. Kati Lubaale akuyamba buyambi nga osabye musabe okukuyamba.

The specifics, roles and functions of ancestral spirits vary within the various clans, but are generally similar for a particular clan.

empewo ezimu zirina obukugu obwenjawulo mukujjanjaba - some spirits are specialized in their healthcare practice.

Specialised diagnostic spirits are not necessarily the best prescribers nor dispensers.

The spirit decides what type of treatment to give a patient depending on the gravity of the problem.

Lubaale namuwa ebibye byeyasaba era memutereeza meaning I did whatever Lubaale demanded to harmonise it.

Spirits decide how their things are done. One time My Senkulu kiwalabye wanted to kusiba maliba of her daughters. He used his diagnostic tools (n'akuba engato) in order to know who should do it. He did it on seven people but the spirits identified me to do the job much as I was the least experienced among them all.

Omwoyo mulamu naye nga tegulina mubiri wadde omusaayi – Omwoyo is alive and living but without a physical body nor blood.

Lubaale historically are myoyo and what I know is that centuries before some of them were human beings. Some are created (mitondere dala mitonzi). Like Tonda ebutonda.

Other Myoyo like Muwanga, Mukasa had human parents so they are ancestral. Mukasa is son of Wannema. Wannema is a son of Kitinda and Kitinda is a son of Musisi. Lubaale are myoyo (spirits) we found on earth.

Mandwa is another name used for the spirits

The ancestral Lubaale in each clan/family has its uniqueness or particularities.

Lubaale can cause illness and disease,

Lubaale belongs to the clan,

Lubaale can heal illness and disease.

Lubaale can bring or cause problems on the physical body, mental and at spiritual level.

Not every spirit works on the healthcare needs of a person

ancestral spirits good in health education include

Some spirits advise other spirits (*emisambwa ejjimu gyegigolola ginagyo*). Spirit Muwanga *atereza* Lubaale Mukasa. When harmonizing Lubaale Mukasa, Lubaale Muwanga that stand in for Lubaale Mukasa (*Muwanga yaberawo*) and during the harmonization process at the fire place Spirit Muwanga says that I am your parent (*akuzadde mu diro nze Muwanga*). *Muwanga yatereza Lubaale n'emisambwa emirala*.

Misambwa

Misambwa emitonde were found on earth and their origin is unknow to man.

The Misambwa move in reptiles like snakes and pythons.

Spirits of Mayembe and Misambwa perform specific duties as assigned to them by the spirit on duty of the overall spirits such as Muwanga, Mukase, Kawumpuli and Muzimu

Misambwa do collaborate

Muwanga

Muwanga is a general spirit, he is the overall spirit in my shrine

Muwanga collaborate with Jembe Lubowa

Muwanga collaborate with other Mayembe through Jembe Lubowa.

emirimu eminene gikolebwa Muwanga omuli okutendeka okulagula, okuwanga amayembe n'emisambwa – big assignments are carried out by spirit Muwanga like training, diagnosis, and empowering of Mayembe and Misambwa.

Muwanga yomu naye ayawukanya enono okusiinziira ku kika kye - Muwanga is the same but differ its specific characteristics depending on the clan norms and ancestral lineage powers.

Omweso gwa Muwanga gwawukana okusinzira nono ye kika Muwanga mwasibuka n'emikolo egyakukolebwako – The constituent of *Mweso* for Muwanda differ depending on the clan to which Muwanga belongs and the rituals performed on it.

Each Muwanga gets accustomed to its *Mweso* and its constituents

Muwanga mandwa Muwanga asamirwa, Muwanga Mukozi naye waliwo ne Muwanga atali mukozi. Muwanga is a Spirit, animal is sacrificed for its harmonization and it is very hardworking. However, there is Spirit Muwanga which is not working

Muwanga is Lubaale

Muwanga is Lubaale.

Spirit Muwanga has a spear and *empiima, olutembe, Ddamula*. Muwanga uses those symbols in healthcare practice.

Muwanga asalirwa enume y'ente myuufu nga yakaasa (Photo) n'embuzi nume myufu eyoluwonzi oluddugavu kumugongo (Photo) n'enkoko eyalujumba emyuufu (photo) – A male brown bull with a spot in the front of the head (photo) and a male brown goat with a black trip running at the top of its back, from head to tail (Photo), and a male brown (lujumba) chicken (photo) are sacrificed for Muwanga.

Muwanga as a human being had his fighter Mayembe including Lubowa, Namuzinda, Kilarile, Kalondoozi, Kawagga, to protect its Lubaale and the grandchildren.

Muwanga used to sacrifice animals and birds for its Mayembe to rejuvenate and strengthen the powers of his Mayembe.

Muwanga followed the traditional norms of Lubaale, - *Muwanga yagoberera ennono y'enkuliiti*

Kawumpuli

Kawumpuli musambwa – Kawumuli is a Musambwa

Kawumpuli is the assistant to Muwanga

Kawumpuli: Kawumpuli is a good spirit in offering health education and health management or treatment

Kawumpuli has a spear which is black in color, *engabo, ekifundikwa*. Every Lubaale spirit has a *kifundikwa*. Kawumpuli is a Mulangira and a Musambwa

Kawumpuli was born as a crippler (*Kipumpuli*).

Kawumpuli is the assistant to Muwanga.

Spirit Kawumpuli ayambulula (does the cleansing activities), Kawumpuli awanga (empowers other spirits), Kawumpuli does other duties as they may come up or as assigned by Spirit Muwanga.

Spirit Kawumpuli is a Katikiro of all spirits (Prime Minister).

How does Kawumpuli spirit work in healing?

Empewo ya Kawumpuli ajjanjabila mu kabonero ke vvu; naddala okulisiga kumubiri oba okulabira mubirooto - Kawumpuli spirit does his healing work mainly through the symbolism of ash; especially physically smearing it on the body or experiencing it in the dreams.

When the main spirit of the shrine is Kawumpuli, all the healing activities rotate around Kawumpuli and is the head of all the healing powers in that family.

Kawumpuli is a mulangira (prince) in the linage of misambwa. Kawumpuli is a son of Ssekabaka Kayemba and Mukadde Nakku.

Ndawula

I also work with Ndawula prince spirit

Ndawula aleta obubonero. Ndawula spirit brings about signs and symptoms

Spirits of Bakabaka or those of Balangira do not have restricted ancestral connections, they can possess anybody of their choice in any clan in Bugands.

Kabaka Ndawula and Mulangira Ndawula are not the same Spirits. Kabaka Ndawula and Mulangira Ndawula are different and they ask for different things for their harmonization.

Kabaka Ndawula is given a backcloth (*Olubugo)*

Mulangira Ndawula is given a *Kifundikwa*

The late kings used to perform rituals and ceremonies to prevent community problems

When Ndawula appears, his requirement include his smoking pipe, his Kanzu long dress and his walking stick

Mukasa

My shrine for Lubaale Mukasa is called Kagerekamu. It constitutes

Lubaale Mukasa must have enkanamu, effumu, olutembe, eryato, ensuwa, he might have a shrine called kagerekamu, ensuwa, ensumbi and any other as the spirit may demand.

Lubaale Mukasa Kabaka, and normally works through delegation.

Mukasa has a silver spear

Spirit Mukasa is a Kabaka (Royal King) so this Spirit Mukasa does not directly carry out healthcare services, but delegates such activities and duties to other spirits such as Spirit Ddungu

Lubaale Mukasa ye *nannyini bweza, yagaba ezzadde* is associated with child bearing.

When Lubaale Mukasa and Lubaale Musoke are harmonized, there is hope for producing children In the family.

Lubaale Mukasa can cause one to feel cold all the time (atintimya buli kadde), can also cause loss of teeth when he is demanding *ensimo.*

Lubaale Mukasa agaba ezzadde

The color of the animals which are sacrificed for Lubaale Mukasa is a white male goat,

Mukasa collaborates with other spirits.

Mukasa works through delegating other specialised spirits

Mukasa may refer a client directly to a specialised spirit for an appropriate ritual or action. For example if a person seeking good luck and Mukasa realises that the client needs to first harmonise with spirit Kiwanuka, Mukasa will refer the client to Kiwanuka for the needful to be done so as to actualise the good luck.

Spirits Mukasa, Kiwanuka and Musoke do not directly work with Mayembe but refer to Muwanga who then instructs the Mayembe through Jembe Lubowa

Kadduwanema

Kadduwanema is one of the major spirits endowed with powers and abilities in health management.

Kadduwanema is not very common in health management but consulted by junior spirits when issues of health management get diffiuclt.

Kiwanuka

In some clans/families, Lubaale Kiwanuka must have a shrine containing a muvubo-gwesezo-sasa, yet it is not the case in other clans/families

In most cases Lubaale Mukasa is closely related with Lubaale Kiwanuka, and Lubaale Musoke. The process of Kusamira Lubaale normally is done in the same seating period for the three Lubaale Mukasa, Lubaale Kiwanuka and Lubaale Musoke. However, there other Lubaale that include; Lubaale, Kitinda, Lubaale Wannema, Lubaale Musisi etc.

Lubaale Kiwanuka asuula abant *eddalu.* Lubaale Kiwanuka is the one responsible for madness in people.

Lubaale Kiwanuka must have *ekiwu* which is made from the skin/hide of the brown adult male sheep sacrificed to harmonize Lubaale Kiwanuka.

Lubaale Kiwanuka has a brown spear (effumu lya kiwanuka limyufu) and e*nyondo emyuufu* both of which are usually made of copper

Lubaale Kiwanuka can cause mental disorders (eddalu).

Lubaale Kiwanuka heals headaches, *entununsi*

Musoke

In our shrine we gave Lubaale Musoke a female goat. We gave Lubaale Mukasa a white male goat and a white male cock. This is what is done in Baganda

The color of the animals which are sacrificed for Lubaale Musoke is a female goat embuzi *luyina omweru oba omumyufu*

Lubaale **Musoke** yaletera omukyala okulemera munsonga. Meaning that Lubaale Musoke is the one responsible for causing Menorrhagia – abnormally prolonged menstruation periods

Musoke has a bronze spear

Lubaale Musoke can cause infertility, miscarriages

When Lubaale Musoke has demands to make, it can cause symptoms related to infertility, frequent thirst, frequent urinations, and this may be relieved by provision of Ensumbi for Lubaale Musoke

Muzimu

I believe that when I die I will become a Muzimu. *gwendivvunukirako alinanya obuzaale bwange nempewo zenakolanga nazo.* So when I come back as a spirit Muzimu, the person I will possess will clearly state my lineage and the spirits I am worked with.

I believe that when I die I will become a Muzimu. *gwendivvunukirako alinanya obuzaale bwange nempewo zenakolanga nazo.* So when I come back as a spirit Muzimu, the person I will possess will clearly state my lineage and the spirits I am worked with.

The Muzimu belongs to the clan (kika) and serves the clan and the community

The Muzimu does not belong to an individual. The Muzimu has its own bulombolombo.

It might want its shrine, its rituals, it might want an animal. The Muzimu has its own reasons to request for a particular animal or anything it demands for.

The Muzimu might for instance demand for a female goat and after it produces you can once in a while ick from the siblings and feast. The Muzimu might want the goat to be a shock absorber any problems which would have otherwise affected the family members. The muzimu might ask for engabo (shield). The spear belonging to the Muzimu- *effumu liba lya kyanga*. The muzimu has a Kifundikwa and a Kanzu which are its dressings. A Muzimu might ask to have a fire place (Kyoto). In my setting my Muzimu has no Kyoto but has a shrine (*enyumba yo Muzimu weri*). The structure of the shrine depends on the particular Muzimu depending on its particular reasons. Entindi is like a store or porch where secretive things belonging to the spirits are kept

Omuzimu comes into play on occasions which are specific, the omuzimu is the spirit which plays the role of overseeing these spirits. The omuzimu was originally a human being and those spirits were the ones he/she used to work with when he/she was alive.

In my family we have male and female Mizimu.

Our female Muzimu is called Nakasi. Nakasi was a female traditional healer, specialised in handling infertility in female, and destroying court cases.

Our main male Muzimu, (name withdrawn) in the family had a twin spirit (*gweyawanga mu Banda*) that was responsible for all the riches in the family. Many family members attempted, in vain, to attract this Muzimu because of its riches. However, at the end, it selected the least expected 14-year-old boy in secondary school (senior two) (name withdrawn). The young boy was the one responsible for the Muzimu, its twin spirit and the associated powers and riches.

Mayembe

Amayembe is not Lubaale.

Amayembe are also spirits (myoyo).

Mayembe are the spirits that carry out the required healthcare activities as instructed by Muzimu and Misambwa spirits.

Balongo

What are Balongo

Abalongo are categorized into *abalongo abazaale* (ancestral twins) and *abalongo abatonde* (Natural twins). Balongo manifest as twin spirits, twin forces and twin children. Some Balongo are Misambwa. All types of Balongo are important in healthcare practices

Buli kikolebwa balongo bebakulembezebwa meaning that whatever is done, the rituals of Balongo are done affront

Abalongo balongoosa Balongo rectify situations for the better

Twin spirits are empowered by Muwanga.

Ddungu

Omusambwa Ddungu munonyi gwa nsimbi n'emikisa. Obuzibu bwomuzukulu bwe buba bwansimbi oba emikisa, omusambwa Dungu gwegumukolako. Musambwa Ddungu is specialised for hunting for money and good luck. When a client's problem is about money, or need of good luck, spirit Ddungu is called in to address the issue.

Ddungu; Ddungu is a good spirit in offering health education

My Ddungu spirit collaborate with Kayizzi and other spirits especially looking of good lack for clients.

When my clients come seeking of good lack, Mukasa sends me to pray at Ddungu fire-place who then collaborates with Kayizzi to hunt for good lack.

Mayembe

Some Mayembe are natural (*matonzi*). The bible talks about Mayembe.

Mayembe are helper spirits and are soldiers (*Amayembe mayambi, masirikale*).

Mayembe protect their grandchildren or the slaves of their masters.

Mayembe help the Misambwa in different services.

Mayembe do not receive instructions from Mukasa, Kiwanuka or Musoke. However in the process of currying out or implementing their activities, Mayembe can freely interact with Mukasa, Kiwanuka or Musoke spirits to accomplish their duties.

Kinene – Bulamu

Kinene batenda is spirit Bulamu and Kinene nyumba is Walumbe are twins

Natural places

"*Nkimanyi nti ebifo byobutonde bilina amanyi*" I believe that different natural places (ebifo by'obutonde) have different power (amanyi).

There is a time when people whose belongings had been stolen, I would go to a mountain and carry out some rituals and they would retrieve their belongings from the thieves. I would carry out similar rituals in another place but power would not work as the powers experienced from the mountain.

Waliwo ebifo mubutoned ebilina amanyi n'obuyinza - There are places with natural powers and abilities

The use of herbs and other medicines may fail to treat obulwadde or olumbe. Yet I may just go to a Plant of Kiwanuka or Mukasa which was planted and imbued with power for specific use, I use local brew and pray for that power to raise and I use it to effect a healing.

There are places where if you know what to do, and how to do it, you just kneel and request for what you want and you will get it. However, such places have been destroyed and lost such powers. *Enkayana mubifo bino zibadeyo nyingi* Examples of such places with natural powers; Ewa Kibuuka, Ebusabala, Essese, Ewa Ttonda ebutonda nebilala bingi

I advise researchers like to go to such places, kneel and pray for increased powers, financial abilities, knowledge, and wisdom, (*obusobozi, kumanya n'okutegera*). These powers to help you to get to the right people with the knowledge and powers, people who would not derail you.

Symbols and symbolism

The symbols for the Spirits represent connections with the specific spiritual entities in space which, if in harmony, are responsible for the peace, health on earth. Otherwise, if out of harmony are responsible problems, illness and diseases

The color of the animals which are sacrificed for Lubaale Mukasa is a white male goat, Musoke embuzi *luyina omweru oba omumyufu* (female goat)

Regalia

Symbolic spears, walking sticks, *ekiwu*, *olutembe*, … for specific spirits are imbued with *amanyi n'obuyinza*, which powers can be called upon and they respond appropriately. This is approximated to writing a letter, which you sign and stamp

Traditionally, some places are demarcated for specific spirits. Such places include trees like *Omukokowe* for Lubaale Kiwanuka. These symbolic places and trees may be imbued with *amanyi n'obuyinza* through ritualistic processes and ceremonies that meant to *Kunyeza n'okukakasa* the imbued *amanyi n'obuyinza*

Spears

We, *Balubaale* (traditional healthcare spiritualists) make use of spears. I believe that the spears are tool for fighting. Those spears you see there were not put there as a decoration. Those spears are symbols of the spirits and if one of the spears is missing, the owner (particular spirit) will demand for it and if I delay, I may experience olumbe. That is why those symbols are placed there for purpose. Particular spirits can demand for *ngabo* (a shield).

Symbols like spears are specified for specific spirits like Mizimu, Misambwa, Mayembe, Lubaale.

Ekiwu

Ekiwu is made from the skin/hide of the brown adult male sheep sacrificed to harmonize Lubaale Kiwanuka. *Ekiwu* is used for seating on while the person possessed by the Lubaale is carrying out the healthcare functional services.

Ekiwu is the skin taken from the animal which was sacrificed for that particular spirit on which he sits while carrying out his services.
