# Supplementary material for: Perspectives on health, illness, disease and management approaches among Baganda traditional spiritual healers in Central Uganda
Source: PLOS Glob Public Health. 2024 Sep 6;4(9):e0002453. doi: 10.1371/journal.pgph.0002453 (PMC11379289; doi:10.1371/journal.pgph.0002453)
Supplement: S6 Data — (PDF) [file pgph.0002453.s006.pdf]

Study participant 6 - transcription

Contents

Study participant 6 - transcription 1

Socio-demographics 2

Historical background of Mamba clan 3

Shrine 3

Muwanga shrine (Esabo lya Muwanga) 3

Places with spiritual powers 4

Natural places 4

Shrines 4

Mulubaale 5

Problems associated with becoming a Mulubaale 5

Becoming a Mulubaale 6

Senkulu 7

Mweso (Diagnostic tools) 8

Ancestral Spirits (Lubaale) 8

Misambwa 9

Muwanga 9

Ndawula 10

Kawumpuli 11

Lubaale we nyanja Mukasa, Musoke, Kiwanuka 11

Mukasa 12

Kiwanuka 12

Musoke 13

Ddungu 13

Muzimu 13

What is a Muzimu? 14

Mayembe 15

Jembe Lubowa 16

Namuzinda 16

Kasajja Lwazi 16

Kilarile 16

Balongo / Twin forces 16

Abalongo abazaale 17

Abalongo abatonde (Natural twin forces) 17

Healthcare management ... 17
Causes of illness and disease ... 18
Spiritual causes ... 18
Witchcraft ... 18
Health assessment (diagnosis) ... 18
Treatment and healthcare ... 19
Rituals ... 19
Prayers ... 20
Treatment of Olumbe ... 20
Communal meals and ceremonies ... 21
Treatment of obulwadde ... 21
Prevention and protection ... 21
Okuganga ... 21
Muwambo ... 21
Health promotion ... 21
Words and phrases that describe health ... 22
Words and phrases that describe illness ... 22
Lumbe ... 22
Kisirani ... 22
Kulamiriza ... 22
Biteega / Kiteega ... 23
Nyabingi / Nabingi ... 23
Words and phrases that describe disease ... 23
Symbols and symbolism ... 23
Sex in spirituality ... 25
Taboo ... 25

Socio-demographics

My name is (name withdrawn). I do not use my Christian name because I not a Christian, I am a traditionalist. I am a Mulubaale. I am a 65-year Muganda Female widow and belong to Mamba clan, Kakoboza of Bukerekere from Kakiri. I belong to Uganda N'eddagala N'obuwangwa Bwafe Traditional Healer's Association. This to have unity. You cannot do something alone.

I belong to the Traditional religion. I stopped in Primary Four (P.4). I am a traditional healthcare spiritualist with 32 year working experience. I also do subsistence farming. I stay here in Singo County (Saza), Mubende District / Mityana, (xx) Parish, (x) village.

I train traditional healthcare spiritualists

Historical background of Mamba clan

My clan is the original Mamba clan and it has important rituals it performs on the king of Buganda kingdom. When the king dies, the next king goes to Bukerekere, for rituals to be performed upon him. His hair is shaved (*amusula ejjoba*) and the head of the clan (*Nankere*) removes his inner pants and the king also removes his pants and they both exchange the pants. That is why you hear that the descendants of the Mamba of Kakoboza of Bukerekere clan are princes and princesses.

In the past there are around 8 descendants of the clan (Bakerekere) who were kings of Buganda. After the king ascends to the throne, the Nakenkere never sets his eyes on the king not until he dies and he will see his successor during his rituals, there is a reason for it. They roast a banana and they cut it into two and share it. The shrine belonging to the clan is called Namiryango, after the rituals the king passes through one entrance and the Nankere passes through the other. They do not use a razorblade to shave the king's hair but there is a tool called Kamweno which is used. They use it on the king, then wrapped properly and is kept for another king who will have succeeded the current king after his death.

Shrine

We had a family shrine, however, when man father returned from Rome pilgrimage, he instructed people who burnt it down. Although after many years, he also died of fire burns.

I got a spiritual message construct another clan

My shrine is a Sabo. Here in my shrine, the spiritual power (*amanyi*) in this shrine is derived from the spirits existing in the shrine.

Our clan shrine (*Kiggwa*) was at Kiwanga village. Our Kiggwa shrine was shifted to Kisaana 30 miles on Mityana road because the land was sold when our grandfathers died. However, when my father after visiting Rome, he thought it Satanic to have the shrine at the land he had control over. My father used the child of my aunt to burn down the Kiggwa (clan shrine). Fortunately, the Lubaale regalia was not in the shrine so it was not burnt. *Lubaale teyaliimu mu Kiggwa era Lubaale teyayokebwa.*

The main spirit in this Sabo shrine is Muwanga with Kawumpuli as his Katikiro.

Muwanga shrine (Esabo lya Muwanga)

When building Muwanga's shrine, it is the spirit which selects the place and empowers the place where the shrine is built.

The spirits had already explained that we used to stay at Mawundwe and our land (kibanja) is occupied by one Sepiriya. My grandfather said that he hears of Separiya at Mawundwe. When we reached at Mawundwe, we found Sepiriya there. We told him that we have come after getting a message from the spirits. The spirit Muzimu possessed me and told Sepiriya that these are my grandchildren. This land (kibanja) was ours. The Muzimu spirit told Sepiriya that there

is some place where there was a pot full of “emumbwa”. Sepiriya agreed that was true. The spirit muzimu narrated the history of the place and what used to be its residence. The Muzimu spirit said that I want you to go where I was buried and on my tomb grew a kirundu tree. The Muzimu spirit instructed us to cut a side branch which is going to act as the pillar of my shrine. We had not built the shrine as yet. That is what we also did. After cutting the tree it rained heavily and we all got soaked by water. We put the tree on a truck. We went and cut rids and other trees. Those days we never used to construct shrines using blocks but we used rids and mud.

The shrine was built in one day, We built the shrine in one day and finished it. Muzimu spirit told us that he refused us to carry out his ceremonies at the trainer’s shrine because you have taken a long time looking for what you do not know and now you have discovered it we have brought back in the family, wealth, grandchildren but not to carry out ceremonies at the Senkulu’s to leave your luck there. Afterwards we all got money and become very rich.

Places with spiritual powers

Natural places

Ebifo byobutonde birina amanyi gobutonzi mumpewo. Natural places do have spiritual powers.

Emiti gilina amanyi g;obutonzi ageyolekera mu mpewo Trees have spiritual powers.

Amanyi g’emiti egili mubifo ebitonde masukulumu. The combination of spiritual powers of trees within natural places is much higher.

Omuntu asobola okufuna obujjanjabi obuva mubifo ebyobutonde naddala nga abifunyeeka obubaka. One can get healthcare services from natural places on messages from ancestral spirits.

There are people who stay on the sacred places as custodians, but not necessarily traditional healthcare spiritualists.

Shrines

However big the shrine is, it is supposed to be built in one day.

Mpanga amasabo – I have the authority to empower shrines with spiritual powers.

I told the family to build another shrine here at Tula since the one in the village had been burnt down. I bought all the things required for the construction of the shrine. On the eve of the ceremony one of my paternal aunts (*senga*) instructed me not to construct the shrine because I was a widow. My aunt asked me who was to be the head of the shrine, and I answered that the head would be my grandfather as we had done for the previous shrine. (*Akasolya bakalasiza ku jaaja wange, kubanga nze ndi mukyala*). The head had to be my grandfather because I was a lady. Even though I am possessed by spirit Muwanga, and a male Muzimu I cannot be a head of the shrine because I am a woman (female). But my aunt objected to the construction of the shrine at my home. So I gave the animals which were meant for sacrifice to the family members to eat them. From then she got seriously ill which forced her to send her son to me instructing

for the construction of the shrine. We arranged with family members and built the shrine though she did not attend the ceremonies. The sickness did not stop. They consulted a healer who told them that she was sick but had wronged the spirits. The healer gave them medicine so that they could release her from the hospital bed (Okumusibula mu dwaliro) which was done and she was released. The healer instructed them after they release them from hospital, the patient should be taken straight to the shrine. She was also advised to make a meal for the spirits to appease them. Instead she went direct to her home. She prepared a meal for the spirits at my aunt's place who also had spirits as I had told you earlier. Those spirits warned her that, that meal was meant for the spirits she had wronged when she stopped the ceremonies of constructing the shrine. She ended up dying. The sickness was as if she had fire originating from the legs.

Mulubaale

Sibuli alinyibwako empewo nti mujjanjabi – not all spirit mediums are healers.

When ancestral spirits possessed a person, they are asked if they had healthcare functions.

Problems associated with becoming a Mulubaale

Lubaale abonyabonya mungeli nyinji. Obuzibu bwo lumbe mwanyinaze bweyalwala bwali busibuka ku Lubaale, atekwa kusamirwa". Ancestral spirits can torture in many ways. Lubaale tormented my brother to an extent of cutting off his nose. When the torture was unbearable, my brother came back to the family and surrendered to ancestral spirits (Lubaale). He had reached the extend he could not move in public because of the smelling nose-wounds. He had failed with hospitals and herbalists. He consulted many traditional healthcare spiritualists who told him the same thing that the source of his health problems was ancestral spirits (Lubaale) that needed to be harmonised with rituals.

The process of appeasing my spirits were miraculous.

It was later found out that it was spirit Ndawula responsible for his torture.

When we were about to be released from the Senkulu's shrine, my brother, who was suffering from the nose, got annoyed because the spirits had not possessed him and yet he was the one who was being tormented by the spirits, more so other spiritual healers had told him that he was the one to be possessed by the spirits. He visited a healer and he was given medicine. He bewitched me and I suffered from "*ettalo*" which almost killed me. That night the pain was too much and our Senkulu thought that the problem was due to Mayembe spirits which had not yet had their rituals done.

My tormented brother asked me if the spirits possessed me while in my mother's womb. I asked him why he had asked me such a question. He retaliated that "I have to ask you because, the spirits have tormented me to an extent of breaking off my nose but they (spirits) have not possessed me but possessed you." He picked a grudge against me ever since. The next day my brother left unceremoniously. The healer who cured me of the "*ettalo*" was called Mukasa of

Namagoma. He gave me two "*mumbwa*" one was for drinking and the other for scrubbing. Lots of puss came from the wound. Since then, my brother never used to greet me though these days he does greet me. He is alive and the nose got cured.

The Lubaale started tormenting the family members, giving us clear sign of its demands. Some of my family members were arrested, imprisoned and later killed.

Lubaale wange yabanjira ku mwanyinaze. Our family Lubaale made my brother suffer so much with a foul-smelling wound on the nose for a long time. On many consultations he was informed that the problem was related to the family Lubaale that needed to be harmonized. The family was referred to Makolo in 1972 to for ancestral spirits harmonization.

Becoming a Mulubaale

I started the *kusamira* spiritual rituals when I was still a young girl. I had given birth to my twins, but not yet married.

The family members were mobilised and contributed all the demands for kwaza Lubaale. We gathered as a family, and we went for graves yard cleaning (okulima ebijja). The grave yards has the body remains of the *Muzimu*, and one cannot determine which *Muzimu* will possess which person –*(temumanyi muzimu gwani gwemugenda kusamira).* The spirits revealed that the family had non-ancestral Mayembe spirits brought by some family members, which had to be identified, separated and removed, to facilitate the ancestral spirits to select their medium. The process ended with a communal meal.

We (family members) went to a traditional spiritual healer known as Makolo of Kitegombwa. We were there for long. When we started the process of ancestral exploration, the spirits possessed me and my paternal aunt (Senga) the spirits did not possess my brother who had a diseased nose.

I was the first to be possessed with a male Muzimu spirit of my great-grandfather Zikwa, which demanded to drink local brew and was given. The Muzimu Zikwa was of my grate-grandfather as identified by my grandfather whom he claimed was his father, although the grandfather did not know here his father had been buried. At my grandfather's request, the Muzimu Zikwa directed and led the family to Mr. Sepiriya land where his body was buried at Mawundwe on Mityana road. My grandfather said that he heard of Sepiriya at Mawundwe, and found him when reached at Mawundwe, We told him the cause of our visit.

The Muzimu told the family about the details of its Mayembe.

Nze sinywa mwenge wade emindi naye omuzimu gwa jajjange bweguninya gwo gunywa omwenge ne mindi – For me I do not use local brew nor smoke a pipe, but when the ancestral spirit possesses me it drinks local brew and smokes a pipe.

Then my aunt was possessed by a *Muzimu Lukuba* who was of a brother to the Muzimu spirit which had possessed me. The two *Mizimu* Zikwa and Lukuba were brothers.

In my case we did rituals for spirits like Musoke, Mukasa, the Mayembe-Lubowa and his wife Nakawunde at my Ssenkulu's (Makolo) shrine.

After all the spirits had come and their rituals done, Muwanga spirit, the grandfather of all spirits, was the last one to possess me. However, the rituals for spirit Muwanga took 18 days (Emyenda ebiri) and were performed at our Kiggwa shrine *(kubutaka*) at kikonge on mityana road. All these instructions were given by Muwanga spirit. Spirit Muwanga demanded for a brown bull and the hide from that bull is the one which I sit on while doing his duties.

The Kusamira process was facilitated by my elder brothers who had money so all the necessary rituals were carried out properly.

When the spirits possess someone, they should be asked how their ceremonies/rituals were performed from the ancestors. For example traditional healer Kimbowa from Buddo had his trainer (Senkulu) as Ewunike, a lady. It therefore depends on the history (*obulombolombo*) of the spirits.

Senkulu

I train others to be traditional healthcare spiritualists. I work with other traditional spiritualists who are male and I act as the grandmother.

The spirits of the trainee may participate in selecting the trainer (Senkulu). I got a spirit medium who came for training from me. When he arrived, and barely had he sat down than his ancestral spirits possessed him and communicated to me, "I have brought my spirit medium to you for training in this and that for six months. I would like him to stay with you, learn and we shall pay you this much". The spirits told me the scope of what their medium should learn from me, the duration of the training and how much I was to be paid. I did not have the chance to bargain nor an option to refuse. I accepted the trainee and the spirit guided us throughout the process.

When the spirit of the trainee possesses him/her, the spirit of the trainer also possesses the trainer (Senkulu) and introduces its self in details. For example, the case I witnessed as a researcher. A 15-years boy with his father got into the shrine of a lady who was seated on her "kiwu" dressed in her regalia with already prepared materials for a ritual of dressing the spirit of the boy. There were three knotted backcloths, two baskets (*bibbo*), a walking stick, a spear, and many plants species. The lady got possessed by a male spirit that introduced its self as Kawumpuli, to the boy seated alone on a new backcloth (*omwaliiro*) in presence of his father. The spirit Kawumpuli narrated its ancestral royal ancestral lineage in details including its both its father (Kabaka Kayembe) and mother (Nakku) and guardian mother Nabuzaana. Recounted its upbringing, how he become to be the Prime Minister (Katikiro) of all the spirits of Baganda. The spirit also introduced its medium by names (withdrawn), paternal and maternal ancestral clan lineages and their burial grounds, and then the details of the shrine. The Kawumpuli spirit made a statement that "You (Muzimu name), in case you are not comfortable with this shrine and spirits, feel free to express yourself in any way and we shall stop." Immediately, the boy got possess by a Muzimu spirit that mentioned its name (withdrawn) and affirmed the process to continue and it left the boy soon after. Kawumpuli spirit instructed its human male assistant to introduce his name, both parents and their clans and detailed his ancestral lineage. I then

requested the spirit to allow me to make audio and video recording and permission was granted by the spirit.

Kawumpuli, placed right hand on boy's head and left hand on right shoulder and loudly called upon all the ancestral spirits of the boy to come near and be in vicinity. The spirit described all the materials one by one and placed then in-front of the seated boy and told many parental words of wisdom to the ancestral spirits of the boy assumed to be in vicinity.

upon the head of the boy using his

Mweso (Diagnostic tools)

Omuzimu gwa Jajjange Mulaguzi gwegwayasa ejjoba, negusaba omweso gwagwo gwegwakozesa mubulamu, era negulungamya Ssenkulu entekateka n'empanga yagwo.- I was possessed first by the Muzimu spirit of my grand-father, Mulaguzi, who was a Mulubaale. It requested for its diagnostic tools (omweso), which it used while still in human form, directed the *Ssenkulu* its composition and ways of empowering it.

Omweso gwatekebwatekebwa era neguwangibwa Ssenkulu nga omuzimu gwajjajange gumulingamya gundikumutwe, era negunyonyola emandwa yagwo enkulu mukujanjaba – The diagnostic tools were constituted and empowered by Ssenkulu while guided by the Muzimu of my grandfather was possessing me. It explained who its major spirit for healthcare function was.

Omweso nga guwedde bulungi, Ssenkulu yagukwasa Omuzimu, nagusaba bweguba gumatide, gukozese omweso ogwo gumulagule – Ssenkulu handed over the well-constituted Mweso to the Muzimu and requested it to divine him with it if the Muzimu is satisfied with it Mweso. The Muzimu used the newly constituted mweso and divined the Ssenkulu. When Senkulu was divined, he got satisfied that the *Muzimu* was a *Mulaguzi (*Diviner), knows and can use its *mweso* properly. That marked the beginning of my *kulagula (divination) and* until now I use the same mweso (divining tools).

It is not correct to have two *mweso* that one belongs to Muwanga and the other to Kawumpuli.

You can tell which type of spirit carrying out diagnosis depending on the type of diagnostic tool it is using, for instance Kawumpuli uses *mweso* only while Muwanga can also *use engato*.

Ancestral Spirits (Lubaale)

obyinza n'obusobozi bwa Lubaale butambuzibwa mu kika – Lubaale's powers and abilities are inter-generational within the clan.

Lubaale is categorized into two Lubaale related with sea waters (*Lubaale we Nyanja*) and Lubaale of the mainland (*Lubaale wokulukalu*). *Lubaale we nyanja* is comprised of spirits like Mukasa, Musoke, Kiwanuka.

Lubaale we nyanja is similar or the same but is owned by different Mizimu

Lubaale wolukalu is comprised of Kawumpuli,

Any disputes between various spirits is referred to Muwanga.

The working capacity of the spirits depend on many factors. The working abilities of the spirits depend on the fact that they were given what they demanded, they were fully harmonised and are appeased and serviced regularly.

Spirits do not work on credit; spirits prefer to be paid instantly for their offered services.

Spirits are very cruel and merciless when there are demanding for their dues

When the spirit medium for Lubaale is a female, she has to have a male person or relative who stands in as Muko wa Lubaale when Lubaale is doing male related functions. For example *Lubaale atuzibwa kumwaliiro nga Muko wa Lubaale akute ku Mutende.*

Misambwa

Misambwa like Ndawula, Kinene and Bulamu are harmonized at 3.00 am in the morning and when it comes to 4.00 a.m they will not appear. Time 3.00 a.m is associated with Misambwa spirits.

The Misambwa spirits are associated with odd numbers like 9, 19, 29 etc.

There are *Misambwa emizaale* (born) and *Misambwa emitonde* (created-natural). The Misambwa spirits are not sacrificed for, but given fruits like pineapples, oranges, mangoes sugarcanes. The natural Misambwa like rocks are given clothes or backcloth as they might demand in messages (mu*bubaka*).

Baluntansozi mainly use prayers towards Misambwa spirits in their traditional healthcare practice as spiritualists.

Muwanga

Spirit Muwanga performs his duties and has his *engatto*

Rituals for other spirits can be done at the trainer’s shrine but Muwanga is done at the Mutende’s shrine.

Muwanga is Lubaale and is the grandfather of all spirits (mandwa). *Muwanga ye jjaja we mpewo zonna.*

Muwanga says what he is demanding. Muwanga is given a male brown goat and a male brown bull, male brown chicken (*Lujumba omumyufu*) cock regardless of the sex of the person who the spirit (Muwanga) has possessed. Muwanga is also given *omweso, engato, empiima*.

Muwanga is Lubaale wa Lukalu as is Kawumpuli

Muwanga is known in two forms Muwanga we Kaligwa and Muwanga we Nseke.

Muwanga we Kaligwa

Muwanga we Kaligwa is the original Muwanga, and he uses two sets of diagnostic set (Mweso); *Omweso gwe Ngato n'omweso gwe Nsimbi*.. Muwanga we Kaligwa, had one of his sons he named Muwanga whom he worked with learned the diagnostic skills using the Diagnostic set of multiple elements including cowry shells. However, Muwanga the son started drinking alcohol, become a drunkard and a disgrace to his father. Muwanga the son moved on with life and the place where he settled was referred to as Nseke, but continued with healthcare provision. Like his father, he continued using the diagnostic set that included cowry shell (mweso gwa Muwanga). However, he did not learn the diagnostic tool of using pieces of animal skins (mweso gwe ngatto), so in case he failed, he would refer clients to his father at Kaligwa, who made diagnosis using *mweso gwe ngato*.

Muwanga we Kaligwa, lived to old age. He accessed and worked with nature spiritual powers (*amanyi go butonzi*). When Muwangwa we Kaligwa died at very old age, his spirits continued to access and work with natural powers.

Muwanga we Kaligwa does not drink alcohol.

Muwanga we Kaligwa was a great healer within the Kingdom of Buganda and a healer to the King. He was very popular and stayed in the mountains. People who needed his services looked for him in the mountains. Some people claim that Muwanga we Kaligwa is the nature Muwanga.

All Baganda clans have access to Muwanga whose working details depend on the clan and its responsibilities. A few clans access Muwanga we Kaligwa, while the rest access the spirit of Muwanga we Nseke.

Muwanga we Nseke

Muwanga gave had a child he named Muwanga and worked with him during his spiritual services. Both the father and the son were called by name of Muwanga, did the same work at stayed at the same place Kaligwa. In the beginning, Muwanga the son, worked with and learned from his father. Muwanga the son worked like his father and offered healthcare services. However, when he became an adult, he started drinking alcohol, become so much of a drunkard and a disgrace to his father who distanced himself. Muwanga the son, left his father's place at Kaligwa, settled at a place called Nseke, worked like his father and become known as Muwanga we Nseke,

Muwanga we Nseke drinks alcohol.

Ndawula

Spirit Ndawunal tormented my brother by making his nose rot with foul-smell and non-responsive the western medicine and herbs.

Spirit Ndawula, who is a Mulangira as is Kawumpuli, was born with diseases and suffering from jiggers, kinyindwa and skin disease. Spirit Ndawula is a Kabaka after he has harmonized.

The shrine of bakabaka is called Lubiri. Ndawula has *endeku* from which can be used by everybody to drink from.

Kawumpuli

Who is Kawumpuli?

Kawumpuli is Lubaale and Kawumpuli was born and therefore a mulangira. Kawumpuli is referred to with various names such as; *kubbo lya mulimba likala ku lusse lwenju*.

Kawumpuli from the beginning was called a Lubaale spirit before his origin was well known but when his origin was revealed we came to know that he is a mulangira who was born.

Kawumpuli is a son of Kabaka Kiyemba and his mother is Nakku of Fumbe Clan. When the mother Nakku realised that the child was disabled, she abandoned the child in the banana plantation when he, Kawumpuli, was picked and raised by Nabuzaana. Nabuzaale physically transferred Kawumpuli with her to Bunyoro, but spiritually Kawumpuli stayed in Buganda. That is how Nabuzaana become the mother for the whole nation and Kawumpuli was given the honour of Katikiro (Prime Minister).

The spirits of Buganda and Bunyoro united in spirit, which explains why Baganda spirituality has Bunyoro spirits like Kaliisa, Nabuzaana,

Kawumpuli was not born as a twin, but what made him prominent and unique was that he was born a crippler and was born by a King and a princess. It is said that Kawumpuli normally possesses the princesses. Kawumpuli is a Mulangira and a Musambwa spirit

Since spirit Kawumpuli also falls in the category of Misambwa that is why they do not sacrifice in his presence because animals are not sacrificed to spill blood for the Misambwa spirits

Kawumpuli is given a Lujumba cock (*Lujumba omumyufu ne bidugavu)*

Spirit Kawumpuli is given a male black goat having a beard, the goat is shown to him but is not sacrificed in the spirit’s presence, the way it is done for Lubaale Mukasa, Musoke and Kiwanuka The beard from the goat is well prepared and is usually put on when spirit Kawumpuli is carrying out healthcare services.

Spirit Kawumpuli is given a male black cock

Lubaale we nyanja Mukasa, Musoke, Kiwanuka

Lubaale Mukasa, Musoke and Kiwanuka are sacrificed for in the morning at day break. time 5.00 a.m is associated with Lubaale spirits

The Lubaale spirits is associated with seven (7) times and its rituals and ceremonies are associated with seven (7) times while the Mayembe and Misambwa are associated with nine (9) times.

When the traditional healthcare spiritualists are giving medicine they tell their clients to take or use it for 3, 7, 9 days depending on the associated spirits. Spirit Muwanga also is associated with 9.

Lubaale Mukasa is the father of Lubaale Kiwanuka and Lubaale Musoke.

Lubaale Mukasa and Kiwanuka are harmonized on the same day at the same time at 5.00 a.m in the morning. Lubaale Musoke is harmonized the next day at the same time 5.00 am after Lubaale Mukasa and Lubaale Kiwanuka are harmonized.

Lubaale Mukasa, Musoke and Kiwanuka take fresh banana juice (*mubisi*).

The skin hides from the sacrificed animals are tied around the person possessed by the spirits with one from male and the other from the female animals which is called *kikyusankanamu*

Mukasa

Lubaale Mukasa expresses its arrival by signs of sailing in a boat on water using a boat and enkasi – *Lubaale Mukasa ajja aseyeeya kumazi nga akuba enkasi.* Upon its arrival, immediately Mukasa greets by saying Sese Davu Sese Davu, Gayira Sese gayira.

Spirits are able to explain all the details about themselves. When Mukasa expresses its presence during Lubaale exploration, it is asked what it is demanding from the family. Mukasa is asked a series of questions regarding its characteristics, its regalia and rituals so as to prove itself to the people present. Mukasa must explain where it comes from. (Mukasa is expected to say he has come from Sese and arrived through Bukasa landing site). Mukasa is also asked what is taboo to him from within the lake, (expected answer is Nkejje)

Mukasa is a son of Kadduwanema and marries a wife Nagaddya from a place called Nkumba.

Lubaale Mukasa is given a white male goat and a male white cock. The while animal skin from its sacrificed animal is used as *enkanamu* to tie around the waist of its spirit medium and the feathers from the white male chicken are used to make a *fumu lya* Mukasa (Photo)

Lubaale Mukasa also has *effumu, eryato and enkasi*.

Kiwanuka

Lubaale Kiwanuka is given a male brown or white sheep. When Lubaale Kiwanuka demands for a white sheep, he would have resembled his father (*aba yefanaganyiriza taata we*). Lubaale Kiwanuka is also given a spear (*effumu) and embugo*

Kiwanuka spirit is the only spirit that can cool down the ruth of Bamweyana – for example, if Bamweyana is so annoyed and furious and intended to do any harm, it is Kiwanuka that can keep him down.

Musoke

Lubaale Musoke is given a female goat and yet he is male because when all the spirits are sacrificed for male animals the clan will not reproduce. The reason why Lubaale Musoke is harmonized with a female goat even when he has possessed a male person is to bring reproduction (*oluzaalo*) in the clan. Lubaale Musoke is given a spear (effumu).

Kiwanuka and Musoke assist othe spirits that offer healthcare services.

Ddungu

Spirit Ddungu is a musambwa belonging to the wild *(Ddungu Musambwa gwa kuttale*). Ddungu is harmonized by a male goat (*ngayabitanga*) and those rituals are done in the wilderness (kuttale). The Musambwa Ddungu is the one who determines which type of *bitanga* goat he wants. There are three types of bitanga; white, brown and black. Those symbols represent the functions of the spirit and those symbols are endowed with spiritual powers *(amanyi)* which they use in healthcare services.

Muzimu

Nkongojja omuzimu gwa Zikwa – I am a medium for Muzimu spirit of Zikwa

Zikwa is a male spirit of my great grandfather – yet some people say a male Muzimu cannot possess a woman. I am a woman possessed by a male Muzimu.

A male Muzimu can possess a female as its medium depending on the characteristics it is looking for within that clan.

Omuzimu gwe gunanyini Lubaale, era omuzimu gwegunanyini Lubaale w'agwo. Omuzimu gweguyitibwa okutangaaza n'okutambuza Lubaale w'agwo. Omuzimu is the owner of Lubaale and more knowledgable about other ancestral spirits under its control. Muzimu is the one called upon to highlight about its other ancestral spirits and grant permission for the continuation of its harmonization process.

Omuzimu gunanyini Lubaale gwegwo agwasoka okukola Lubaale wagwo, n'omuzimu ogwasembayo okukirizibwa okutebenkeza lubaale oyo. The Muzimu spirit that owns the Lubaale is that which first made that Lubaale or the last Muzimu to have been authorised, and harmonize it.

Omuzimu ogumu tegusamirw bantu babiri - There could be more than one person in the clan who are possessed by spirits. However, the same Muzimu cannot possess two people at the same time.

When the Muzimu is called upon, it comes and possesses a person, it says whether it had other ancestral spirits under its control. It is requested to mention their names and related details so as to corroborate the previous information

The omuzimu tells the clan members the Lubaale it owned and even when the Lubaale spirits come, they confirm the Muzimu which owned it.

It is also a must that to carry out spiritual rituals without the presence of spirit Muzimu is quackery (*Era tteeka bwoba osamiza Lubaale nga tekuli Muzimu oba obba bubbi banno*). Because the Lubaale you are carrying out rituals for has no origin, then how do you carry out those rituals?

When you are making rituals for someone as a Senkulu, the Muzimu spirit is the one which narrates what it had in the past, after he has told you his names and where he was buried. The other categories of spirits come to just confirm. Sometimes the other spirits do come and tell who their master is and assist to call their master tell the details.

The Muzimu spirit is the owner of all the other spirits. During Kisamiza, the Senkulu cannot any spiritual rituals to harmonize other spirits before the Muzimu comes to confirm its spirits. The Muzimu is also asked, *Bwewali osamira Lubaale onno, emabega yava kwani?)*

When the Muzimu spirit comes, it is asked "did you have spirits? "if He answers that I had, the Senkulu will ask for the details of the spirits.

For instance, If I die my Muzimu will be Natembo, if it possessed any one of its choices, they ask the spirit that who are you? The Muzimu will answer that "I am Natembo" Did you have spirits? "I did and my spirit Natembo will narrate all the spirits it had. But what is the origin of the spirits which possessed you. In that case the family members confirm that these spirits really belong to the family.

Muzimu is the main spirit that owns the Lubaale and its other spirits. Harmonization of all the other spirits cannot take place without the presence of the Muzimu *nanyini* Lubaale. The Muzimu will possess a family member who will carry out the functions the owner of the Muzimu used to do while alive. If the Muzimu was a person who was a traditional healthcare spiritualist in his or her lifetime, then the person the Muzimu possess will carry on its functional role of a traditional healthcare spiritualist.

Traditionally, when the Muzimu choose its human medium, all other spirits of that Muzimu would only possess that individual spirit medium. However, other spirits in the clan would possess other members of the family but not those spirits owned by the identified Muzimu. *Omuzimu gwasamiranga empowo zaagwo zona*.

Female spirits, the wife spirits of Mayembe are harmonised with female animal sacrifice but without any chicken. Traditionally, women never used to each chicken.

Omuzimu gwelondera omuko ne Lubuga waagwo. It is advisable for the Muzimu to select its Muko and Lubuga from members of its one family, otherwise Lubuga and Muko can turn out to be dangerous to the spirit medium especially when jealous develops

What is a Muzimu?

Omuzimu gwe mwoyo nga guvuzze mumubiri gwomuntu. omuwoyo gusobola okwawukana nomubiri nga ofudde, webase, oba nebwoba nga otunula. Omwoyo bwegwawukana nomubiri, omubiri tegubanamanyi Omuzimu is the human soul out of its physical body. The soul may

leave its human physical when on dies, is sleeping or even when one is seeing. When the soul separates with the physical body, the body is effortless.

Omuzimu bweba gusumikira nga guyimiriddde. baguwangira ikibo kya balongo, ekya Lubaale, ensawo, omuggo, e fumu nendeku. Oluvanyuma omuzimu nomukongozi batuzibwa ku mwaliiro – The beginning rituals for Muzimu are done while standing, include empowering its basket for twins, the basket for its Lubaale, its bag made of a backcloth, its walking stick, a spear and a guade. At the end the Muzimu spirit and its medium are made to seat at a plain backcloth sheet

Mayembe

Amayembe gezimu ku obusobozi n'obuyinza bajjajjafe byebaleta okukola emirimu n'ensimbi, okukuuma ezzadde n'ensinbi – Mayembe are some of the powers and abilities brought to the clan by our ancestors for work and making money, to protect both the family and the money.

Mayembe getaaga okuddabiriza n'okuzibwa obujja buli luvanyuma lw'ebanga egere. Mayembe require timely rituals to rejuvenate their powers to best perform their respective roles

Amayembe geyogerela ebyago nenkola – Mayembe spirits themselves regarding what they need, why and how.

Amayembe gapapa nyo – Mayembe do things too much in a hurry

The demands by Mayembe during harmonization, are determined by what they were given by their ancestral owners and the way they were spiritually empowered in the beginning (*empanga ya Mayembe muntandikwa*).

Mayembe spirits were brought, as medicine, to the clan by our ancestors.

Some Mayembe spirits were bought from traditional healthcare spiritualists, to assist the wives of our ancestors to have children especially for those who could not produce children.

Annually, animals or chicken are sacrificed for Mayembe to rejuvenate the functional powers of the Mayembe. (*amayembe ganywesebwa buli mwaka)*

Every Mayembe spirit will narrate how it was brought into the clan and the purpose for which it was brought and the rituals and ceremonies which were performed in the beginning as it was being bought.

The Mayembe spirits will also confirm the Muzimu it belonged to, the Muzimu that brought it.

What is demanded by the Mayembe spirit is said by the Mayembe spirits themselves and the Muzimu which owned those Mayembe spirits

Jembe Lubowa

Jembe Lubowa is also referred to as Lubowa Lwakabondo. Most people say that Jembe Nalubowa is the wife of Jembe Lubowa but in my case Jembe Lubowa'a wife is Jembe Nakawunde.

I worked on someone's spirit Mayembe and they (spirits) instructed me that their housing is *ensumbi*.

Jembe Lubowa is the head of all Mayembe spirits. A multi-coloured dominantly black male goat (*bitanga omuddugavu*), a male Lujumba chicken, are sacrificed for Lubowa Lwakabondo, and is housed it a Buffalo horn of the right side.

When harmonising and empowering the male jembe spirit, its wife spirit must be called in and they are harmonised together. If the jembe is empowered without its wife spirit, it is a problem to you the individual, the healer and the community. The colour of the male animal used for the male spirit; the female wife spirit uses the same colour but female in nature.

The housing for the female wife spirit can be stated by either the husband or the female spirit itself.

Jembe Lubowa collaborate with many other Mayembe, Misambwa and Mizimu spirits.

A multi-coloured dominantly black female goat (enkazi ye bitanga ebiddugavu) is sacrificed for the wife of Lubowa.

Namuzinda

The wife of Jembe Namuzinda is Nakavuma and a female multicoloured dominantly black goat is sacrificed for its harmonization.

Kasajja Lwazi

The wife of Jembe Kasajja Lwazi is Nalubowa and a female black goat is sacrificed for its harmonization.

Kilarile

The wife of Jembe Kilarire is Nanziri and a female goat with a coloured belly is sacrificed for its harmonization.

Balongo / Twin forces

Nkongojja empewo ya Lubanga, nolwekyo nko emikoro jabaloongo – I am a medium for Lubanga spirit, so I have the ability to carry out rituals for twin spirits and twin forces.

Bearing twin children is a gift from ancestors and majorly Deity Mukasa. Twins are associated with the word Bweza.

If there are twin sets in the clan, rituals are performed for them *okumala abalongo.* Traditionally the ritualized twin sets used to stay with their paternal grandparents. Currently the situation has changes for fear of witchcraft, so parents stay with their ritualized twin sets.

When rituals for the spirits are done properly well (*abalongo nga bakolebwa nebatelera bulungi)* they are a source of family pride, there is peace and money in the family and clan. Rituals for the humanly twin spirits may include sacrifice of chicken or animal.

"abalongo bamirundi ebire, abazaale n'abatonde" There are two types of twin sets. The humanly twins are named Waswa, Babirye or Nakato and Kato.

Abalongo abazaale

"abalongo abazaale tebasamirwa" The humanly twin spirits do not possess people.

Abalongo abatonde (Natural twin forces)

"abalongo abatonde balinya kumutwe nebasamirwa" The non-human twins can possess people. such twin spirits include *Magobwe,* the python (Timba) and Leopard (*engo)* twin spirits

Some natural twine spirits are expressed in rocks and mountains, and referred to as Misambwa (*abalongo abatonde. Abalongo abatonde bassa bubaka* – Natural twin spirits do not talk but communicate through messages mainly through dreams. Such massages can be given to anybody

The rituals for natural twin spirits and no sacrifice is involved.

Abalongo abatonde tebayiwa musaayi, era mubifo ebyenkizo munono nga kunsozi tebatera kusalirayo bisolo. The natural twin-spirits are not associated with blood of any nature and it is common practice not to sacrifice animals at sacred places such as mountains

"Abalongo abatonde bagabulibwa kijjulo okuli amatoke agempogola n'obutiko obubaala" - The rituals for natural twin-spirits involve offerings in communal meal that involve *amatoke agempogola n'obutiko obubaala*

Twins can be produced when one is human and the other is non-human such as an animal or water, they are also twins (balongo). The only thing is to know who was borne first. The first is Waswa (male) and the second Kato; Babirye (female) and the second Nakato (female).

I also carry out rituals for the Balongo spirits at the client's residence. If client is in a single room (*muzigo*), we may do the rituals at the grandparents' home. If they have conflicts at their home and he comes here, he builds a hut to act as his *kasolya*

Healthcare management

Lubaale ajjanjaba mungeri zanjawulo - Ancestral healthcare spirits carry out healthcare services in various ways.

Misambwa giwa bubaka, bubonero, kola mikolo nabijjulo - Misambwa spirits give messages, symbols and rituals and communal meals

The rituals and ceremonies for the *Misambwa emizaale* and those of *Misambwa emitonde* are different.

Lubaale spirits work in shifts. When spirit Muwanga works today, the next week it be spirit Kawumpuli, the other week Muzimu might be the one working. Muzimu also performs healthcare practices. Another week it might Jembe spirit working. The Mayembe spirits can perform diagnostic functions, they can do it even without touching the *mweso*. The spirit Jembe just tells you what the origin of your problematic situation is. The spirit Misambwas is not so common in people. In most cases Misambwa bring messages but it is not common for Misambwa to possess someone and carry out diagnosis. You never know there could be such incidences but on my side I find it difficult for that to happen

Causes of illness and disease

Spiritual causes

One of my two maternal aunties (*Senga*) working at Mulago national referral Hospital developed illness of the hands to the extent that she could not handle anything. The many consultations done revealed that ancestral spirits were demanding to be harmonised and appeased.

Witchcraft

Olumbe lusobola okusindikibwa abemyoyo emibi nga bayita mu ddogo - Olumbe can be caused by bad hearted individual through witchcraft.

He visited a healer and he was given medicine. He bewitched me and suffered from “etalo” which was about to kill me. That night I was dying of pain. My trainer thought that was due to the spirits Mayembe which had not yet had their rituals done.

Witchcraft can be done while on a fire-place. It is worth to note that some fire-places cannot be used for witchcraft

Health assessment (diagnosis)

okumanya olumbe nekyaluleta kyetagisa omweso – To assess or make a diagnosis of *an illness and know its cause* requires use of traditional diagnostic tools.

Olumbe may be associated with hearing of voices with instructions to the affected person to which the affected person may or may not respond.

omulalu angamba nti agoberela amaloboozi byegamugamba – The madman informs me that he is responding to the instructions given to him by the voices he is hearing

People come to me, a traditional healthcare spiritualist when they have problems in their families. On consultations with the spirits, the spirits inform the families that some of their problems are caused by or associated with the family Lubaale. By the time the family comes to me for consultations, they already have independently made consultations and are generally aware and had been informed that Lubaale was the cause of the problems. If they had come as

individuals, I advise them to get together as a family *baaze Lubaale waabwe* to explore their Lubaale for the Lubaale to come and disclose to them what needs to be done.

When the family members are ready, they come as a family and we start on the process of *okwaaza Lubaale*. Okwaaza Lubaale is the process calling upon the Lubaale spirits by rituals of drumming and singings, when the family members are properly prepared together. If all goes well, the various Lubaale spirits will come, possess some members of the family and start talking to the family members in presence of the experienced traditional healthcare spiritualists (*Senkulu)*. The spirits are interrogated by the lead *Senkulu* and other family members, to narrate their origin, how they to the clan/family, what they demand of the family and how to harmonize then properly. The spirits are given ample time three to nine (3-9) days to talk and are repeatedly asked to confirm who they are and what they need. The talking spirits are requested to call upon their other spirits to also come and narrate their stories and demands for harmonization.

When the *kwaza* process has been successful, the family members are given time to look for what the spirits have demanded and when the family is ready, the family will come to the same Senkulu or choose another Senkulu to lead and guide the harmonization process of their family Lubaale.

Treatment and healthcare

Sacrifices and offerings are made during healing rituals

Sacrifice is made to target specific illness

I hold the animal or bird sacrifice on the area of illness and ask the illness and its cause to leave the body and enter the sacrifice. I then slaughter or burn the sacrifice. The meat of the sacrifice will be eaten, but not by the client, or burnt to ashes if the illness was deadly.

Rituals

For rituals involving fire, I often use incense and supplication to chess the spirits responsible for illness to leave the human body and go will the illness through the offered animal or bird. *"Nteka akabaane kumuliro ne namiriza nga nsidikiriza olumbe n'empewo ayaluleta okuva mumubiri"*

Rituals and ceremonies are involved healthcare services. The spirits might cause *olumbe n'obulwadde* and these (spirits) are harmonized through rituals and ceremonies, the *olumbe and obulwadde* will be healed.

The ailments brought about by the demanding spirits are treated by harmonization of those spirits through rituals and ceremonies. The spirits also have meetings/conferences in which they decide what to do in order to bring about their harmonization

Prayers

Originally prayer was not part of our culture of healthcare practice but with the invasion of the western culture, prayer also became part of our culture.

"*Ffe tetusaba, okusinga tulamiriza bulamiriza*" – Ours is not prayer but supplication"

In Bulubaale, prayer could be equated to communication to the spirits asking for what one wants or thanking for what one has been given by the spirits.

It is believed that during communal prayers the spirits meet and listen to the people in a congregation (*olukiko*).

There are times when the spirits set days for communal prayers, on which days the spirits listen and attend to the people/community problems. The community also makes preparations in order to be ready to communicate with the spirits. So in those prayers every particular person asks for what he/she wants.

Treatment of Olumbe

okujanjaba olumbe kisobola okwetagisa saddaka yenkoko enkoko ya lujumba, embuzi, endiga oba ente okusinziira ku lumbe nejelwava - The treatment of an illness may require sacrifice of chicken of specific colours, a goat, sheep, or cow depending on the type and cause of the illness.

okujanjaba olumbe ntela kukozesa binyoni n'ebisolo – I use birds and animals in management of illnesses

olumbe lujjanjabibwa mikolo, biwebwaayo n'okusaddaka – management of olumbe is done through rituals associated with offerings and sacrifice.

Nkozesa ebimira ebilimu amanyi agenjawulo mukujjanjaba lumbe - I use plants imbued with known spiritual powers in management of illnesses

Olumbe lutabula obwongo nelusuuka eddalu elitasobola elitategerekata wadde okujanajabibwa naddagala lya kizungu – Olumbe may reach the brain and cause madness, whose origin or cause cannot be established nor treated by biomedical methods and processes.

When my aunt consulted, she was told them that she was sick because she had wronged the spirits. The healer gave them herbs, with instructions that when discharged from hospital, they go straight to the shrine and make a communal meal for the spirits.

I have another brother who was cured of madness by my Lubaale spirits. Every August month of every year he used to get mad. One time we were in the shrine (Kiggwa) and they told us my brother had gotten mad again, spirit Muwanga possessed me and told family members to go and bring my mad brother. When he was brought, they told them to bring a spear, shield and a backcloth (*efume, engabo n'olubugo*). Spirit Muwanga said that they should be given to him, he has a name that he denounced. The name he had denounced was Muyini. He instead

called himself Erifazi Sebowa. After giving him those things and renaming him Muyini the madness got cured. He had taken a long time falling mad.

That meant that they were appeasing the owner of the name (Muyini) by giving him "*ekifundikwa*". That is why the names given to people or names of the ancestors have a reason. Others advise one to remove a name from somebody because that name might have a bad history. Sometimes people come to spirit Muwanga with problems whose origins are from their names. The spirit would advise that person to be renamed and the current name dropped.

The spirits may just use one person to get to the actual person they are going to use to perform their duties. They may use another person because of financial capability, they may go through, illness, or poverty and many other ways. In my case the spirits never tormented me but they did it through my brother and possessed me instead

Communal meals and ceremonies

Each of the ingredients and constituents of the communal meal have their significance and the way they are performed.

Treatment of obulwadde

bulwadde are easily treated and cured. For example fever.

Herbs or use of plants in their raw or processed forms may be used to treat and cure *Obulwadde*.

Prevention and protection

Okuganga

okuganga is for both prevention and protections

Okuganga is a form of power used to prevent problems coming your way.

Omubiri gwe gugangibwa - it is the physical body that is protected

prenvention "*okuganga, kiziyiza"*

Muwambo

Muwambo is a protective substance put at home to prevent and protect against bad things from reaching the home. *Muwambo* prevents, protects and treats the family against sickness, poverty, bad omen, witchcraft,

Health promotion

Words and phrases that describe health

Obulamu obulingi has extended meaning for an adult person. This when whatever you do is successful; you have a good job, you harmonized your ancestral spirits and spirits, you have enough money, you have a stable family, have children, have good friends and many others without any disturbances. Otherwise, if you have not harmonized your ancestral spirits and they are still demanding, you will also be on the demanding side of your health. You will have no money, yearn for what to eat and life will not be good for you and your family.

Obulamu obulungi kwekubako wottudde nga ofunye ne sente zo. Health is when you have settled life with your own money.

Bweza is being grateful for what is good, and gratitude is extended to the ancestral spirits. When someone gifs you anything good, you say Bweza as an expression of gratitude – thank you.

Words and phrases that describe illness

Lumbe

Olumbe: is when the health condition is not clear and its cause in not known. It has failed to be identified in the western health facilities.

Olumbe or obulwadde are named or defined by the root cause. Such as ancestral spirits, witchcraft, punishment,

"*Olumbe lwambala omuntu era lujjibwaawo namikolo omuli okwanbulula"- Olumbe* only possesses the person and can be removed through rituals such as ritualistic baths.

Kisirani

Ekisirani lumbe. Ekisirani can be treated easily by use of plants or herbs in ritual baths. "Ekisirani kikwata mubiri" Kisirani is on the outer surface of the physical body. A traditional healthcare spiritualist can easily identify a person with kisirani.

A person with kisirani is not paid when he works, does not associate well with his workmates, or bosses. Everybody or everything seem to work against him or her all the time.

Kulamiriza

Use of repetitive words that go with actions in the healing process is *kulamiriza*.

Kulamiriza is used to address illness (lumbe), either to get rid of olumbe or to cause it. The words aare known to the spirits, I call upon the spirits and pray to them to affect my intention and prayer, good or bad. I repeatedly use the words known the specific spirits for their actions

Kulamiriza works most towards Mayembe, when they are being given instructions of what to do

Words can be used for healing depending upon the natural powers of both the spirit medium and the spirits involved. What the spirit medium speaks out, the spirits will put implement

The spirits like Muwanga, Mukasa, Kiwanuka and many other spirits use the power of words they say to heal people. This mainly happen because these spirits work with many other spirits that implement their words.

Biteega / Kiteega

ebiteega mpewo za Barundi – Biteega are spiritual forces of Barundi people of Burundi

Nyabingi / Nabingi

Nabingi mpewo zaba Nyarwanda - *Nyabingi* are spiritual forces of Banyarwanda people of Rwanda

Bidandi mpewo za ba Tanzania - Bidandi are spiritual forces of people of Tanzania

Words and phrases that describe disease

Obulwadde, nga omubiri tegweyagala - *Obulwadde* is when the physical body is weak, not feeling well.

Obulwadde bukwata omubiri – *Bulwadde* affects the physical body

"Obulwadde bukwata omutwee nebutabula obwongo." Obulwadde may attach the brain and cause madness.

Okujanjaba obulwadde tebulimu nyo kulamiriza – treatment of disease does not involve of use of repetitive healing words

Symbols and symbolism

Every spirit has its own symbol.

The symbols of Muzimu *are engabo and effumu,*

Kawumpuli has a symbol of a spear.

Ddungu has symbols of *olulutula, ekitimba, endeku, engombe, ebide* which represent hunting and those are what spirit Ddungu uses when hunting for whatever humans want.

Ekita are containers of alcohol for the spirits.

The *emuli* are symbols that belong to spirit Muwanga which Muwanga uses in healthcare services.

Coffee beans (*Katamukago*) a symbol of unity

Matembe (Black seeds with a hole through) a symbol associated with good things towards individual and family including life, riches, being loved and favoured, saying words liked by others and leadership roles.

Cowrie shells *(Ensimbi)* is a symbol for money rooted in the family from ancestors, through generations yet to come. For all of them to be rich.

Lweza *(Aerva lanata) ne* Bombo *(Momordica foetida)* are plants when put together and knotted into a ring (mugge) are referred to as *Lujjula* (photo) that symbolise elements of good luck to the family in regards to riches, good and successful life.

Kakumirizi (*Sida cuneifolia*) is a symbol uniting family member in peace

Mukasa (*Senecio discfolius*) is a plant that symbolise (Bweeza) good luck and good things towards the whole family

Namirembe (*Agerutum conyzoides*) is a symbolic plant for peace and prosperity

Mavigamukulu (*Leucas martinicensis*) is a symbolised plant that when prayed upon, the prayers are answered

Kayayaana (*Vernonia sp*) is a plant with symbolism of favours, to be favoured and preferred by all people of all categories and classes

Muwanga (plant) is a symbol for authority, power and abilities

Lusika (plant) is used to symbolize the ability to attract whatever is good from far and near towards oneself and to all family members.

Kafugankande (*Microglossa angolensis*) is a symbol for the king of shrubs used for healing,

Mulamula (*Dracaena fragrans*) is a plant associated with authoritative powers and as a basis for making a judgement (*ddamula*)

Lumanyo lyakati oba omuddo ( ) are plants symbolised for ability to know and understand (*Okumanya n'okutegeela)*.

Nabugira ( ) is a plant that symbolise being attractive in terms of what comes out of the individual and character

Lumuli lwenkalira (reed that dried while still at its base) symbolise ancestral spirits that did well the protective and security roles to protect the individual, family and grandchildren

Money in form of coins were placed in the twin basket (*ebibo bya balongo*) to symbolise the need for money.

Symbol of nine (9) relates to rituals that involve Muzimu, Misambwa, Mayembe and Lubaale

Symbol of seven (7) relate to rituals involving twins and twin forces.

Ensawo yo Muzimu (*Kyaafa-kilinaki*) is a bag for the Muzimu and its Lubaale made of backcloth and empowered by the spirit Muwanga or Kawumpuli through a ritual referred to as *Okuwanga ensawo y'Omuzimu ne Lubaale we*. This bag is meant to protect money that comes in from being wasted

Sex in spirituality

In the tradition of Busamize, the *Senkulu* is considered the spiritual father of the *batende* and it is a taboo for the Senkulu to have sexual relations with his or her *Mutende*

Taboo

Taboos are associated with reasons why the taboo was put in place. So it is always important to understand the taboo and its original context so as to understand it contemporary relevance.
