# Supplementary material for: Perspectives on health, illness, disease and management approaches among Baganda traditional spiritual healers in Central Uganda
Source: PLOS Glob Public Health. 2024 Sep 6;4(9):e0002453. doi: 10.1371/journal.pgph.0002453 (PMC11379289; doi:10.1371/journal.pgph.0002453)
Supplement: S7 Data — (PDF) [file pgph.0002453.s007.pdf]

Study participant 6 transcription

Contents

Study participant 6 transcription 1
Socio-demographics 3
Muluntansozi and Mulubaale 3
Baluntansozi 3
Balubaale 4
Problems associated with Becoming a Mulubaale. 4
What is Lubaale? 4
Becoming a Mulubaale 4
Kwaza Lubaale 5
Okusamira Lubaale 5
Okutendeka Lubaale 5
Senkulu (Trainer) 6
Royal spirits 6
Sources and Access to healthcare information 6
Sources of healthcare information 6
Access to healthcare information 6
Causes of illness and disease 7
Spiritual causes 7
Health Management 7
Health assessment (Diagnosis) 7
Diagnostic tools (Mweso) 8
Treatment and health management methods 8
Kusawula 8
Kwambulula 8
Kyogero 8
Kyogo 9
Kuganga 9
Kusandaga 9
Kijjulo (Communal Meal) 9
Communal Prayers 9
Prevention, and protection 10
Health promotion 10

Sacrifice 10
Offerings 10
Words that describe health, illness and disease 10
Words that describe health 10
Bweza 10
Words that describe illness 11
Words that describe disease 11
God (Katonda) 11
Ancestral Spirits (Lubaale) 11
Muzimu 12
Misambwa 13
Misambwa emitonde (Natural spirits) 13
Misambwa emitonde - Characteristics, roles and functions 13
Misambwa emizaale Ancestral spirits 13
Muwanga 13
Bamweyana 14
Kawumpuli 14
Ddungu 15
Lubaale 15
Mukasa 15
Kiwanuka 16
Musoke 16
Mayembe 16
Role and function of Mayembe 17
Lubowa 17
Abalongo (Twin spirits) 17
Mulongo w'enkoba 17
Balongo abatonde 18
Abalongo abazaale 18
Okumala abalongo 18
Twin forces in healing (*Abalongo mukujjanjaba*) 18
The royal spirits (Abalangira n'Abambejja) 18
Spirit Mediums 19
Nature and Natural places 19
Shrine 20
Symbols and symbolism 20

Regalia 20
Colours 20
Black 20
Animals and birds 20
Plants and other natural materials 20
Witchcraft (Eddogo) 21

Socio-demographics

My name is (name withdrawn), a Muganda, male of Nvuma clan. I am 50 years and I practice traditional beliefs. I stopped in Primary Seven (P.7). I am a traditional healthcare spiritualist with 27 years of experience and practice subsistence farming. I am married.

Nvuma clan is a very special, and the chosen clan of God

My home area is (x) village, in (xxx) sub-county, Mpigi district in Mawokota County (Saza). My shrine is Lubiri with roots to Sekabaka Kiyemba the 6th King of Buganda whose Masiro are at Sentema in Busiro County (Saza). This shrine is of Lubiri with different departments.

I belong to Uganda N'eddagala Ly'ayo traditional healers association. This was the first traditional healers association started by our ancestors. Their objective to prepare, strengthen and promote the spiritualist because of the disruptions against our traditional spirituality (lubaale wa ffee) by the colonialists

Muluntansozi and Mulubaale

I am both a Muluntansozi, and a Mulubaale

I am a trainer. I train other healthcare spiritualists.

A trainer in Bulubaale is Senkulu

A trainer in Buluntansozi is Kabona

Baluntansozi

Bwenali nga ntendekebwa obuluntansozi munsozi, Lubaale yagibwa kumutwe, emisambwa emitonde mukifananyi kyabamtu, negitusomesa okutegela nga abantu,okukozesa ebimera mukujjanjaba. kiyamba okumanya ekyokukola nebwobera nga tetulinyidwako lubaale. When I was in the mountains during training as a Muluntansozi, my Lubaale was off my head and the natural spirits started training us as human spiritualists in the use of medicinal plants. this helps knowing what to do even without intervention of ancestral spirits.

"Mubutonzi tekikola mulamwa empewo okulinya kumutwe, wabula okutambulira kumalobozi agakutegeza ebimera n'enkosesa yaabyo mukujanjaba abalwade". For Balutansozi, it is not usual nor necessary to be possessed by spirits for the spirits to work, but instead respond to guiding voices which tell you the plants to use and how to use them to manage the patient.

"Obutonzi buyonjo nyo" Nature is very clean and smart.

"Obutonzi bwegendereza munkozesa ya mabala gabwo". Nature is very particular in its symbolic use of colours.

Spirits are associated with specific colours and at my shrine spirits are assigned to be on duty on specific working days. The spirit on duty is identifiable by the dressing code of the day. For example, when Lubaale Kiwanuka is on duty, the dressing code of the spirit medium is red colour.

Balubaale

Obukugu bwo Mulubaale mukujanjaba businzira mu Lubaale we. The professional abilities of a Mulubaale are rooted in his/her ancestral powers

A good traditional healthcare spiritualist has and utilizes both the natural and ancestral spiritual powers. "*Omulubaale omulungi alina era akozesa amanyi gobutonzi n'obuzaale"*

When a Mulubaale restricts himself to using ancestral spirits only, he is not capable to address all healthcare issues because some health issues may require natural spiritual powers.

Problems associated with Becoming a Mulubaale.

Lubaale yansula eddalu n'antwala mu kibira okumala emyaka esatu. The ancestral spirits took me to a forest where I stayed for three years as a mad person. But during that secluded time, I was tough many things by the Misambwa.

Omuzimu gwa Ssekabaka Mutesa II, taata wa Kabaka Muwenda Mutebi II ali kuntebe kati, gwansumbuwa nyo, onkunsobozesa okukola emikolo Kabaka Mutebe asobole oluwayira - I used to be disturbed by the Royal spirit of Sir Edward Muteesa, the father of the current King of Buganda Ronald Muwenda Mutebi II. This royal spirit wanted me to perform some rituals at Ssese Island to enable his some, king Mutebe to get a wife.

What is Lubaale?

The open space between the earth and the sky is what is referred to as *Lubaale*

Obulubaale get its meaning from the explanations and interpretations.

Becoming a Mulubaale

Twakerelanga mukwebuza na kunyonyolwa birooto – The first activity every morning was consultations and dream interpretations.

empewo ezakyankala olwokulogebwa, ezakugibwa, ezasibwa obuteyoleka n'okulungamya abazukulu zitelezebwa. The ancestral spirits that were bewitched, restricted or in captivity and unable to express themselves nor guide their clients were normalised.

Kwaza Lubaale

Kwaza Lubaale – is exploration of ancestral spirits –

Okwaza is a cultural diagnostic process involving family and clan members conducted by a knowledgeable traditional healthcare spiritualist. In the process, the clan deities are called upon to express themselves and their requirement from the family

Kwaza process takes an initial period of nine (9) days

Okusamira Lubaale

Okusamira Lubaale kijuliza emyoyo emingi egiriyo mu Lubaale. Okusamira Lubaale sets reference to the many souls and spirits in the open space. Thus, in aspects of spirituality, the phrase "*tugenze kusamira lubaale"* refers to the process of seeking the manifestation of some of the many spirits in the open space. These spirits presentation may be haphazard and disorderly.

Okusamira are words of secrecy, revealed while in the shrine, meant to be kept to one self, and to be reflected upon and researched

It is also thought that Kusamira came from the saying that *ebigambo byenkugamba bya**nsa** byetaga ku**mira*** meaning that the words used during the Kusamira process were very meaningful and needed to be taken with top secrecy. Similary Nsamizi is the place where the Misambwa spirits used to stay, as reflected in the song "*akama ke misambwa kali mu gwaffu emanga, akama mukanonyereze x 3"*

During okusamira Lubaale, it is the Muzimu which is called upon first.

Okutendeka Lubaale

emizimu gitekateka ginaajo – meaning superior ancestral spirits are responsible for preparing the newly expressed spirits,

Okutendeka tutekateka omukongozi n'empowo mubujanjabe obwenono. *Okutendeka* is to prepare the spirit medium and the spirits in health management based on ancestral knowledge and roots.

Okutendeka kikolebwa abamanyi nga balina obuyinza, n'empewo emanyirivu okusingawo – Okutendeka is performed by knowledgeable individuals with authority and the experienced and superior spirits.

Newankubadde okusamira lubaale kitegeza kunonya n'okusikiriza egimu kumyoyo egiri mu Lubaale, okutendeka lubaale kitegeza okukwanaganya n'okusengeka eninanyiganya ne neyoleka yemyoyo egiri mu Lubaale nga gitemba kumutwe. Much as okusamira Lubaale means seeking and persuading some of the spirits in the open space, Okutendeka Lubaale refers to the

orderly and sequential arrangement and presentations the attracted spirits may follow as they take possession of the human spirit medium, especially depending on their functional roles.

Okutendeka empewo n'omukongozi mubujanjabi obwenono kikulu era kyetagisa nyo – training of the ancestral spirits and their medium in healing based one's cultural roots is important and very necessary. Ancestral spirits and their medium can be misguided if not well trained

Senkulu (Trainer)

Most people whose time for training has reached, come to me when they are complaining of some bad health conditions and on investigations, majorly through their own dreams or through omweso, the indications are that it is time for the person to start the training to become a healthcare spiritualist.

Royal spirits

I am also a *Mumbowa*, by birth, with roots in the royal Palace *of Sekabaka Kiyemba* the sixth (6th) of Buganda whose royal Tombs (*Masiro)* are in Sentema Busiro.

I am the Kabona of Jumba in Nakifuma where the current king goes for power. In that case I am not a mumbowa of current Kabaka, since he comes to knee for power from Nakifuma where I am a Kabona.

Sources and Access to healthcare information

Sources of healthcare information

Obubaka buva wa Tonda, Katonda The healthcare information is directly from God, Tonda.

olusi ebyokukola mbifuna kuza ku Muzimu At times I get instructions of the healthcare information from Muzimu.

Omuzimu gufuna *amanyi n'obuyinza* kuva wa Tonda (Katonda). Muzimu gets its powers and authorities from God.

Lubaale ateledde alungambizibwa Tonda (Katonda) - Well harmonised spirits get healthcare information directly from God.

Access to healthcare information

nafunanga obumanyi n'okusomesebwa okuyitira mu birooto n'okulabanga ebifananyi byabantu nga bansomesa ebyokukola, naddala ebimera bye ddagala, nenkozesa yaabyo mukujjanjaba abantu. - Whenever I would be going for any form of training, I would get messages through dreams and visions of ancestral people what to do and how to do it, especially how and when to use plants for managing clients.

I was also trained by both the spirits and the people about counselling clients.

The spirits have a committee from which they choose a messenger who can deliver a message to the human being. The Muzimu can also deliver information, can be messengers.

nfuna okwolesebwa n'amaloboozi I get healthcare information through visions and voices. The spirits can bring a picture of a tree which I am going to use for a particular treatment and the voices tell me how to use it.

Causes of illness and disease

Spirits cause problems, illness and disease to inform and force their ways into stubborn and negligent individuals and families. Spirits can be determined and persistently forceful towards stubborn people.

Spiritual causes

Mukasa, Musoke and Kiwanuka are furious spirits when offended. Spirit Mukasa can cause shivering.

Health Management

"Empewo zitera okundaga omulwadde gwezigenda olundetera nga tanajja, era nezinungamya bwentekwa okumukolera nga atuuse. Omuladde oyo bwajja nkola nga bwenalungamizibwa." I normally get spiritual information regarding the patients to come and what I am supposed to do for them. When the clients physically come, I do as I was guided in the dream. - I believe that the ancestral spirits of the client request my ancestral spirits to manage the client.

Health assessment (Diagnosis)

omulwadde nga anajja, njolesebwa obuzibubwe oba obulwadde bwe olusi nekyokumukolera – When a client is about to come to me, I get him/her in visions showing me his/her problems or ailments and at times I get to know what to do, while still in the vision. (give an example) ……

ntela okuwulira amaloboozi nga gambulira ebikwaata kumuntu ntudde mumaaso, amaloboozi gangambwa ebyokumubuuza era negandabula wa nembye. - I often hear voices telling me about the client in front of me, and telling me what to ask him/her and alerts me when I am lied to. (give an example)

ebeseera ebisinga empewo zininya nezizuula ebizibu byabazukulu baazo, - often times, the ancestral spirits possess me and do the assessment of their clients (give an example)

Munsonga ezimu, omuzukulu muwa eddagale obubaka bukkire kuye. Obubaka bwabufuna mubirooto, tubuvvunula netobujamu amakulu olusi nekyetutekwa okukola. – In some particular cases, I give the client some medicine to facilitate his/her dreaming so as to get the information. When the information is got in the dreams, it is brought for interpretation and meaning making and at times the dreams also give out indications towards the solution. (give an example)

The spirit possessing me may use its *mweso* (diagnostic tool) to make a diagnosis and the root causes of the problems.

abantu banebuzako okulungamizibwa n'okwetereza - people consult me for guidance and finding solutions

Nsoka kuba mweso nga sinalungamywa muntu anebuzizaako – I first consult the ancestral spirits through the use of the omweso before I do any offer any advice.

Okulagula is a traditional cultural process by a traditional healthcare practitioner to foretell and advise, based on retrospective and prospective foresight

Diagnostic tools (Mweso)

There are different types of mweso. Each spirit has its own type of mweso and how it is used. The mweso may consist of dust, stones, coffee berries, endegge and other constituents, engatto etc

A spiritualist who carries out accurate diagnosis must have spirits of Muwanga or Kawumpuli.

Treatment and health management methods

Whatever ailment that attacks the spirit/mental manifests in the physical body because the spirit is within the physical body.

The spirits decide when and which ritual to carry out depending on the ailment or situation. "*Buli lusozi n'engo yalwo*".

olusirika bwe bujanjabi obusinga – self isolation is complete silence while Sleeping, dreaming, and fantasizing is considered as one of the best ways to achieve a healing

The patient should be put in a good mood in order to get treatment.

Kusawula

Okusawula is a cultural treatment process practiced by a traditional healthcare spiritualist.

Kwambulula

Okwambulula is a ritualistic cleansing process of that normally involves use of materials such as plants, birds and animals with spiritual powers and abilities

Kwanmbulula is the same as *kyogero* in children and in adults *kyogo* it is renewing someone (*okuza obujja omuntu*).

Kyogero

Kyogero is used in children to clean, prevent, immunize, treat and protect children from *olumbe no bulwadde*

Most common plants included in kyogero include;

- **barks of** Kirikiti, Mugavu, Mwoloola, Muzanganda, Kifabakazi, Kajjolyenjovu, Ntaseesa (leaves)
- **Shrub roots and/or leaves of** Kigonge, Kafugankande, ekitooke kya Nakitembe (roots), Mulamula (roots), Mutulika leaves, Kamunye,
- **Grasses of** Lweeza, Bombo, Mubiri, Kawulira, Kavamagombe, Kayayaana, Mavigamukulu, Namirembe, Mukasa, Muko wewumbeko (for female children *okufuna kunsonyi*)

Kyogo

Kyogo is used in adults to cleanse, prevent, immunize, treat and protect adults from *olumbe no bulwadde*

Kuganga

Okuganga is measure to protect the body, mind, and spirit from problems that could be sent in form of witchcraft, or accidentally coming one's way. *Okuganga* is a protective measure done on the physical body, through *kusandaga* (scarification), or *kuyingiza* eddagala (pressing the very fine powders of the medicines through the soft areas of the dry skin.

Okuganga is advised and performed by a traditional healthcare spiritualist following consultation regarding continuous problems experienced by a person or family

Kusandaga

Okusandaga is when scarification is done on the body and medicine is made to enter the body which might not necessarily be the case in *Kuganga*

Kijjulo (Communal Meal)

In *butonzi* God calls the people to come for communal prayers through dreams and other direct means (*bubaka*) while *in buzaale* the Muzimu mobilises the people in its own ways to come for a communal meal *ekijjulo.*

A communal meal (ekijjulo) is meant to confirm a ritual "*kukatiriza ensonga ziba zikoleddwa".*

Communal Prayers

I believe in prayer. Prayer is *kusaba and kwegayirira*. Prayer involves asking for forgiveness in whatever you have wronged God knowingly or unknowingly, consciously or unconsciously.

During prayer I acknowledge the blessing of nature and the spiritual entities created by Katonda (God).

Prayer also involves asking for your needs. God sometimes appears to me especially when I am going for a journey. (*Viira emandwa etule nga kyeyakulagula kitukiridde*). The spirit

may tell you something and leave a sign. For instance, a spirit may tell you that to prove that I am the one, I (the spirit) is the one who has brought rain when I go the rain will also stop.

I have to pray to Katonda because he is Katonda. I humble myself while praying.

"Nebaza Katonda kulwamanyi n'obuyinza bye yawangira mubutonde nebitonde by'amu"

"Katonda nkwebaza okumanya n'okutegera byewampa okusobola okukozesa amanyi n'obuyinza ebyobutonde okutebenkeza obulamu bwaffe"

"Nzikiririza nti si nze nzijanjaba naye obujjanjabi buyitira mu ba jjajja"

Prevention, and protection

Some spirits can offer protection of an individual or family in supernatural ways. Such spirits may make scarification on the body while the client is sleeping at night only to realise the scarification marks on waking up in the morning.

Omuwambo is a form of protection for a place and its content. For example *Omuwambo* can be placed in a compound to offer protection of the home, the property and the people at home.

Health promotion

Sacrifice

Abalubaale tusaddaka nsolo na binyonyi so si bantu. - Us traditional healthcare spiritualists sacrifice animals and birds but not human beings.

During healing rituals, I and the client hold the sacrifice make supplication – *"tukwata ku saddaka netulamiriza"* and I command "leave this body and take the pains with you into the sacrificed" *Vva mumubiri ogende mu saddaka".*

I tell the client to request the illness to leave his/her body and go into and enjoy the sacrifice

Offerings

Fruits are given as offerings

Words that describe health, illness and disease

Words that describe health

Bweza

Obweza refers to *Mirembe* which applies to the physical body, financial, shelter and food.

Words that describe illness

Words that describe disease

Obulwadde kyekintu ekikosa omubiri gwo - *Obulwadde* is anything which antagonizes your body.

Human body can be antagonized by headache, pain, thirst, wounds, or any symptoms that are used to make a diagnosis

Bulwadde affect any part of the body, mental or spiritual

God (Katonda)

Within the natural settings, Mukasa becomes God.

When God created the earth, God created Wanga, whose counterpart, in the ancestral lineage, is Muwanga. God also created a human being named Kintu.

Then God asked that is that person who I have given you breathing (asaa) then he answered that yes he is breathing from which originated Mukasa implying that muka-air, asa – breathing- Mukasa. God is the one who provides the air for breathing. In that case Mukasa becomes God

Tonda says that "I am everything".

Wanga is God who comes as spirits like Muwanga, Lubowa and others. Muwanga is Lubaale who is son of God whom he gave kingship. So Muwanga is a king. All children of God are kings. Musoke is the first bone of God.

Ancestral Spirits (Lubaale)

Lubaale yena ali mu bbanga – All ancestral spirits are in the open space. Lubaale includes *Mizimu, Mayembe, Misambwa*

Empewo zitumibwa manya ga mirimu gyezijje okukola. The spirits get their names from the functional role they have come to perform. Many years ago, African spirituality was invaded by Europeans to the extent that its practice was prohibited and illegal. So, the spirits stopped to mention their actual names and instead simulated the name of the function the spirits have come to perform. For example, Spirit Muwanga come to *Kuwanga*. Spirit Lubowa came to *Kubowa* (hijack). *Omutonzi* prepared the spirits and their respective functional roles. So, these names we use to refer or call the spirits are not their actual names but names of their functional roles.

Empewo zirungamizibwa. The spirits can head to advise

Some spirits came directly from God, such as Lubaale Musoke, Lubaale Kiwanuka

The major spirits from God are seven (7) and are represented in the rainbow. They are

The major ones are the seven which we call rainbow: Wanuka, Nabawanuka, Wanga, Bulamu, Mambya kusalawo, Musoke-Kiwanuka- Mukasa. These spirits are usually found in caves on mountains and believed to be from God.

I cannot know the spirit, for it is the spirit that tells us its names. In the actual sense the spirit tells its name relating to the work it has come to do instead of telling you its actual names. For example, "*nze Muwanga nzize kuwanga*". " *nze Lubowa nzize kubowa"*

Muzimu

Omuzimu gwe mwoyo ogwayo eyaliko omuntu mubwe nyama - Muzimu is a spirit of once a human being with a physical body.

Muzimu is an ancestral spirit which was originally a human being.

Omuzimu gwa Kintu gwe omuzimu omukulu mu Baganda – The *Muzimu* of Kintu is the major spirit among Baganda. Kintu was a child of Katonda (God)

Buli kika kirina omuzimu omukulu ogukulira emizimu emirara jona mu kika - Each clan has an original head, who is responsible for all Mizimu in the clan

Omuzimu is responsible for the ancestral spirits it owns while still as a human being while still living on earth

Muzimu is a spirit, it can possess a person and talk for itself.

Muzimu gives the details of what it possessed and other explanations in that regard

Buli Muzimu gulina ekifo gyegusinziira - Mizimu have sacred places from where they originated.

Muzimu is the only spirit which says its true name, a name not related to its function.

Omuzimu gwegunanyi mpewo zona zenkozesa wano – Muzimu owns all ancestral spirits I use here

Omuzimu belongs to the clan in one's ancestral lineage.

Muzimu is a spirit, invisible and is moving air.

When a person dies, his/her soul does not die, it becomes a *Muzimu*.

*Omuzim*u is in the clan, family and it is a spirit.

The *Muzimu* goes to the mountains to pray to God. The message from *Muzimu* is taken to the spirits in the caves which directly communicate with God. Therefore, theses spirits are on *emyaliiro esatu; omwaliiro ogwo obuzaale, ogwawakati, ogwawagulu.*

Omuzimu is the owner of Lubaale, Mayembe and Misambwa.

Omuzimu assigns duty to other spirits such as Lubaale, Mayembe and Misambwa.

The omuzimu is there to plead on behalf of the human being

Misambwa

Misambwa are classified according to nature of formation, and function.

Misambwa can be created naturally (mitonde) or given birth to (mizaale).

Misambwa are male or female

Misambwa are soldier spirits

Misambwa emitonde (Natural spirits)

In some sacred natural places, natural spirits (emisambwa emitonde) may that appear in physical image of a person and lead the trainings.

Omusambwa can provide a communal meal (ekijjulo). Omusambwa is a kabaka. When the jembe fails the Misambwa can come into play. Omusambwa is chief over the jembe.

Misambwa emitonde - Characteristics, roles and functions

Ancestral spirits are symbolised by putting on backcloth (*olubugo).* The Lubugo is never washed, does not get dirty. Traditional healthcare spiritualist put on the backcloth to be different from others

Misambwa emizaale Ancestral spirits

Muwanga

Nkongojja Muwanga – I am a medium for Muwaga spirit. *Muwanga mpewo* - Muwanga is a spirit.

Nzijjanjabisa Muwanga – I use Muwanga spirit for healing in my daily activities.

Are soldier spirits

Muwanga is Lubaale

Muwanga is the one who is harmonized last after all other spirits have been harmonized. Muwanga is Lubaale and at the same time is kabaka. Muwanga is the one who imbues power into other spirits - *Muwanga yawanga empowo endala zonna*. Muwanga is superior over Muzimu. Omusambwa is superior over the Muzimu but the Muzimu is the one which arranges for the harmonization of other spirits. Muzimu is respected by all other spirits including Muwanga. The Muzimu is not more powerful than other spirits but it is a big pillar for all the

spirits. Omuzimu found the Misambwa on earth. Mayembe are soldiers (protectors) of Lubaale. All the spirits are connected, coordinated and work hand in hand

Muwanga is the owner of the mweso. Every spirit has its own especially. After diagnosis by Muwanga or Kawumpuli, the Muzimu decides who is to carry out the treatment depending on the type of ailment.

"Amanyi ga Muwanga gali mu kigambo" The power of Muwanga is in his words" What Muwanga wills to happen and puts on his words will happen, because his words are powerful, authoritative and are actualised in reality.

Muwanga may use plants and animals or their parts in healthcare by calling upon the powers and abilities enshrined in them to contribute to healing.

Bamweyana

Bamweyana is a spirit and the person it possesses behaves as mad.

Bamweyana spirit uses crude alcohol (*walagi) and* marijuana (*enjaga).*

Bamweyana spirit is considered a judge of Tonda.

Kawumpuli

Kawumpuli is believed to cause all epidemics and pandemics.

Kawumpuli, when annoyed, brings about epidemics, pandemics and other strong diseases.

Kawumpuli was borne a crippler child (*kiwumpuli, kintuntu*), but with a lot of powers. Kawumpili is the greatest *mulaguzi*. Kawumpuli is confident and very sure of the diagnosis he makes and proudly says "if you fail with me, the only last trial is with Muwanga, with the most sure diagnostic tools *omweso gwe ngatto*

Kawumpuli is great in treatment of ailments.

Kawumpuli is very good at diagnosis and treatment.

Kawumpuli atereza empewo implying that Kawumpuli helps in harmonization of other spirits.

Kawumpuli is the cause of most ailments and is involved in treatment of most ailments.

Nalunga is the mother of Kawumpuli and she did not want to see her child crippler – Kawumpuli.

Kawumpuli is also a Sebakabaka.

Ddungu

Originally Ddungu does not use dogs to hunt. It was the Kayizzi and Kanoonya that used dogs to hunt while hunting with Ddungu that is how Ddungu become associated with dogs during hunting

Ddungu is accurate on target

The actual Ddungu is tested by being given a spear to spear an animal at a long distance. In case he fails to hit the target, it is likely that He is not true Ddungu

Ddungu main role or purpose is to hunt for Money, Good luck, Blessings, Clients etc.

Lubaale

Lubaale is the mixture of spirits, "*Lubaale mugoteko gwa mpewo"*.

"Abalubaale balungamizibwa empewo nebavumbula beddagala" -Traditional healthcare spiritualists are guided into discovery of medicines by spirits

The spirits know the plants with medicinal values to use. Such plants when used by the guidance of the spirits and they are effective. Human beings take the plants in laboratories for the chemical analysis for their active ingredients.

Mukasa

The Mukasa for whom I am a medium introduces himself as Mukasa son of Semusulo Kadduwanema

Mukasa is a diviner (*mulaguzi*) imbued with supernatural powers as compared to other spirits. The respondent pointed at a white goat and said "This white goat belongs to Lubaale Mukasa and Lubaale Mukasa demanded for it".

Lubaale we Nyanja (Musoke, Mukasa, and Kiwanuka are associated with peace (*Merembe and Bweeza). Lubaale Mukasa mugabi we zadde* - Lubaale Mukasa is the spirit responsible for reproduction

This associated with a song; *"Mukasa akera, akera mu nkoko, Mukasa akera, akera munkoko embereberye, mukasa akera nagaba obweza, mugabi w'obuma, mugabi wezadde".*

I witnessed a client who came and presented his case to the spirits. Lubaale Mukasa asked him to bring a small wooden boat and *enkasi* (*akaato n'enkasi*), and pick water from a Lake Victoria (Nalubaale). When the client brought them the following day, Spirit Mukasa told him to wash the small boat with the lake water, then pour some lake water into the small boat, place it near the fire-place for twins (*ekyoto ky'abaana*) and use some of the remaining lake water to bathe himself during day time for the next three consecutive days while staying at the center. The spirit went on to explain, "The purpose is to use superior powers of the lake to clean you and remove anything unwanted that was placed on you and set free all your ancestral spirits that have been in bondage". After the 3 days, the lake water still remaining in the small boat was poured at the middle point of the shrine of the water spirits (*esabo lya Balunyanja*).

Later when I asked why the small boat water placed near the fire-place for twins (*ekyoto ky'abaana*), Mukasa spirit possessing its medium explained that the children (twins and twin forces needed be protected from being used to make mistakes, because the mistakes made by the twins would be counted on the client. The spirit gave a detailed outline for the twins, twins forces and the children, and concluded by saying "*nze Salongo omukulu w'abaana bona n'abalongo, nze nta era nze ngaba obweeza*" - "I am the spiritual father of all children, twins and twin forces, I am the one who makes people free from bondage and giver of blessings".

Kiwanuka

Lubaale Kiwanuka demands for a brown male adult sheep.

Lubaale Kiwanuka is associated with lightening (*Laddu)*

Lightning strikes and darkens the area it strikes (*egullu-laddu bwerikuba lidugazawo*) that is why, at times Spirit Kiwanuka may demand for a brown sheep with a black patch at its belly below.

Lubaale Kiwanuka is a soldier. Wanuka is the soldier spirit that protects God. Wanuka spirit is the counterpart of the ancestral Lubaale Kiwanuka. Ancestral Lubaale Kiwanuka uses fire while Wanuka uses the power of the Sun or lightening.

Musoke

Lubaale Musoke is also a soldier but acts as a lawyer (muwolereza).

Lubaale Musoke is worse than any other spirits when he gets angry. When Lubaale Musoke gets angry, he is able to easily sucks blood and water out of the person's body making him/her very anaemic and dehydrated.

Mayembe

Nkozesa amayembe mukujjanjaba – I use Mayembe in my healthcare practice

Amayembe ganwa omusaayi – Mayembe use blood

Amayembe gasaddakirwa – Sacrifices are done for and by Mayembe

Amayembe ngasaddakira nkoko, njiibwa, mbuzi, ndiga, oba nte – I sacrifice chicken, peageon, goat, sheep or cow for the Mayembe

Amayembe ngasaddakira nga nzijjanjaba olumbe oluzibu enyo, oluva ku ddogo - I sacrifice when I am managing illness due to witchcraft.

Mayembe a mazaale ganywesebwa omusaayi nga gawangibwa. - Ancestral Mayembe spirits are given animal blood when being harmonized.

Mayembe, Misambwa and Balongo are *basirikale* (soldier spirits).

Mayembe a mazaale are ancestral spirits.

Amayembe amatonde tegakozesa musaayi gwa nsolo yonna, ensolo yago bata nte mukifo ky'ago. Amayembe amatonde (Natural Mayembe) are not associated with animal blood, instead the animals given to the Natural Mayembe is offered and released free at the natural place related with that Jembe. Such an animal is neither sacrificed nor killed but only let free

Role and function of Mayembe

Amayembe gambulula – Mayembe can perform ritualistic cleansing. *Nkozesa amayembe mukwambulula abalwadde* – I use Mayembe in the cleansing rituals

Mayembe basirikale era jje dwanyi meaning Mayembe are soldier spirits

Lubowa

Lubowa Jembe – Lubowa is a Jembe spirit. *Nina era nkozesa e Jembe Lubowa* – I possess and use Jumbe Lubowa. At my shrine Jembe Lubowa does not have the right nor the mandate to start a gathering involving Muzimu and Misambwa spirit.

Lubowa *asiba* (can put people and other spirits in bondage)

Abalongo (Twin spirits)

Nina abalongo - I have twin spirits

Abalongo kye ki? What are abalongo?

A human being can give birth to twins where one twin is human while the second would be child is an animal, stone or a river. An example of a river twin is Waswa Mayanja that was delivered first then followed by a female human being called Nakato. The river twin has supernatural powers and naturally connected to its human twin. The river twin can transform into an animal like a leopard (engo) or transform into a python snake either of which visits its human twin.

Balongo manyi a matonde oba amazaale agatambulira mu bubiri. Balongo are natural or ancestral twin forces

Abalongo gemayembe agasooka mu kika, baserikale, bakuumi, balongoosa kinomu ne mukika. Balongo are the primary clan helper forces that provide protection, purification, rectification and help maintain stable individuals and family.

Abalongo bebasiba era bebasumulula, ebimanyiddwa n'ebitamanyiddwa. Twin forces are traditionally known to be responsible for causing problems and also the lead in resolving any problems and any health issues. So, they are always called upon first in any issue to undo whatever was done by known or unknown forces.

Mulongo w'enkoba

Buli kabaka alina omulongo ayitibwa 'omulongo wenkoba'. Each king, natural or humanly, has a twin set referred to as "*omulongo". Balongo babakabaka abazaale bawundibwa nga basirikale be*. The twin forces for any humanly king are decorated and act as the king soldiers.

There is no ruling Kabaka without a twin set (*mulongo)*, who's main Jembe protect and provide the King with messages about the future.

Balongo abatonde

Some natural places, especially mountains, have twin features and are referred to have natural twins "abalongo abatonde". For example, Buswa Bulongo mountain.

Abalongo abazaale

Abalongo abazaale use animals and reptiles as their mediums and their twin names follow the human twin names relating to their order of twin. The first twin to come out is Waswa for male or Babirye for female and the second that follows is Kato if male and Nakato if female. So, there can be Waswa and Nakato or Mayanja male or Namayanja, female.

Okumala abalongo

Abalongo bamalibwa era bawundibwa. Rituals to harmonise twin forces are referred to as "*okumala abalongo"* and to decorate the twin sets is "*Kuwunda Balongo"*.

Twin forces in healing (*Abalongo mukujjanjaba*)

Abalongo bakozesebwa batya mukujanjaba? How are twin-forces used in healing process?

All Baganda clans have twin-forces (*Balongo*) used in healing although, not necessarily, their main healing spirits. In some families, *Balongo* (twin-forces) are the main healing spirits while in other families, *Balongo* are complimentary spirits in the healing process.

The royal spirits (Abalangira n'Abambejja)

Nkongojja Basekabaka, abalangira n'Abambejja – I am a medium for royal spirits of former Buganda Kings and the male and female royals.

Most Kings were soldiers and their spirits behave as soldiers

Basekabaka balamuzi – Kingly spirits pass judgements

Sekabaka Kiggala is the only prince (Mulangira) who become a king two times. Kiggala was a Prince (Mulangira) who become a King the first time. However, King Kiggala had sexual relations with his princess sister (Mumbejja) and they produced a child. This child was considered a **twin prince** since he was produced by King and a Princess. Kabaka Kiggala was forced to give his throne to his son. However, when his son got problems, Kiggala was enthroned as a King for the second time.

The royal kingship is categorised into ancestral and natural. *Obwakabaka mulimu obuzaale n'obutonde.*

Bakabaka abazaale bazimba embiri zaabwe kunsozi basobole okubaka amanyi The ancestral Kabaka build their palaces at top of mountains in order to be able to tap power.

This helps the bakabaka to get power from the creator (*Asobole okubaka obubaka okuva ewomutonzi).* The bakabaka gets visions (abonekerwa).

The King is called Kabaka because the Kabaka receives messages from God and disseminates these messages within ones kingdom (*Yabaka ebiva ewa katonda n'abisasanya.*).

The mountain on which the *Kabaka* builds his palace (*Lubiri*) has natural spirits which spirits own the mountain and is the power of God. Such spirits derive their powers from God.

The natural royal spirits, *bakabaka abatonde*, are believed to be children of God, who descended on earth to help the ancestral royal spirits.

The power from above is the one which rules the power from earth. (*obuyinza obwawagulu bwebufuga obwa'wansi*).

Spirit Mediums

Some spirits have animals, such as goats, which they use as their mediums.

Spirits such as Ddungu and Kayizzi, are characterised by hunting and are associated with animals such as dogs. That is why traditional healthcare spiritualist usually have dogs within the environment of their workplaces, the shrines.

Spirits are associated with animals of different colours that they depend for.

Nature and Natural places

Ebifo ebimu byatumibwa amanya okusinzira kubyali bikolebwa mukifo ekyo – some places derived their names depending on the activities taking place. For example, the place Nsamizi on Entebbe Road, derived its name from the activity of *Kusamira* that used to take place in that place. Even today *tusamira.*

Nature (obutonzi) sits on a human being

Each spirit has two types, the ancestral and the natural parts. (*buli mpewo eli mubuzale nemubutonzi)*

Some natural places are associated with specific spirits.

Rivers are associated with Balongo and Misambwa.

Mountains are associated with Basekabaka, Misambwa, and Balangira spirits

The Misambwa can own the mountain, but that mountain has other spirits as well which spirits are sent by the main Misambwa for different errands.

I believe that these natural places are sacred with supernatural, mystical spiritual powers.

Shrine

"*Sseddugge ye mpagi ekuuma e Ssabo okubera ekittikiro*" meaning that Sseddugge is the pole at the centre of the shrine that keeps it at the top of which is put e kittikiro. At that place, normally put are the symbolic regalia in a seemingly disorganised way that include, endeku yo mwemge, milk, omwenge, omubizi gwenjuki (honey), walagi (high content spirits). This caused disharmony within the spirits. The spirits that are not comfortable with alcohol will be offended by those spirits carelessly using alcohol.

Symbols and symbolism

Spirits are associated with their own symbols such as spears, shields etc.

The symbols have particular significance and meanings depending on the spirit which is demanding for it.

I change the symbols, like the smoking pipe and dressing depending on the spirit which is possessing me.

Regalia

Different spirits have specific requirements and their regalia should all not be placed in one place to avoid some spirits offending others by mixing up what others do not want or drink. Some spirits that do not drink alcohol will be offended by the presence of alcohol near their regalia

Colours

This male black goat is for the Mayembe.

Spirit Bulamu demanded for a black male adult goat.

Black

To dress with back cloth has meaning

Animals and birds

Such animals demanded by the spirits are reproduced at the spiritualists place yet others are bought as demanded by the spirits.

Animals are imbued with spiritual powers

Plants and other natural materials

Different trees have different spiritual powers in their natural settings. "*Emiti gilina amanyi agenjawulo mubutonde bwago*"

This plant tree *(omukokowe*) belongs to Lubaake Kiwanuka. I did not plant this mukokowe myself, I found it in the place. This *Mukokowe* tree has a lot of powers associated with Lubaale Kiwanuka - *obuyinza bwa kiwanuka bukkira mu Mukokowe*

The *mukokowe* tree and its wood is not used for cooking food and it is used in Kiwanuka's fireplace only (*Ekyooto kya kiwanuka*). Spirit Kiwanuka may demand for its fire place.

When a mukokowe tree is used as fire wood can lead for lightening to strike people or animals - *Laddu ayiza okukuba abantu oba ebisolo*.

Within ancestral spirituality, trees are planted and such planted trees can be imbued with spiritual powers. *"Emiti egisimbibwa mubujjajja jewangibwa negibera namanyi aganjawulo"*

Omuwafu is a tree plant with special spiritual functions. The *Muwafu* tree is normally planted fruit tree associated with ancestral grave-yard.

Akabaane (the *Muwafu* sap), when smoked, is used as incense to communicate with the spirits and *Katonda* (God). *The Kabaane* is used by Christians, Muslims and Traditional spiritualists possibly for similar issues.

Keeping the natural environment intact and in organic form promotes health.

Encroachment and destruction of the environment is a source of many diseases

Witchcraft (Eddogo)

Ddogo is kutataganya.

Ddogo (witchcraft) can be done to a person to negatively affect one's finances, business, family, and inability to produce children, marriage, even to affect the way one thinks.

Empewo zilogwa. The spirits can be bewitched.

A person may be a witch if his/her ancestors were witches.

People with problems related to witchcraft come and consult with me a traditional healthcare spiritualist to look for a solution.
