# Supplementary material for: Perspectives on health, illness, disease and management approaches among Baganda traditional spiritual healers in Central Uganda
Source: PLOS Glob Public Health. 2024 Sep 6;4(9):e0002453. doi: 10.1371/journal.pgph.0002453 (PMC11379289; doi:10.1371/journal.pgph.0002453)
Supplement: S8 Data — (PDF) [file pgph.0002453.s008.pdf]

Study participant 8 transcription

Contents

Study participant 8 transcription 1
Socio-demographics 2
Baluntansozi and Balubaale 3
Baluntansozi 3
Balubaale 3
Becoming a Mulubaale 3
Ssenkulu 4
Ssenkulu, characteristics and responsibilities 4
Ssenkulu selection or appointment 4
Mukongozzi (Spirit Medium) 4
Sources and access to healthcare information 4
Sources of healthcare information 4
Access to healthcare information 5
Words that describe disease 5
Ancestral spirits (Lubaale) 5
Lubaale we nyanja 6
Lubaale wo kulukalu 6
Lubaale alinya ku mutwe 6
Lubaale wa Sanduuke/Mugugu 7
Muzimu 7
Characteristics of Muzimu 7
Regalia for Muzimu 7
Misambwa 8
Characteristics of Misambwa 8
Muwanga 8
Kawumpuli 9
Mukasa 10
Musoke 10
Kiwanuka 10
Mayembe 10
Characteristics of Mayembe 10

Abalongo (Twin spirits and spiritual forces) 11
Health management 11
Causes of health, illness and disease 11
Spiritual causes 11
Cultural causes 11
Other causes 12
Diagnosis 12
Mweso 12
Treatment 12
Health improvement, prevention and protection 12
Rituals 12
Communal Prayers 14
Places for ancestral spirits and spiritual significance 14
Natural places 14
Shrines 14
Mbuga 14
Lubiri 14
Fire places 14
Incense (Kabaane) 15
Ekyooto 15
Emindi 15
Symbols and symbolism 15
Colours 16
White 16
Brown 16
Back 16
Yellow 16
Animals and birds 16
Plants and other artifacts 16
Walking sticks 16

Socio-demographics

I am Muwonge Hassan, a muslim, male, Muganda by tribe. I belong to Ngabi Clan. I am 38 years old. I studied and stopped in primary seven (P.7). I am married. I am a Mulubaale with 21 years of practical experience and a subsistence farmer. This is a *Kiggwa* for our Clan and is headed by Spirit Muwanga here at Kawuku village, Butenda Sub-county, Bukomansimbi

District, Buddu County (Saza Buddu). I belong to a traditional healers association called LUTHA (LUbowa Traditional Healers Association), which I head. I do not belong to any major traditional healers associations because of their many internal fights across the associations, which makes it difficult to understand their missions and goals

Baluntansozi and Balubaale

Baluntansozi

Baluntansozi do subscribe to natural forces which they access from natural places, mainly mountains. *Abaluntansozi tebakililiza mu Lubaale*. Baluntansozi believe in ancestral spirits.

Balubaale

I am a *Mulubaale*, and I get possessed by ancestral spirits. We may also be connected to ancestral places with natural powers, but such places have family or tribal ancestral connections.

Becoming a Mulubaale

Omuntu okusamira ela naawa Lubaale ekitiibwa kyetagisa Lubaale n'asoka namuluma era namubonyabonya – For a person to become a Mulubaale, have value for and respect Lubaale, it is pertinent for that person to first suffer and experience pains from Lubaale.

"Lubaale yambonyabonya nyo. Lubaale yandwaza nyo, yansula eddalu, yanemesa okusome, okukola, ate nga yankwata ndi muto" I suffered a lot due to Lubaale. Lubaale made me very ill, I become mad, neither attended school nor could I work, yet it possessed me at a tender young age.

nze nakulira bu kojja. My mother was a business woman who was always travelling in business related activities. When my mother conceived me, she remained unaware of her pregnancy until very late when she started labour pains and delivered me soon after. This caused much confusion at home. My father unaware of the pregnancy, refused to give me a name, and denied me as his child. It was my uncle, the elder brother of my father, who gave me a name because I resembled my father in all aspects. In my father's family, I was the only single child by my mother, so my mother's family raised me.

Lubaale yankwata nga ndi muto era nga yeyogerela. ekyo kyanvirako okubonabona enyo olwa Lubaale, nga abenyumba tebakiriza olwokuba ediini yo busilam esiside era nga emamidde awaka. I greatly suffered due to ancestral spirits (Lubaale) because the highly religious Muslims family members resisted strongly. Subsequently, Lubaale used a lot of force for the family to heed to the spiritual demands. My movements were restricted by tying me with ropes. The spirits made me very stubborn towards the whole community in Bukomansimbi and neighbouring districts of greater Masaka.

Lubaale alonda bulonzi omukongozi we mu b'ekika kye. Lubaale selects its medium within its particular family lineage. For me, I grew up with my mother who had separated with my father.

We went for Kusamira as a family, but I was the only one possessed by spirits with healthcare function

Lubaale wa kika era yanuma anonya ba kika kye – I was possessed by ancestral spirits and they tortured me while looking for members of its lineage.

Lubaale waffe yamala kuterezebwa n'akkalira abewaffe okufuna kumirembe. It was only after Lubaale was fully harmonised that peace prevailed at our family home.

Ssenkulu

Ssenkulu, characteristics and responsibilities

Ssenkulu omutuufu tasobola kutendekako mandwa gyebatamutendekako The Ssenkulu (main trainer) cannot train spirits he has no expertise of.

Ssenkulu wa Lubaale yaloperwa ebya Lubaale oyo era yamugambako singa wabaawo ebitatukiridde. It is the trainer (Ssenkulu) with the capacity to discipline Lubaale, address its undesired acts or mediate any conflict between Lubaale and its medium.

Ssenkulu selection or appointment

Obutende bwange, Lubaale yagaana ba Ssenkulu betwelowoleza. During the process of becoming a Mulubaale,my Lubaale refused and objected to the trainers we had proposed. I was taken to a Ssenkulu proposed and preferred by my elder brothers for their own personal interests, however, when the spirits possessed me, they were very furious to him and attempted to beat him up before he run out of his shrine for his life.

Ssenkulu okutendeka Lubaale kitone okuviira dala mubuzaale - Being an effective Ssenkulu is a natural gift given at birth.

Mukongozzi (Spirit Medium)

Okwagala empowo oba okuzeyagaliza tekyivirako mpewo kukulinya – Being friendly to spirits or wishing to be a spirit medium is not a basis for the spirits to select a person as their medium.

Sources and access to healthcare information

Obubaka means the spirits communicate to you in dreams, voices or visions, with you as compared to when the spirits possess you and talk for themselves.

Sources of healthcare information

Obubaka n'endagiriro byenfuna era nengobelera biva mu ba Jjajange – The information and instructions I get and follow are from my ancestors. The information relates to individuals, family, or community.

*Emandwa esa obubaka.*The spirits are the source of the information

Emandwa zeyogelera. The spirits talk for themselves

Obubaka come from the spirits Mizimu, Lubaale, Mayembe, Misambwa, Balongo, Balangira.

Abogerezeganya nange mumaloboozi baneyanjulira amanya gaabwe ne bwebampita – The entities whose voices talk to me introduced themselves to me clearly, mentioning their names and at times how they are related to me.

Access to healthcare information

Nfuna obubaka nga buyitira mumaloboozi alusi ndaba abantu abogera nange nga bambulira ekyokukola – I get information through hearing voices and at times seeing people talking to me and giving me instructions on what to do

nywa emindi kubanga bajjajange baginywanga era banjetaga njinywe naddale bwebaba nga bakuninya kumutwe oba nga ngenda kukola nabo. I smoke a pipe because my ancestors used to smoke it and they demand for their smoking pipe and require me to use it when they are possessing me, or when they are going to work with me.

Nywa emindi nga netegekera empowo okunkwata zogere. I smoke a pipe when preparing for the spirits that possess me. However, when the spirit wants to come it will come and possess me even if I am not prepared with the smoking pipe

Words that describe disease

Obulwadde ye muntu okufuna ekitali kitebenkevu mubwongo bwe oba omubiri gwe

Ancestral spirits (Lubaale)

Lubaale wa mitendera ebiri, Lubaale owokunyanja ne Lubaale owokulukalu. – Ancestral spirits *(Lubaale)* have two major categories, water related spirits and the dry-land spirits.

si *buli mpewo nti esobola okujjanjaba* – not all ancestral spirits can offer healthcare

Lubaale alondera gwezinakwata okusinzira ku bukojja bwe - Normally *ancestral spirits* choose whom to possess depending on the preferred good qualities from your mother side

Nasamira Lubaale omuganda agoberela Muwanga, nensamira ne Lubaale wa bachwezi nomunyoro agobelera Muleguza

Mu Lubaale tukumakuma n'okutebenkeza emwooyo – *Lubaale* is used to organis and harmonise souls and spirits

Sisobola kunyonyola nzijanjaba yange bwekola, nange ndabira awo. I cannot explain the treatment mechanisms, but I only experience. For example, a mad person is brought here at my shrine, while shouting and fighting and I touch his head with my *nkyinga*, he immediately cools, settles and normalises, he even requests for something to eat. I also wonder how it happens, but our healthcare practice is a practical service, it is not about explaining or theorising.

Buli mandwa/mpewo esobola okujjanjaba – all ancestral spirits can offer treatment

Lubaale ajjanjaba – ancestral spirits offer treatment

Lubaale akuuma n'okutebenkeza abantu ne'ebyaabwe, mubyenfuna, ebyobulamu, embeera ezabulijjo, nebyobufuzi - *Lubaale* has a role for health promotion, protection and harmonizing people's lives socially, economically and politically and their properties.

Ensi yona yetololera ku Lubaale - All activities of Baganda in the world revolves about Lubaale

“Buli Lubaale attendekebwa era abeela ne Senkulu”. Ancestral spirits undergo harmonization process and training done by an identified trainer (Senkulu).

Newankubadde Lubaale mukulu era mumanyi, naye buli Lubaale attendekebwa – Although ancestral spirits are old and knowledgeable, ancestral spirits must undergo harmonization and training.

Lubaale okusinga tatambula nyo naddagala, Ancestral spirits often don’t use herbs for healing

Lubaale asinga kozesa manyi na buyinza okujjanjaba – Lubaale often use spiritual powers and abilities for healing

When the ancestral spirits are well harmonised, the benefits should be materialised soon, unless there were mistakes in the process.

Lubaale use herbs to complement its powers and abilities for healing.

Plants have spiritual powers that I tap into for healing.

Ancestral spirits have general and specific functions

Lubaale we nyanja

Lubaale we Nyanja asinga okukozesebwa mukujjanjaba mulimu Kaddu-Wannema, Mukasa, Kiwanuka, Musoke - The water spirits used in healthcare include Kaddu-Wannema, Mukasa, Kiwanuka, Musoke

“obudde bwemikolo gya Lubaale w’enyajja jja nga emambya esala. Lubaale atendekebwa mumasala g’emanbya”. The rituals for water-related spirits are normally performed very early morning before day-break (dawn) beginning at around 5.00 am.

Lubaale atendekebwa nga emambya esala - ritualistic training for wetland spirits is done at day-break (dawn) beginning at around 5.00 am.

Lubaale we kika atumbula ebyobulamu, ezadde enfuna, enkolagana n’embera okutalira awamu – Clan ancestral spirits promotes health, reproduction, income, relationships and general social well-being.

Lubaale wo kulukalu

Lubaale wo kulukalu asinga okukozesebwa mukujjanjaba - Muwanga, Kawumpuli, Bamweyana, Ndawula, Lubowa - dryland ancestral spirits used for healthcare include Muwanga, Kawumpuli, Bamweyana, Ndawula, Lubowa

Lubaale alinya ku mutwe

Lubaale wa Balubaale alinya kumutwe ne ye yogelera – Ancestral spirits for Balubaale choose a human medium, whom they possess and talk for themselves through him/her.

Lubaale may skip a generation or two in case such generations are not competent to handle its detailed cultural norms. – in such a case the Lubaale and its content will be kept intact in its suitcase. In our case, my great-grandfather is the one who competently harmonised the clan Lubaale. upon his death, his children and grand children were in the Christian and Muslim religious wave, what saw most cultural artefacts burnt. However, my linage did not burn but

secretly kept our Lubaale intact until our it picked its medium within our generation and demanded to be harmonised.

My father new about the ancestral spirits, but did not want to get involved. So, he just kept its content intact in the wooden case so as not to conflict with his religious understanding. So, it was after his death that our Lubaale was activated.

Lubaale wa Sanduuke/Mugugu

Lubaale wa sanduuke/mugugu akola kutumbula bakika kye – This set of ancestral spirits work for the promotion of the members of its clan

Lubaale wa sanduke/mugugu assinzira mu Kiggwa okukolera abekika kye – These ancestral spirits are based in the clan shrine to promote its clan members

Mubanga eggere, abekika batuula okuddabiriza Lubaale we kiggwa – The clan members gather annually for communal rituals to rejuvenate their clan ancestral powers and abilities

Muzimu

Omuzimu ogwankwata ye Mukasa – I was possessed by a Muzimu by names of Mukasa

When the Muzimu appears, it tells its details to the members of the family and explains what it requires of the family members. It may require birds, goat, sheep or cow of specified sex and colours.

Characteristics of Muzimu

Emizimu jota kukyooto – Mizimu use fire for wamth

Ebyomuzimu sibyakukwatibwa bukusu, omuzimu gweyogerela ebyaagwo. Issues of Muzimu do not require be crammed because when the Muzimu comes it verbalises it requirements.

Mizimu vary in the ways they work depending on the ways and abilities of its human nature. Muzimu is the spirit left when a person dies. The Muzimu will retain the characteristics of its human nature. If the individual was a healer who used words to affect a healing, hie/her Muzimu will retain that characteristic so that when it medium says will be affected out.

Regalia for Muzimu

Muzimu also requires a *Kanzu eyomulera, ekifundikwa kyolubugo, effume, engabo eyenkundi limu*

Ebifundikwa byambazibwa ba mukago muddiiro omuzimu gwelisiimye naye nga sibakika kye – Bifundikwa are prepared and dressed over by those whose shrine the Muzimu has preferred, but not clansmen

Ebifunidikwa ebyennono eyenkuliiti biyabaribwamu mubisera ebigere, naddala mumboneka y'omwezi – The regalia of the original culture is dressed at periodical times especially at the beginning of the new moon

Ebifundikwa byeyambulwa oyimiridde – The regalia is undressed while standing

Ebifundikwa tebitwalibwa mu kabuyonjo – Do not go to the toilet while dressed in the regalia

Muggo - Muzimu may require a walking stick made from a specific plant possibly picked in a particular season by people of a specific clan.

Ancestral regalia should be safely and securely kept where it will not be discriminated upon.

Misambwa

Emisambwa egimu baali Bantu - some Misambwa were human beings

Emisambwa jitambulira mubintu bina (4). Emisambwa ejitambulira mubantu, Emisambwe ejewalula nga Timba, emisambwa egitambulira mubisolo, nga Engo. Emisambwa egitambulira mu mazzi nga Mayanja, Ssezzibwa. Some Misambwa are able to move and appear in four forms; Misambwa are able to move and appear like human beings. Some Misambwa are able to move and appear reptiles in a form of python. Some Misambwa are able to move and appear fierce animals like a Leopard. Some Misambwa are able to move and appear water bodies like rivers Mayanja and Sezzibwa

Emisambwa gyagala mbuzi, nte, nkoko n'amafumu - normally require goats, cow, chicken, spears

Emisambwa has specific colour preferences depending on the colour each Musambwa was given or associated with in the beginning.

Characteristics of Misambwa

Misambwa joota kukyooto – Misabwa use fire for warmth

Emisambwa emilangira , nga Kawumpuli, Bamweyana, Ndawula, tegisalirwa. No sacrifice is made for the royal spirits like Kawumpuli, Bamweyana and Ndawula,

Muwanga

Muwanga ye jjajja wa Lubaale – Muwanga is the grand-parent of all ancestral spirits (Lubaale). *Muwanga musambwa guli kukika* – Muwanga is a Musambwa at a clan level. Muwanga yakkira mukika kye Ngonge. Ngonge clan has more powers of Muwanga than other clans. Muwanga at a clan level is in two forms *Muwanga we Kaligwa* and *Muwange we Nseke. Muwanga we Kaligwa* does not drink alcohol, yet *Muwange we Nseke* is very alcoholic. However, both Muwanga work in the similar ways. *Muwange we Nseke* is appeased by gifts including alcohol.

I am possessed by Spirit Muwanga. Muwanga is the Spirit with most requirements among all Spirits. Spirit Muwanga asks for and is appeased by a gift of a brown male adult perfect cow, goat, chicken sacrificed for him. "*ente eta buli mulwadde Muwanga gwalijjanjaba okuwona"*- the sacrificed cow symbolise the authority given to Muwanga to health whoever client he will handle. In my shrine setup here, we annually sacrifice a brown male cow and brown male chicken to rejuvenate Muwanga's healing powers and authority. In case of lack of resources, cow meat roasted for Muwanga can suffice to appease Muwanga.

Muwanga mubutonde ya tondebwa era nayitibwa Kisinde – Muwanga in nature was created and named Kisinde. *Muwanga mubuzaale yazalibwa* – Muwanga in a human form was birthed by a woman and named Muwanga. Muwanga is soft spoken, in an old trailing voice hardly audible at the beginning. Muwanga is very knowledgeable of the working modalities of nature. Muwanga is very confident in his works and does not work in hurry, he takes his time. Muwanga uses Mweso to devine, *Muwanga alagula*. – Muwanga divines, *Muwanga atendeka*

– Muwanga trains. *Muwanga alina obuyinza obusumika n'okuteleza Lubaale ate ajjanjaba n'omubiri* – Muwanga has the spiritual authority to empower and harmonize other spirits and their spirit mediums. Muwanga can also treat diseases of biological origin using herbs. Muwanga is a herbalist. Muwanga is very knowledgeable in medicinal plants and uses herbs to treat illness, disease and solve other social and health related issues.

I estimate that currently 70 – 80% of the spirits that present themselves as Muwanga are not true Muwanga. Many people claim to possess and work with spirit Muwanga but fail to carry out the activities and duties of Muwanga. It all started when Mutesa 1 lost grip of his cultural values because of what came with the missionaries. He gave out most of his original Misambwa in excitement - *Mutesa 1 yalagajirila ebibye kulwebyo ebyali bizze*.

Muwanga is best known for detailed and most accurate diagnosis. With help of Mweso gw'engatto, Muwanga can be able to know all the details regarding Lubaale within the family and clan and also the problems and illnesses being caused by Lubaale. *Mweso* is also used to know the approach towards the needed solutions to the problems, illness and diseases caused by Lubaale. Traditionally Muwanga does not use Mweso gwe nsibi.

Kawumpuli

Ninyibwako omulangira Kawumpuli - I am possessed by Kawumpuli, a royal spirit in Buganda kingdom.

Kawumpuli is referred to by many names that include Selukeera,

Kawumpuli katikiro wa Lubaale owe Nyanja – Kawumpuli is the Prime Minister for all the water spirits.

Kawumpuli alagula, mulaguzi – Kawumpuli divines, is a diviner

Kawumpuli si Lubaale – Kawumpuli is not a Lubaale .

Kawumpuli atendeka Lubaale - Kawumpuli trains ancestral spirits

Kawumpuli ajjanjaba – Kawumpuli offers treatment

Kawumpuli katikiro wa Muwanga – Kawumpuli is the Prime Minister of Muwanga

Kawumpuli tasalirwa kisolo wadde ekinyonyi,- Kawumpuli does not require any sacrifice.

Kawumpuli alina omweso era omweso gwa Kawumpuli gusalirwa enkoko ya Lujumba nga mpanga. – Kawumpuli has his divining tool (mweso) which is sacrificed for a male chicken (Lujumba) (Photo).

Mweso gw'ensibi (Photo) is the primary diagnostic tool used by Kawumpuli spirit for detailed health assessment, diagnosis and establishment of the cause problems, illness and diseases associated with Lubaale.

Mukasa

Mukasa wa mikisa na bweeza - Mukasa is for good luck and blessings

Mukasa agaba zadde – Mukasa offers fertility and children in a family

Mukasa is associated with white colour

Musoke

Embuzi ya Lubaale Musoke nkazi nga ya Luyina/Kiyina - Musoke is the only male ancestral spirit which require and given a female adult goat for sacrifice rituals.

Luyina is a coloured bar across the belly of the animal, usually a female goat.

It is also possible for Lubaale Musoke to demand two goats a female and male.

Musoke is the Spirit associated with menstrual cycle of women

Kiwanuka

Lubaale Kiwanuka ebibye bya langi myuufu. Lubaale Kiwanuka is associated with red / brown colour

Lubaale Kiwanuka wa Ndiga enume nga myuufu – Ancestral spirit Kiwanuka is associated with red and brown colours and is given an adult male brown sheep

Kiwanuka is one of the confidants of Bamweyana spirits and in one that can control the limits of Bamweyana spirit

Mayembe

Nina amayembe gakulirwa Jembe Lubowa - I have Mayembe led by Jembe Lubowa.

Amayembe baselikale - Mayembe are like soldier spirits

Ejembe lyange elya Lubowa telinywa mindi – My Jembe Lubowa does not smoke nor possess a smoking pipe

Ejembe lyange elya Lubowa telilina Kyooto – My Jembe Lubowa does not possess a fire place

Characteristics of Mayembe

Mayembe do not use own fire places, and indeed they do not use fire places

Amayembe masilikale tegatekwa kutuula ku Kyooto kwoota. Mayembe are soldier spirits and are not meant to sit down and gather warmth from a fire place

Amayembe gange galina enyumba yaago ne Ndeku – my Mayembe have their own specified house and *Ndeku*.

Amayembe gange tegalina fumu lyago, gatabaaza fumu lya mukamawago aba amutumye. My Mayembe do not have a specified spear, because they use the spear of the master spirit that has sent them of any mission.

Amayembe gajjanjaba - Mayembe spirits offer treatment.

amayembe gatumwa – Mayembe can be sent to do anything

amayembe gakwata olumbe negajajaba obulwadde - Mayembe are the ones sent to get rid of olumbe and manage disease

amayembe gawaba nyo – Mayembe often go off the track

Mayembe, currently have been used over-the-board, Mayembe are wrongly used to do anything and subsequently make the roles of Muwanga, Muzimu, Misambwa less relevant. Time comes when it is the Mayembe to call in their boses, the Muzimu, Misambwa for particular tasks, yet traditionally it is the Mayembe to be given tasks by Muzimu, or Misambwa.

Abalongo (Twin spirits and spiritual forces)

Lubaale waffe alina abalongo be – Our Ancestral spirits have twin spirits

Abalongo bajjanjaba; Bwoba nga olina ebizibu, oba obulwadde, osobola okuteeka ekigali mubalongo beekika nowona olumbe, obulwadde oba obuzibu bwo.- Abalongo are spirits and can offer treatment.

Health management

Mu Lubaale tujanjaba mwooyo. *Lubaale* is used to treat the spirits.

Lubaale asawula abantu bona, sibakika boka – Ancestral spirits manage health for all people not only its clan members

Causes of health, illness and disease

Spiritual causes

Spirits are known to be responsible for causes of good health, illness and diseases. When the spirits are acknowledged and well harmonised, they cause good health to individuals, family, clan and the whole community.

However, when spirits send signals and they are not acknowledged, they persistently send more with increasing intensity and magnitude to the extent of causing illness and disease in individuals, family, clan or community, with increasing intensity and magnitude until there are acknowledged and harmonised.

Spirits are so determined not to be failed in their mission of harmonization. Spirits can cause problems, illness and disease when spirits want to be taken out of imprisonment and out of hard-labor work (obuddu). Spirits can cause problems illness and disease when they were led astray (*Empewo nga yawabizibwa (yawaba*)

Cultural causes

Abaana oba abalongo nga babanja okudda mukika kyabwe – children or twins and twin forces (Balongo) can cause problems, illness and disease when they are demanding to be taken to their families and clans

Muzimu nga gubanja ezzadde lyaagwo – When the Muzimu is demanding for its offspring (children or grandchildren) to be taken to their rightful family and clans.

Other causes

Diagnosis

Many spirits are able to identify and distinguish spiritual and non-spiritual causes of problems, illness and diseases, although not in greater details. Spirits most known for diagnosis are Muwanga, Kawumpuli, Mukasa, some Mizimu and some Mayembe. Spirits use diagnostic tools called *Mweso*. Mweso has many types but most known are mweso gyengatto used by Muwanga and Mweso gwensimbi used by spirits Kawumpuli, Mukasa and Muzimu.

The major ancestral spirits for health assessment and detailed diagnosis are Muwanga and Kawumpuli.

Munono a Mayembe si malaguzi - Originally Mayembe spirits are not used for health assessment nor diagnosis and Mayembe did not have mwaso.

Mweso

Omweso can be used for consultations of any type/kind. One mentions the issue and the mweso will provide the solution.

Originally, *Mweso* were used only by royals for better understanding of various issues. However, latter, some local people who were very bright got to know and use *Mweso.*

Mweso communicates with its empowered owner and it takes time to learn. *Omweso guwangibwa eri nanyini gwo.*

Mweso gwe ngatto

Omweso g'Engatto za Muwanga has nine (9) rectangular pieces of animal skins;

Mweso gwe nsimbi

Mweso gw'ensimbi constitutes of many items including Ensimbi engezi, ensibi ensiru, plant parts, animal parts, rocks, amatembe, enje, ntalogwa, sente zebinusu, ………

Treatment

Each spirit has its own unique regalia, requirements, process and particular ways of working. Although currently one spirit may be used to do multiple roles, originally each spirits had particular spiritual roles in relationship to Lubaale, the problems it may cause and the healthcare and treatment methods.

Health improvement, prevention and protection

Different spirits contribute variably to health improvement, prevention and protection.

Rituals

Rituals are a place of learning to an inquisitive person who gets interested in what is being done, why it is done, why that way and what materials are used and why those particular materials. The rationality of most rituals performed are confirmed by Mukasa. So the presence of Mukasa is of significance before or after rituals, even in simple rituals

Most rituals that involve passing on powers (*okuwanga*) require the physical presence of spirit Muwanga

Communal rituals

Annually or occasionally the whole family gather *for* communal rituals, which include ritualistic animal sacrifices, offerings, communal meals, singing and drumming in a jovial mood to appease ancestral spirits

Communal rituals have been used for healing for many years by our ancestors

Emikolo gitebenkeza omwoyo, nekiyamba omubiri okusuuka – Rituals bring peace to the soul, which helps the body to heal.

Emikolo gitambulira mubuwabuzi bwampewo – rituals are guided by ancestral spirits

For communal meals (ebijjulo), there should be intention and purpose. Communal rituals; Bind people together, especially family members, are for thanks giving, express appreciation, are contributed to by all participants. Communal rituals serve for protection and prevention (*obukuumi, okugema n'okutangira*)

Communal meals (ebijjulo)

Communal meals are contributed to by all people concerned. The fumes/ steam that comes out of the meal is most significant for the spirits. Communal meals constitute foods, drinks, fruits among others.

Communal meals are used to unite family members, *Okukakanya obusungu bwempewo* - to appease the spirits and lower their wrath), *Okwetonda /Okusaba obusonyiyibwa* - to apologize and request for forgiveness, *Obukuumi* – protection.

Foods;

Drinks; local brew (mwenge omuganda, banana juice,)

Fruits: normally 7 or 9 different ripe fruits ready to be eaten

Sacrifice (Saddaka)

Emikolo gitambulira wamu no kulamiriza - rituals move together with supplications

Saddaka kikulu nyo mu mikolo gyokujanjaba - Sacrifice is very essential in healing rituals

Offerings

Communal Prayers

Ancestral Spirits direct their praise and prayers towards the creator and his creation *“empewo zitendereza Katonda n’obutonde bwe”* including nature, natural spirits, twins and twin forces, other spiritual entities,

Abalyamere basaba *Amagezi, Okumanya n’okutegera, obulamu obulungi, okufuna ensimbi, ezadde eddungi, obuwanguzi, omukisa. okuwebwa, okuganja, okwagalibwa, okugabirirwa, emirembe mu nsi* – human beings mostly request for wisdom, ability to know and understand, good health, wealth, health and well-behaved children, success, blessings, being loved and favored, being given and peace.

Places for ancestral spirits and spiritual significance

Natural places

In Buganda Natural places (Ebifo byo bukiro) include forests, mountains, rocks, water bodies such as Nyiize, Walusi, Tanda, Bukasa, Nalubaale (Lake Victoria)

It is a mandatory requirement for the spirit mediums to visit the natural places (*obukiro*) where their spirits have their bases, especially in this case for spirits Muwanga we Kaliggwa, Mukasa, Kiwanuka and Kawumpuli

Shrines

kino Kiggwa kya Muwanga kya kika. This is a clan shrine headed by Spirit Muwanga.

Mbuga

Mbuga bifo byo bukilo bwa manyi gobutonzi – Mbuga are places where natural forces and powers descended. Mbuga have natural forces and powers, have natural spiritual powers, each *Mbuga yo kubutonzi* has one major natural spirit **Misange** but not Misambwa.

Lubiri

A new Lubiri always became significant whenever a reigning King visited that shrine. Lubiri has many shrines including that of Ndawula, Bachwezi ……..

For humans, Lubiri is for a King even when there are many royals (*Balangira n’Abanbejja*), however, if there is no King, then the Lubiri belongs to all the royals

Lubiri may have two basis forms (1) Lubiri kubutonzi (2) Lubiri ku buzaale

Fire places

Ekyooto kya Misambwa na Mizimu kutuula kwoota. A *Kyooto* (fire place) is meant for Misambwa and Muzimu, but not Mayembe

Incense (Kabaane)

I put the incense on fire at the fire place to immediately purify the environment, attract ancestral spirits, clear participants of any bad intentions and to facilitate healing by repelling any bad spirits causing the illness

Ekyooto

There are many forms of fireplaces that serve various functions and demanded for by particular spirits like Misambwa, Muzimu, Mayembe and Balongo. In my case here at this place, I have fire place Gombolola, for Misambwa Muwanga and Ddungu, and for Muzimu. I do not have fire place for Mayembe.

Kyoto can be used to call family members to get together (case of Suku at Kikumbo)

Muwanga alina ekyooto kye.

Ekyooto kisinziribwako okusitila amanyi n'obuyinza bwe Misambwa ne Mizimu – The ancestral fire place is the basis for activating ancestral powers and abilities for Misambwa and Mizimu.

Ekyooto kisobola okusobezebwa. – The ancestral fire place can be adulterated and its abilities weakened.

Ekyooto ekyasoba tekiba namyani – The spoilt ancestral fire place is without power and authority

Emindi

Nze bwenywa emindi enambika ejja – For me when I use a smoking pipe, I receive appropriate spiritual guidance on what I am supposed to do for my clients to provide for their health needs.

The use of a smoking pipe is dependant on the spirit medium and the ways s/he connects with his/her ancestral spirit for communication. The smoking pipe facilitates connectivity and communication between the spirit and spirit medium, and the information flow from the client to the spirit medium in his human capacity.

Symbols and symbolism

There is a connection between the astronomical, physical and spiritual worlds and they are connected to our thoughts *"waliwo enkwatagana wakati wobwengula, ensi, n'emyoyo ebiyungirwa mubilowoozo byaffe"*

"Buli Lubaale alina langi ye" meaning that every Lubaale has its associated colour

Colours

White

White colour is associated clear ways or paths, brightness, good things, fertility, blessings

White colour for a cloth is universal for most spirits. even Kiwanuka spirit can possess a person dressed in white Kanzu.

Trainees (Batende) start their trainings while dressed in white Kanzu, which enables various spirits to possess them.

Brown

Muwanga akozesa bi myuufu. Spirit Muwanga is associated with brown colour

Back

Yellow

When a person is dressed in yellow colour, there are some spirits that can not possess him/her.

For example, when dressed in yellow colour, Muzimu gwa Ssekabaka (the spirit of a former king) can not possess him/her.

Animals and birds

Lubaale Mukasa awebwa embuzi enume enjeru etukiridde – Ancestral spirit Mukasa requires and is given an adult, male, white goat without any damages.

The birds and animals for the Muzimu are normally black in colour.

Plants and other artifacts

There are no specific plants associated with Muzimu.

Walking sticks

Most ancestral spirits have their attached regalia and artifacts. Walking sticks have spiritual strength and powers. The walking sticks for Muzimu (photo), for Musambwa (photo), and Mayembe (photo), are characteristically different
