# Supplementary material for: Perspectives on health, illness, disease and management approaches among Baganda traditional spiritual healers in Central Uganda
Source: PLOS Glob Public Health. 2024 Sep 6;4(9):e0002453. doi: 10.1371/journal.pgph.0002453 (PMC11379289; doi:10.1371/journal.pgph.0002453)
Supplement: S9 Data — (PDF) [file pgph.0002453.s009.pdf]

Study participant 9 transcriptions

Contents

20200520 Mpanga transcriptions 1
Socio-demographics 3
Mulubaale 3
The process of becoming a Mulubaale 3
Kwaaza lubaale 4
Sources and Access to healthcare information 5
Sources of healthcare information 5
Access to healthcare information 5
Incense 5
Okusamira 5
Words and phrases that describe Health, Illness and Disease 6
Health 6
Illness 6
Disease 6
Health management 6
Assessment of clients (Diagnosis) 7
Kulagula 7
Omweso – 7
Okusawula 7
methods of treatment 7
Rituals and ceremonies are used as therapies. 7
Kwanbulula 8
Kuganga 8
Kusandaga (Scarification) 8
Communal activities 8
Communal meal – ekijjulo 8
Materials used for health management 8
Plants 9
Animals, birds, reptiles 9
Others Liquids, soils, rocks 9
Forms of medicines 9
Concoctions 9

Decoctions ... 9
Others ... 9
Spirituality ... 9
Prayers ... 10
Ancestral Spirit – Lubaale ... 10
Identification of authentic ancestral spirits ... 11
Kitambo ... 11
Misambwa emitonde ... 12
Muwanga Ssebyoto ... 12
Ndawula (kabaka) ... 12
Mukasa (Kabaka) ... 13
Misambwa emizaale ... 13
Muwanga ... 13
Mayanja ... 14
Kawumpuli ... 15
Bamweyana ... 15
Mukasa ... 15
Kiwanuka ... 16
Musoke ... 16
Mizimu ... 16
Mayembe ... 18
Lubowa ... 18
Kalondoozi ... 18
Kasajja ... 18
Namuzinda ... 18
Sacred places ... 18
Nature (Obutonde) ... 19
Forests and trees ... 19
Waters ... 19
Mountains and anti-hills ... 19
Shrines (Masabo) ... 19
Fireplaces (Byoto) ... 19
Symbols and symbolism ... 20
Spears and arrow ... 20
Plants ... 21
Animals, birds, reptiles and insects ... 21

Animals........21
Birds........21
Regalia........21
Reptiles........22
Regalia for Ancestral spirits........22
Dreams........22
Causes of illness and diseases........22
Witchcraft........22
Animal sacrifice........22
Referral........22

Socio-demographics

My name is (name withdrawn). I am 59 years catholic male, married Muganda of Funbe Clan. I stopped in Primary Six (P.6). I am a *Mulubaale,* staying in Gomba County (Saza), Gomba District and practice substance farming. I have 29 years of practical experience as a Mulubaale.

This is a personal shrine (Sabo), but I head our ancestral clan shrine ekiggwa and the clan ancestral spirits (*nze mukulu we kiggwa kye kika kyaffe, n'empewo zaffe)* where family gatherings take place. I am not in any formal traditional healer's association because, I become a Mulubaale through ancestral appointment by clan spirits. I was not trained through any associations.

Traditional healthcare spiritualists have professionalism

Mulubaale

The process of becoming a Mulubaale

The process for me to become a Mulubaale started when I was in primary three (P.3). I failed to continue with attending classrooms because tears were running out of my eyes, I could not see the blackboard and I become very irregular in my school attendance. When I informed my maternal uncle (kojja), a spiritualist, he later called me to their clan ritualistic gatherings. This was in 1973 and spirits possessed me for the first time. He involved me for most of the gatherings he conducted as spiritual healer and when possessed, I was involved in offering treatment to people during the ritual session.

We were staying next to a lake and fishing was the business and source of money for all my friend. As I grew up, I become stubborn and refused to join my uncle during his healing rituals, I preferred fishing since I had gathered fishing nets and could get money. This was when my suffering started. Most of the time, my fishing nets got entangled and I could spend most of the time trying to disentangle the fishing nets, so I had not time to join my uncle for healing rituals.

Problems continued in my life as we often capsized in the lake, many of my friends died and. I survived but with severe bodily injuries. (He opens his trousers and shows me the scars out of such injuries). My last time in the lake was unbearable, we lost everything, two of my friends died and I sustained deep injuries. Since then, I vowed never to go back fishing in the lake up to today. I latter understood that the need to harmonize my ancestral spirits was source of all the problems.

Family members of my father had also experienced multiple problems and on various consultations, they were told that they needed to explore ancestral spirits to understand the source of their individual problems. I joined the family and other clan members who organised for the ancestral exploration (ok*waza empowo z'ekika)* at a spiritualist place. We were about eighty (80) people and this was my first time to know and interact with most of my family members. Most of them saw me for the first time.

The process of exploration of ancestral spirits proceeded very well until when the unexpected happened. The ancestral spirits possessed me, instead of the expected family members who had even financed the process. The family split up and the process was halted as those who had financed it got annoyed and left.

After some months I was guided by the spirits to Mulubaale and trainer in the neighbourhood who was having a training session. On arrival, spirits possessed me and I joined the drummer group and started singing. To the admiration of the trainer, at my young age, I sung ten (10) good new songs of *Lubaale* throughout the night under the influence of Bamweyana spirit, and he invited me to join him wherever he went for training. I used to sing with my uncle who is over 100 years now, but when he sing now, it is as if he is only seventy years.

The trainers subsequently offered to harmonise my *Lubaale* at no labour cost provided I bought all the requirement of animals, birds and local brew that *Lubaale* had demanded. I got some money, bought the white he-goat for spirit *Mukasa*, the brown male sheep for spirit *Kiwanuka*, the required hens, local brew and food. The *Senkulu* (trainer) advised me to invite my family member, who came for *the Kusamira* process. Jembe Kalondoozi also appeared and was given it he-goat. I underwent training by both my *Senkulu* and my uncle with whom I participated in most of their training and ritualistic activities.

Kwaaza lubaale

Kwaaza lubaale is a process of exploration of ancestral spirits.

Kwaaza lubaale is usually prompted by sufferings by family members, who on consultation with spiritualists are advised to explore spirituality to establish the root cause of their problems.

Kwaaza lubaale is done for the family by a spiritualist to establish family ancestral spirituality and its demands of the family

Okwaza is when a family goes to a spiritualist to consult and explore family spirituality, its status and requirements.

Okwaza is not *kusamira*.

Sources and Access to healthcare information

"Mumulimu gwange ogwokujanajaba, nfuma obumanyi nga mpita mu biroto, obubonero, n'ebikolwa." - During my healthcare practice, I get healthcare information through dreams, signs and actions.

"nsubira obubaka bwenfuna mu biroto buva mu bajjajjange" – I think these massages through dreams used to come from my ancestral spirits.

Sources of healthcare information

"ndota, kyendose nekibawa era nekirabako". – I experience and see what I dream about. This gives me confidence about my source of information and promotes my healing abilities in my community.

"ntela okumanyira ku bubonero n'ebikolwa". I normally get information through signs and actions

When I spend a night or sleep in the shrine during the day I get a number of messages through dreams.

Access to healthcare information

"Mubiroto nfuniramu obubaka olusi nempewo nezinjogerelako nga zirungamya abazukulu kubyobulamu bwabwe." I access information through dreams, and at times the spirits directly talk through me advising the clients what to do regarding their health issues.

Incense

I use the Incense to clear the space of any bad spirits, facilitates communication with ancestral spirits and for ritualistic healing

Okusamira

okusamira kwekuteka munkola ebyava mu kwaaza Lubaale, okumuwa byeyasaba n'okumukolera byeyetaaga - *Okusamira* is the process of implementing the results of ancestral spirits exploration, offering its demands and acting upon its requirements.

Okusamira is a ritualistic process of praising ancestral spirits by a family involving the ancestral spirits possessing family members and verbally talking through mediums to the family members, giving blessings and availing opportunity to the family members to consult with the spirits regarding their concerns.

Tusamira *Lubaale* – We praise ancestral spirits

Lubaale asamirwa

Ejembe, Omuzimu, omusambwa, Abalangira tegisamirwa naye biberako bubeezi

Words and phrases that describe Health, Illness and Disease

(I, the researcher mentioned some words describing health, illness and disease. already said by the previous respondents for his expanded knowledge).

Health

Obutebenkevu - Good health

Obweza kitegeeza Bulamu Burungi na Mirembe – *Bweza i*s associated with good health and peace.

Illness

Omuntu olumbe lumukubye – somebody has suddenly been attached by yet undefined bad health condition.

olumbe lugwa bugwi – Illness normally occurs suddenly happens.

Ekisirani lumbe, kibuza emirembe, kyonoona ebintu byo. – *Ekisirani* is an illness, it removes peace and spoils everything and money.

Enyiike ebera mumutima era ekwata mwoyo, - *Enyiike* is an illness that affects the soul.

Disease

Obulwadde kyoleka obumu kubobonero obuli kumubiri, okugeza nga enjoka - *Disease* express some of the symptoms associated with the physical body, example colic abdominal pains.

Obukosefu ntandikwa ya lumbe oba bulwadde.. Obukosefu is body weakness that may be the beginning of illness or disease.

Health management

During health management, the spirits of different people collaborate with each other.

Mpewo zezijanjaba - Spirits carry out treatment.

Herbal medicine is imbued with spiritual power used in treatment.

The major spirits for health management are mainly three namely Muwanga, Kadduwanema and Mukasa.

The major spirits for health care have their main Mayembe (soldier and protective) spirits. Lubowa for Muwanga and Kasajja for Mukasa.

Bamweyana is not a major spirit in health management and Bamweyana does not have a Jembe that it works with.

Assessment of clients (Diagnosis)

mpa omuzukulu akaseela akawelako ambulire ebibye nga twogelezeganya naye. I give enough time to the client to tell me more during consultation with me.

Kulagula

Kulagula is divination

Okulagula is being told by a spiritualist about something known or unknown to you to assist you to take appropriate actions.

Omweso –

Omweso gwa Muwanga

The major ingredient of *omweso gwengatto za Muwanga* is the piece of skin of the brown bull sacrificed during *Muwanga*'s harmonization process.

Okusawula

Okusawula is associated with healthcare

Okusawula is a healthcare process that starts with health assessment (*okukebera/Okulagula)* through management or treatment health concerns of an individual or family.

methods of treatment

Nsinga kozesa bigambo mukujanjaba abalwadde bange. I offer treatment to my clients mostly through use word (*bigambo*),

Eddagala kyolitumu okukola kyelikola, bwolituma okujanjaba lijanjaba – The medicine will do what you send it to do, when you send to treat it will treat

I use particular words to communicate to the prepared herbal medicine and instruct it to perform certain health functions. These particular words add spiritual power to the herbal medicine which in turn is used in treatment rather than the active ingredients of the herbal plants.

The herbal medicine is imbued with spiritual power depending on the name of the plant used in relation to the health function it is going to perform. When picking the herbal plant, what you instruct it to do using words is what it does, what you call it is what you send it. Spirits carry out healthcare practices.

Methods of health management are ritualistic

Rituals and ceremonies are used as therapies.

Every person has spirits,

rituals and ceremonies bring various people together with their spirits, which congregates their spiritual powers to enhance healing.

Rituals and ceremonies are carried out by spirits. The spirits decide when, why and the rituals and ceremonies to be used as for healing.

Health care rituals include Kuganga, Kwambulula, kunaaba byogo

Kwanbulula

Emiwambo is a combination of herbal plants and spiritual symbolism used in ritual of cleansing (*okwambulula*).

Kuganga

Okuganga kwetangira eddogo, ebisindikirize, ebikwateko, ebibuuke n'ebirye. Okuganga is a preventive measure against any form of witchcraft, sent, touched upon, jumped and eaten.

Okuganga kikuuma omubiri n'empewo, - *Okuganga* protects both the physical body and the spirits.

Kuganga is for prevention and protection

Kusandaga (Scarification)

Okusandaga yemu kungeri omubiri jeguyingirizibwamu eddagale neliyita mumisale kumubiri neliyingizibwa mumubiri nga liyita mumusaayi - Scarification is a healthcare method (kusandaga) used to introduce preventive, protective or health promotion medicine into the body. Scarification involves cutting the human skin to let our some blood which blood is touched with powdered medicine to let the medicine enter into the body.

I do not use scarification in my treatment methods, because the process involves letting out and touching human blood which is contrary to the norms of my spirits. *Ennono ye mpowo zenkongojja tezikolagana na musaayi gwa muntu*.

Communal activities

Communal meal – ekijjulo

Ekijjulo ekitukiridde kijjanjaba – a communal meal perfectly done plays a role in effective healing

Materials used for health management

The instinctive communication between the spirits, spirit medium and the natural materials of plants and animal kingdom is very significant and key to their therapeutic values and abilities.

The therapeutic value of the natural material you just picked are different from the materials requested from nature for therapeutic use. " eddagala lyonoze ne lyosabye enkola yaalyo yanjawulo"

Plants

Animals, birds, reptiles

Others Liquids, soils, rocks

Waters

Lubaale Mukasa akozesa mazzi – Lubaale Mukasa uses water

Lubaale Mukasa akozesa mazzi okugaba obweeza - Lubaale Mukasa uses water to give blessings

Local brew

Lubaale tanywa mwenge - Lubaale does not drink local brew nor alcohol

Lubaale Mukasa, Musoke, ne Kiwanuka tenywa mwenge – Ancestral spirits Mukasa, Musoke and Kiwanuka do not drink alcohol

Lubaale does not use alcohol for drinking nor include it as a material for ritual cleansing *(ekyogo and ekyogero).*

Milk

Forms of medicines

Concoctions

Decoctions

Others

Spirituality

Nzikiriza nti Katonda jaali ne mpewo ze ezenjawulo - I believe that the Creator exists and the spirits are His creation. Both Creator and the spirits are invisible.

Bwensaba Katonda n'obutonde bye omuli n'empewo nzirwamu - I believe that when I pray to Creator and his creation including the spirits I get the response I want.

Prayers

I believe in prayer. I believe in the powers of prayers. Prayers have power.

All things on earth belong to, Creator (Katonda) I have to pray to the Creator.

bandetera omulwadde nga muyi nange mubuntu nemutya, naye empewo bwezinkwaka nezimujanjaba nawona, nange newunya – when a terminally ill client or patient is brought to me I humanly fear to hundle, however, when the spirits possess me and treat the patient, I also wonder how the spirits work. That is why I call upon and thank the spirits through prayers.

Communal prayers are more effective that single prayers. (*Agali awamu gegaluma enyama)*

Ancestral Spirit – Lubaale

Tonda ye yakola Wanga, Wanga y'azaala Muwanga. Wanga ye nanyini Lubaale ela Wanga yeyawa Muwanga amanyi n'obuyinza eli empewo za Lubaale zona. Tonda created Wanga and Wanga produced Muwanga. All Lubaale belonged to Wanga who gave Muwanga power over other Lubaale.

All ancestral spirits were produced as human being

Every spirit has its own specialty in healthcare functions.

Nkongoja Lubaale, Mayembe, Misambwa, Mizimu, Abalongo na Abalangira – I a medium for ancestral spirits in forms of Mayembe, Misambwa, Mizimu, twin spirits and royal spirits.

Lubaale ye *Mukasa*, Nende, Wannema, Musisi, *Musoke* ne *Kiwanuka*.

Buli Lubaale aba ne Ssenkulu amulungamya – Each set of clan ancestral spirits have a *Ssenkulu* who spearheads all its harmonization rituals and ceremonies.

Each *Lubaale* has an origin and belongs to an ancestral lineage.

Lubaale tanywa Mwenge. Lubaale does not drink alcohol.

Kiwanuka, Musoke oba Mukasa basobula okubera Omuzimu, ejjembe oba Omusambwa – the name Kiwanuka, Musoke or Mukasa can be for Muzimu, Jembe or Lubaale and their characteristics and roles may be different.

The three most significant ancestral spirits for healthcare among balulaale are Muwanga Sebyoto, Kabaka Mukasa and Kadduwannema.

What is *Lubaale*? *Lubaale ze mpewo ezisamirwa nga Mukasa, Kiwanuka, Musoke, Nende, Musisi, Wanemma. Lubaale* were people who once lived on this earth, had parents and did many good and miraculous things

Empewo endala ziberako bubeezi eg. Omuntu tasamira Kawumpuli, aberako bubeezi Kawumpuli. Omuntu aliko Ejembe, Omuzimu, omusambwa,

There are things which spirits cannot do. Spirits cannot put on a drip on a patient, such patients are referred to western trained doctors.

The details of the methods of the working of particular ancestral spirits vary within various Baganda clans.

Identification of authentic ancestral spirits

Genuine and authentic ancestral spirits are identified based on;

1. Their immediate environment and its content
    a. The presence or absence of things like
        i. alcohol and its containers
        ii. banana juice (Mubisi)
        iii. meat
        iv. fruits ,
2. Their medium
    a. The dressing code of its medium visa vi the spirits that possess him/her
        i. Nature (cloth, back-cloth, animal skin,..)
        ii. Related symbols and symbolism
            1. Cowle-shells, (ensimbi), head gear (Omuge),
                a. Some spirits can not tolerate presence of cowrie shells
                b. Some spirits can not possess a person putting on a head gear with cowrie shells
        iii. Colour
    b. The first words the spirit medium speaks relating to the spirits that possess him/her
3. The spirits themselves
    a. The nature and type of the divining tools (Omweso)
    b. The voice of the spirit can be used to identify them
    c.
4. The client
    a. The role the spirit claims to do
    b. The words the spirit speak out\
    c.

Kitambo

Ekitambo ye mandwa enkulu mu kika – Kitambo is the most important ancestral spirit in a clan spiritual lineage and it is in every clan.

Kitambo is related to night dancing (*busezi*). *Buli muntu yazalibwa mu busezi; yazalibwa nga ali bukunya, ne bazadde be bamufuna bali bute*, - everybody was born a night dancer, is born naked, and the parents were naked while in the sexual act of the produced him/her

Ekitambo is associated with a naked human body and the eating of human parts. It all starts when the bodies of a man and woman are meeting in a sexual act, where they are normally completely naked. Any new born child is completely naked and nearly all children suckle their mother's breast for breast milk which is part of the human body.

Kitambo is the most important spirit in the Buganda culture. *Ekitambo kitekebwatekebwa a baggunju abeddira obutiko. Ekitambo* is harmonised best by abaggunju people of mushroom clan.

anybody born of woman possess the spirit *Kitambo*.

Families that properly harmonised all the requirements of *Ekitambo* are filthy rich for it is responsible for providing riches to the family and its descendants, over generations

Misambwa emitonde

Muwanga Ssebyoto

Muwanga Sebyoto omutonde si Lubaale. -Muwanga Sebyoto is not part of Lubaale

Muwanga si Lubaale - Spirit *Muwanga* is not a *Lubaale.*

Muwanga teyali muntu – Muwanga was not a human being

Muwanga akola nyo, - Muwanga is very hardworking

Muwanga tapapa, akwatampola, - Muwanga does not harry, he takes his time

Muwanga tawawaala – Muwanga is not vocal

Muwanga is confident – Muwanga is yematila

Muwanga amanyi ekyokukola – Muwanga is very aware of what he is doing

Muwanga yebuuza nyo – Muwanga makes very many consultations with teams of specialised spirits

Muwanga delegates – *Muwanga asigira emirimugye. Muwanga okusinga alagira bulagizi ekyokulola empewo endala nezikola – Muwanga* work through instructions to other spirits.

Muwanga is accountable – *Muwanga buli kyakola ekyebalirako*

Characteristics and functions of Muwanga

Muwanga anywa omwenge – Muwanga drinks and uses local brew

Empewo ya Muwanga esangibwa mu buli kika kya Baganda ela ya wanga empewo endala zona. Muwanga spirit is is found in all the 52 clans of Baganda and is the spirit with the powers and ability to empower all other spirits, such as *Lubaale*, *Mayembe*, and *Misambwa.*

Emirimu ja Muwanga kuwanga, alagula, ajanjaba, - The functions of *Muwanga* include to imbue spiritual powers and abilities, make diagnosis and offers treatment.

Ndawula (kabaka)

Ndawula wamirundi ebiri – okutonde n'omuzaale

Ndawula causes illness and diseases with protrusions and nodules at times associated with rooting and foul smell. Disease commonly caused by Ndawula include body swellings, jiggers, and wounds

Mukasa (Kabaka)

The shrine of Mukasa does not contain alcohol

The dressing code of Mukasa is a white Kanzu

Misambwa emizaale

Muwanga

Muwanga omuzaale wamilundi ebiri. Mubuvubukabwe yali abela Kaligwa, ate Mubukadde bwe yawangalila mu Nseke. Kyekyo lwaki waliwo Muwanga w'Ekaligwa ne Muwange womu Nseke. Muwanga has two life phases, youthful Muwanga of Kaligwa village and elderly Muwanga of *Nseke* village.

My Muwanga uses his divining tools (mweso) preciously and for a very short time (less than five minutes)

In my case, if a spirit spends a lot of time using the diving tool of Muwanga (omweso), then that is not Muwanga neither Kawumpuli

Lubowa lye jembe Muwanga lya singa okutambula nalyo mu mirimuje – Lubowa is the main Jembe that Muwanga works with in its activities.

Muwanga works during day time. Muwanga does not work at night.

Muwanga we Kaligwa

Later in life, Muwanga migrated to Kaligwa village in …. Sub-county, …. District, …. County, where lived with a **two-blade spear** and later died at an old age.

The symbol of *Muwanga* we Kaligwa is a spear with two heads, his smoking pipe has two heads or nine heads.

Muwanga we Nseka

In the beginning, youthful Muwanga had **a single-blade spear** and lived at Nseke village found in ….. sub-county Mpigi district, Mawokota County. He later moved to Kaligwa.

Characteristics and functions of Muwanga

Muwanga yewanga - Spirit *Muwanga* is self-reliant during his harmonization process as compared to other sprits where he spearheads their harmonization processes. In other words, his processes are done by a spiritualist who is possessed by spirit *Muwanga*.

Muwanga demands for a brown bull for his harmonization rituals

Ekijjulo kya Muwanga kyante - The communal meal for *Muwanga* is a bull.

Mayanja

Mayanja si Lubaale, Mayanja Musambwa - Mayanja is not *Lubaale* it is a Musambwa,

Spirit Ndawula demands for and is given, fresh banana juice, fruits juice, fruits and honey

Mayanja use various creatures as it mediums including river waters called river Mayanja, reptiles such as a python snake, animals of cat family like a leopard,

Musambwa gwa Mayanja gwansa ekijulo okukikolera wansi womuti gwe kikindukindu – Mayanja spirit asked me for a communal meal to be performed below a Kikindukindu tree

Mayanja may manifest in variable categories as Kiwanuka, Musoke, or Ndawula

History of Mayanja

God created animals and rivers and assigned roles and responsibilities over them to Musoke and Mukasa. They both fought for superiority over each other claiming that they had each of them has more powers than the other. Katonda (God) wanted to prove to them that none of them is superior, instead they are equal but with complimentary roles.

So as to humble both of them, Katonda created a river through a birth canal of a woman by giving birth to a river, without the intervention of both Mukasa and Kiwanuka, which river was named Mayanja. So Mayanja shared both the wetland (water) and dryland (animal of cat family with various colours) spirits

Mayanja river was birthed as a first twin (Waswa) by a wife of a brother to King Kigala. So, River Mayanja is a twin royal spirit (Mulangira-Mulongo) endowed with lots of natural powers. King Kigala took advantage of Mayanja, the royal twin forces to become a great and longest serving King of Buganda.

This instated a unified belief that what Katonda created and the powers and authority imbued within can work independently but may be complemented by the powers of other creation or creatures.

It is acknowledged by some previous Kings of Buganda that royal twins are gifted with extreme natural powers

So the spirits work in a complementary manner

Spirits have powers to implement using nature but without control nor ownership over nature.

Mayanja is a very protective spirit of it medium.

Mayanja and Lukoma were brothers of the same grandfather Tembo the 4th King of Buganda.

The fathers to River Mayanja and Lukoma were brothers.

Lukoma was a royal son of King Kigala. Lukoma was unique, gifted and very weak child. So, because of his weakness, he was not raised like other royal sons of King Kigala.

Kawumpuli

Kawumpuli si Lubaale, ye Mulangira - Kawumpuli is not *Lubaale* but a royal spirit

Kawumpuli ye Katikiro wa Lubaale we Nyanja – Kawumpuli is the Prime Minister for all Water related ancestral spirits

Kawumpuli mulangira era y'alagula kulwa Muwanga.

Kawumpuli yeyalaguliranga Muwanga – wewava enjogera nti "bwolemwa ewange genda ku wangato" – Kawumpuli was delegated to carry out diagnosis on behalf of spirit Muwanga – the origin of the saying that "if you fail with me in diagnosis, then you go to the last option of Muwanga who uses "*engato*" for diagnosis

Kawumpulu awongerera Lubaale nalamiriza nti; ebizibu, enyimbe n'endwadde byewaleta mu family eno nga obanja,byona twaale biveewo nga bwenkwambaza ekifundikwa kino. Ojanga bulungi netwogera. Ekifundikwa kyomuzimu kiba kuddyo, nekya Mayembe. Ekifundikwa kya Lubaale yena kiba kukono. Kawumpuli akwasa omukongozi effume ne ddamula nga bwalamiriza

Okuwongerera Lubaale kwekukunganya, okukakanya n'okuteeka essuubi mu Lubaale nti ensongaze zitegeleddwa bulungi era zikolwako

Kawumpuli works only during day time. Kawumpuli does not work at night

Bamweyana

Bamweyana mulangira omusambwa omuzaale. Bamweyana is a royal ancestral spirit.

The immediate environment of the set-up (Mbuga) for Bamweyana must contain a hip of burnt or burning rubbish (*embuya ya Bamweyana ebaamu kasasiro gwe ba yokya*)

Bamweyana amanyiddwa mukusula eddalu, oba okuvaako obulwadde obusuula eddalu. Bamweyana is a known spirit for causing madness or the source of illness of madness.

Bamweyana awonya olumbe lwe ddalu. Bamweyana is responsible for treatment of illness of madness

Kawumpuli is a very thorough and precise spirit with its use of the divining tools set (omweso)

Kawumpuli will just look at the spread of the divining tool (omweso) and be able to divine the client with precise and detailed information.

Mukasa

Lubaale Mukasa asaba enkanamu - Lubaale Mukasa demands for a skin hide from a white goat of a specified sex and age.

Lubaale Mukasa does not use alcohol but only uses water or fresh banana juice (*omubisi omusogolerewo*).

Lubaale Mukasa alina effumu – Lubaale Mukasa has a "spear"

Kiwanuka

Kiwanuka usually uses lightening, he can come through lightening and strike when he (spirit *Kiwanuka*) is picking herbal medicine and in such instances lightning strikes without destroying anything. (*Kiwanuka awanula ebirungi, Kiwanuka awanula ebibi)*

Lubaale Kiwanuka rarely requests for a goat.

Lubaale Kiwanuka requests for a male brown mature sheep.

Lubaale Kiwanuka does not use alcohol but only uses water or banana juice

Kiwanuka is dressed in red Kanzu

Musoke

Musoke akwatibwako ebikwatagana n'ebyokuzaala - *Lubaale Musoke* is the one responsible for reproduction

Lubaale Musoke does not use alcohol but only uses water or banana juice

Musoke and Kiwanuka have their own specialised works or roles in healthcare. Musoke and Kiwanuka are only requested by the healing spirits to offer supportive services but both are not healthcare spirits.

Mizimu

Muzimu gwe gunanyini Lubaale - *Omuzimu* is the owner of my *Lubaale*

Omuzimu si Lubaale - *Omuzimu* is not a *Lubaale*

Omuzimu is spirit of person who was a human being.

Omuzimu is the spirits of person who is dead.

Omuzimu gukomawo gyetuli ffe abalamu mu kika okutubulira ebyagwo n'emirimu gyegwakolanga - *Omuzimu* comes back to show us what it possessed and used to do.

okugeza, bwendifa omuzimugwangwe gulikomawo negukwata kubekika kyange naddala abaana oba abazukulu, neguyagala ebyo byenettanira okukola kati kunsi – for example, when I die, my spirit (omuzimu) will come back and possess anybody within my clan especially my children or grand-children and demand for what I am interested in while on earth.

Omuzimu gweyogerera negusaba ebyagwo - *Omuzimu* speaks for itself and demand for its particular things.

Omuzimu omusajja gutera kusaba era neguwebwa Kifundikwa kyolubugo, effumu n'omuggo - A male *Muzimu* normally asks for and is given a *kifundikwa* ky*olubugo*)- (backcloth), spear and a walking stick.

Omuzimu omukazi gutera kusaba era neguwebwa essuuka yolubugo gwesumike, akambe, n'ekibbo - A female *Muzimu* normally demands for and is given sheet of backcloth that it wraps around the body, a knife and basket.

However, when a male *Muzimu* demands for a basket (*kibbo*), then that basket must have belonged to its *balongo*, the twin spirits.

Omuzimu gwomukazi tegutera kukwata musajja, okujjako nga abantu baagwo abensa nga baweddewo - It is rare for a female *Muzimu* to possess a male person as its medium, unless the worthwhile people in its clan are diminished.

Omuzimu omusajja gukwata omukazi nga omukongozi wagwo. A male spirit can possess a female person as its medium.

Nze ndi mukyala naye nkongoja muzimu musajja gunanyini lubaale wange - I am a female medium for a male Muzimu, the owner of my Lubaale

nkongojja empowo nyingi, nga zetesetese bulungi mumitendera Omuzimu gwegunanyini Lubaale a kulirwa Muwanga. – I am a medium for many ancestral spirits owned by Muzimu and headed by Muwanga Spirit.

spirits may take alcohol even when their medium do not drink alcohol - - For instance, *nze sinywa mwenge wadde empewo zenkongojja zikozesa omwenge* - I personally, and most of my ancestral spirits do not drink alcohol, but I am forced to have alcohol and its regalia in my shrine.

Nze n'empewo zaage tetunywa mindi naye empewo zankaka okuba na buli kika kyamindi mu sabo lyange kulwebanwanyi n'abazukulu. Me and my ancestral spirits do not smoke a pipe, but I am forced by my spirits to have all types of smoking pipes for the various spirits in my shrine for their comrades and clients who may need to smoke.

Bwenemelerwa okutekawo omwenge ne taaba mu sabo nfuna olumbe, olusuuka bwembitekawo - When I fail to provide alcohol and tobacco in my shrine, I am punished by getting an illness, which subside when I comply.

Characteristics and functions of Muzimu

Omuzimu gwenkongoja gunywa omwenge. The Muzimu for which I am a medium drinks local brew.

Omuzimu bwe gutagululwa gwolekelawo ensanyu lyagwo – when the Muzimu is relieved of its prohibitive problems, it visibly expresses its happiness.

Omuzimu tegubulirwa ani gwe gunakwaata – Muzimu is not advised which person to select as it medium.

In a family there are male and female Mizimu.

Mayembe

Anayembe galaba kumpi nyo, gali kubiliwo - Mayembe are short sighted, they focus more on the current things

Mayembe gesiba kukintu, naye akasera katono - Mayembe can be highly focused, but for a short time

Mayembe demand different things depending on a particular Jembe

The most mentioned *Mayembe* among Balubaale are Lubowa, Kalondozi, Namuzinda and Kassaja

Mayembe tegawongerera Lubaale – Mayembe has no powers and authority to harmonise Lubaale.

Mayembe work all the day and night.

Lubowa

Lubowa spirit protects Mukasa

Lubowa uses alcohol, but respectfully Lubowa does not use alcohol in the shrine of Mukasa.

Kalondoozi

Kalondozi si Lubaale, Jembe. - Kalondozi is not *Lubaale*, it is jembe

Kasajja

Kasajja lye jembe ekulu Mukasa lyasinga okutambula nalyo mumirimujje – Kasajja is the main Jembe that Mukasa works with in its activities.

Namuzinda

Namuzinda jembe ekulu elitambula ne Kawumpuli mumirimu je – Namuzinda is the main Jembe that moves with Kawumpuli and its activities.

Namuzinda jembe lya mu Buganda, lyaali jembe lya Kabaka Jemba or Kabaka Suuna I. - Namuzinda is a jembe in Buganda, it was for King.Jemba or/and King Suuna I. Essentially, Jembe Namuzinda was set for fighting wars and constantly informing the King of the progress of the wark.

Mauembe spirit can work remotely over long distances. Jembe Namuzinda is used in health management to effect healing over long distances.

Jembe lya Namuzinda baliwanga mu …………

Sacred places

Prayer by a well-prepared person in sacred natural place is important for effective healing.

Nature (Obutonde)

Ebifo ebimu ebitonde bilina amanyi n'obuyinza ebyenjawulo. - Some natural places are imbued with special spiritual powers and abilities. Natural places with special powers and abilities are rocks, mountains, trees, thickets, forests and rivers.

Waliwo ebifo byebibira byenogamu ebimera by'eddagala nelikola bulungi, naye bwembinoga mubifo ebitalina manyi eddagala telikola - There are some natural places like forests where I pick a certain type of herbal medicine to treats specific illness and disease, but if I get similar plants from other places, the herbal medicine is not effective.

Ekimuli kyo musambya gwo mukibira ngujanjabisa bulungi nyo okusinga ekimuli kyomusambya gwokuttale – I use the flowers of Markhamia platycalyx plant from the forest more effectively than the flowers of the same plant from the gardens

Kino kinyweza endowooza yange nti ebifo ebimu bilinya amanyi n'obuyinza ebyenjawulo agakozesebya mukujanjaba - This make me believe that some natural places have special powers that can be tapped in for healing

Forests and trees

Waters

Mountains and anti-hills

Sometimes, communal meals are carried out on an anti-hill because anti-hills are habitats of some *Misambwa*. The *Misambwa*, which use python as medium demands for offer of eggs.

Shrines (Masabo)

Muwanga Shrine

Mumbuga ya Muwanga otula nga bwoyagala naye tositama - Within Muwanga's shrine one can seat as feels like but not squatting

Fireplaces (Byoto)

Wano ninawo ebyoto bingi era buli Kyoto kilina empewo nanyinikyo eyakisaba, era kirina emirimu n'emisoso jaakyo - I have many fire places here, and each fire place has a particular ancestral spirit that requested for it, its functional role and associated taboos.

Ekyoto kino (pointing at one fire place) *tekitayokelwako nyama, ate kiri* (pointing at another one) *tekikumwa bulyomu, banjawulo abakikuma okusinzire kumperwo n'ekigendererwa* – This fire place, pointing at it, is not used for roasting meat, while the other one, (pointing at it at a distance) is not lighted by any body, it is particular people who make its fire light depending on the particular ancestral spirits and the intended functional role.

There are specific fire places associated with specified spirits for general or particular functions.

Ekyoto kya Muwanga

Ekyoto kya Jjaja Muwanga kigatta era tekiboola. The fire place for spirit *Muwanga* is a unifying Kyoto. It does not limit its user spirits and their functionalities.

Ekyoto kya Ddungu

Ekyoto kya Ddungu is associated with roasting of meat and eating roasted meat.

Symbols and symbolism

The symbols represent the presence of the spirits. When the spear of Jembe Lubowa is not present, the Jembe Lubowa might not carry out its functions but when it is present spirit *Muwanga* can instruct spirit Lubowa to carry out healthcare services which Jembe Lubowa will execute immediately.

The symbols represent the various spirits which carry healthcare services. If they are not present the spirits are not facilitated to carry out their functions.

Symbols represent the essential apparatus the spirits use in their healthcare functions.

Some spirits work through messages and signs "*empowo ezimu zikozesa bubaka na bubonero*"

Spears and arrow

ancestral spears were originally made from wood of specific trees

effumu lya Lubaale Mukasa likolebwa mumuti gwa Musaala ne liwundibwa ebyoya byenkoko ayakoze emikolo gye n'obitiit obweru – The "spear" for Lubaale Mukasa is comprised of wood piece of Musaala tree, and feathers of decoration from the chicken that was used for its spiritual rituals and white beads.

Effumu lya Lubaale Kiwanuka likolebwa neliwundibwa ebyoya bye'nkoko ya lujumba omumyufu n'obutiiti obumyufu – the "spear" for Lubaale Kiwanuka is made of and decorated with feathers of male brown *Lujumba* chicken and brown beads

effumu lya Kawumpuli likolebwa ne liwundibwa ebyoya bye nkoko ya Lujumba omuddugavu – the spear for Kawumpuli is made of and decorated with black feathers from a male black lujumba chicken.

Effumu lya Lubaale Musoke likolebwa ne liwundibwa byoya bya nkoko ya lusubi - Lubaale Musoke has a spear made of and decorated by feathers of *enkoko ya lusubi*

The symbol for spirit Kibuuka is an arrow (Kasaale).

Plants

plants have various health functions. Some plants are given names according to the functional role it is intended to perform.

Animals, birds, reptiles and insects

Animals

Lubaale Kiwanuka demands for a brown male sheep

Lubaale Kiwanuka lwasabye embuzi eba ya luyina mu bbala erimyufu – in rare cases *Lubaale Kiwanuka* demands for a goat with a patch in its belly. This is significant when the goat is to be utilized by both Kiwanuka and Mukasa his brother or Kabaka Mukasa his grand-father.

Kiwanuka akoseza eddiba lye ndiga ye mikolo je nga e kiwu kye -

Misambwa,, usually demand for particular animals to be reared at home or shrine and when these animals grow they are used during communal meals.

The *Misambwa* spirits commonly demand for animals of multiple colours (*bitanga*) which represent various types of spirits

the animal for Misambwa spirits is skinned while on the ground to enable other living things to have their share of the animals

Birds

Enkoko ya Kawumpuli ya Lujumba omuddugavu *(nzirugavu nga elimu ebimyufu)* – Chicken for Kawumpuli is black with brown feathers

Enkoko ya Kawumpuli elundibwa bulundibwa, si ya saddaaka. – The chicken for *Kawumpuli*s is just reared but not sacrificed.

Enkoko ya Kiwanuka ya Lujumba omumyuufu - *Kiwanuka's* chicken is *Lujumba mumyuufu*.

Regalia

Ekiwo

Ekiwu is when the skin/hide from the animal sacrificed for spiritual harmonization is used mat to seat on by the spirit medium.

Enkanamu

Enkanamu is when the skin/hide from the animal sacrificed for harmonization rituals of ancestral spirits Mukasa, Musoke and Kiwanuka is tied around the waist of the spirit medium

Others

Personally, I do not drink alcohol, nor smoke any tobacco however if my shrine misses any specific smoke pipes, tobacco or local brew, I will fall sick until I have provided them.

Reptiles

Timba atambulira mu nyo emisambwa naddala egiwumulira mubiswa ebifulufu - Python is a common medium for *Misambwa* whose major habitant are non-active anti-hills.

Regalia for Ancestral spirits

Regalia for *Muwanga* include Kifundikwa, *muggo, kanzu*, *mwambe*, e*ffumu*.

Dreams

A dream of a brown bull is interpreted to refer to *Muwanga*.

Causes of illness and diseases

Witchcraft

Eddogo (Witchcraft) is materials forces used to bring negative purpose.

Animal sacrifice

It is not allowed to carry out sacrifices while the spirits are possessing someone. After the spirits have seen the animal for sacrifice and receives it the spirit dispossesses the person and goes aside and the human beings slaughter the animal without being possessed by the spirits.

Lubaale tatunula ku musaayi nga ali kumutwe - *Lubaale* do not look at blood while possessing a person.

Referral

I can also refer a patient to a fellow traditional healthcare spiritualist when the patient is related to me and would think that my diagnosis is due to me knowing him/her. I can also refer a patient to a fellow spiritualist when my spirits are not specialized in treatment of the ailment of that patient.
