# Supplementary material for: Perspectives on health, illness, disease and management approaches among Baganda traditional spiritual healers in Central Uganda
Source: PLOS Glob Public Health. 2024 Sep 6;4(9):e0002453. doi: 10.1371/journal.pgph.0002453 (PMC11379289; doi:10.1371/journal.pgph.0002453)
Supplement: S10 Data — (PDF) [file pgph.0002453.s010.pdf]

Study participant 10 transcriptions

Contents

Study participant 10 transcriptions 1
Socio-demographics 2
Lubaale 3
What is Lubaale? 3
What does it mean to say *Lubaale maliba?* 3
Lwaki Lubaale Maliba? Why is Lubaale variably dressed? 3
Mulubaale 3
Becoming a *Mulubaale* 3
Ssenkulu 4
Sources and Access to healthcare information 5
Sources of healthcare information 5
Dreams 5
Access to healthcare information 5
Dreams 5
Spirits specializations 5
Words and phrases that describe health, illness and disease 5
Words that describe Health 5
Words that describe Illness 6
Characteristics of olumbe (illness) 6
Words that describe Disease 6
Spirits 6
types of spirits 7
Mizimu 7
Misambwa 8
Misambwa Misange 8
Misambwa Misangwa 8
Mukasa 8
Kiwanuka 9
Musoke 10
Muwanga 10
Ddungu 11
Royal spirits 12

Bamweyana ... 12
Ndawula ... 12
Kawumpuli ... 12
Mayembe ... 12
Characteristics of various types of spirits ... 13
Roles and responsibilities of spirits ... 13
Abalongo (Twins and Twin forces) ... 14
Other spirits ... 14
Kitambo ... 14
Balongo (Twins and Twin forces) ... 14
Healthcare management ... 15
Health assessment - Diagnosis ... 15
Treatment ... 15
Methods of health management ... 15
Use of words ... 15
Communal meal ... 15
Communal prayers ... 15
Prevention and protection ... 16
Health promotion ... 16
Symbols and symbolism ... 16
Symbols ... 16
Shrines ... 16
Places and artifacts with spiritual powers ... 16
Sacred Natural places ... 16
Artifacts and regalia ... 17

Socio-demographics

My name is (name withdrawn), a Muganda, male of 46 years and Ngeye clan. My parents are Muslim but for me I follow traditional beliefs. I am a Mulubaale for 30 years, married and stopped in Primary four (P.4). I stay in this Butambala County (Saza), Butambala District, (xxx) Sub- County, (x) village. I am a sustenance farmer.

This is a personal shrine (Sabo), but the family shrine (Ekiggwa) is at another place herr in Butambala headed by my elder brother (name removed).

I belong to Uganda N'eddagala ly'ayo traditional healers' association where I benefit by knowing and learning from each other.

Our Lubaale belongs to the family, is ancestral and when I die it my will possess my child or grandchildren at its will. "*Lubaale waffe wa nnono*".

Lubaale

What is Lubaale?

Lubaale zensonga ezisamirwa abantu abadiringana mukika. for example, if your Lubaale is associated with healing, that healing ability will be inheritable and whoever *asamira* that Lubaale, he or she will be perfecting that inheritable healing ability in the family.

Lubaale we Kika mu nono tasobola kwokyeba, naye Lubaale eyatandikibwawo kinoomu a yokwebwa. Lubaale that is inherited in the clan can not be burnt but Lubaale of an individual started by that individual can be burned at the will of that individual.

What does it mean to say *Lubaale maliba?*

Lubaale maliba means that *Lubaale* is dressed variably depending on its own customs. Lubaale may be dressed in backcloth, white cloth, or in cloths of various colours.

Lwaki Lubaale Maliba? Why is Lubaale variably dressed?

- Suppose the main Lubaale in one's clan is Mukasa, and the twin forces (obulongo) are in category of Mukasa. so Lubaale Mukasa and its twin forces will all be dressed in the white colours of Mukasa.

Mulubaale

Becoming a *Mulubaale*

I was very stubborn, experienced many problems in my childhood and failed to study. I could fight and throw stones to my fellow students. I stopped in Primary Four (P.4) and loved to stay in forests. I used to have severe headache and high fevers and at age of 15 years, I become mad and was tied on ropes

olwe bizibu n'obulwadde ebingi byenalimpitamu, taata wange munaddiini yawalilizibwa okuntwala ku Mulubaale jebakamutemera into Lubaale yeyali abanja, era nalina Kusamira. - Because of the multiple problems and illness is was experiencing, my very religious Muslim father was hesitantly forced to take me to a a traditional healer (*omulubaale)* for consultations where he was told that ancestral spirits (Lubaale) were responsible for my problems and demanded to be harmonised

When the ancestral exploration started, the spirit of my grandfather (*omuzimu gwa jjajjange)* was the first spirit that appeared and possessed me. It said it was a Mulubaale using natural and ancestral powers to heal people and had selected me for the work and unless I became a Mulubaale my health condition would not improve. My very religious had carefully kept his father's ancestral spirits regalia which he handed over to me. My father declared that everybody was free and he would not participate, support nor interfere.

The six of us, me, my sister, our aunt and three elder brothers went to the spiritualist shrine and started Kusamira, a process that praised the ancestral spirits and requested the spirits to join the process. After four days of singing and drumming, ancestral spirits (Lubaale) possessed me, and introduced itself as Muzimu by name of Kisero.

Ssenkulu asked the Muzimu spirits what it demanded from the family. The Muzimu spirit of Kisero demanded to its regalia that included *Kifundikwa kyolubugo.* It further described its spirits in form of *Misambwa*, *Mayembe* and *Balongo* the (twin forces) which were later called one by one and interrogated by the trainer for their demands, roles and responsibilities towards the family and community.

At the end of the whole process, a list of requirements made by the various spirits were compiled told to us and the Ssenkulu advised us to go back home and look for all the requirements. The requirements included various regalia, adult birds and animals of specified sex and colours, and local brew. He told us to get back to him when we were ready with all the requirements.

After about six months, we came back to the spiritualist shrine for the rituals and ceremonies of giving some ancestral spirits their requirements through the processes of *okusalira omugerengejjo, okulaayiza empewo n'okuzikakasa kumutwe.* As of now, I am not yet done with all the spirits.

I was joined by two brothers and an aunt in the Lubaale harmonization process that went on smoothly. All the spirits that appeared clarified their demands and rituals that were successfully done. By the end of the process, I was ready for the construction of three shrines. I started with the shrine for Muzimu, then shrine for Muwanga and finally the Jembe Lubowa.

Ssenkulu

Ssenkulu ye Musajja omutendesi wa Lubaale omukulu, alungamya Lubaale, omulubaale n'enkwatagana y'abwe, okutandikira ddala mukutendekebwa – *Ssenkulu* is the male chief trainer for Ancestral spirits. He does counsel the spirits, the trainee and their relationships since the beginning of the training.

Mubutende bwange, nze n'empewo zange twalungamizibwa Ssenkulu, lubaale okujiranga mubwangu singa aba ayitibwa, obutetebanga mumikolo egitamukwatako nga tayitiddwa. - During my training, my ancestral spirits were counselled by the *Ssenkulu*, to always be prompt when called upon and never to involve itself in ceremonies and issues that do not concern it.

Ssenkulu wange yatutekateka nze ne empowo zange okuba nga tukwatagana bulungi. - My *Ssenkulu* harmonized my spirit with me so we are compatible.

Empowo ezisingwa mu Lubaale zilina obukugu obwenjawulo bwezikola mu byobujanjabi - Most ancestral spirits have specialised healthcare services.

Lubaale n'omukongozzi batendekebwa okuyitira mumikolo naddala gyejesabidde – The ancestral spirits and their medium are trained through rituals and ceremonies, especially those that they have demanded for

Sources and Access to healthcare information

Sources of healthcare information

Dreams

Ebirooto mbifunilamu amakulu mangi nyo, era n'obubaka obulagula ntera bufunira mubirooto. I usually get lots of meaningful information through dreams, I also get divining information through dreams.

Access to healthcare information

Dreams

Obubaka obwokujanjaba mbufunira mu birooto, olusi nina okunonya amakulu ebirooto ebimu gebitegeeza, okusobola okumanya ekyokulolera omuzukulu wa Jjaja. – I access healthcare information through dreams. At times I need to get the interpretation of the dreams for me to understand what I need to do for client.

Obubaka obulagula butera okunzigira mubelooto. Divining messages often come to me in dreams

nfuna ebiroto ebimu nga bumbulira lumbe lwamulwadde. I get dreams informing me about the clients' illness.

Spirits specializations

empowo zirina obukugu bwanjawulo; waliwo empewo endaguzi, enozi zeddagala, n'enzijanjabi, - Ancestral spirits have various specialities, some a diagnostic, some are best in harvesting herbal medicines while other are more specialised in offering treatments.

Words and phrases that describe health, illness and disease

Words that describe Health

Obulamu: every living person is healthy the opposite of which is a dead person. Whoever is living is healthy. However, whoever comes here is not a visitor, they are looking of better health. That means they are experiencing disturbances in their health and they need to improve for the better

Words that describe Illness

Tewali njawulo wakati wolumbe n'obulwadde, olumbe bwebulwadde – there is no difference between illness and disease, illness and disease are the same.

Olumbe bwebulwadde nga butaamye – illness is when the disease gets worse. It all starts with *Bulwadde,* then *obulwadde* is treated and can get cured. However when the *bulwadde* fails to be cured and instead worsens it turns into *olumbe* and *then olumbe* leads to death. That is why the last funeral rites are done to *okwabya n'okufulumya olumbe.* This means that it is the *olumbe* which actually finally kills a person.

Olumbe olwokwabya relates to the last funeral rites

erinya lyo lumbe lisinzira ku kyaluleta okugeza Lubaale, buwangwa n'ennono oba ddogo – The name given to a health condition is determined by the prospected causations such as ancestral spirits (Lubaale), cultural and witchcraft

Characteristics of olumbe (illness)

Olumbe may come suddenly - *olumbe lukuba omuntu*

Headache that fails to respond to biomedical intervention but responds to traditional methods including rituals is *Olumb*e especially of spiritual or cultural origin.

For example, if someone's headache and subsequent madness is associated with Lubaale, unless Lubaale is addressed by traditional healthcare interventions the problems will not subside.

Words that describe Disease

Obulwadde : are health conditions that include; Ettalo (acute localised cellulitis , snake bites, headache.

Headache that responds to the biomedical intervention is *obulwadde*

Disease can be brought about by spirits. Difficulty in breathing is considered a disease. Yet changes in the breathing pattern is an indication of the presence of ancestral spirits on a human spirit medium. A child with ancestral spirits may be born with difficulty in breathing as a health condition *(obulwadde*), however through negotiations with the responsible spirit for that condition, the spirit may agree to change its ways of expressing its presence on the child.

Anxiety and depression in a patient may make worse a disease condition in the patient, however the use of words and rituals to the patient may address the anxiety and depression and subsequent improvement in the disease conditions.

Spirits

Empowo zange zeyogelela – my spirits possess me and talk for themselves

types of spirits

Lubaale is a combination of all spirits that can be put into phases or categories.

Lubaale includes spirits like Mukasa, Musoke, Kiwanuka.

Mizimu

Nkongojja muzimu gwa Kisero era guli gumu mu kika kyaffe- The Muzimu that possess me is called Kisero and it is specifically one in my clan. *Muzimu gwa Kisero gwe gunanyini Lubaale* - The Muzimu Kisero is the owner of all the spirits I have and use at my spiritual set-up, and is the head and responsible for all that takes place here. *Muzimu gwemwoyo gwomuntu eyafa*. Muzimu is the spirit of a person who died.

Muzimu gwali muntu era gusabya ebintu byegwakozesanga nga gukyali mubwomuntu – Muzimu was a human being and asks for things it used to use while still in a human form. *Muzimu Kisero mukadde nyo, mukugutereza gwasaba olubugo, effumu, n'omwenge omuganda mu ndeku,* - Muzimu Kisero is of a very old man and during its harmonization, it requested for a backcloth, a spear and local brew in *ndeku*

"Omuzimu gusobola okufuuka omusambwa okusinziira kubanga eddene lye gumaze nga omuzimu, okusinziira ku buvunanyizibwa n'ebyamagero nebyobuzira nanyini muzimu byeyasobola okukola nga akyali omuntu kunsi, oba okusinziira kunkwatagana yo muzimu ogwo nabaatu abali kunsi". When a person died and his/her muzimu spends a long time, his Muzimu may transform into a Musambwa. However, not all Mizimu transform into Misambwa after a long time, but only do so depending upon the good, courageous and or successful and heroic duties and activities they performed while as human beings on earth. It may also depend on how the Muzimu spirit related with the human beings.

"Misambwa gya mu Buganda." Misambwa is a Luganda word that refer to some categories of spirits in Buganda. Among Balubaale of Baganda, it is believed that only some spirits of the dead person (Mizimu) can transform into Misambwa.

When a client enters the shrine, the Muzimu gets to know the problems of the client and then releases the ancestral spirit with the ability to handle that particular issue. When I use the Muzimu for diagnosis then the other spirits do the treatment. *Omuzimu gusobola okunoga eddagala lyomulwadde nga guli kumutwe gw'omukongozzi* – Muzimu can have a functional role of picking medicine for its clients while possessing its medium. However, in some shrines it is Mukasa or Muwanga that appear first to understand and diagnose the client before other spirits are released to manage. Muzimu can foresee what is going to happen in future.

Omuzimu guloosa omukongozi eddagala lyokukozesa kubazukulu b'agwo – Omuzimu causes its medium to dream of the medicine to use on its clients. *Omuzimu gundosa eddagala lyokuwa omulwadde n'enkozesa y'alyo* – Muzimu shows me in the dream, the medicine to use for a specific client and instructs me how to use it. *Omuzimu gwa Kisero gumpa obubaka* – Muzimu of Kisero gives me information and directions on what to do.

Omuzimu gulaba ebirimu mumaaso ebitalabika kati – the Muzimu can foresee what is not currently visible. *Omuzimu gulagula* – Muzimu can make a health assessment and a diagnosis. *Omuzimu gwa Kisero gugaba eddagala* – The Muzimu ofKisero gives herbal medicine to its clients

Omuzimu gusobola okusomboola obuzibu, olumbe n'endwadde. The Muzimu can be cause of problems, illness and diseases.

Muzimu may have a role to protect its property. The Muzimu may possess a family member to institute disciplinary action against the family members individually or collectively. For example, last year, a whole family had an illness they could not describe and they came to consult with me at my shrine. During consultations, a spirit possessed the youngest child of about ten years and said, *"nze Kalyango, ndi muzimu. Mbavunaana okutunda ettaka ly'ebijja – olumbe lujja kubaluma okutuusa nga mununudde ettaka lyebijja.* Within three months, the family resolved the issue of the grave-yard land and were all in good health.

Misambwa

Emisambwa egimu mitonde emirala mizaale, era jaali bantu – Some Misambwa were naturally created yet others were human beings. There are also Misambwa that were born by human beings but were non-human such as rivers and animals. *Emismbwa egimu mitonde era jeyolekera mubutonde nga emigga, enzizi, ensozi oba* emiti – some Misambwa are natural and are expressed in nature such as natural rivers, wells, mountains or trees. Therefore, Misambwa are of two major categories, Misambwa Misange and Misambwa Misangwa.

Misambwa Misange

Misambwa Misange are spirits that at one time experienced or were in form of a human being. Examples of Misambwa Misange are all the ancestral spirits the Balubaale work with. These include Muwanga omuzaale, Kawumpuli, Bamweyana, Mukasa, Kiwanuka, Musoke, Kadduwanema, Ndawula, Ddungi,

Misambwa Misangwa

Misambwa Misangwa are natural spirits with original powers that did not go through any human body. Such spirits and their spiritual powers are found on mountains, water bodies, forests and other natural places. Examples of Misambwa Misangwa include Muwanga omutonde (Kisinde), Mukasa omutonde eyakkira mu kika kye Nvuma,and Kabaka Bulamu (formally Walumbe) eyakkira mu kika kye Njovu. Such clan through which the natural spirits have extra powers and authority relating to those particular natural spirits. Kabaka Bulamu is a protective spirit against bad spirits that work in the darkness like Biteega.

Mukasa

Lubaale Mukasa, Musoke and Kiwanuka were human beings whose spirits are now Misambwa

Mukasa is part of Lubaale. Mukasa's father is Kadduwanema, and Mukasa is the father to Musoke and Kiwanuka. When Mukasa possesses me, he introduces himself as a water spirit (*mulunynja*), and a son of Kadduwanema. He claims to come from Sese Island where he has his main shrine base. Mukasa presents himself as if sailing on water in a canoe boat using *enkasi*

During spirits harmonization, the water spirits (abalunyanja), Mukasa or Kiwanuka appear first to give guidance relating to the shrines at a particular shrine. Then followed by Kawumpuli and finally Muwanga. People should be guided according to their cultural roots

Mukasa abuuka omugerengejjo. *Mukasa afuna enkanamu, okuva ku mbuzi ye mikolo gye* – Mukasa got the skin-hide from the goat used for his rituals

Lubaale Mukasa yatusaba e kanzu enjeru, ekisingo ekyokumutwe, embuzi enume nga enjeru, ne ssegwanga enjeru – Mukasa spirit demanded for a white Kanzu, a white male goat, a white cock. *Ebyoya bye nkoko eyakola emikologye byatekebwatekebwa okukola effumu lya Mukasa* - The feathers from the cock that performed his rituals were put on a stick to make a spear for Mukasa

Lubaale Mukasa alina obukomo bweru – Lubaale Mukasa has white arm rings, and might have a spear. Lubaale Mukasa does not use a smoking pipe and does not smoke, nor drink alcohol. Lubaale Mukasa is a water spirit and uses water in most of its rituals. *Lubaale Mukasa alina ensuwa ya mazzi ge* – Lubaale Mukasa has a clay pot for his water. Mukasa can use only water for solving most issues or healing many illnesses and diseases of spiritual and cultural origin. Mukasa mainly use lake water for healing strong illnesses. Mukasa may use a mixture of water and plants for bathing or drinking to heal illness or solve a problem.

Lubaale Mukasa is responsible for reproduction, provisions of luck and provides blessings. *Lubaale Mukasa agaba ezzadde, omukisa n'obweeza* – Lubaala Mukasa gives fertility, luck and good things. *Mukasa yasumulula* - Mukasa is responsible for releasing people and other spirits out of prison).

Lubaale Mukasa asobola okusaba ekijjulo – Lubaale Mukasa may request for a communal meal.

Lubaale Mukasa works closely with other spirits especially spirit Muwanga. Like Muwanga, Mukasa is in all clans of Baganda and Mukasa must be heard from in most of the rituals regarding ancestral spirits involving Muwanga, because it is Mukasa that confirms the validity of what is done - *Mukasa akakasa ensonga.*

In terms of hierarchy, Mukasa is most superior to all other spirits in relation to Lubaale, that is twin spirits (Balongo), Mayembe, Misambwa and Muzimu. In this regard, *Muzimu gwe gwogelera Lubaale, Mukasa ye nanyini Lubaale ate Kiwanuka yakuuma Lubaale owa baana* – Muzimu is the head and spokes-spirits for Lubaale, Mukasa owns Lubaale, yet Kiwanuka protects Lubaale and its twin spirits. However, Kabaka Bulamu is the spirit which is responsible for releasing Lubaale and its twin spirits.

Abalunyanja balina obuyinza obusukulumu ku Lubaale mubuli kika – Water related spirits have superior powers and authority over Lubaale in every clan.

Kiwanuka

Kiwanuka agira mumusinde – Kiwanuka usually presents himself forcefully, and talks in a strong loud and firm voice. Kiwanuka normally comesor is called in cases or issues that relate to business and money, both in planning and implementation

Lubaale Kiwanuka uses a brown sheep, whose skin forms *ekiwu (photo),* for the spirit medium to seat on while doing consultations and some other spiritual activities. *ekiw*u is the skin-hide of the sheep sacrificed for rituals of Lubaale Kiwanuka and is used for sitting on by Lubaale Kiwanuka Mukasa, and Musoke.

Lubaale Kiwanuka demands a brown cock from which feathers are picked, placed on stick and decorated to make a Kiwanuka's spear referred to as Effumu lya Kiwanuka *(photo),* Kiwanuka

has a metallic hummer referred to as enyondo ya Kiwanuka *(photo),* which originally was from Sese Island and is planted in the ground on top of which is a fireplace (*ekyoto*)

Rigalia for Lubaale Kiwanuka include *enyondo ya Kiwanuka*, brown ndege *(photo)*, brown arm-rings usually made of copper, backcloth made out of Mukokowe or Muserere. *Mukokowe oba Muserere (photo and scientific name) gwe muti gwa Lubaale Kiwanuka, omukomagibwa okubugo lwe* – Mukokowe is the tree from which Kiwanuka's backcloth is made.

Kiwanuka and Musoke spirits can be source of problems, illness and disease. Whey Musoke and Kiwanuka have demands, they will cause problems, illness and disease until such demands are settled. So the person with problems, illness and disease may be referred to Kiwanuka or Musoke spirits to settle their demands so as to continue with treatment thereafter.

Musoke

Lubaale Musoke is associated with yellow colour. Lubaale Musoke uses water, honey and banana juice (mubisi).

Musoke *asaba mbuzi nkazi yaluyina* – Musoke demands for a female goat with a coloured bar across its belly. *Musoke tasaba Nkoko* – Musoke is not associated with any particular chicken

Muwanga

Muwanga is the name at the level of clans but in nature Muwanga is referred to as **Kisinde** Spirits at a clan level share a human body and are given birth to by a woman. (*kukika empewo zizalibwa*)

Nkongojja Muwanga – I get possessed by spirit Muwanga. I am a spirit medium for Muwanga.

Muwanga is a spirit, but originally a human being

Muwanga is one but referred to in two forms, a Muwanga we Kaligwa and Muwanga we Nseke.

Muwanga we Nseke was when he was still a teenager, while Muwanga we Kaligwa was when he was an old man.

Muwanga was a human spiritualist, when he died, he became a spirit Muzimu, which after many years transformed into a Musambwa.

Muwanga yawanga empewo endala eziwangibwa – Muwanga is the spirit that endows powers or empowers other spirits.

Muwanga demands for *enyondo; endege, effumu.(photos),*

The regalia for the Muwanga I use has twos – spear with two heads, smoking pipe with two heads (photos for Muwanga we Kaligwa),

Muwanga yasaba omweso gwe (photo), seddume wembuzi emyufu (photo), ne ssegwanga emyufu (photo), – Muwanga demanded from me a divining tools set, a brown male goat and a brown male chicken.

Muwanga divines, and does diagnostic functions.

Muwanga is a Kabaka and he mostly gets involved in healthcare services as a consultant thus the saying *(olaba biremye wanno genda ku Muwanga owengatto*) meaning Muwanga is most accurate in divination and diagnosis, and is most referred to last.

Muwanga Mujanjabi – Muwanga is a healer and does health management function.

Muwanga awanga – Muwanga empowers other spirits, and property.

Muwanga mulungamya era awabula – Muwanga is a good psychotherapist, psychoanalyst and advisor.

Muwanga atereza lubaale nensongaze - Muwanga is good at issues regarding ancestral spirits, their harmonization and perfection.

Muwanga yakulembera amayembe gonna era gamukolera emirimu je – Muwanga is the leader of all Mayembe spirits which implement most of his works and activities

Muwanga spirit is trusted in his health management style. He is slow but very sure. He explains most of the steps he takes in health management. Muwanga's ability to calmly explain and patiently answer all questions addressed to him relating to the cause and the approach to the solution is so convincing that it leads the client to build trust and belief in Muwanga.

Even when a client is very desperate and impatient, when he/she talks with Muwanga, the client will cool down and become patient with Muwanga and his approach.

Muwanga has his bosses and reports to Kabaka Bulamu, Mukasa and Kiwanuka. This comes to be so because Mukasa is the owner of Lubaale (all the ancestral spirits) and Kiwanuka is the keeper or protector of all ancestral spirits.

Ddungu

Ddungu gwe musambwa omukulu ogwettale - Ddungu is the main Misambwa of the wilderness.

Ddungu was a human being.

Embuzi yo Musambwa Ddungu ya lumyamyamya (photo) – Musambwa Ddungu demands for a goat coloured lumyamyamya

The skin of the goat skinned off from the goat used to harmonise Musambwa Ddungu's is left for people to sit.

Ddungu muyizzi ate byayijja bijanjabisibwa – Musambwa Ddungu is a hunting spirit and whatever is hunted has therapeutic value and is used for treatment

Ddungu offers healing and treats

Royal spirits

Royal misambwa spirits include Bamweyana, Ndawula, Kawumpuli. *Emisambwa emilangira tebagisalira* - No animal sacrifice within the *Lubiri lwa Balangira*

Bamweyana

Bamweyana spirit can be very furious, but when annoyed, he can only be calmed by Kiwanuka

Ndawula

Ndawula is a royal spirit

Kawumpuli

I am a spirit medium for Kawumpuli, but I have not yet completed its harmonization rituals.

Kawumpuli is a royal spirit. As a human being, his father was Kabaka (Name) and his mother was Naku.

Kawumpuli ye Katikiro we mpewo era ayawula empewo - Prime minister for all ancestral spirits and can make clarity within the spirits. Kawumpuli has exceptionally high spiritual powers and authority in Buganda and when a well harmonized Kawumpuli is possessing its medium, no other spirit will possess other people unless with his permission. Kawumpuli works closely with Muwanga. Although Muwanga is much older and experienced, Kawumpuli, a much younger spirit to Muwanga has exceptional spiritual powers, hence work closely with Muwanga.

Kawumpula alwaza – Kawumpuli can cause illness

Kawumpuli has a divining tool (*omweso*), in fact he uses the same divining tool with Muwanga, except that he does not use the skin hides (*Engatto za Muwanga*).

Kawumpuli does not use the power of words to eliminate or chase away illness but uses other means, especially the use of his divining tool. Kawumpuli can use his divining tools to influence anything.

Mayembe

Nina ejjembe Lubowa – I have a Jembe Lubowa

Jembe Lubowa liragula, lijjanjaba, - Jembe Lubowa can divine, offers treatment,

Mayembe Spirit originated from the Bunyoro Kingdom, they came as soldiers in the wars between the Bunyoro and Buganda Kingdoms; thereafter, the Baganda adopted them for their great works.

Ejjembe likebera, lilagula, - Ejjembe can divine, foretell and make a diagnosis.

Jembe can tell me the original cause of my clients' problems

The jjembe can see and tell me in details the presence of other spirits that have not yet expressed themselves.

A jembe may help in positioning child in the right clan in case there was a problem in that regard.

A Jembe can inform me what medicine to pick and give to the client.

Jjembe is in form of power or energy.

The demands for Mayembe vary depending on the constituents and rituals of their individual original making, the family clan lineage, the functional role and state of harmonization.

During the harmonization of Jembe Lubowa, it demanded for a black male goat, and a black cock that we used.

Amayembe gakuuma, gajanjaba, nokutumbula obulamu. The Mayembe provide protection, security, carry out treatment and health promotion.

Bwebandetela omulwadde, amayembe gamwekenenya, negamulondoola obuvo n'obuddo, ewabwe mu kika, ebukojja bwe, okuzuula obuzibu oba olumbe kwebuva, olwo negatubulira ekyokumukolera. - When a client is brought to me, I call upon one of the Mayembe spirits to come, explore the client's paternal and maternal ancestry for the origin of the problem or olumbe and then advise on the what to do next.

Characteristics of various types of spirits

Lubaale yetaaga okulabibw'awo n'okutendelezebwa, kyekyo luwaki tosamira Lubaale. Ancestral spirits like to be acknowledged and respected, that is why we acknowledge and praise Lubaale.

Roles and responsibilities of spirits

Obwetavu bwange mbowlekaza obutoonzi newankubadde obutonzi bumanyi kyenetaga – I forward my humanly needs to the creation or creator, even-though aware that the creation or creator is aware of my needs

Nkuma ekyoto, nenfukamira kumaviivi nensaba okwogelezeganya n'empewo - I make a fire, kneel and pray to communicate to the spirits.

The spirits provide in various ways and times.

bwensaba mba njogerezeganya namanyi gabutonde – my prayers are intended to communicate with natural spiritual powers.

Neyawula kubalala nenjogerezeganya n'abalongo baffe mubibbo. I get alone and communicate with our twin spirits symbolised in the baskets.

Abalongo (Twins and Twin forces)

Abalongo basobula okuleta ebizibu, olumbe oba obuludde, ate okubakolera emikolo gyebetaaga okutelera ebizibu, olumbe oba obulwadde bivaawo = The balongo (twin spirits) can cause problems, olumbe and obulwadde, performing their required rituals of harmonization can resolve the problems, illness or diseases

Other spirits

Kitambo

Kitambo mulongo wabwa Kabaka - Ekitambo is a twin-spirit of the king's lineage.

Kitambo belongs to every clan and may or may not manifest.

Buli kika kirina e Kitambo. Ekitambo ekiterezebwa mu kika, kilabilira nekigabirira abekika kyonna mu nzaalo, obugagga n'obulamu obulungi - Each family has a Kitambo, which when well-harmonised, provides for the family in all aspects including reproduction, wealth and good health.

Omuzukulu wange yandetera abenyumba ye bona nga bagwirwa olumbe bona buli kiseera. Bwenakebera nga ekitambo ky'abwe kyekibanja. Okuteleeza ekitambo ky'asaba embuzi nume nzirugavu ne ssegwanga nzirugavu netukola emikolo nebawona bona era nebatereera bulungi – My client brought his whole family to my shrine, constantly disturbed with an illness. On consultation, it was their family Kitambo that required to be harmonised. It demanded for a black male goat and black male chicken and after the rituals the whole family normal again fully cured.

Balongo (Twins and Twin forces)

Balongo (twin sets) have many types; There are Balongo (twin sets) who are born (*Abalongo abazaale*); there are Balongo (twin sets) who are decorated (*abalongo abawunde, nga Kinene*); There is *kasowole* who is a Mulongo but born single (when being born, the legs come out first); *Abalongo Ameru* is when a girl is born alongside a boy and *Abalongo Amadugavu* is when a two babies of the same sex are born together.

Tebatekwa kusaba – bwebasoba babanja bubi – issues of the twins and twin forces should be hundled with very carefully and should not go wrong. When they go wrong, twins and twin forces demand for correction in very bad ways by causing multiple problems, illness and diseases within the family and the whole clan.

Some particular individuals are responsible for twins and twin forces (*abaana*) for Buganda kingdom, for the clans and families. the twins and twin forces (*abaana*) are responsible for choosing the individual responsible for handling to completion of the issues related to them.

Mukasa spirit has to stand surety, with evidence, for the preferred individual to handle issues of the twins and twin forces (abaana)

Healthcare management

Health assessment - Diagnosis

With respect, I sensitively ask may questions to be aware, of the cultural diversities of my clients including tribe and clan taboos for effective health management - *Mbuza nobwegendereza obuwangwa nenono mubika bya balwadde bange obutasobya nga mbajanjaba*

Treatment

Amanyi n'obuyinza byempewo bye biwonya - It is the spiritual power and abilities of the spirits that heals.

Methods of health management

Belief has a high influence on one's health status and ability to fight disease. The belief that "I can" has a way it taps into some power sources that subsequently strengthen the body's ability to fight of illness and disease. Belief help the body to tap into other complementary forces to fight disease.

When the clients believe in you and your words, much of what you tell and do for them easily convinces the forces within their bodies to positively work towards health improvement. It seams the body has mechanisms that are positively or negatively influenced by the words heard.

I receive desperate clients, but if I manage to convince them otherwise, their improvement begins immediately. – "*Bwetwenafuya mubirowoozo tunafuyira ddala ne mumubiri* " -when we weaken in our thoughts we subsequently weaken bodies abilities"

Use of words

The disease that have their origin in ancestral spirits can be uplifted by use of words towards both the spirits and the patient and his/her support family members .

Communal meal

Communal meals (ekijjulo) are prepared during communal prayers.

Communal prayers

Communal prayers are involved in communal rituals and ceremonies.

Communal prayers are important because they are communal acknowledgement of the spirits.

spiritual powers are renewed or rejuvenated during the communal prayers and ceremonies

Communal prayers are necessary because the spirits belong to the clan for the benefit of the spirits and the family/clan/community.

During communal prayers not everyone gets what he/she prayed for or the results may be or not be instant, requiring some patience.

Prevention and protection

Health promotion

Symbols and symbolism

Each symbol in the shrine has its meaning and importance

Symbols are spiritual elements and their presence signify proximity of spiritual powers

The symbols in the shrine give an image of the spirits represented in the shrine

Symbols

The skin of mondo is the cloth for Musambwa Mayanja.

This *nkinga* was inherited from my ancestors and it belongs to spirit Mukasa.

Nkinga is made from specified wood by the user spirit

Shrines

The pillars are the base of the roof. The *enkata* on top is what is called *mwali.* Why it is called mwali; if you find a shrine without a mwali then that spiritualist was not well trained.

The mwali is where the spirits base is in the shrine. in my shrine, the pillar is made up of Musambya tree and a Mugobero from overseas.

Places and artifacts with spiritual powers

There are places and artifacts with spiritual powers

Sacred Natural places

There are sacred natural places like mountains, rocks, in lakes which have variable supernatural spiritual powers.

There are also sacred places with supernatural powers which do not require human beings to go there, there are sacred natural places which are visited by only one sex, say men but not women, there are those natural places which are not visited singly etc.

A sheep is not carried to Bukasa where spirit Mukasa originated from. (*tosomosa ndiga Nyanja*).

Artifacts and regalia

Nkanamu is the skin from the goat sacrificed for the harmonization rituals for Lubaale Mukasa and Lubaale Musoke which are tied around the person possessed by Mukasa or Musoke during their harmonization process.
