# Supplementary material for: Perspectives on health, illness, disease and management approaches among Baganda traditional spiritual healers in Central Uganda
Source: PLOS Glob Public Health. 2024 Sep 6;4(9):e0002453. doi: 10.1371/journal.pgph.0002453 (PMC11379289; doi:10.1371/journal.pgph.0002453)
Supplement: S11 Data — (PDF) [file pgph.0002453.s011.pdf]

Study participant 11 transcription

Contents

Study participant 11 transcription 1

Socio-demographics 3

Buluntansozi and Bulubaale 3

Buluntansozi 4

Bulubaale 4

Problems associated with becoming a Mulubaale 4

Okwaaza Lubaale 4

How I became a Mulubaale 4

Ssenkulu 5

Sources and access to healthcare information 5

Sources to healthcare information 5

Access to healthcare information 6

Omulubaale mukujanjaba 6

Motivation for Balubaale 6

Challenges in my work as a Mulubaale 7

Words and phrases that describe health, illness and diseases 7

Words that describe Health 7

Words that describe Illness 8

Words that describe Disease 8

Healthcare management (Health assessment, prevention, treatment, protection and health promotion) 8

health assessment (diagnosis) (Okukebera) 8

Diagnostic tools (Omweso) 9

prevention and protection 9

Treatment and referral 9

Health promotion 10

Collaboration 10

Causes of illness and disease 10

Methods used in healing 10

Rituals 10

Ritual cleansing (Okwambulula) 11

Fire rituals ... 11
The tongue and use of words ... 11
Sacrifice - Okusaddaaka: ... 12
Offerings ... 12
Sources of power for healing ... 12
Ancestral spirits (Lubaale) ... 13
Misambwa ... 13
Misambwa emitonde ... 14
Bulamu ... 14
Misambwa emizaale ... 14
Muwanga ... 15
Kawumpuli ... 16
Bamweyana ... 17
Kadduwanema ... 17
Mukasa ... 18
Kiwanuka ... 19
Musoke ... 20
Nabuzaana ... 21
Mizimu ... 21
Mayembe ... 21
Jembe Lubowa ... 21
Balongo (Twin and Twin forces) ... 22
Kitambo (Kinene Nyumba) ... 22
Kabaka ... 22
Ndawula ... 23
Bulamu ... 23
Sacred and natural places ... 23
Natural places ... 23
Shrine ... 23
Symbol and symbolism ... 24
Ekibbo (basket) ... 24
Olubugo (bark-cloth) ... 24
Ensawo (a bag) ... 24
Ekyooto (fire place) ... 24
Olumiri (Reed) ... 24
Ensaasi ... 25

White colour 25
Plants.. 25

Socio-demographics

My name is (name withdrawn). I am a married male aged 52 years, a Muganda of Bunyoro origin. I am a Mulangira belonging to Balangira Clan. I believe in traditional beliefs – I am a traditionalist. I stopped in Primary Five (P. 5), a traditional healthcare spiritualist and a subsistence farmer. I stay here in Buddu County (Saza), Lwengo district, (xxx) sub-county, (xx) Parish, (x) village. This shrine is a Kiggwa. I am the head of the Babiito Clan (omukulu wekika kya Babiito abalangira) and I am both a Muluntansozi and Mulubaale. I have 36 years of experience. I started my practice in 1982 and officially started operating from my shrines in 1984. I am a member of Uganda N'eddagala N'obuwangwa Bwaffe traditional healers' association. *"ekibiina kitukumira wamu nga abakugu mumulimu gwaffe"*- an association binds us together as members of the same profession.

Buluntansozi and Bulubaale

My life is associated with many miraculous happenings, problems and suffering.

I enjoyed hunting bird with catapult, join hunters with their dogs and looking after animals at home.

Miraculous happenings

I was told that my mother produced me with a small drum and a small ball.

When I was eight (8) years, I fell in a deep wide water well (*oluzzi lwo Musambwa)* and I spent two days trapped by a huge stone under the waters. The whole village was alarmed, they contributed money to get a deep-water diver from the neighbourhood district to come a remove my dead body and my grave was prepared by the village community. To the surprise of everybody, towards the end of the second day of the diver's searching, he found me trapped under a big stone, but still alive, I was removed, resuscitated to life, which marked the beginning of my spiritual journey to which my family contributed alot.

I was gifted in football, and music and drama. At school, I enjoyed drumming and singing, and songs would naturally flow. Similarly, I played football very well and I was made the school captain since primary one. However, I was stubborn and a fighter, I stopped in Primary five (P.5) and I was dismissed from five different schools because of fighting. When I get annoyed, I would feel something taking over my body, giving me greater strength, so I could fight and badly beat anybody, whatever size or age. One time fought the whole school.

Problems and suffering

Over forty deaths were attributed to Lubaale our family, which finally forced our very religious family to accept and harmonise the family Lubaale.

Buluntansozi

Bulubaale

My Ssenkulu told me at a tender age of 9 years that I was to undergo many problems in my life due to my spirituality, and indeed it has come to pass. I have seen and undergone very many problems in my life time.

Nasamira Lubaale Mukasa, Kiwanuka, Musoke, Ddungu Kawumpuli, Nabuzaana,

Ability of the traditional healthcare spiritualist to heal is due to his ancestral powers, so his/ her words of blessings can lead to healing.

Use of plants by a traditional healthcare spiritualist in healing is additional to ancestral powers and abilities.

Problems associated with becoming a Mulubaale

"Nalwaala nyo mubuto bwange". I had multiple ailments during my childhood, sickly and very thin. When I was about 12 years of age, I used to suffer from severe headache, cough and abdominal pains. Something used to talk to me from within my stomach and I could hear it, me alone. my sickness did not respond to biomedical treatment. One time, my eyes turned brick red.

Okwaaza Lubaale

Abekika nga tebanatuula empewo basoka nebazitelawo embuzi enzilugavu ennume, n'enkoko empanga eyakiwugulu egatta okujjawo obukko mu Lubaale.

How I became a Mulubaale

Ssenkulu mandwa ne *Nankulu mandwa bebatambuza Lubaale wewaffe mukika.* It is not true that anybody can become a traditional healthcare spiritualist. In our clan, it is in our traditional that only two people become healthcare spiritualists a male called *Ssenkulu mandwa* and a female called *Nankulu mandwa* and they are the only two who represent the spirituality of the clan.

Lubaale yankwata ndi mulubuto lwa nyabo, seyagalira – I was picked to serve as a Mulubaale while still in the womb of my mother, it was not voluntary to me.

Obutende bwange bwali bukakali nyo okusinge obutendeke bwamaje - The spirituality training was very difficult and tough much beyond army training.

Ndi mutendeke mu Lubaale wa Barundi, lubaale wa Bachwezi, Lubaale wa Banyoro ne Lubaale was Baganda. - I was trained in many tribal spirits including the spiritualisty of

Baganda, Banyoro and Bachwezi. In that process I come to know very many plants that are used to treat a variety of health-related ailments.

After all rituals relating to my ancestral roots in Buganda and Bunyoro, I was enthroned as both a cultural and healthcare spiritualist at the age of 16 years with over 250 people in attendance including family and community members in presence of traditional and cultural spiritualists. Extended family mobilised everything for the occasion including cows, sheep, goats, chicken, local brew and backcloths.

Every traditional healthcare spiritualist must undergo training (*obutende)* by a Ssenkulu who trains him/her the plants, animals and minerals materials, their spiritual, cultural and therapeutic values. Much of the plants and their treatments that I used to dreams were evaluated and supplemented by the Ssenkulu. It is encouraged that you keep writing as you are told and then practically pick the plants and other materials as you are told to pick them by the Ssenkulu. *(Practice makes perfect).* Also the Ssenkulu practically works with you to use those materials to effect any treatments or use them for whatever is to be done.

During my training, I was allowed to write whatever I was taught but at the end of my training I was told the all that I had learned had to be in my brain but not on paper. When I was being finally released, I was told to bring all the books I had written, and all of them were burned to ashes in front of my eyes. The *Ssenkulu* finally said to me all that you needed was in your brain and now you are released to start work.

Ssenkulu

Lubaale waffe yeyelondera Ssenkulu eyamutereza Omuzimu gwaninya nga totutuse ewa Ssenkulu negumubulira ekyotukolera, ne sente zetuba tumuwa. Our Lubaale selected the Ssenkulu (name removed) to harmonise it.

Omuzime gwaninya kumutwe, negulagira Ssenkulu ebyokukola n'esente ezokumuwa. - I was possessed by the Muzimu, which instructed the SSenkulu what to do and determined the amount of money to give him.

After 9 days of hectic *kusamira* process, the family members returned home and left me behind with the Ssenkulu for apprenticeship *(Kutendekebwa).* The Ssenkulu treated me with due respect during the training because of my unique and mysterious character.

Sources and access to healthcare information

I was trained by many trainers (Ssenkulu) (names removed).

Sources to healthcare information

"Lubaale andoosa eddagala lya bazukulu be" – Lubaale makes me dream the medicine to pick for its clients.

"neyongera obumanya eddagala nga ndi mu mutende" – I came to know more medicinal materials while under *Butende* (training and apprenticeship)

Nzikiririza era nteeka munkola obubaka bwenfunira mubirooto. – I believe in and implement the messages I get through dreams

Nzikiririza mubirooto nobubaka bwaabyo. I believe in dreams and the contained message

Access to healthcare information

"Lubaale andoosa eddagala lya bazukulu be" – Lubaale makes me dream the medicine to pick for its clients.

Obubaka nsiga bufunira mubirooto. I get most spiritual communications through dreams.

I used to get information through dreams, I am also getting information through dreams even these days.

Ntera okufuna obubaka mu biroto bwentekwa okujanjaba omulwadde nasuuka - At times I get the message in dreams how to manage the client for the client to improve.

Omulubaale mukujanjaba

Nina obukugu mu ddagala eligaba emikisa, elikwata amayembe, eligabe enzaalo, elikwata ababbi, eliganga, eliwonya eddalu, n'ebirara bingi. I have expertise in medications for good luck, for netting bad spirits, for infertility, getting thieves, for protection, treatment of madness among many others.

Olusi emikolo jejikolebwa okujanjaba. At times, rituals are the only requirement for healing to occur.

Nakola ekijjulo nenelabira okugabula empewo ya Nagaddya. Mukyala wange nalwala olususu nga lumusiwa nyo, netwawula obulili okwewala olumbe okunkwata. nemutwala muddwaliro newatabawo njawulo. Kulunaku olwokusatu nange olumbe lwolususu lwankwata, nengenda muddwaliro nesifunawo njawulo. Bwenajukira nenkola ekijjulo kya Nagaddya, Olumbe lwolususu kufembi nelusuuka era neluwona. - Just recently, my wife developed a generalised irritating itchy skin. We went to the clinic and got treatment but the medicines did not work. The skin got worse and everybody at home got scared. Much as we separated beds for fear of getting infected, her situation got worse and I got infected with the same skin infection on the third day. I also went to the same clinic but got no relief. However, when I come back home, I realised that during previous week I had done some communal meal (*Kijjulo),* but forgot to serve some spirit called *Nagaddya*. On realising that, I prepared a communal meal for spirit Nagaddya. Surprisingly, after the communal meal, both our skin itchy rash improved and cleared.

Motivation for Balubaale

It is motivating to be able to successfully manage or treat very difficult health conditions, yet I am not educated. I find interest in handling cases which others have failed to manage. I am

interested by clients who come to me convinced that they are going to get cured or their problems are going to be resolved by me.

I am interested in conducting *Kusamiza* rituals (*nyumirwa nyo okusamiza n'okuzukuza abaana)*

Nyanguyilwa era nyimilwa nyo okugaba eddagala lyemikisa

I find it easy to make the regalia for Lubaale such as *ensawo ya lubaale, ebisingo bya Lubaale,*

Kinyanguyira nyo okwambulula

Challenges in my work as a Mulubaale

I become challenged when a client is in a bad condition yet he/she cannot afford the cost of treatment. I find it difficult to loan out treatment or to remind people to pay for the treatment they got from me on credit.

Ngeri jentegeramu olumbe n'obulwadde, nebiruleta yempa obusinziro bwenkozesa munzijanjaba. My ways of understanding and appreciation of the health issue and its causes determine my approaches to health management (treatment, prevention and health promotion).

Words and phrases that describe health, illness and diseases.

Words that describe Health

Omuntu alina omukisa nafuna emirimu ye mulamu. A person with good luck and is able to get required jobs, and can afford the basic human for the individual and family is healthy.

Obulamu obulungi nga omubili gwona glkola bulungi. Good health is when all the physical and non-physical body parts are functioning properly.

Obulamu obulungi nga bajjajjaabo, n'empowo zawaka batebenkevu. Good health is when one's ancestral spirits and powers are in good harmony with the individual and the family.

Obulamu obulungi bwebo nga obwongo butebenkevu / butebemkedde. Good health an individual has a settled mental status.

Obulamu obulungi omuntu aba tawankawanka mu mutima gwe.

Obulamu obulingi bwe bulamu obutebenkevu nga bweyagaza - good health is when one has a settled good and enjoyable social health.

Obulamu obulungi tekigaana muntu kulwala naye aba alina obusobozi obwejjanjaba

Words that describe Illness

Olumbe n'obulwadde bitegeza kyekimu – Illness and disease are the same thing and refer to the same thing *"kawokadda ne kasangwawo"*

Words that describe Disease

obwavu lulwadde, Omuntu omwavu tabela mulamu -poverty is a disease, a person who is poor is not in a desired health state.

Olumbe era bwebulwadde. What is important is not the difference between *olumbe* and *obulwadde* but their root cause.

Bulwadde oba olumbe byombi bitta. Illness or disease both can lead to death

Obwavu bulwadde - Bwobela omwavu oba mulwadde – Poverty is a disease, when you are poor you are diseased.

Healthcare management (Health assessment, prevention, treatment, protection and health promotion)

Many people come to me (*a Mulubaale*) to say thank you for the healthcare service offered to them. Normally I do not even remember the people and what was done but they bring rewards to me and my spirits.

A good Mulubaale does not need to make any self-advertisement, but the spirits will bring their clients.

health assessment (diagnosis) (Okukebera)

abantu baja jendi okwebuza kubizibu ebyekusa ku byobulamu, ebyemikwano n'ebyensimbi - People come to me to consult for causes of their health, relationships and economic challenges.

Nzikiririza mu mweso gwa Muwanga ne Kawumpuli byegulaba nebyegunganba okukolela abazukkulu. – I believe the diagnostic tools, process, and the results by spirit Muwanga and Kawumpuli and their clients

Health assessment is mainly to establish the cause of the problem, illness or disease.

Health assessment establishes whether the cause of the problem, illness or disease is spiritual, cultural, biological or physical.

When the cause of the problem, illness or disease is spiritual, the assessment establishes the specific spiritual entity responsible

Diagnostic tools (Omweso)

Omweso gukubibwa era negutegelebwa empewo nanyini mweso. – The diagnostic tool is best used and understood by its owner spirit

The diagnostic spirit may consult with the investigative Mayembe spirits to establish the spirits responsible

The case is then referred to the identified responsible spirits to establish the real cause and source and look at the options of solving the issue

It the source of the problem, illness or disease includes some unmet demands, then those demands are settled between the client and the spirits responsible.

The whole information may be relied to the client by the consulting spirit, Muwanga, Mukasa or the demanding spirit or the investigative Jembe spirit.

For example, when it is established that the type of madness of the client is caused by Kiwanuka, Kawumpuli or Bamweyana, the client is to the respective spirit for understanding the cause of the madness, the plan of management, the actual management process, the settlement of the demands, the rituals to be performed, the symbolisms and the required fees and payment plan.

prevention and protection

Treatment and referral

I got a client with a severe throbbing headache that had failed to respond to medication from hospital. On consultation, Kawumpuli spirit possesses me and told him *"funa enyondo ya Kawumpuli n'ensawo ye omukongozzi abikoleko emikolo obiteke awaka wo*" – "get the specified regalia for spirit Kawumpuli, do the instructed rituals and place the regalia at his home", which we did and his headache got cured.

One time I had a medical doctor who was my late friend, called Dr. Kigayaza, (he died) from Kalisizo, who become mad while doing is doctorate. He failed to respond to Western medicine and he was brought to me in my shrine. He had been possessed by Kitambo for which I did rituals using a goat. He responded very well and got cured and continued with his studies. (What exactly did you do?) he responded that "Using the rituals, I successfully transferred the Kitambo spirit from him (Name) into the goat.

After some years, when he had completed his studies, he came and appreciated what I did to him and asked how did I cured his madness without giving him medicines?

Nzikiririza mu Misambwa emilaguzi, Muwanga ne Kawumpuli n'enzijanjaba yaagyo – I believe in the diagnostic spirits Muwanga and Kawumpuli, and their treatment prescriptions

Ekiteega has a lot of power and *ekiteega tekikwatagana na Ndiga.*

For the treatment to be successful, what are the requirement, payments and why is Muwanga so central in the process?

Health promotion

Collaboration

I support collaboration between Traditional and Western healthcare providers.

Causes of illness and disease

Lubaale atateledde asobola okuba ensibuko yo lumbe n'ebulwadde. un-happy and un-harmonized ancestral spirits can be a source of illness and disease.

Kawumpuli causes a throbbing headache

Kitambo can cause illness in many forms which are relieved with rituals

Kayizzi causes chest pain with a cough. Kayizzi is the son of Kaleguza and his wife is Nabulezi.

Musoke bwabanja akunywa amazzi n'omusaayi– Musoke causes severe dehydration and severe anaemia in a person

Methods used in healing

Amawasira guba mugatte gwaddagala - Amawasira is a combination of medicines

Rituals

Emikolo egikolebwa gijanjaba olumbe n'obulwadde– performed rituals can effectively heal illness and diseases.

Olumbe bwendukolela emikolo nelugaana okuwona nga ndutwala mubekizungi – when I perform rituals and the *illness or disease* fails to respond, then I refer it to hospital for management by Western medicine.

obulwadde n'olumbe ebiletebwa empewo zekika mbijanjaba nakukola mikolo – I manage most of the diseases and illnesses caused by ancestral spirits by performing rituals.

Nagayala okufuna obusaana bw'ekyooto okumala enaku satu (3), abaana bange bankubira esimu buli omu nga mulwadde nange nalwaala. Naye bwenategeka togende tuteme obusaana nawonela kuttale, n'abaana bankubira amasimu bona nga bawonye. Obulwadde bwaali mikolo. – One time I had neglected having the firewood "Acacia hockii" (*Kasaana)* for my one of my fireplaces (*ekyooto)* at my shrine. my children rung me when each of them was feeling ill and I also become ill. However, when I organised to go and fetch the Acacia hockii (*Kasaana*) firewood for the fireplace (*ekyooto*), my health become better and thereafter all my children rung me when their health had improved. So, it was the rituals of the fireplace by use

of particular firewood that had caused our illness and also healed us. Since then, every family member is active to ensure that Acacia hockii firewood for the fireplace is always available.

Emikolo gisobola okujanjaba obulwadde – rituals are effective in health management. Rituals are effective in health promotion. Rituals are effective in health prevention

Ritual cleansing (Okwambulula)

During ritual cleansing one uses *amawasira amaddugavu okusooka* followed by *amawasira ameeru* (plants with white flowers) to get the appropriate cleansing.

Abakazi babambulira kukitooke kyanakitembe nga bayimiride kumbugo

Abasajja babambulira kukitooke ky'ensowe nga bayimiride kumbugo

Abawala/abakazi batelwawo ekibbo – ekibabbulula

Abalenzi/abasajja batelwawa ebibbo nensawo satu-satu, Balongo, Lubaale na Mayembe

Fire rituals

Okukuma ekyoto kisobola okuwonya obulwadde oba olumbe – Fire rituals alone can be enough to effect healing of illness or disease.

The tongue and use of words

Ebigambo byo mulubaale bisobola okuwonya oba okukyusa embeera – The words of a traditional healthcare spiritualist can heal or change the situation.

Olusi nkozesa lulimi na bigambo okujjanjaba abalwadde bange. In some cases, I only use words of my tongue towards my clients to effect healing or rectify a situation.

When I use my healing words to the client, I am actually talking to his/her spirits which hear and contemplate over my words for an appropriate action. I use deliberate words to general or specific spirits to request the spirits to leave the client free especially when the spirits have a genuine cause for their action. However, I may command the spirit and force it out if it is stubborn or unjustified in its actions towards the client.

The spirits within an individual can be strengthened or weakened by the same individual depending on the words he thinks or talks out. For example, when I manage to convince the client to think and talk encouraging and good words, this strengthens his or her spirits which respond in the similar manner. Equally, if weakening and discouraging words are used to the client, his/her spirits are weakened and discouraged and act accordingly.

What we do and what we believe in inform the spirits within us which spirits act likewise.

The words spoken by spirit through its medium are very powerful and authoritative words addressed to the client, but more importantly to the spirits, within vicinity of the clients.

Likewise the saliva or handshake by the spirit medium are authoritative, powerful and very significant for the client an

Sacrifice - Okusaddaaka:

Mubigabiro mubaamu ekifo kya Mukasa

Okusalira sikyekimu no kusaddaaka – Animal throat cutting is not the same as animal sacrifice

Saddaaka tusala nte, mbuzi, ndiga, nkoko – It is the cattle, goats sheep or chicken that are used as sacrifices

Saddaaka ekozesebwa okukwambulula gwe togilyako. A person should not eat meat of the animal or bird that has been used on him/her for the ritual cleansing (Kwambulura)

Saddaaka eyokwambulula egibwako omutwe nelekebwa okusambagala– the animal is be-headed and left to struggle while dying. (Other people can eat its meat)

Saddaka the sacrifice of an animal or bird is based on one's intention and willingness. However, these days, sacrifice of animals or birds is a forced processes as ascribed by the healer, which is contrary to the original context of sacrifice (saddaka)

The words said during the sacrifice are the words followed by the implementing spirits following the intentions of the sacrifice.

The words said during sacrifice are very important while the sacrifice is being done.

Offerings

Ensozi bazisaddaakira. Offerings are made for the mountains

Saddaaka ziba zabigabiro munsozi, mumayinja, kunzizi oba kunyanja.- The offerings done in natural sacred places such as mountains, rocks and water bodies

Sources of power for healing

Nzikiririza mumanyi n'obuyinza bwempewo – I believe powers and authority of the spirits

Emisambwa giteesa negilagula nejijjanjaba. Misambwa spirits can discuss, make a diagnosis and offer treatment.

Omulubaale empewo bwemulinya, afuna obumanyi obwenjawulo – When a traditional healthcare spiritualist is possessed by ancestral spirits, he/or she gets exceptional abilities to know.

Nzikiriza nti waliwo amanyi n'obuyinza ebinyambala negajjanjaba – I believe that there are supernatural powers that possess me and do health work through me. One time people brought to me a patient who developed sudden severe pains and rigid fingers. I did not know what to do, so I smoked my pipe, and soon after, the severe pains and rigidness of the fingers disappeared and he got healed. I did not tough him, I did not pray for him nor give him any medications. It was through the powers of smoking the pipe. I cannot explain how it happened but experienced it happening.

The patient belief in my contributes towards their healing

Ancestral spirits (Lubaale)

Waliwo empewo entonde n'empewo enzaale mu Misambwa, Mayembe n'obulongo. Empewo entonde ezisinga zakiranga mu ba Kabaka. – There are natural and ancestral spirits in form of Misambwa, Mayembe and Twin forces. The natural spirits mostly descended on earth through Kings.

Muwanga Sebyoto ne Kabaka Mukasa misambwa mitonde. – Muwanga Sebyoto and Kabaka Mukasa are natural spirits. *Emisambwa emitonde ze bayita Malayika*. Natural spirits are the ones referred to as Malayika

Lubaale gye Misambwa emizaale, nga Kawumpuli, Muwanga, Nabuzaana, Nakayima, Kayizzi.

Ancestral spirituality can be weakened in its functional powers by additional foreign spiritual entities. – *empewo zekika zononebwa bwezigatibwamu amanyi agatali ganoono yaazo.*

Lubaale yena asalirwa ebisolo oba enkoko nga bisajja – it is male animals or birds that are sacrificed for Lubaale. Even if the Lubaale is possessing a woman.

Teli Lubaale ayambala ngoye. – Lubaale is not associated with cloths

Omusawo siyagaba Lubaale, Lubaale yelondela anamusamira

Lubaale webukojja, ava kuluddа lwamaama asobola okukwata kubaana be

Okusalira: Lubaale bamusalira

Misambwa

Nzijanjaba nga nkozesa emisambwa emitonde n'emizaale – I use both natural and ancestral misambwa for health management

Bachwezi ze mamdwa ze Bunyoro, nga Kaliisa. Abachwezi balunzi bante, banywa amata, era bakozesa omuzigo – Bachwezi are spirits of Bunyoro such as Kaliisa. Bachwezi use milk and ghee.

Misambwa emitonde

Emisambwa emitonde giri mwenda (9) era tegilagula; Mukasa, Kibuuka Omumbaale, Kiwanuka/Kagolo, Musoke, Kintu, Nambi, Ddungu, Bulamu, Nakayaga. Buli Musambwa gulina emirimu gyaagwo - Natural Misambwa are nine (9) and they do not divine – Namely Mukasa, Kibuuka Omumbaale, Kiwanuka/Kagolo, Musoke, Kintu, Nambi, Ddungu, Bulamu, Nakayaga. Buli Musambwa and each has specified duties and responsibilities

Emisambwa gilaba obulwadde, gilagula era jijanjaba.- Misambwa spirits can diagnose, devine and offer treatment and healing

Bulamu

Bulamu Musambwa

Bulamu si Kabaka – era kyabulimba omuntu okugamba nti akongojja Kabaka Bulamu – Bulamu is was not a King and it is a lie for someone to claim to be a medium for Bulamu

Misambwa emizaale

Emisambwa emizaale genkozesa milaguzi, gilagula gye Kawumpuli ne Muwanga – The Ancestral Misambwa spirits that I use to divine are Kawumpuli and Muwanga

Kadduwanema, *ne batabani be* Mukasa, Kiwanuka *ne* Musoke, *misambwa mizaale* – Kadduwanemma and his sons Mukasa, Kiwanuka and Musoke are humanly ancestral spirits

Ndawula, Ddungu, Bamweyana, Mayanja, ne Kawumpuli misambwa mizaale.- Kadduwanema, Ndawula, Ddungu, Bamweyana, Mayanja, and Kawumpuli are humanly ancestral spirits.

Emisambwa emizaale emigabi gye mikisa gye; Bamweyana, Nabuzaana, Ndawula, Nakayima, Kayizzi, Kawumpuli ne Namuziinda – The ancestral Misambwa spirits that I use to offer good-luck are Bamweyana, Nabuzaana, Ndawula, Nakayima, Kayizzi, Kawumpuli ne Namuziinda

Nzikiririza mumanyi n'obusobozi bwe Misambwa. I believe in the powers and abilities of Misambwa spirit

Muwanga

Muwanga musambwa muzaale. Muwanga is an ancestral spirit

Muwanga had two wives

Muwanga yabula bubuzi – Muwanga has no ancestral graves, he just disappeared

Ente ya Muwanga myuufu – The cow as a medium for Muwanga is brown. The rituals for Muwanga are done using brown cattle

Muwanga abere ne Ngoma – Muwanga has a particular drum made of its animal hide

Eddiba ye ngoma ya Muwanga liva ku ddiba lye nte ye.

Muwanga has general and specific songs *"Bazibajira kuzikuba nga zamu Mwanga, bazibajira manga eli …"*

Muwanga yasinga mu kulagula, - Muwanga is the best in divination

Muwanga alaguza ngatto, omulimu eddiba lye nte ye, gyebamusalira – The divining tool for Muwanga is composed of some hide peace got from the animal that was used for his harmonization rituals

Muwanga alaguza omweso - Muwanga uses a divining tool referred to as Mweso

Jaja *Muwanga ajjanjaba. Jaja* Muwanga is known for his ability to treat and addresses human suffering including physical, social and spiritual ailments.

Jaja Muwanga has two sets of diagnostic tools (Emweso); (*Omweso gw'engatto, n'omweso gw'empiki, ensimbi nebilala*) one diagnostic tool made of nine peace of hides of wild animals and another divining tool made of multiple items including black beads, cowry shells, among others.

Jaja Muwanga akozesa ebigambo mukujanjaba, awanga ero yawangulula Spirit Muwanga uses the powers in his words to heal, he has the powers to empower and also to disempower things and spirits

Jaja Muwanga uses the spiritual powers contained in the created living and non-living things. These include powers contained in plants, animals, reptiles, birds and insects on addition to use of waters, rocks.

Jaja Muwanga asinga kukola na Mayembe agakulirwa Jembe Lubowa, mokujanja. okugeza ettalo, olumbe olusindikirize. Ejjembe alikozesebwa kisinziira kumulimu ogwokkola, (Spirit Muwanga mostly does the healing works using Mayembe spirits headed by Jembe Lubowa, in such health conditions like cellulitis, and illnesses that are sent to a person. The particular Jembe Spirit used depends on the nature and seriousness of the issue to be handled).

*Muwanga yawanga amayembe ela amanyi buli jembe kyelisinga okulola obulungi. (*Muwanga is the spirit that empowers all the Mayembe spirit and is privileged to know the speciality of each Jembe and what each Jembe can do best)

Muwanga ye nanyini miti – Muwanga is the master of all medicinal plants

Muwanga atabula eddagala – Muwanga is good in mixing herbs

Muwanga awanga, enyumba, essabo, ekiggwa, abaantu,, emaka, emotoka, nabuli kintu – Muwanga empowers shrines, people, homes and property like houses, vehicles, and everything.

Muwanga ayambulula – Muwanga does cleansing rituals

There is a plant called Muwanga.

Omuti gwa Muwanga gwambulula enyimbe ezalema – Muwanga plant is used to remove very difficult illnesses.

Muwanga is a associated with a brown cow

Muwanga has a special place within a shrine and Muwanga has a special door he uses to enter the shrine. Muwanga does not enter the shrine through the same door as other people.

Muwanga's special place has e Kibbo (basket)

Muwanga's shrine has two doors.

Muwanga has spaces in nature such as on mountains

Its working time is determined by the spirit but Muwanga normally works during day light

Muwanga asalirwa nte. – a cow is sacrificed for Muwanga

Muwanga mulaguzi – Muwanga is a diviner spirit

Kawumpuli

Kawumpuli mandwa nzaale buzaazi – Kawumpuli is an ancestral spirit of human origin,

Kawumpuli yazalibwa nga mupumpuli - Kawumpuli was born as of a woman as a leaper.

Kawumpuli mandwa nkulu nyo mu Buganda – Kawumpuli is a very important ancestral spirit in Buganda.

Kawumpuli alina abakuumi be munanya mangi, ewange omukuumi we omukulu ye Kisekula-omuttu – Kawumpuli has its security spirits by many names, its major security spirit here is called Kisekula-omuttu.

Kawumpuli nga abanja alumya ekifuba ekyomunda ekitawona,– When Kawumpuli has its demands, it can cause chronic chest pain without cough that fail to respond to medication.

Kawumpuli mulaguzi mulungi nyo – Kawumpuli is very good at health assessment and diagnosis.

I believe in the effectiveness of the instructions given to me by Jajja Kawumpuli after using his diagnostic tools omweso.

Kawumpuli tasalirwa.- Animal sacrifices are never made for Kawumpuli spirit

Kawumpuli mulaguzi.- Kawumpuli is a royal spirit

Effumu lya Kawumpuli lisalirwa ku Ddungu, enkoko ya lujuumba omuddugavu, negibwako ebyoya n'olulimi okuwanga effumu lya Kawumpuli.

Effumu lya Kawumpuli likolebwa mubyoya bya nkoko ya Lujuumba omuddugavu

Nga tolina fumu lya Kawumpuli, Kawumpuli tasobola kulagula – Without the ritual spear of Kawumpuli, the spirit Kawumpuli can't divine

Bamweyana

Emandwa ya Bamweyana teyalinyanga kumutwe - The spirit of Bamweyana never used to possess people

The wife of Bamweyana is called Nakayaga. Bamweyana is royal, puts on a Kanzu,

Bamweyana is dressed in rags and kutiya (sisal sack).

Kadduwanema

How does Kadduwanemma perfume healing and what does he use?

Kadduwanema bwaba abanja obuvune bwakutekako buba bwa kulemela (When the spirit of Kadduwanema is demanding for harmonization, the signs of physical manifestation is lameness)

Kadduwanema mujanjabi, ela omuntu eyatebenkeza empewo ya Kadduwanema nagitambulilamu bulungi ayanguyirwa omukanya ebizibu ebiletebwa empewo ya Kadduwanema n'okubijanjaba nga akozesa omugo gwe, (Kadduwanema is a healer and a person who harmonised the Kadduwanema spiritual powers can easily understand and is able to heal problems caused by the demanding Kadduwanema spirit by use of his walking stick).

characteristics of the walking stick of Kadduwanema (*Omuggo gwa Kadduwanema guba Munene nga gulina oluwuzi oluguyitamu*) [Photo] *bweguwangibwa jaja Muwanga, guba namanyi n'obuyinza. gukozesebwa mukujanjaba naddala obulemu kumubili.* (The empowered walking stick of Kadduwanema by spirit Muwanga has power and authority and is used in healing especially physical bodily handicaps)

How is the walking stick used in healing physical bodily handicaps? *Olugo gwa Kadduwanema gutekebwa mumazzi, amazzi negakozesebwa kumubiri oguliko obulemu* (the Kadduwanema walking stick is placed in water, which water is used fo the healing of bodily handcaps)

Kadduwanema akozesa omuggogwe, amazzi oba akakomo mukujanjaba. Kadduwanamma uses a walking stick, a bungle and water for healing. his walking stick is enshrined with healing powers and authority.

*Kadduwanema akozesa nyo enkoko okumala omulimu gwokujanjaba (*Kadduwanemma mostly uses chicken for sacrifice to successfully accomplish his healing rituals)

Kadduwannema is considered a very good healthcare spirit.

Kadduwannema often uses the services of his children Mukasa, Musoke and Kiwanuka of offer treatment.

The treatment methods used by Kadduwannema are considered superior to the healing methods used by his children Mukasa, Kiwanuka and Musoke.

Mukasa

Emamdwa ya Mukasa ezina nyo amazina – Mukasa spirit is a very good dancer

Most good dancers are a medium for Mukasa spirit

The spirit of Kabaka Mukasa is a special spirit and closely related to Jaja Muwanga *Omutonde*.

Kabaka Mukasa, omusambwa omutonde yazaala omwana Kadduwanema. Kadduwanema yazaale abaana bana, abalenzi basatu, Mukasa, Kiwanuka, ne Musoke. *omuwala omu Nakku yafa nga muwere.* (Kabaka Mukasa, a natural spirit delivered four children. three were boys namely Mukasa, Kiwanuka and Musoke. The fourth child was a female named Nakku, but she died at infancy)

Kabaka Mukasa omutonde yazaala Kadduwanema, ate nga Kadduwanema ye taata wa Mukasa omuzaale. Kabaka Mukasa omutonde is the father of Kadduwanema, yet Kadduwanema is the father of Mukasa omuzaale. *Kadduwanema alina amanyi n'obuyinza ebisukulumu* (Kadduwanema has superior healing powers and authority over his children Mukasa, Kiwanuka and Musoke)

Whatever Mukasa uses is unique to Mukasa. Mukasa has a white Kisingo. The songs for Mukasa are different from the rest of the songs. Most of the songs for Mukasa relate with waters of the lake, since Spirit Mukasa is the head and responsible for all waters on earth and the breathing abilities of all humans, and other creatures. Muka ssa

Katonda yawa Kabaka Mukasa omutinde obuyinza bungi nyo kulukalu n'ekulubisi. Akozesa ebigambo, era alina amanyi– Katonda gave Kabaka Mukasa the natural spirit, a lot of spiritual powers over both the dryland and the wetlands creation. Kabaka Mukasa uses the power of his words to heal and his has powers.

Kabaka Mukasa can delegate the healing work to any other category of spirits (Muzimu, Mayembe, Misambwa, or Balongo), although Mukasa works mostly with twin forces (Bulongo).

Lubaale Mukasa, omusambwa akozesa mazzi n'amanyi agagalimu okujaanjaba. Lubaale Mukasa mostly uses water and its contained spiritual powers to heal.

Lubaale Mukasa akozesa ebisolo mukujanjaba (Lubaale Mukasa uses animals for healing).

*Lubaale Mukasa wa mikisa, era n'obweeza bwa Mukasa (*Lubaale Mukasa is for provision of good-luck and good health),

Kabaka Mukasa omutonde talagula, aja lwambonekelerwa eli abekika – Kabaka Mukasa, the natural spirit is not a common figure in diagnostic process. Mukasa is of occasional appearance towards the clan and its people

Lubaale Mukasa has children. Song; *"Mwanguwe enkuba tebakuba, abana ba Lubaale Mukasa"*

Ekifo omukumibwa ebintu bya Lubaale Mukasa tewatonya bintu bye kukubwa nkuba

Lubaale Mukasa akakasa bukakasa bibera bikoleddwa

Mukasa akola bwamisana – Mukasa spirit works only during day time

Mukasa bamusalira enjuba tenaba kuvaayo – throat cutting rituals for Lubaale Mukasa are performed at dawn before day-break

Mukasa yansaba; elyato lye, enkasi, ensuwa, ekisenso, ekifundikwa, - Mukasa demanded for from me its boat, enkasi, ekisenso and ekifundikwa

Kiwanuka

Kiwanuka is a natural spirit not ancestral

Kiwanuka is associated with fire

Kiwanuka is associated with red and brown colours

Ekisingo kya Kiwanuka kebeera kimywuufu (red)

The decorating beads (obutiiti) for Kiwanuka are red in colour

Enyondo ya Kiwanuka myuufu (red)

Kiwanuka yakubisa Laddu. - Kiwanuka is the one responsible for lightening

Kiwanuka is associated with thunder and lightening

Kiwanuka ayita ne Mukasa – Kiwanuka is closely related with Mukasa

Kiwanuka anywera kunsuwa ya Mukasa –

Kiwanuka may require a specific red water pot,

Kiwanuka's spear is red/brown,

Akakomo ka Kiwanuka kanywuufu

Song "*Kanjokye tulizimba endala kanjokye; Kiwanuka omulalu*, …"

Kiwanuka yawanuka enkuba – Kiwanuka is the one who causes it to rain

Is associated with red eyes when it has unmet requirements

Kiwanuka is associated with plants of red flowers

Enanda emyuufu is associated with Kiwanuka

Kiwanuka is associated with endiga emyuufu (brown sheep)

Kiwanuka asalirwa endiga emyuufu (brown sheep) *nga bukya* (at about 5.00 am)

Kiwanuka is associated with natural places of the mountains

Kiwanuka is associated with shrines.

Kiwanuka and Mukasa spirits, the sons of Kadduwannema, only collaborate with other spirits in offering healthcare but are not directly involved in offering healthcare

Musoke

Lubaale Musoke wa nsumbi. Munsumbi ya Musoke batekamu e jinja lye Nyanja

Musoke is a grandson of Mukasa

Omulimu gwa Musoke mpuliziganya – The role of Musoke is to do with communication especially among different spirits

Musoke mutonde – Musoke is a natural spirit not ancestral spirit

Musoke is associated with yellowish colour

Musoke requires few things; *ensumbi nga elimu ejjinja lyenyanja*,

Musoke is associated with plants of yellow flowers.

Musoke is associated with some specific songs.

Musoke tatela kusalirwa – Musoke is not normally associated with animal sacrifices. Musoke is normally associates with and benefits from rituals of Mukasa.

The association of Musoke with embuzi ya Luyina, a goat with a coloured bar beneath is a lie fabricated by people of these days.

Musoke awongerelwa Nsumbi nga elimu amazzi – *Nsumbi* containing water (lake or rain water).

Musoke abera kumazzi – Musoke is associated with water bodies

Musoke is associated with the rainbow

Nabuzaana

Empewo ya Nabuzaana egabulwa biwowoolo – Nabuzaana spirit is offered a communal meal made of lungs of an animal

Mizimu

Omuzimu gwenkongojja mulaguzi – The Muzimu for which I am a medium, divines

Omuzimu omukulu gwegunanyi Lubaale gensamira – My main Muzimu is the one which knows its Lubaale that it harmonised

Mayembe

Lubowa Jembe tonde, era Jembe Lubowa lyelikulira amanyembe gonna. Jembe Lubowa is a natural spirit and is head of all Mayembe. The other Mayembe are ancestral and man-made.

Amayembe malaguzi. Nze nkozesa a Mayembe Lubowa ne Kasajja okulagula – Mayembe can divine, for me I use Mayembe Lubowa and Kasajja to divine

Nina Amayembe – I have Mayembe

Amanyembe baselikary ba Lubaale, Mayembe are soldier spirits for Lubaale

amayembe bagula magule nebagassa mu Lubaale okukola emirimo ejenjawulo _ Mayembe are bought and introduced into Lubaale to do various duties

Nina ejembe Nambaga.

Nina ejembe Kiwanuka

Amayembe agaaleta olumbe agakwatiddwa gokyebwa oba galungamizibwa negakozesebwa mubulungi. (The Mayembe spirits that were responsible for the illness that are imprisoned are either destroyed by burning or they are rehabilitated and then utilised for the good)

Buli Jembe lisalirwa embuzi – an animal is sacrificed for each Jembe

Jembe Lubowa

Okuva edda nedda, Ejembe Lubowa telyalinyanga Mukazi, ebyo byanakuzino – since time immemorial Jembe Lubowa never possessed a female as its medium.

It is only these days that Jembe Lubowa can have a female as it medium

Mayembe work with Muwanga

Muwanga assigns duty to Mayembe

Mayembe work for Muwanga

Balongo (Twin and Twin forces)

Waliwo obulongo obutonde, obuzaale, n'obuwunde. There are twin forces that are natural, ancestral and the decorated.

Amanyi g'Obulongo obutonde gasinziira n'okweyolekera mubitonde nga ensozi, amayinja, enyanja, emigga, n'ebimera, Natural powers have their bases and are manifested in the mountains, rocks, massive water bodies, ocean/sea/lake, rivers and plants.

Obulongo obuzaale n'amanyi abwo byeyolekera mu bintu ebizaale nga enyanja Nalubaale, emigga Mayanja, Ssezzibwa, ebyewalula, n'ensolo nga oluse lw'engo. - Ancestral twins and twin forces were given birth to by human beings such as Nalubaale lake, Mayanja and Ssezzibwa rivers, reptiles and animals like the cat family.

Abalongo tebalagula – Twins spirits do not divine

Abalongo bebafuga Lubaale yena omuli e Misambwa na Mayembe – Twins are responsible for and control ancestral spirits including Misambwa and Mayembe

Abalongo bebalongoosa ensonga zona. - Twin spirits are known for purification all issues

Lubaale Mukasa na Balongo bawemulirwa nga bakola emikolo byaabwe – obscene language is used while performing rituals for Lubaale Mukasa and the twin forces

When a person produces a set of twins, and the rituals for the twins are not performed properly the parents of those twins end up in much troubles such as being put in Prisons, becoming very poor. The ritualistic meal by the family, *okwalula abalongo* for the twins include; *enkejje, empombo, obutiko obubaala, emere enyige, enyama, mayuni,* associated with music, drumming, local brew and use of obscene language (*ebigambo ebiwemula)*

Kitambo (Kinene Nyumba)

There are two types of Kitambo. *Waliwo e Kitambo ekilya kyebalogesa n'abaantu newabaawo e Kitambo Kinene Nyumba*

Rituals of animal throat-cutting for Kinene nyumba are performed at night - *Throat cutting rituals Kinene nyumba asalirwa kiro*

Kabaka

Kabaka azalibwa buzalibwa – Kabaka is a human born of a woman

Ndawula

Ndawula Mulangira era Ndawula Kabaka. – Ndawula is a royal but also a King

Ekitambo ekilya

Ekitambo ekilya kyekuusa nyo n'omusaayi olusi mubirooto

Ekitambo / Kinene Nyumba

Ekitambo Kinene Nyumba omuntu aloota abasezi, okuloota ebinya, okuloota abafu, oloota abaana, akuloosa ebyomubuto nga oddayo nga osoma kusomero. Oloota nga okozesa abakyala (sexually), assuula ebiggw ebyefananyiriza ensimbu,naye tovaamu musaayi and you cannot bite your tongue.

Kinene Nyumba aleeta olumbe olwefananyiriza ensimbu bwaba abaanja

Kinene Nyumba bwaba abanja nomuwa ensawo ye, notekawo ekibbo, nogula embuzi ye enzirugavu (black goat), nokola omulengejjo,

Kinene Nyumba can manifest as a Jembe, as a Musambwa, as Ddungu or as witchcraft. - *Kinene Nyumba afuuka a Jembe, afuuka omusambwa, afuuka Ddungu, afuuka eddogo*

Bulamu

Bulamu akola bwakiro – Spirit Bulamu works only at night

Sacred and natural places

Natural places

Munsozi mubaamu ekifo kya Mukasa

Shrine

Ancestral spirits normally base their activities in an empowered shrine.

The place where a shrine is to be build is identified and marked by a spirit possessing its medium. - *Empewo yelinya nelaga era eteeka laama awazimbibwa essabo ly'ayo.*

Empagi y'esabo esimbibwa empewo nga elinye kumutwe.– The central pillar of the shrine is positioned by the spirit possessing its medium

Omulya mere tasimba mpagi eyensonga mu sabo- A human being, if not possessed by spirit, does not position the central pillar of the shrine.

Buli Mbuga elimu ekifo kya Mukasa

Amasabo gakyuse nyo enakuzino, - The shrines have changed a lot these days

Amasabo agasinga tegakyali kumusinji gwa nnono - Most shrines are not rooted in the cultural and ancestral lineage.

Symbol and symbolism

Okuterekerela is the process or act of putting the symbolic artefacts for the spirits such as a basket, spear, bark-cloth, a bag or a fire place, -

Ekibbo (basket)

Ekibbo kibulula emikisa, - a basket is associated with reviving good luck

Ekibbo kibulula okuzaala abaana – a basket is associated with addressing infertility and bearing of children.

Olubugo (bark-cloth)

Olubugo (bark-cloth) is a symbol of ancestral spirits

Ensawo (a bag)

Ensawo (a bag) is a symbol of accumulating wealth. Ensawo is given to symbolise that one should save money and accumulate wealth. Some of the saved money is used to do spiritual rituals and ceremonies whenever the spirits so require.

Ekyooto (fire place)

This fire place provides warmth for my ancestors and all the ancestral spirits that need warmth, and the spirits in the wilderness.

Olumiri (Reed)

Olumuli/emmuli ziloga, wadde abantu balowooza nti zimulisa - Reeds are used for witchcraft, although most people think reads are used for brightening

Omuntu ataliiko Lubaale bwakozesa olumuli aba aziyiza ddogo n'ababbi – a person not possessed by ancestral spirit may use reeds to wade off witchcraft and evil people.

Ensaasi

Ensansi bwezinyenyezebwa zikowoola emisambwa.- The shaking of Ensansi and the unique sound produced invites the presence of ancestral spirits in the vicinity

White colour

Buli ekimela ekyelu guba muti gwa Mukasa – all plants white in nature are associated with Lubaale Mukasa.

The animals used for the Mukasa are all white in colour; white goat, white chicken, white cow,

Plants

There is a plant called Mukasa

Olweza plant lwa Mukasa - *Bweza bwa Mukasa ne Namukasa*

Amawasira ameeru ga Lubaale Mukasa - plants with white flowers are indicative of Lubaale Mukasa

Amawasira amaddugavu ga Jajja Bulamu – Plants with black flowers or leaves are indicative of Bulamu Spirit

Kawumpuli is the one associated with the Mukokowe plant,

Kiwampuka is not associated with Mukokowe plant.
