# Supplementary material for: Perspectives on health, illness, disease and management approaches among Baganda traditional spiritual healers in Central Uganda
Source: PLOS Glob Public Health. 2024 Sep 6;4(9):e0002453. doi: 10.1371/journal.pgph.0002453 (PMC11379289; doi:10.1371/journal.pgph.0002453)
Supplement: S12 Data — (PDF) [file pgph.0002453.s012.pdf]

Study participant 12 transcription

Contents

Study participant 12 transcription 1
Socio-demographics 2
Shrine 3
Sabo 3
Muwanga Shrine (Essabo lya Muwanga) 3
Other shrines 3
Lubiri 3
Bulubaale and Bulutansozi 3
Mulubaale 4
Health, Illness and Disease (Obulamu, Olumbe n’Obulwadde) 4
Obulamu 4
Olumbe 5
Charactaristics of Olumbe 5
Causes of Olumbe 5
Managemanent of Olumbe 6
Obulwadde 6
Characteristics of Obulwadde 6
Causes of Obulwadde 6
Management of Obulwadde 6
Health management 6
Diagnosis 6
Diagnostic tools 6
Omweso – 6
Offering healing 6
Good luck ritual 7
Human nature 7
Omwoyo – The Soul 7
Endowooza = thoughts /psychological 8
Spirits 8
Ancestral spirits (Lubaale) 8
Omuzimu 10

Kusumikirwa/kwambala/kuwongererwa ebifundikwa – Authoritative knotted dressing of Muzimu (Semusu photo)....10
Misambwa....11
Emisambwa Emirangira (Royal Misambwa)....11
Kawumpuli....11
Non-Royal Misambwa....11
Ddungu....11
Misambwa ejazalibwa abantu nga Misambwa....12
Abakyala be Misambwa (the wives of Male Misambwa)....12
Mayembe (Jjembe)....12
Jembe definition....12
Characteristics of Mayembe....13
Functions of Mayembe....13
How Mayembe work....13
Eddogo Witchcraft:....13
To manage witchcraft.....13
Management of witchcraft through negotiations....14
Management of witchcraft by use of medicines and rituals....14
Balongo....14
Tell me about your healthcare practice *(Mbulira ku nzijanjabayo)*....15
Tell me about Lubaale....15
Mukasa....15

Socio-demographics

My name is (name withdrawn). I am a married Muganda but my mother is a Musamya who migrated from Tanzania, I am a Mulangira, Male, 27 years old. My religion is traditional religion but I was a Protestant. I did not attend any school. I work as traditional healthcare spiritualist (*Ndi musawo wa kinansi*) using both natural and ancestral spirits, Muluntansozi (butonzi) and Mulubaale (buzaale). I work and stay in Kyaggwe County (Saza), Buikwe District, Nyenga sub-county, Kikwanya Parish Wanzyu – Namirembe village. I belong to Uganda N'eddagala Ly'ayo traditional healers' association.

twetaga emirembe, enkwatagana, obukulembeze obulungi, ekutegerebwa n'okukyikilirwa mu gavumenti eyawakati tubele namateeka amalungi getugoberela – We need peace, cooperation, good leadership, to be recognised and have representatives in central government so as to be orderly, have appropriate laws and be law abiding.

Shrine

I have two types of shrines. Sabo, the base for my ancestral spirits (obulubaale) and Lubiri for the natural spirits (Obulutansozi).

Sabo

Muwanga Shrine (Essabo lya Muwanga)

The following spirits use the same shrine, Muwangas'shrine

- Muwanga,
- Kawumpuli,
- Mukasa,
- Musoke,
- Kiwanuka,
- Kadduwannema and
- Mayembe.
  - it is worth to note that Mayembe do not have their own shrine, they use the same shrine with Lubaale spirits. So the original ancestral spirituality, Mayembe should never ask for a special shrine

Akasolya ke sabo lya Muwanga kalasibwa Mukasa, Kiwanuka nga bakozesa endiga emyuufu nga nume, embuzi njeru nume, enkoko mpanga nga nyeru, ne nkoko empanga nga ya lujuumba. – The rituals for the roof of Muwanga's shrine are done by Mukasa, Kiwanuka, using adult brown male sheep, white male goat, white male chicken, brown male chicken with black strips (lujumba omumyuufu)

Other shrines

The following spirits each has its own shrine

- Muzimu
- Ndawula
- Bulamu
- Ddungu (after hunting it settles in its shrine with all it has hunted)
- Bamweyana (gets his shrine – he is royal)
- Bachwezi (Kaliisa,)

Lubiri

Bulubaale and Bulutansozi

Nkozesa amanyi n'obuyinza ebyobulubaale n'obulutansozi kubanga obulubaale buyimiridewo ku butonzi"

I use powers and abilities of both the ancestral and natural spirits since the ancestral spirits have their base on nature. the powers and abilities of the ancestral are based upon the powers and abilities of the natural spirits. I cannot imagine a healthcare spiritualist without utilization of both natural and ancestral spirits. these powers support each other.

Omulutansozi naye asamira empewo zekika, kuba naye muzaale buzaazi. Nze natambuza ekitundu kyo buluntansozi n'ekyobulubaale era nsamira empewo zekika.

a Mulutansozi can also get possessed by ancestral spirits because s/he has ancestral origin. for me, I undertook the training as a Mulutansozi and I also trained as a Mulubaale, and I get possessed by ancestral spirits.

Newankubadde omntu asobola okuba nga asamira musambwa goka, nga akozesa mmusambwa, naye aba aliko empewo zekikaakye. However, an individual may have harmonized and utilize only one natural spirit (Musambwa), but even-then s/he has spirits of his/her ancestral origin.

Mulubaale

Omulubaale alengerera wala era ategeera mangu – a Mulubaale is far foresighted and understands very fast

Omuzimu bwe gweyoleka negwogera, gugogolwa n'empewo zagwo zona. – When the Muzimu appears and talks for itself, it is given a cleansing ritual with all its spirits.

Health, Illness and Disease (Obulamu, Olumbe n'Obulwadde)

Obulamu

Obulamu obulungi buli ku mwoyo owolubeelera – good health is a permanent experience of peace by the soul.

kunsi teli bulamu bulungi – There is no good health on while on earth.

Obulamu kunsi bwamubiri - Life on earth related to the physical and biological body

Obulamu obulungi bukolerera mwooyo, so simubiri – Good health is the work of the soul but not the physical body

Abagagga bangi tebasobola kwebaka, so nga nze omwavu nebaka nenegolora paka nkya. – Many rich people do not sleep, yet for me a poor man I get a comfortable sleep until morning

Obulamu obulungi kwekutukiriza nga olumirirwa abantu abalala. Nga omusawo, abalwadde bona, alina atalina mbakolako. Omuntu nga talina bbala bbi, nga talina kikolimo – obwo

bwebulamu obulungi. – Good health is to perfect and care for others, For a healthcare provider, is to care for all the rich and the poor alike, a person without bad taint and without curses.

Olumbe

Olumbe kintu ekitategerekeka, *amanyi agekizikiza, telunyonyolekeka* Olumbe is something very difficult to understand, powers of darkness and very difficult to explain.

Charactaristics of Olumbe

Causes of Olumbe

Olumbe is mainly caused by spirits and witchcraft.

Spirits causing *olumbe* are not all evil, some are good intentioned ancestral spirits using *olumbe* to inform, insist or institute punishment to the individual, at times for and on behalf of the family or community. The spirits may be demanding to be harmonised for family and community benefit and they institute *olumbe* to an individual or many family members. For example, I had a family where one of the female children refused to play her role as spiritual head because she was a dedicated Seventh Day Adventist (SDA). She was happily married to a rich man, but her family was rotting in poverty and multiple health related problems. During one family gathering, the family members pleaded with the spirits to instead turn their demands towards their sister who was blocking the spiritual harmonisation process because of her unwillingness to attend family spiritual gatherings nor play her ancestral roles. The spirits started sending signals of sickness, suffering and poverty to her. She was a successful poultry farmer. Suddenly most of her chicken got sick, drained her money in treatment but finally all the thousands of chickens died and she lost all her money. She took a loan to raise more chicken, which also died and left her debited with a loan. She become ill but her illness was confusing and could not be detected in the hospitals. Her concerned husband consulted with me and it was divined that her wife had denied her role as a spirit medium and a family spiritual head. She was aware but stubbornly refused the role. She was to suffer until she accepted her role or died before the spirits could chose another. The spirits would not opt for another person unless she is dead. That is how stubborn both the spirits and people can be.

Olumbe may be caused by witchcraft. A person may send witchcraft to another out of jealousy, malice, or in retaliation so as to cause bodily harm in form of illness and disease, or loss of job, business, money or property. For example, …………

Do you know how to cause witchcraft? If I know how to undo witchcraft, then I can do it, However, I do not do it because my ancestral spirits would punish me terribly if I did, unless the ancestral spirits agree to the justifiable cause. For example, *Omulundi gumu, nali nzijanjaba omukazi eyali alogebwa mujawe, nga omukazi tawona. Buli eddogo lyenzijawo nga oli ayongera eddala nga takoowa. Okutaasa ono nalina kuloga oli, era kyenakola nengatagata emiti, ne saddaka ye nkoko, nenamiriza nti gwe (name) lwolilekela awo okuloga mujjawo lwoliwona, nensindika eddogo nga ndi wano. Bweyatandika okwelwanako ono nawona*. One

time, I was treating a woman continuously bewitched by a co-wife, until I got fed-up. In order to save my client from death, I had to make and send witchcraft to the lady bewitching her. I combined peaces of plants, made a sacrifice of a male cock, instructed the witchcraft that until she stops bewitching her co-wife will she get better of the illness. My client soon got cured.

Managemanent of Olumbe

"Gwenzijanjaba olumbe silina kumukwatako, nina kulumba webakolera eddogo elyo nendijjawo omulwadde gyaali nawona". The physical presence of the client with *olumbe* may not be necessary, I just need to address that source of the problem for the patient to get better wherever s/he is.

Obulwadde

Characteristics of Obulwadde

Causes of Obulwadde

Management of Obulwadde

Health management

Diagnosis

Diagnostic tools

Omweso –

Nga omuntu yakatuuka, nga tanayogera kintu kyona nsoka kukuba mweso negundaga oba omuntu andi mumaso mukyamu oba mutuufu. (when a client is seated in front of me, I first use the diagnostic tool to find out if the client is a rightful person or not.

Offering healing

Buli muntu mujanjaba okusinziira ku kimuleese, kubujjajjabwe n'amanyi agamutuddemu, n'endagiriro genfuna okuva mubajjajjange – I offer personalised healthcare determined by the

client's concerns, his/her ancestral lineage and personal powers, and the guidance I receive *from my ancestral spirits*

Abazukulu bange mbawa eddagala eryo kunaaba, okunywa, okufuuwa mu miindi n'okunuusa. - I use herbs for clients in forms for bathing, drinking, smoking, sniffing,

Olusi nkozesa olulimi, ebigambo amanyi n'obuyinza okujjanjaba abalwadde bange – at times I use my tongue, words and the powers and abilities contained therein, to heal my clients.

Okugeza, bwemba nungamizibwa bulungi, nsobola okugamba bugambi omulwadde wange nti "genda owone" nagenda nawona olumbe nelumwamukako – For example, I am well guided, I may just say "go and get healed" the client will go and get healed of his/her illness.

omulwadde muwandako eddusu nawona olumbe – I use my saliva authoritatively to heal my clients

nkozesa emikolo okujjanjaba – I use rituals to offer healing

emikolo ejjijjanjaba gilina emisoso n'ennono y'aajo – Healing rituals have their particularities in form of requirements and process.

Some rituals involve drumming, singing and dancing from which some participants find relief.

Good luck ritual

Okugaba omukisa nkozesa amazzi ga Mukasa, enkoko enjeru n'ebigambo byemikisa nga nfukamidde wansi womuti gw'omusomba – For good luck to my clients, I use water described by ancestral spirit Mukasa, a white chicken and particular good-luck words while kneeling below a specific tree known as "Musoomba"

Human nature

Baganda were created in different clans

The clans have responsibilities over the human individuals within their clans

By nature, people are not aware why they were created.

Many people just do things without appropriate consultations

Many people do what they are not supposed to do and leave out what they are supposed to do

Every clan of Baganda has a share of the ancestral spirits and ancestral forces

Every clan in Baganda has ancestral healthcare spirits.

Omwoyo – The Soul

Omwoyo gugatta omuntu nomutonzi – Soul connects the human and the creator

Omwoyo guli mubantu boka – Omwoyo gwegufula omuntu omuntu – The soul is specific for humans. The soul is unique for humans only

Omuntu bwavaamu omwoyo afuuka mufu. – when a person loses the soul, it is a dead body

Omwoyo gwe muzimu – The soul is what is referred to as Muzimu

Omuzimu gukuvaamu nga offudde negukwata omulala. – *Muzimu* leaves the body when on dies and it can possess another person

Endowooza = thoughts /psychological

Obwongo = brain, mind,

Endowooza eyawukana n'obwongo - Thoughts are different from the mind or brain

Osobola okulowooza ekitasoboka! You can think of the impossible! You can think of picking a lorry of money on the roadside

Osobola okuba n'obwongo naye nga totegela. For example; when a man rapes a 2-year child. Such a man has the brain but lacks the understanding

Entegeera n'endowooza byawukana

Spirits

Muwanga and Mukasa are natural spirits

Nature does not differentiate between water and dryland spirits.

Muwanga can do his rituals both on water and dryland.

Spirits were created in none-physical forms and can not be touched.

Spirits were given some authority and power over other creatures

Some spirits can put together parts of nature and voices emerge out

Ancestral spirits (Lubaale)

Ancestral spirits practice healing using herbs to bathe, drink, smoke and sprinkling.

owalubaale tasobola kukwata Jembe. - owalubaale asobola okukuwa eddagala nolikansira mukifo ejjembe nelidduka.

Ancestral spirits belong to the clan and are hereditary, and much as they are knowledgeable and experienced, they can be misled.

Ancestral spirits select and prepare their spirit mediums. When ancestral spirits newly express themselves and use their new human medium, both the human medium and the ancestral spirits can be misled by their trainer (Senkulu) or any other malicious person,

Buli mpewo bwezebbulula, zibeera mumubiri mupya nomulembe mupya byezirina okuyiga n'okwetegereza. – whenever the ancestral spirits possess a new human spirit medium, they are in a new human new generation and environment both of which they need to know and learn. For example, the spirits which possess my grandfather one hundred years ago, when they possess my son, the spirits are in entirely new body and in a new generation with very different environment and ways of working which the spirits may need to learn and know. Likewise, my son has a lot to learn about the spirits and their various ways of working and preferences. Note that the spirit will have all its powers, abilities and authority but will need to be harmonised with the new human medium for appropriate working relationship within that clan. When the same spirit possesses another human medium in another clan, the same powers, abilities and authority may be used differently depending on the cultural background of that clan.

That is why the spirits normally participate in selecting their trainers (Senkulu), at various times of training their spirit mediums. The trainer (Senkulu) must be of particular background, belong to a specific clan, with known capabilities to offer to their human medium. Normally, the spirits are aware of what they want their mediums to learn from a particular trainer (Senkulu) for a specified duration, after which the spirit medium must move on.

Abakongozi baswaaza empewo, naddala bwebataba batendeke– the spirit medium often embarrass the ancestral spirits, especially when they are not trained. The spirits may be very powerful and authoritative but when the spirit medium is even not aware to the spirit s/he that possess him/her. S/he does not know the spirits requirements, its preferences, nor how to prepare for its coming, let alone how to call in the spirit.

Spirits may accept to be misled and they follow to allow their spirit medium to learn through the mistakes. Some spirit mediums are very careless, and the spirits make them suffer so as to learn through the consequence of their mistakes.

Spirits usually have reasons for the difficulties or problems they let their mediums experience.

Lubaale awangibwa ensawo y'olubugo, ekwatibwamu mukongozi ne kitaawe, bwaba yaliwo nga ewangibwa. Lubaale is given as empowered bag made of backcloth only handled by the spirit medium and his father, especially if he was present while making it.

Ensawo ya lubaale tekwatibwaamu mukazi – the inside of the Lubaale's backcloth bag should never be touched by a woman, not even other relative. That is why it is advisable to put the bag and sensitive spiritual acquisitions under strong lockable suitcase. When praying, the rest of the people can make their prayers and put money offerings into the twin baskets (*ebibo bya balongo*) but never in the Lubaale bag.

The accumulated money in ancestral bag can be used for ancestral rituals, buying spiritual requirement and materials. If the money is to be used for non-spiritual work, never borrow that money, but instead request humble yourself, explain in detail and request to use the money but not to borrow. Borrowing ancestral money is a big binding commitment that one may fail to fulfil and suffer the consequence instead. The money from the ancestral bag should not be used for burial related rituals, dating women, to buy fish, groundnuts, sweat-potato. However, the money from ancestral bag can be used to pay for treatment,

Omuzimu

Olunaku lwona nsobola obutafuna kasela kulya mere nga ndi bizze nyo, ndabira awo nga omulwadde azze omuzimu negulinya kumutwe negumujjanjaba – I may be very busy through the day and fail to have time to each food, and towards the end of the day a patient comes and the Muzimu possesses me and manages the client.

Omuntu bwafa afuuka omuzimu – When a person dies, his/her spirit turns into a Muzimu. Muzimu is central in traditional healing practices.

Muzimu can manifest itself as a Musambwa. Some Mizimu have characteristics of Misambwa

The ways of working of Mizimu and Misambwa are not much different

Muzimu and Kawumpuli greet by saying Gusinze

When the Muzimu is satisfied that it has been properly cleaned of any witchcraft and unnecessary attachments, it expresses itself and extends thanks to the spirit that has spearheaded the process. The process I witnessed had been spearheaded by Kawumpuli – the Prime Minister of all the spirits. It also expressed its gratitude to the spirit medium for being willing and brave and then appreciated all the family members for their contribution and participation. The Muzimu was asked what it required to follow. In response the Muzimu requested for its regalia (*Kusumikirwa bifundikwa*).

Kusumikirwa/kwambala/kuwongererwa ebifundikwa – Authoritative knotted dressing of Muzimu (Semusu photo)

The one being dressed was a male. There were three knotted backcloths. One of Muzimu, the second for its Lubaale and the third for its Mayembe. That is the knotted backcloth stood for Muzimu, Lubaale and Mayembe.

Ekifundikwa kyo Muzimu

While dressing the backcloth for Muzimu, the knots are positioned on the right side of the shoulders

Ekifundikwa kya Lubaale

Lubaale is understood from the concept of "*Luba*", the lower jaw, because Lubaale talks using the lower jaw.

Muwanga, Kawumpuli, Ndawula, Kadduwanema, Mukasa, Kiwanuka, Musoke all these are considered Lubaale – *Lubaale wenkuliiti*

While dressing the backcloth for Lubaale, the knots are positioned on the left side of the shoulders

Ekifundikwa ya Mayembe

While dressing the backcloth for Mayembe, the knots are positioned on the right side of the shoulders placed on top of that of Muzimu.

Misambwa

Misambwa girina amanyi mangi – Misambwa have much authoritative powers. *teri kintu kirina manyi mangi kusinga musambwa* – There is nothing as strong as Misambwa

Misambwa gikozesa nyo buyinza mukujanjaba okusinga eddagala. Misambwa mostly use authority in healing than use of herbs. *Asinziira waali nalaba ekiri ebunayira nasobola okukikwata nakijayo nakireeta –*

Misambwa emitonde gikozesa buyinza – Natural Misambwa use authority for healing

Emisambwa eginsinga mizaale naye egimu, naddala emirangira, gitambilira mumanyi agobutoonzi – Most Misambwa are humanly but some few, especially the royal spirits work with natural powers.

Emisambwa Emirangira (Royal Misambwa)

Emisambwa emilangira mu nnono ya Lubaale include Kawumpuli, Bamweyana, Ndawula - The royal Misambwa within norms of Lubaale include Kawumpuli, Bamweyana, Ndawula,

Kawumpuli

Kawumpuli is a royal and royals are not restricted to any particular clan in Buganda. *Kawumpuli musambwa musange – muzaale*. Kawumpuli was a royal but deformed child whose father is Sekabaka Kayemba, the 9th King of Buganda and his mother was Nakku from Fumbe Clan.

Kawumpuli was a Buganda royal who was raised in Bunyoro, grew up, died and his physical body buried in Bunyoro. Kawumpuli was born as a twin (deformed) but with special spiritual powers. Kawumpuli parents feared to associate with the deformed child in the public and was abandoned in the bush by his mother Nakku with the knowledge of his father King of Buganda Kabaka Kayemba. Kawumpuli was picked from the bush by a lady from Bunyoro called Nabuzaana who become his guardian mother and raised him from Bunyoro. Kawumpuli started divining while still a child.

The health pandemic challenge, the plague that hit Buganda that time was attributed to Kawumpuli and even named after him. Plague is called Kawumpuli. Spiritually Kawumpuli was brought came back to Buganda as a Musambwa, and was very influential in Buganda spirituality to the extent that his spirit was given extra-ordinary powers over all the spirits in Buganda. He became the Prime Minister of Buganda spirits *(Katikiro we mpewo mu Buganda)*

Kawumpuli, on coming back to Buganda as a spirit, he come with his guardian mother Nabuzaana as a spirit who was confirmed and honored as a mother of all children (Nakazadde)

Non-Royal Misambwa

The non-royal Misambwa within norms of Lubaale include Mukasa, Kiwanuka, Musoke, Kadduwanema, Kaliisa, Nabuzaana, Nakayima, Ddungu,

Ddungu

Ddungu spirit was formally called Mpoobe while still a human being. Mpoobe (Ddungu) was a hunter. One time his hunt (fumbe) run into an anti-hill (*ekiswa*) which normally is a house

for Musambwa (Kabaka Bulamu), upon explanation of what is was doing to Kabaka Bulamu, the Kabaka gave him permission to hunt everywhere (*Ddungu lyona balimuwa okuyigirayo)*. Ddungu never used dogs in his hunting, he only used spear and was very accurate in his targets using his spear. Ddungu would rarely miss his target even at a good distance. However, those he used to go with for hunting like Kayizzi and Kanoonya had dogs they used for hunting. That is how Ddungu become associated with dogs and hunting net.

Services of Ddungu spirit are usually required when the clients require money, riches and other material things.

Misambwa ejazalibwa abantu nga Misambwa

Muyanja Musanbwa muzaale naye si Lubaale - Mayanja is a Musambwa birthed by a human being but is not part of Lubaale.

Emigga Mayanja ne Ssezibwa Misambwa muzaale naye gutambulira mu butonzi era gilina amanyi manji – Mayanja and Ssezibwa rivers are Misambwa birthed by human beings but with lots of natural powers

Ancestral Misambwa use herbs for healing - *Misambwa emizaale gikozesa ddagala*

Abakyala be Misambwa (the wives of Male Misambwa)

Mayembe (Jjembe)

Mayembe stand in for Misambwa and Mizimu

Mayembe carry out duties assigned to them by Misambwa and Mizimu

"Mayembe kalabalaba we Misambwa ne Mizimu mumirimu ne nkola gyaabyo"

Sub-types of Mayembe; *jjembe ery'omubbanga (Bagalawo), jjembe ly'omulukaayi wagulu, ne jjembe elyo kumutwe*

Mayembe are free and open in their talking and working

By their nature, Mayembe follow Misambwa, Mizimu and other spirits - "*obuwange bwa Mayembe kulondoola mpewo"*

Mayembe greet by saying Ndala ndala

Mayembe si Lubaale – Mayembe are not Lubaale

Jembe definition

Characteristics of Mayembe

Jembe is very fast, acts fast, and does as soon as possible, provided it is paid for the work

Jembe use lots of force and wants money very much

Functions of Mayembe

How Mayembe work

Ejjembe lyesitukira neligenda mukifo woyagala nelikola omulimu, nekubulira ebiriyo

Ejjembe likwata kungulu, tekikwata mumubiri, era osobola okulikwata nokijjawo omuntu nawonerawo. The effects of a jembe are usually superficial on the human body, and if handled and removed within a short period, it may leave no physical or biological effects on the body.

"lumu bandetera omukazi nga bamusindikira e jjembe nga mulwadde muyi, tayogera. ejjembe nendikwaata nendijawo nafukirawo omulamu nokwogera noyogera bulungi natambula nadda awaka nga mulamu katebule" One time, a severely ill lady, unable to talk due to a Jembe spirit sent to attack her. I caught and removed the Jembe from her, and she immediately improved, started speaking and she finally went home in good health.

Kifaaru - *omuntu atali musamize bw'afa omuzimugwe gubonabona era ejjo emizimu egibonabona nga jiyenjeela tusobola okugikunganya ne tugitekamu emiti ejenjawulo netubisalira saddaaka, netulamiriza, netugituma okutukiriza amanya g'oyo n'ekyetwagala okukola oba okubeerawo. Okugeza; Okutta; enfa y'ona gyetulamirizza; accident, kufa kikutuko, affe nga atambula,*

Eddogo Witchcraft:

To manage witchcraft.

Witchcraft is managed through various ways that may include negotiations, use of medicines and rituals or use of force.

Witchcraft brought about by spirits is easier to handle than witchcraft use of materials such as plants, animal parts, or other natural materials.

Management of witchcraft through negotiations

To manage witchcraft due to spirits, you may need just to negotiate with the spirits, justify why the attacked individual was innocent, incur the cost of what was given to send the spirits and pay for sending the spirits back to the sender or to the wild.

Omusajja bamusindikira amayembe okumutta olwokumutebereza okwenda ku mukazi wa mukwano gwe, nebamundetera nga ebintu bimutuga ataawa. Amayembe nakiriziganya nago nengazza wabbali tumale okwekanya ensonga. Amayembe bwegamuvaka yatewulukuka natelera, nemubuza ambulire amazima ku byamanyi kumukazi wamukwanogwe. yabyegana ela bwetwanonyereza twakizuula nga omukazi yali tamwagalangako. Amayembe ng anago gamatidde, nakiriziganya nago, nengawa engassi, gaddayo omusajja nawona. A man was brought to my shrine gasping for air due to an attack by Mayembe spirits because he had been suspected of have a sexual affair with the wife of his friend. I negotiated with the spirits to get aside so as to establish the truth. I told the man to tell me the truth about his relationship with the wife of his friend, he denied any sexual relationship with her. Together with the Mayembe we investigated and established the truth that he was innocent. I reimbursed the expensed, paid the Mayembe for them to go back. That is how I healed the man.

Eddogo lyeddagala zibu okujjanjaba. Eddogo lyeddagala lisooka kubuna ne lisasaana omubili gwona. Era kitwala ebanga okujanjaba omubiri okuwona. Witchcraft due to use of natural materials such as plants, animals' parts, rocks etc., is harder to manage. Witchcraft due to herbal materials spreads in the whole body and it takes some good time to remove it and treat the body.

Management of witchcraft by use of medicines and rituals

Healing of illness and diseases caused by witchcraft by use of medicine and associated rituals is a much slower process and is the best when the witchcraft has taken long in the physical body and has affected the biological processes of the body.

Okujjanjaba eddogo, osooka kulikunganya mumubiri, noliseetula nolijja mubifo ebyobulabe nga omutima, nolisengulira mubifo ebyomubiri ebirara nga okugulu. Olwo nogenda nowangulula byebawanga muddogo. To manage illness due to witchcraft, I first arrest its spreading and localise in less vital, and more muscular areas of the body such as limbs, before I dismantle or disable the witchcraft.

Balongo

Twin forces mostly use Nalongo for healing. Twins are always the first to address in healing. There is a saying *"abalongo kyebasiba teri asumulura ate kyebasumuluma teli asiba"*

Twins are very strong entities. There are natural and ancestral twins. The natural twins and twin forces descended in particular natural places where they can be accessed.

The powers of the Baganda Kings are embedded in their twins. Kings of Buganda has twin sets whose powers and guidance help the king to rule at least for thirty years. The natural twin sets for Kabaka Muteesa II were in the grass-hopper and a snake (*Janzi no Musota*)

Abalongo tebetika – nothing is placed on top of the basket for twins

Twins and twin forces are a responsibility of everyone and communicate through messages contained in dreams by various individuals

Twins and twin-forces need to, at times forcefully demand to be cared for or attended to.

Nanyini baana yeyabagya mumpompogoma – Twin and twin-forces are owned by the individual who derived from very difficult conditions or circumstances

Tell me about your healthcare practice *(Mbulira ku nzijanjabayo)*

Ezijanjaba yange esinziira ku ekirese omulwadde, byenzudde, n'okulungamizibwa kwempewo – abasinga baja banonya Mukisa, kujjawo ddogo, Kwambululwa, mirimu, mikwano, lumbe oba obulwadde. My way of working is determined by what the client is seeking for, my findings and the spiritual guidance I receive. Most clients come seeking for Luck, removing witchcraft, cleansing, jobs, relationships, illness and diseases

There is no fixed working process or management of a health situation. There are many variables in the process and I am spiritually guided based on these many variables, so there are many ways of solving the same problem within individuals.

Tell me about Lubaale

Lubaale is a general terminology among Baganda for ancestral spirituality, with categories whose details vary within the clans. The major categories include Lubaale wenyanja omuli Musoke, Kiwanuka ne Mukasa, Misambwa, Mizimu, Mayembe, na Balongo,

Some categories of Lubaale such as Balongo and Mizimu cannot stand alone in healing practices, instead they work while in collaboration with Misambwa, and Mayembe.

Mukasa

Mukasa ye nanyini Lubaale - Mukasa is the owner of Lubaale

Mukasa ya layirira Lubaale era ya mukombera kunsimu – Mukasa is the one who swears on behalf of Lubaale is Mukasa is the spirit which bears the last, most strong test for Lubaale spirits – licking of the red-hot iron with the tongue.

Mukasa yeyemirira Lubaale - Mukasa stands in for Lubaale in most issues relating to Lubaale.

Mukasa ye taata (Salongo) wa baana mu Lubaale – Mukasa is the father of twins in Lubaale

Mukasa mujanjabi mu Lubaale – Mukasa is a healer ancestral spirit.

Mukasa yakakasa ensonga zona mu Lubaale – Mukasa is the spirit that puts a confirmatory stamp on all issues regarding Lubaale and its activities.

Mukasa yagaba obweeza (emikisa) – Mukasa is the spirit that gives/ bestowers good luck

Mukasa yeyeyimirira Lubaale munsonga ezisinga obungi – Mukasa stands in for Lubaale most of the cases.
