# Supplementary material for: Perspectives on health, illness, disease and management approaches among Baganda traditional spiritual healers in Central Uganda
Source: PLOS Glob Public Health. 2024 Sep 6;4(9):e0002453. doi: 10.1371/journal.pgph.0002453 (PMC11379289; doi:10.1371/journal.pgph.0002453)
Supplement: Attachment — Submitted filename: PGPH-D-23-01744 with sticky notes.pdf [file pgph.0002453.s014.pdf]

PLOS Global Public Health

Perspectives on health, illness, disease and management approaches among traditional health care spiritualists in Central Uganda.

--Manuscript Draft--

**Manuscript Number:**	PGPH-D-23-01744
**Article Type:**	Research Article
**Full Title:**	Perspectives on health, illness, disease and management approaches among traditional health care spiritualists in Central Uganda.
**Short Title:**	Baganda spiritualists' perspectives on health, illness and disease
**Corresponding Author:**	Yahaya Hills Kagali Sekagya, MPH<br>Mbarara University of Science and Technology<br>Mbarara, UGANDA
**Order of Authors:**	Yahaya Hills Kagali Sekagya, BDS, MPH, Dip (Law)
	Charles Muchunguzi, BA (SS), PGD M.A, PhD
	Payyappallimana Unnikrishnan, BAMS, MA, PHD
	Edgar M. Mulogo, BDS, MPH, MSc., PhD
**Keywords:**	Spiritualists (Balubaale); Health; Illness; disease; qualitative; Baganda, Buganda
**Abstract:**	In Uganda, spirituality is closely associated with traditional healthcare, however though prevalent, is considered controversial, mystical, less-documented and is often misunderstood. There is a paucity of literature on the description of health, illness, disease, management approaches among spiritual traditional healthcare practitioners. This article examines perspectives on health, illness, disease and management approaches among traditional health care spiritualists in central Uganda.<br>Balubaale are Baganda health care spiritualists engaging ancestral spirits in health care and management. The study used a qualitative study design in particular grounded theory. Unstructured, qualitative interviews and observation were used on 12 male and female purposively selected Balubaale in Central Uganda. Data was transcribed, coded and thematically analyzed using ATLAS ti. 22 Computer software based on inductive approach.<br>Findings show that the word and concepts describing health, illness, disease and management approaches are descriptive and contextualized in the nature of the problem, the prospected root-causes, and therapeutic approaches involved. The words for illness “Olumbe”, disease “obulwadde” and management approaches divination (kulagula), ritual cleansing (Kwambulula), amulets (Ensiriba and Yirizi), and Scarification (Kusandaga) have spiritual and social dimensions, contextual meanings and attachments.<br>Further research must be done among other ethnic groups to compare findings and terminologies to facilitate communication and policy consideration
**Additional Information:**	
**Question**	**Response**
**Financial Disclosure**<br><br>Enter a financial disclosure statement that describes the sources of funding for the work included in this submission and the role the funder(s) played. This includes grants and any commercial funding of the work or authors.	World Bank grant to PHARMBIOTRAC of Mbarara University of Science and Technology (ACE II) funded the first author PhD study.<br>Funders did not play any role in the manuscript preparation nor submission

Powered by Editorial Manager® and ProduXion Manager® from Aries Systems Corporation

This statement will be typeset if the manuscript is accepted for publication.<br><br>*Please review the submission guidelines and the instructions link below for detailed requirements and guidance.*	
**Competing Interests**<br><br>On behalf of all authors, disclose any competing interests that could be perceived to bias this work.<br><br>This statement will be typeset if the manuscript is accepted for publication.<br><br>*Please review the instructions link below and PLOS Global Public Health's competing interests policy to determine what information must be disclosed at submission.*	On behalf of all authors, I declare that we have no competing interests
**Data Availability**<br><br>Before publication, Authors are required to make fully available and without restriction all data underlying their findings. Please see our PLOS Data Policy page for detailed information on this policy.<br><br>A **Data Availability Statement**, detailing where the data can be accessed, is required at first submission. Insert your Data Availability Statement in the box below.<br><br>Please see the data reporting section of our submission guidelines for instructions on what you need to include in your Data Availability Statement.<br><br>This statement will be typeset if the	Data is available and is attached.

manuscript is accepted for publication.<br><br>*PLOS allows rare exemptions to address legal and ethical concerns. If you have legal or ethical restrictions, please use the box below to detail these in full sentences for the Journal team to consider.*	

Manuscript Click here to access/download;Manuscript;Perspectives on Health Ilness, disease.docx

Perspectives on health, illness, disease and management approaches among traditional health care spiritualists in Central Uganda.

Yahaya H. K. Sekagya[1, 2,*], Charles Muchunguzi[3], Payyappallimana Unnikrishnan[4] and Edgar M. Mulogo[5]

[1] Department of Pharmacy, Mbarara University of Science and Technology, Mbarara, Uganda

[2] Research and Training department, Dr. Sekagya Institute of Traditional Medicine, Uganda

[3] Department of Environment and Livelihoods Support Systems, Mbarara University of Science and Technology, Mbarara, Uganda

[4] Professor, University of Transdisciplinary Health Sciences and Technology, Bengaluru, India

[5] Department of Community Health, Mbarara University of Science and Technology, Mbarara, Uganda

Yahaya H. K. Sekagya (ORCID iD) 0000-0001-9721-7158

We have no known conflict of interest to disclose

* Yahaya Hills Kagali Sekagya, E-mail; ysekagya@gmail.com (YHKS)

Abstract

In Uganda, spirituality is closely associated with traditional healthcare, however though prevalent, is considered controversial, mystical, less-documented and is often misunderstood. There is a paucity of literature on the description of health, illness, disease, management approaches among spiritual traditional healthcare practitioners. This article examines perspectives on health, illness, disease and management approaches among traditional health care spiritualists in central Uganda.

Balubaale are Baganda health care spiritualists engaging ancestral spirits in health care and management. The study used a qualitative study design in particular grounded theory. Unstructured, qualitative interviews and observation were used on 12 male and female purposively selected *Balubaale* in Central Uganda. Data was transcribed, coded and thematically analyzed using ATLAS ti. 22 Computer software based on inductive approach.

Findings show that the word and concepts describing health, illness, disease and management approaches are descriptive and contextualized in the nature of the problem, the prospected root-causes, and therapeutic approaches involved. The words for illness "*Olumbe", ,* disease *"obulwadde"* and management approaches divination (*kulagula)*, ritual cleansing (*Kwambulula*), amulets (*Ensiriba* and *Yirizi)*, and Scarification (*Kusandaga)* have spiritual and social dimensions, contextual meanings and attachments.

Further research must be done among other ethnic groups to compare findings and terminologies to facilitate communication and policy considerations.

Keywords: Spiritualists (*Balubaale*), health, illness, disease, qualitative, Baganda, Buganda

Introduction

Traditional Health Care Spiritualists (THCS) are indigenous healers who believe in spiritual guides and use these spiritual guides during health management (1–5). The worldviews of THCS regarding health, illness, disease, and their management approaches are rooted in local cultures and traditions, and are uncommon in literature (6). This paucity was contributed to by the colonizing ideology that branded indigenous people as “primitive” and their knowledge systems rendered obsolete, irrelevant, and worthless (4,7). Subsequently their descriptive views of health, illness and disease were unsuitable for the “Modern” world that emphasized measurable power, data and words (7). In addition, the introduced education curricula banned people using their local languages, cultures, ritual and all spiritual things, and instituted numerous unethical punitive laws and regulations (8). This deadly knowledge apartheid was fueled by historical humiliation and deep mass resentment (9). Hoppers and Sandgren suggested an equitable dialogue between academy and indigenous knowledge holders (10). These should then inform the social contracts that underpin the different understandings of knowledge within the diverse settings of global societies (11). True knowledge and wisdom are better expressed and communicated as first-hand personal experiences in the vernacular knowledge of their places (12,13).

Traditional health care spiritualists have had multiple accusations and have lost pride and confidence in use of their local descriptive words for health, illness, disease and health management approaches (14). Nonetheless, traditional medicine is still valid and is used worldwide (15,16), by 42% Americans, 48% Australians, 70% Canadians, and Germans (17). Health management in context of people’s traditions and customs is encouraged since its existence will determine the basis for its acceptance, criticism or rejection (18,19).

Even though scientific disciplines of Sociology and Philosophy of medicine have their own concepts of health, illness and disease (20–23), it is not logical/valid to challenge

indigenous research to uncover the traditional logic and methods in relation to concepts with mainstream scientific equivalent. Instead the insistence should be for traditional knowledge to continue research without undermining its knowledge nor reduce its holistic cultural values (24,25). However, the challenge remains for policy formulation where use of local descriptions for health, illness, disease and health management may reflect divergent meanings in contemporary science (26,27).

Although, nearly 80% populations in developing countries use traditional medicine (18,28), it should be noted that indigenous health care spiritualists and their use of local descriptive words during health management are not acknowledged (6,29). However, there are reported efforts to revitalize indigenous health systems towards achievement of sustainable development goals three (SDG3) to achieve universal health coverage (UHC) (30,31). Unfortunately, most efforts have been directed towards understanding herbal medicine from biomedical perspectives, consequently excluding spirituality and the totality of traditional medicine from mainstream health care options (14,32,33). Other related studies focus on perception of illness by patients (34–39), and exclude the perceptions of the indigenous health care spiritualists. Some authors argue for intra-cultural learning to understand and appreciate culturally embedded worldviews (29,40), and attached meanings.

Often traditional health care spiritualists may sound superstitious, and is usually only understood by communities of health care spiritualists. Their health care practices, understanding and descriptions of health, illness, disease need to be explored further and explained to global audience even when scientific explanations justifying their clinical practices may be missing (41). The Uganda national policy guidelines on traditional medicine recommended that it is important to understand and build consensus about basic definitions, terminologies and classifications of health, illnesses and diseases by various sub-categories of traditional medicine so as to promote information sharing, policy formulations and research

methodologies (42). This would enable conceptualization of spiritual health care practice in various cultures. Despite the national recommendations and policies, there is a paucity of literature that describe health, illness, disease and management approaches as understood by traditional health care spiritualists in Uganda. The purpose of the study was to explore the descriptions of health, illness, disease and health management among Baganda traditional health care spiritualists in Central Uganda.

Method

Study Design and Setting

A qualitative and exploratory research design using ethnographic approach was used to explore terminologies that described health, illness, disease and for health management practices among *Balubaale* in Central Uganda. Uganda area covers 241.555 km^2 and is divided into four regions namely Eastern, Western, Northen and Central. It has 156 districts and a population of 44.2 million people of which 88.6% are in rural areas and subsistence farmers (43). ~~Central region, the focal area of the study, is Buganda, comprised of 27 districts.~~

Sampling and Data Collection

We identified participants through their governing associations (NACOTHA, Uganda N'eddagala Ly'ayo, Uganda N'eddagala N'obuwangwa Bwaffe) by their leaders (45). Additional identification was done by district and cultural leaders. We approached the Healthcare spiritualists at their work places (shrines) and invited them to participate. Those who agreed and fulfilled the inclusion criteria and signed ~~an~~ informed consent were recruited for the study between 15th July 2019 and 29th April 2020. The inclusion criterion of being a spirit medium for Muwanga affected the need to balance the male and female representation. Anybody below 18 years were excluded. Twelve (10M, 2F) adult healthcare spiritualists (*Balubaale)* residing and working in Central Uganda were purposively selected (44), based on their background knowledge and at least 10 years of experience. Selected participants were mediums for ancestral spirits including spirit Muwanga. Related studies successfully used small numbers of participants and retained validity in their studies (45,46). Data was collected through unstructured interviews using open-ended questions and partial integration observation methods. The first author closely worked with the study participants at their work

places for two years and participated in some daily health care activities. He interviewed the practitioners as appropriate which lessened interference with their usual practices.

Data management and analysis

The data was transcribed by the researcher and research assistants directly into English while maintaining depth of context in Luganda, the local language of spiritualists. Transcribed data was coded and thematically analyzed using ATLAS ti. 22 Computer software based on inductive approach.

Ethics consideration

Ethical approval of this study was sought and obtained from the Research and Ethics Committee at Mbarara University of Science and Technology (No. MUREC 1/7) and the Uganda National Council of Science and Technology (SS 4947). The study was also cleared by Traditional Healers' Associations (NACO/0485/2019) and Buganda Kingdom and the Office of the President (ADM 194/212/01). Written informed consent was also obtained from the individual subjects.

Findings

Socio-demographic characteristics of respondents

Study participants were twelve (10 Male, 2 Female) *Balubaale*, that is Baganda traditional health care spiritualists using ancestral spirits in health management. They were aged between 27-77 years (Mean age 54), from eight (8) Buganda Counties, eleven (11) Districts, and nine (9) Baganda clans. Majority (66%, n=8) belonged to traditional religion. Two (2) never attended school, six (6) never completed primary level and four (4) entered secondary level. All respondents belonged to healers' associations, had above ten (10) years of experience and were subsistence farmers.

Description of health, illness and disease among spiritualists

Concepts that described health, illness, and disease among *Balubaale* were embedded in words and phrases used during the various health management approaches. Research findings were based on responses and observed communications of spiritualists while in normal states and as spirits possessed them during health care practices. Most words did not have English equivalent in mainstream medicine. They were left in Luganda local language and only given nearest equivalent to the root idea. The words imitated descriptive root causes, therapeutic approaches, and or associated ancestral spirits. Other words were directly connected with natural materials used during health management such as plants, animals, birds and water sources and were influenced by the cultural settings.

Words and phrases used to describe good health varied depending on the state of the spiritualists. While in their normal states, spiritualists used common words and phrases for good health, but when possessed by ancestral spirits as spirit mediums, the words become more complicated. Spirits used words for good health during greetings at first expression of

their presence. However, for illness and diseases, the words used by the spiritualists were the same in either state and ritualistic in nature describing health management approaches.

Perspectives on Good Health

The phrase used to describe good health by the spiritualists while in their normal states was "*Obulamu obulungi*". However, when possessed by ancestral spirits they used a word "*Bweeza*". According to the research findings *Obulamu obulungi* (Good health) is a complex concept that is bi-dimensional that focuses on physical health and nonphysical health.

Physical and Psychological health

Good health in relation to Physical and Psychological health include words that refer to the physical body (*omubiri*), mental (*obwongo*), psychic (*ebirowozo*), and behavior (*empisa*). The spiritualists expressed that much of the manifestations in the physical and psychological reality were mainly at the non-physical level, where they concentrated more.

Nonphysical health

Obulamu obulungi (Good health) goes beyond describing the desirable human condition when one's bodily physical health is well. It also focuses on non-physical self, such as the state of the soul and the good relations with ancestry. For example, one participant said that *"Good health includes aspects of the body, spirit, mind, moral, soul"* (Participant 4). Furthermore, *obulamu obulungi* also focuses on the affordability of basic human needs such as food and shelter for individuals and their family members. Good health was also associated with success in one's endeavors, happiness, joy, stable family, having children and good friends. For example, *"... good health is when a person can afford basic needs like food and housing for self and family"* (Participant 5). The participants made it clear that good health does not mean that one did not get ill or have disease, but one has the ability to timely access appropriate health care to address their biomedical, social, cultural, mental and

spiritual causes of their undesired health. According to the participants, good health was also contributed to by good luck (*omukisa*), which participants described as natural (*omukisa omuzaale*) or acquired through use of rituals and local medications (*omukisa omuywere eddagala*). One participant said *“Once the needful is done for the ancestral spirits, good luck opens up and everything gets on very well"* (Participant 2).

“*Bweeza”* on the other hand was frequently used by the spiritualists, spirit mediums and spirits for good health during a trance. Bweeza is usually aligned with good health and the inner peace that it comes with. For example, *"Bweeza is a summary word for good health."* (Participant 4) and *“Bweeza means and is associated with peace."* (Participant 9). The term is usually related with the spirit Mukasa which brings good health. So, when one says “*Bweeza”* and the people around would jointly reply *“Bweeza bwa Mukasa”* in agreement that good health, good luck and peace were from ancestral spirits especially spirit Mukasa. *Bweeza* was also associated with blessings, fertility, bearing of twins and rains.

Perspectives on illness (*olumbe*) and disease (*obulwade)*

Illness was referred to as *olumbe.* Research participants described *olumbe* as an unclear and unnamed bad health condition that often occurs suddenly and has many possible root causes. In addition, the person who suffers from *olumbe* sometimes has a number of complicated medical conditions which are often not cured by mainstream medical approaches. For example, one participant said; *"Illness normally occurs suddenly, a person is just attacked by undefined bad health conditions"* (Participant 9). Similarly, another participant said; *"Olumbe is something very difficult to understand, is associated with powers of darkness and very difficult to explain."* (Participant 12)

Furthermore, participants described *olumbe* in relation to its prospected root-causes, majorly the ancestral spirits (*Lubaale) and witchcraft (ddogo). Lubaale* was considered responsible for causing *olumbe* especially when the ancestral spirits have unfinished business

with the person or family. When there are unmet demands, *olumbe* manifests in the physical body, psych and social status of an individual or family. The participant continued to explain that o*lumbe* due to ancestral spirits may spread into the whole body before it manifests, has hereditary tendencies, and takes a while to treat. One participant said; *"Olumbe attacks the soul and spirit, take long to be noticed, and may as well be inherited."* (Participant 5). A supplementary statement by another participant *"… madness due to ancestral spirits responds to rituals but not drugs."* (Participant 10).

Nearly all illnesses were attributed to witchcraft. Witchcraft was used to describe invisible, spiritual, or physical manifestation of illness caused by an individual towards another, using words and materials with spiritual powers, usually out of jealousy. One participant explained that *"illness may be caused by witchcraft sent by another person out of jealousy."* (Participant 12). *Olumbe* due to witchcraft may affect an individual and the whole family, and if it results in death, the individual dies with it, and such o*lumbe* is often inheritable, unless the needful is resolved. One participant said *"a person dies with olumbe and olumbe is inheritable"* (Participant 5). Among Baganda, o*lumbe* is expelled out of the family by performing ritual of the last funeral rites after someone has died. A participant explained that; *"In Baganda culture, olumbe is expelled out of a family by the last funeral rites."* (Participant 4).

On the other hand, the study participants did not express any singular word for disease, but a multitude of signs and symptoms that manifested on the physical and biological body parts such as fever, pain and vomiting. One participant said; *"There seems not to be a single Luganda word that properly describes disease."* (Participant 4). Another participant explained that *"Obulwadde is a combination of obvious signs and symptoms, such as swellings that pain."* (Participant 2). *Obulwadde* takes on the name of the affected body parts. A participant clarified that *"Disease affect the body parts or organs and manifest as*

symptoms of pain in the head, eyes, nose, teeth, liver, kidneys, intestines, heart, lungs, legs, arms, etc." (Participant 3). In other words, *obulwadde* was not connected to any root-causes nor any spirits.

Differences between illness (*olumbe*) and disease (*obulwadde*)

For the majority of the participants there was a clear distinction between illness and disease in terms of causes, effects, and the management processes. During health assessment, illness (*olumbe*) was connected to ancestral spirits and witchcraft as root causes, while diseases (*obulwadde*) were attributed to causes of organisms such as bacteria and worms, and/or physiological malfunctions of the related organs. For the effects, illness (*olumbe*) majorly affected the non-biological parts of human, like the soul and spirit while the disease (*obulwadde*) affected the biological body organs and their functions. For example *"illness only engulfs the body but does not enter into the body organs while the disease attacks and destroys the body organs"* (Participant 3)

The management process for illness (*olumbe*) was mainly through rituals engaging use of energies from living and non-living things, symbols and communication with ancestrors to prevent and/or remove the causative agent and protect against other attacks. Participants reported that Illness (*Olumbe*) is not cured by biomedical treatment, but gets worse instead. Yet the management of disease (*obulwadde*) attacks to kill offending macro/micro organism and restore the function of the affected organs through treatment using herbs and physical manipulations. For example; *" illness is when the disease gets worse and fails to respond to biomedical treatment and finally leads to death. That is why the last funeral rites are performed to exit the illness out of the deceased's house" This means that it is the olumbe which actually finally kills a person."* (Participant 10)

Although majority of participants could distinguish between the concepts of illness and disease, some participants did not find it important to differentiate between these

concepts of illness and disease, and they were considered the same. For example, one participant said *"there is no difference between illness and disease, illness and disease are the same."* (Participant 10). Similarly, another participant said *"Illness and disease refer to the same thing, they are the same.* (Participant 11).

Study participants reported that they used particular words for health assessment and health management depending on context. It was reported that these words and phrases are imbued with powers and authority of the spiritualists, and were potentiated by use of specific rituals, prayers and sacrifices, which were, at times, carried out in specified sacred places.

Different words that are used by the spiritualists during health assessment and health management refer to the different aspects of the process. Some of these words cut across the different domains of prevention, protection, treatment, and health promotion depending on context. The words used are descriptive of prospected cause of illness or disease, purpose and methods or approaches used for prevention, protection, treatment, and health promotion. To best understand these concepts well we have subdivided into two sections that is words used in health assessment process and words used in the health management process. Words used for the health assessment include *kulagula (*divine/diagnosis*),* and *kwaaza Lubaale* (cultural ritual process of investigating ancestral spirits).

Divination (*Kulagula*)

According to the research participants, divination (*Kulagula*) describes a cultural process of health assessment and diagnosis among *Balubaale.* It usually focuses on assessment and diagnosing of illness, rather that disease. During divination, the spiritualists (*Balubaale)* may foretell and give advice to their clients based on reflective and future foresight of what may or may not be known to the clients, and support their clients in taking appropriate actions. *Kulagula* is conducted through supernatural means and its purpose is to

find out clients’ needs, root causes of particular situation, and knowledge and information about future unknown spiritual aspects.

During divination (*Kulagula),* the spiritualists usually use a health assessment tool (*omweso*), and a process involves throwing up of shells and beads that have particular meaning and connotation concerning how they fall in relation to one another. The number of times the spiritualists throw up the shells and beads depend on the nature of the problem and guidance by the invoked ancestral spirits in charge of the divination.

The *Mulubaale* starts off the *kulagula* process by getting in touch with *Muzimu,* (*Mizimu* for plural), the spirit of a dead person, that owns the rest of ancestral spirits, *Misambwa* and *Mayembe*, under its leadership. The *Muzimu* may manifest and talk through a human spirit medium. The research participants variably described *Kulagula* as follows below;

“Divination is the process we use to know, inform, foretell and advise our clients, based on reflective and future foresight." (Participant 7).

“Divination is telling a client about something known or unknown to them to assist in taking appropriate actions." (Participant 9).

" Divination is when a client seeks and consults with a spiritualist to know and understanding." (Participant 5)

Divination is usually followed with exploration of ancestral spirits.

Exploration of Ancestral Spirits *(Okwaaza Lubaale)*

Okwaaza Lubaale is a cultural ritualistic process of exploration of ancestral spirits that involves family and clan members. The process is led by known and knowledgeable spiritualist *(Ssenkulu)* and involves cleansing rituals, singing, drumming and calling upon ancestral spirits to express themselves through any family members of their choice. The process also serves to establish root cause of illness and disease to individuals and family

members. The process may take three to twenty-seven (3-27) days. One participant stated that *"okwaaza Lubaale is a Baganda cultural practice to explore clan ancestral spirits. It also serves to diagnose and establish root cause of problems within family."* (Participant 7). The process is often prompted by illness and disease of individuals in a family, and any family member may be possessed by the ancestral spirits. One research participants share their personal experience; *"In my father's clan, we experienced multiple problems and illness. One time, about 80 of us gathered at a spiritualists big shrine to explore our ancestral spirits. The spirits possessed me and narrated the whole story, even though I was young and not staying with my immediate family."* (Participant 9).

Health management process

The Luganda word that best describes the health Management process is *Kuganga.* Participants described *Kuganga* as measures that the mulubaale takes to treat and protect the physical and spiritual bodies, mind, spirit, against problems, spiritual attacks and witchcraft. It should be noted that Kuganga is not limited to health management process alone but it may include protection of family, home, or property. Participants said that *Kuganga* is often performed under instructions and guidance of ancestral spirits. *"okuganga is a form of power used for protection and prevention against evil spirits and associated problems."* (Participant 6); *"Okuganga protects both the physical body and the spirits."* (Participant 9);

Words used in the health management process (*Kuganga*) may include *Kwambulula* (ritual cleansing), *Kusandaga* (scarification), *Ensiriba* and *Yirizi* (Amulets), *Kyoogo* (concoction of herbs for ritual baths) and *Kyogero* (decoction of herbal mixture for bathing children).

Ritual Cleansing (*Kwambulula*)

Participants expressed that *Kwambulula* is a ritualistic methodological cleansing process involving techniques that is linked to spiritual powers of living materials such as plants, animals, and birds, and non-living materials including waters, rocks and minerals. These materials are used in combination with ancestral spirits and other spiritual powers that are invoked by the spiritualist. The purpose of *Kwambulula* is to remove undesired spiritual influences from individuals and families, for purposes of protection, prevention, treatment and health promotion, and shrines. For example, one participant explained *"Shrines are routinely and ritualistically cleansed of any evil spirits, un-appropriate previous undertakings or witchcraft."* (Participant 2).

Scarification (*Kusandaga*)

Participants described *Kusandaga* (Scarification) as health management approach that involves incisions on physical body with sharp instruments. On healing for some patients, the incisions become visible scars on their bodies. The freshly made incisions serve as a way of introducing medications into human body to prevent, protect and promote good health. Participants believed medication put on freshly made incisions is more effective when it gets in contact with human blood while inside the body. Participants explained; *"Scarification is to make incisions on the skin with sharp instruments and introduce medication into the body."*(Participant 9). The scarification process is usually done by a trained *mulubaale* although sometimes, the ancestral spirits themselves make scarifications to the clients. As one participant reported, *"at times, the ancestral spirits do scarification to clients at night while they are sleeping."* (Participant 3). It should however, be noted that not every spirit does scarification, some spirits did not subscribe to rituals involving scarification nor contact with human blood.

Amulets, (*Nsiriba* and *Yirizi*)

Amulets included *Nsiriba* and *Yirize*. Participants described them as combinations of small pieces of physical materials with spiritual powers. Physical materials included parts of animals, birds, insects, reptiles, rocks, plants and minerals ritualistically imbued with ancestral spiritual powers. Amulets are tied together and put close to the body for prevention and protection against intentional and non-intentional negative influence of evil spirits and witchcraft. It is common practice to place the amulets in the waist or ankle or wrist or upper part of the arm of the individual. The placement of the amulets may either be put in one place or sometimes in more than one place depending on the instructions given by the *mulubaale* or the ancestral spirits.

The Concepts of *Kyoogo* and *Kyogero*

Ekyoogo was described as a concoction of medicinal herbs, with spiritual values, mixed with water, and are used for ritualistic cleansing and spiritual sanitization of people, especially before acceptance into spiritual space or spiritual activities. For example, one participant described *"Kyoogo is concoction used for cleansing, especially during most spiritual activities. Water type, its content, and timing for ritual cleansing are dependent on purpose of undertaken ritual and guidance by ancestral spirits."* (Participant 1).

Kyogero was defined as a decoction of medical herbs with spiritual powers used to bath infants and children to cleanse, immunize, prevent, treat, protect, and provide good luck. It is bathed daily, continuously for most of the infants and early childhood years.

Discussion

The main findings to research question "what words do you use to describe health, illness and disease?" are based on knowledge passed on from ancestral spirits, senior spiritualists and from their roles as spirit mediums. Good Health is visualized beyond the bodily physical health and focus on non-physical health aspects that include spirit, soul and good relations with ancestral spiritual, social and cultural life. On the other hand, Illness is conceptualized as an unclear bad health condition that often occurs suddenly with many possible root causes, often attributed to disgruntled ancestral spirits and witchcraft. Illness affects human soul and spirit and gradually manifest in the physical signs and symptoms. Disease is then seen as a multitude of signs and symptoms that manifested on the physical and biological body parts such as vomiting, fever and pain. Disease is related to micro and macro organisms and physical damages of the body, and is not related to any spiritual causes.

These descriptions are similar with what has been described elsewhere and support the argument that health is not a universal concept (47–49). Health is variably approached and interpreted by numerous people, cultures, societies and groups, and there is no single approach that can comprehensively define health in a way that would stand valid and good for all the people, in all communities and places (50). This is in line with WHO definition of health, which is defined as a state of complete physical, mental and social well-being and not merely the absence of disease or infirmity promoted social welfare as an integral component of overall health, and linked health to social environment and living and working conditions (51). This definition prompts advocating for working, practical, and operational definitions of health, and adoption of policies and programs for maintaining and improving health (19).

The *Balubaale* clearly distinguished disease from illness. Whereas disease relates to concepts of the proper anatomical, physiological and biological irregularities and

dysfunctions of body organs, tissue and cells (52–54), illness on the other hand is perceived as personal and interpersonal suffering based on social-cultural standards and values (55–57). The Balubaale say that disease is a multitude of signs and symptoms that manifest on the physical and biological parts of the body. Their definition aligns with the universal definition of disease which is based on objective, empirically identifiable, measurable, neutral and value-free pathological issue, engaging microorganisms and health determinants that can be epidemiologically and bio-statistically determined (50).

Illness is philosophically based on mystical concepts (58), usually culturally structured (47,59,60), and attached with various social meanings (61,62). This aligns with the *Balubaale's* concept of illness. They believe that illness is unclear and unnamed spiritual health condition caused by ancestral spirits and witchcraft. Their description of illness is embedded in their culture. Just like other authors who assert that illness is associated with misfortunes (63) and somatic experiences reflective of bad relations with supernatural forces and ancestral spirits (64). Illness has various possible cause that may include, but not limited to curses, witchcraft, and failure to observe social taboos (65). At other times, illness may manifest as a divine punishment (47,66).

Illness and disease were also expressed in perspectives of first person and third person. Illness is the first person experience and feeling of the condition (53,54), while disease is the third person's perspective and knowledge of somebody's medical condition (53,67). Inthorn expressed the same differently (68). He said that illness is the subjective understanding of the lack of health, while the perspective of medical professionals on medical conditions is the disease (68). They added that illness and disease are not the same, since a person can feel well and at the same time have an undiscovered tumour, or a person can feel sick without a medical condition (68).

Another concept of importance is the concept of health assessment. In the Balubaale world of heath management, health assessment involved divination, and ritual cleansing. The purpose of these two health assessment procedures is to establish the health status, the prospected root cause, the management approaches and prevention. For instance, divination is mainly descriptive of the prospected root causes, purpose and management approaches. This is in line the findings that healers use divination to establish the etiology of illness (69). The Balubaale use of divination is also in line with what Sabir et al., (2023) says, that traditional healers sometimes use divination to assess health status. Furthermore in line with this, traditional healers in North Eastern Ethiopia do the same, they use divination to establish the etiology of illness (71), while other healers use divination to distinguish between illness of supernatural origin and illness described as of normal or natural (72). It should be noted that this contrasts with western medicine approach (white Man's approaches) where health statues and illness are assessed with physical examination involving inspection, palpitation, percussion and auscultation (73,74).

Another example is ritual cleansing according to the Balubaale it is one of the methods used in traditional health management. This aligns with other culturally practices like the Swati traditional healers in Mpumalanga Province, South Africa who use ritual cleansing to manage depression and mental illness (75). In related case, in Peru, Ayahuasca rituals are used to cleanse and understand the meaning of the illness and establish a balanced relationship with illness and treatment (76). Another related example of ritual cleansing is in Indonesia where traditional rituals are used to reduce anxiety and build better mental health in response to natural and non-natural illness (77).

Study limitations

This study was conducted at multiple healers' shrines (sites) and was limited to one category of traditional health care spiritualists (*Balubaale*). This limited the breadth of the data as other categories of traditional health care specialists could have provided different perspectives or relevant information to the research question. The study area was limited to Central Uganda, however there is a possibility that if the study area was extended beyond the Central Region, more insightful conclusions could have been generated. These limitations restrict the generalizability of the study findings, even though the researcher closely worked with the research participants for two years, which enabled the researcher access sacred places and privileged information.

Conclusion and recommendation

The research findings show that although the concepts of health, illness, disease are universally used and may mean the same thing. The Baganda traditional health care spiritualists *(Balubaale)* use them uniquely to describe health, illness, disease, and management methods in their cultural context and are descriptive of the nature of problem, prospected root causes, therapeutic interventions, approaches, and associated spiritual entities.

We recommend more studies in other ethnic or tribal groupings, to explore words used to describe health, illness, disease and related management approaches so as to compare results and guide inter-medical systems communication and health policy formulations.

Acknowledgement

We are indebted to the leadership of Mbarara University and Pharmbiotrac program for soliciting the financial support for the first author PhD study, in particular, Dr. Casim Tolo, Professor Patrick Ogwang and Engineer Anke Weisheit. We extend my utmost gratitude to the study participants and their spiritual guides for information, free atmosphere and time extended to the research process. We are grateful to the leaders of traditional healers' associations, namely Sylivia Namutebi (Maama Fiina) of Uganda N'eddagala Ly'ayo N'obuwangwa Bwaffe, Musasizi Karim of NACOTHA, and Karim Walyabira of Uganda N'eddagala Ly'ayo. We are thankful to Buganda Kingdom administration for introducing us to the clan leaders whose support made the interaction with the spiritualists smooth. We thank the research assistants Mr. Eric Kibirige Mukasa and Ms. Nambuya Barbra for the field work. We also thank Dr. John Chrysostom Katongole of Munyonyo Botanical Gardens, Jovent K. Obbo of Bugema University and Dr. Kizito Simon of Makerere University for their comments on the draft report.

Supporting information

All the data supporting our findings are contained in the paper.

Reference

1. Abraham L. Indian systems of medicine (ISM) and public health care in India. Rev Healthc India. 2005;187(223).
2. De Taeye L. Living Presence in Postcolonial Congo: A Comparison between the Narrative Evocations of Ancestral Spirits in Jacques Bergeyck's Het stigma and Sony Labou Tansi's La vie et demie. Canadian Review of Comparative Literature/Revue Canadienne de Littérature Comparée. 2022;49(1):155–69.
3. Mokgobi MG. Understanding traditional African healing. Afr J Phys Health Educ Recreat Dance. 2014 Sep;20(Suppl 2):24–34.
4. Reddy S, Subedi B, Guite N. Introduction: Ethnomedicine and Tribal Healing Practices in India: Challenges and Possibilities of Recognition and Integration. In: Reddy S, Guite N, Subedi B, editors. Ethnomedicine and Tribal Healing Practices in India: Challenges and Possibilities of Recognition and Integration [Internet]. Singapore: Springer Nature Singapore; 2023. p. 1–31. Available from: https://doi.org/10.1007/978-981-19-4286-0_1
5. Van Dyk AC. Traditional African1 Beliefs and Customs: Implications for AIDS Education and Prevention in Africa. South African Journal of Psychology [Internet]. 2001 Jun [cited 2023 Jul 27];31(2):60–6. Available from: http://journals.sagepub.com/doi/10.1177/008124630103100208
6. Haverkort B. Relation between different knowledge communities: Rejection, substitution, complementatiry, and co-creation of sciences. In: Millar D, Shankar D, Burgoa DFreddy, Millar D, Shankar D, Burgoa DF, editors. Towards Co-creation of Sciences: Building on the Plurality of Worldviews, Values and Methods in different knowledge communities. New Dehli, India: Nimby Books, Content Services & Publishing Pvt Ltd.; 2012.

7. Lefler LJ. Traditional knowledge and health: Lessons from the Eastern Band of Cherokee Indians. Weaving Our Discipline with Community. 2020;2014(45):110–25.

8. The Witchcraft Act. 1956; 1935; 1928, 1998.

9. Odora Hoppers CA, Richards H. Rethinking thinking: modernity's "other" and the transformation of the university. 1st ed. Pretoria: University of South Africa; 2012.

10. Hoppers CAO, Sandgren B. Meeting of minds and futures: The nature of knowledge in diverse global settings. Journal of Adult and Continuing Education [Internet]. 2014 Nov [cited 2023 Jun 12];20(2):79–91. Available from: http://journals.sagepub.com/doi/10.7227/JACE.20.2.7

11. Hoppers CAO. Beyond critique to academic transformation : Reconceptualising rurality in the global south. Postcolonial Directions in Education,. Postcolonial Directions in Education [Internet]. 2017;6(2):145–64. Available from: https://www.um.edu.mt/library/oar//handle/123456789/26045

12. Bartel R. Vernacular knowledge and environmental law: cause and cure for regulatory failure. Local Environment [Internet]. 2014 Sep 14 [cited 2023 Jul 4];19(8):891–914. Available from: http://www.tandfonline.com/doi/abs/10.1080/13549839.2013.798636

13. Pandora K. Knowledge held in common: Tales of Luther Burbank and science in the American vernacular [Internet]. 2001 [cited 2023 Jul 4]. Available from: https://www.journals.uchicago.edu/doi/epdf/10.1086/385279

14. Adu-Gyamfi S. From vital force to the scientific or an admixture: A historical discourse on individuals value for indigenous medical practices in Ghana. Journal of Basic and Applied Research International. 2018 Mar;

15. Nigosian SA. World Faiths. New York: St. Martin's Press; 1994. 2 p.

16. Vaidyabhushanam RT. Glimpses of Wisdom; A collection of essays on Ayurveda. 1st ed. Karnataka, India: Institute of Ayurveda and Integrative Medicine (I-AIM) and Foundation for Revitalization of Local Health Traditions (FRLHT); 2010.

17. WHO-Africa. Promoting The Role of Traditional Medicine in the Health Systems: A Strategy for the African Region [Internet]. WHO; 2000 [cited 2020 Sep 19]. Available from: https://apps.who.int/iris/bitstream/handle/10665/95467/AFR_RC50_9.

18. WHO WHO. WHO Traditional Medicine Strategy 2014-2023. 2013.

19. Svalastog AL, Donev D, Kristoffersen NJ, Gajović S. Concepts and definitions of health and health-related values in the knowledge landscapes of the digital society. Croat Medical Journal. 2017;(58):431–5.

20. Carel H, Cooper R. Health, illness and disease: philosophical essays. London: New York Routledge; 2014.

21. Hofmann B. On the triad disease, illness and sickness. Med Health Care Philos. 2002;27:651–73.

22. Nordenfelt L. On the disease, illness and sickness distinction: a commentary on Andre Twaddle's system of concepts. In: Disease, illness and sickness: three central concepts in the theory of health. Linköping; 1994. p. 19–36. (Studies on Health and Society; vol. 18).

23. Sadegh-Zadeh K. Fuzzy health, illness, and disease. J Med Philos. 2000;25:605–38.

24. Ngeh SE. Conflicting interests between public health and custodians of indigenous knowledge with regards to curation and dissemination of information about Xhosa initiation rites. 2019; Available from: http://hdl.handle.net/11427/30847

25. Poirier B, Sethi S, Haag D, Hedges J, Jamieson L. The impact of neoliberal generative mechanisms on Indigenous health: a critical realist scoping review. Global Health [Internet]. 2022 Jun 15 [cited 2023 Jun 12];18(1):61. Available from: https://globalizationandhealth.biomedcentral.com/articles/10.1186/s12992-022-00852-2

26. Chen YY, Liu J, Chen H, He Y, Chen H. Divergence and integration of identification methods for geriatric mental health disorders in nonspecialized institutions—a qualitative study of service providers from different departments in Shanghai, China. Aging & Mental Health

[Internet]. 2023 Mar 2 [cited 2023 Jun 12];1–9. Available from: https://www.tandfonline.com/doi/full/10.1080/13607863.2023.2180798

27. Mthembu MV, Ndzinisa N. Expanding Language? Struggles that Characterise the Development of New Words and Meanings on Eswatini's National Radio. In: Mpofu P, Fadipe IA, Tshabangu T, editors. Indigenous African Language Media [Internet]. Singapore: Springer Nature Singapore; 2023 [cited 2023 Jun 12]. p. 117–35. Available from: https://link.springer.com/10.1007/978-981-99-0305-4_8

28. Haque IMd, Chowdhury ABMA, Shahjahan Md, Harun, Dostogir GMd. Traditional healing practices in rural Bangladesh: a qualitative investigation. BMC Complementary and Alternative Medicine. 2018;18(62).

29. Kajawu L, Chiweshe M, Mapara J. Community perceptions of indigenous healers and mental disorders in Zimbabwe. OJPsych [Internet]. 2019 [cited 2023 Jun 12];09(03):193–214. Available from: http://www.scirp.org/journal/doi.aspx?DOI=10.4236/ojpsych.2019.93015

30. Kasilo OMJ, Wambebe C, Nikiema JB, Nabyonga-Orem J. Towards universal health coverage: advancing the development and use of traditional medicines in Africa. BMJ Glob Health [Internet]. 2019 Oct [cited 2023 Aug 24];4(Suppl 9):e001517. Available from: https://gh.bmj.com/lookup/doi/10.1136/bmjgh-2019-001517

31. Holmes D. Report of the global conference on primary health care: From Alma-Ata towards universal health coverage and the sustainable development goals [Internet]. Astana Kazakhstan: WHO; 2019. Report No.: WHO/UHC/IHS/2019.62. Available from: ; https://creativecommons.org/licenses/by-nc-sa/3.0/igo).

32. Krah E, Kruijf J de, Ragno L. Integrating Traditional Healers into the Health Care System: Challenges and Opportunities in Rural Northern Ghana. J Community Health. 2018;43:157–63.

33. Page-Reeves J, Niforatos JD, Mishra S, Regino L, Gingerich A, Bulten R. Health Disparity and Structural Violence: How Fear Undermines Health Among Immigrants at Risk for Diabetes,. Journal of Health Disparities Research and Practice. 2013;6(2):Article 3.

34. Clarke A, Yates T, Smith AC, Chilcot J. Patient's perceptions of chronic kidney disease and their association with psychosocial and clinical outcomes: a narrative review. Clin Kidney J. 2016;9:494–502.

35. Glattacker M, Heyduck K, Meffert C. Illness beliefs and treatment beliefs as predictors of short and middle term outcome in depression. J Health Psychol. 2013;18:139-52.

36. Heyduck K, Meffert C, Glattacker M. Illness and treatment perceptions of patients with chronic low back pain: characteristics and relation to individual, disease and interaction variables. J Clin Psychol Med Settings. 2014;21:267–81.

37. Kaptein A, Schoones J, Fischer M, Thong M, Kroep J, van der Hoeven K. Illness perceptions in women with breast cancer-a systematic literature review. Curr Breast Cancer Rep. 2015;7:117–26.

38. Petrie K, Weinman J. Patients' perceptions of their illness. The dynamo of volition in health care. Curr Dir Psychol Sci. 2012;21:60–5.

39. Schlomann P, Schmitke J. Lay beliefs about hypertension: an interpretive synthesis of the qualitative research. J Am Acad Nurse Pract. 2007;19:358–67.

40. Dei GJS, Jajj M. Knowledge and Decolonial Politics: A Critical Reader [Internet]. BRILL; 2018 [cited 2023 Jun 12]. Available from: https://brill.com/view/title/39014

41. Hartmann WE, Saint Arnault DM, Gone JP. Conceptualizing culture in (global) mental health: Lessons from an urban American Indian behavioral health clinic. Social Science & Medicine. 2022 May 1;301:114899.

42. MOH U. Policy Guidelines on Traditional Medicine Development. Uganda Government; 2005.

43. UBOS - Uganda. Statistical abstract 2022 - Uganda. Uganda: Uganda Bureau of Statistics (UBOS); 2023.

44. Dworkin SL. Sample Size Policy for Qualitative Studies Using In-Depth Interviews. Arch Sex Behav. 2012;41:1319–20.

45. Akol A, Moland KM, Babirye JN, Engebretsen IMS. “We are like co-wives”: Traditional healers’ views on collaborating with the formal Child and Adolescent Mental Health System in Uganda. BMC Health Serv Res. 2018 Dec;18(1):258.

46. Canel Çınarbaş D, Tuna E, Ar-Karci Y. Turkish muslim healers: A qualitative investigation of Hocas and their methods. J Relig Health [Internet]. 2020 Oct [cited 2023 Apr 2];59(5):2397–413. Available from: http://link.springer.com/10.1007/s10943-019-00885-9

47. Ferrari FM. “Illness is nothing but injustice”: The revolutionary element in Bengali folk healing. The Journal of American Folklore. 2015;128(507):46–64.

48. Mishra A. Recipes for immortality: Medicine, religion, and community in South India by Richard S. Weiss. Medical Anthropology Quarterly [Internet]. 2010 Jun [cited 2023 May 15];24(2):274–6. Available from: https://onlinelibrary.wiley.com/doi/10.1111/j.1548-1387.2010.01102.x

49. Sujatha V. Pluralism in Indian medicine: Medical lore as a genre of medical knowledge. Contributions to Indian Sociology [Internet]. 2007 May [cited 2023 May 15];41(2):169–202. Available from: http://journals.sagepub.com/doi/10.1177/006996670704100202

50. Keleher H, MacDougall C, editors. Understanding health. Fourth edition. South Melbourne, Victoria, Australia: Oxford University Press; 2016. 349 p.

51. WHO. WHO Basic Document: Preamble to WHO Constitution. World Health Organization [WHO] Geneva; 2009.

52. Amoretti MC, Lalumera E. Wherein is the concept of disease normative? From weak normativity to value-conscious naturalism. Med Health Care and Philos [Internet]. 2022 Mar [cited 2023 Jun 6];25(1):47–60. Available from: https://link.springer.com/10.1007/s11019-021-10048-x

53. Boon P den. Illness and Disease, a new Perspective: Reflections on Phenomenology of Illness [Bachelor thesis for philosophy of medicine]. Erasmus University Rotterdam; 2019.

54. Sonowal CJ, Konc P. Disease Burden, Health-belief and Treatment-seeking Behaviour among the Particularly Vulnerable Tribal Groups of India. Ethno Medicine. 2021;15(1–2):60–73.

55. Keiran E. Health is in the eye of the beholder: Exploring normative and empirical conceptions of health and illness. SOPHIA: Journal of Philosophy Volume. 2022;XVI(2020–2022):21–34.

56. Madison G. “Illness” ... and its human values. Journal of Existential Analysis. 2002 Feb 13;13:10–30.

57. Paulon M. Hysteria: rise and fall of a baffling disease. A review on history of ideas in medicine. Journal of Psychopathology [Internet]. 2022 Sep [cited 2023 Jun 6];152–61. Available from: https://doi.org/10.36148/2284-0249-461

58. Madeira L, Leal B, Filipe T, Rodrigues MF, Figueira ML. The uncanny of the illness experience: Can phenomenology help? Psychopathology [Internet]. 2019 [cited 2023 Jun 8];52(5):275–82. Available from: https://www.karger.com/Article/FullText/504141

59. Charan IA, Xin S, Zezhuang W, Yao D. Rethinking efficacy: People’s perception of ritual healing and trance religious practices at shrines in Pakistan. Asian Journal of Psychiatry [Internet]. 2020 Aug [cited 2023 Jun 8];52:102020. Available from: https://linkinghub.elsevier.com/retrieve/pii/S1876201820301313

60. Powell R, Scarffe E. ‘Rethinking “Disease”: a fresh diagnosis and a new philosophical treatment.’ J Med Ethics [Internet]. 2019 Sep [cited 2023 Jun 8];45(9):579–88. Available from: https://jme.bmj.com/lookup/doi/10.1136/medethics-2019-105465

61. Kirmayer LJ. Abstracts and Reviews : The Illness Narratives: Suffering, Healing and the Human Condition by Arthur Kleinman. New York: Basic Books (1988). Transcultural Psychiatric Research Review [Internet]. 1989 Sep [cited 2023 Aug 7];26(3):202–4. Available from: http://journals.sagepub.com/doi/10.1177/136346158902600303

62. Kleinman A. Patients and healers in the context of culture: an exploration of the borderland between anthropology, medicine, and psychiatry$nArthur Kleinman. 8. [print]. Berkeley: University of California Press; 1980. 427 p. (Comparative studies of health systems and medical care).

63. Lock M, Vinh-Kim N. An anthropology of biomedicine. Oxford: Wiley-blackwell; 2010.

64. Guidos B, Calvario J, Hendrika van Zuilen M. A brief qualitative study of indigenous traditional medicine providers in Timor-Leste. Diálogos (FFCH - UNTL) [Internet]. 2022 Nov 16 [cited 2023 Apr 3];7:203–16. Available from: https://dialogosuntl.com/index.php/revista/article/view/8

65. Workneh T, Emirie G, Kaba M, Mekonnen Y, Kloos H. Perceptions of health and illness among the Konso people of southwestern Ethiopia: persistence and change. J Ethnobiology Ethnomedicine. 2018 Dec;14(1):18.

66. Trinos K, Mudaly R. Exploring possibilities for including indigenous knowledge into the biology teacher education curriculum: Leveraging insights from Karanga knowledge holders. Alternation [Internet]. 2020 Dec 1 [cited 2023 Jun 19];SP36. Available from: http://alternation.ukzn.ac.za/Files/articles/special-editions/36/05-trinos.pdf

67. Choifer A. A New Understanding of the First-Person andThird-Person Perspectives. Philosophical Papers. 2018;47(3):333–71.

68. Inthorn J. Cross-cultural bioethics as an interdisciplinary approach. In: Kappas M, Kelleher D, editors. A challenge of interdisciplinary research. Universitätsverlag Göttingen; 2012.

69. Salifu Yendork J, Kpobi L, Sarfo EA. “It’s only ‘madness’ that I know”: analysis of how mental illness is conceptualised by congregants of selected Charismatic churches in Ghana. Mental Health, Religion & Culture [Internet]. 2016 Oct 20 [cited 2023 Aug 7];19(9):984–99. Available from: https://www.tandfonline.com/doi/full/10.1080/13674676.2017.1285877

70. M. Sabir, Muh. Ardi Munir, Tri Setyawati, Muhammad Nasir, Ary Anggara, Ressy Dwiyanti, et al. Understanding Rural Perspectives and Practices of Health in Banggai Laut, Indonesia.

IJHESS [Internet]. 2023 Jun 26 [cited 2023 Aug 7];5(3):169–77. Available from: https://jurnal.unismuhpalu.ac.id/index.php/IJHESS/article/view/3734

71. Kahissay MH, Fenta TG, Boon H. Beliefs and perception of ill-health causation: a socio-cultural qualitative study in rural North-Eastern Ethiopia. BMC Public Health [Internet]. 2017 Dec [cited 2023 Aug 7];17(1):124. Available from: http://bmcpublichealth.biomedcentral.com/articles/10.1186/s12889-017-4052-y

72. Salifu A, Hamdan AY, Habibu I. Divination and Health Decision-Making among the Dagomba. ADRRI JOURNALS [Internet]. 2021;30(7). Available from: https://www.journals.adrri.org

73. Kennedy C, O'Connor G. Using health assessment skills in a non-medical prescribing chemotherapy clinic. Gastrointestinal Nursing [Internet]. 2016 Nov 2 [cited 2023 Aug 8];14(9):46–50. Available from: http://www.magonlinelibrary.com/doi/10.12968/gasn.2016.14.9.46

74. Quigley F, Martin DS. History-taking and clinical examination skills from a stoma nurse's perspective. Gastrointestinal Nursing [Internet]. 2013 Oct 2 [cited 2023 Aug 8];11(8):29–36. Available from: http://www.magonlinelibrary.com/doi/10.12968/gasn.2013.11.8.29

75. Ngobe A, Semenya S, Sodi T. Psychological ailments and their treatment protocols: a case study of Swati traditional healers in Mpumalanga Province, South Africa. Afr H Sci [Internet]. 2021 Aug 2 [cited 2023 Aug 7];21(2):884–95. Available from: https://www.ajol.info/index.php/ahs/article/view/211801

76. Maia LO, Daldegan-Bueno D, Tófoli LF. The ritual use of ayahuasca during treatment of severe physical illnesses: a qualitative study. Journal of Psychoactive Drugs [Internet]. 2021 May 27 [cited 2023 Aug 7];53(3):272–82. Available from: https://www.tandfonline.com/doi/full/10.1080/02791072.2020.1854399

77. Sukmawan S, Sahiruddin S. Tradition-responsive Approach as Non-medical Treatment in Mitigating the Covid-19 Pandemic in Tengger, East Java, Indonesia. In: Proceedings of the 1st International Seminar on Cultural Sciences, ISCS 2020, 4 November 2020, Malang, Indonesia

[Internet]. Malang, Indonesia: EAI; 2021 [cited 2023 Aug 7]. Available from:

http://eudl.eu/doi/10.4108/eai.4-11-2020.2308894

Click here to access/download
Supporting Information
Zipped files - Consent forms.rar

Click here to access/download
Supporting Information
Zipped files - Research tools.rar

Click here to access/download
Supporting Information
Zipped files - Ethics approvals and clearance.rar
