# Supplementary material for: Perspectives on health, illness, disease and management approaches among Baganda traditional spiritual healers in Central Uganda
Source: PLOS Glob Public Health. 2024 Sep 6;4(9):e0002453. doi: 10.1371/journal.pgph.0002453 (PMC11379289; doi:10.1371/journal.pgph.0002453)
Supplement: Attachment — Submitted filename: Response to reviewers - illness, disease, health management.docx [file pgph.0002453.s015.docx]

**Rebuttal letter – Response to reviewers**

Dear Sir/Madam

Thank you very much for your time to review our manuscript and for your suggestions and comments. We hereby respond to each of the point raised below

**Overall observation**

The authors seem to think their study is the first (or one of the first) on this subject. This is incorrect. There are many studies that describe similar rituals, practices and concepts and the authors don't seem to have incorporated any of these ideas into their text. This is a serious concern.

*It is true, we started with that incorrect feeling.*

*We have researched and found other similar studies and we have incorporated some of their ideas into our discussions.*

After more reading and research about previous studies have been completed by the authors, there needs to be an extensive revision of the text.

*More reading and research of previous studies has been done.*

*Extensive revision of the text has been done*.

**Suggestions and Comments**

The article is really interesting, as it explores how traditional health care spiritualists in central Uganda see health, illness, disease, and management practices.

*Thank you for the compliment*

**Title**

The title explains the purpose and objective of the article

*Thanks*

**Abstract**

The corrections have been made.

**Introduction**

Overall, the Introduction has serious problems.

*The introduction has been revised and the problems addressed.*

You don't need the acronym THCS. Just call them "traditional healers" as they are referred to everywhere else in the literature. The acronym is too cumbersome

*Thank you for the observation.*

*The acronym THCS is removed.*

*I have called the study participants traditional spiritual healers to be more precise*

Regarding the third, fourth, and fifth sentence in the Introduction: These are oral cultures and are highly stigmatised especially by locals (viewed as "evil"/witchcraft). These are the primary reasons there is little research on this subject.

*Thank you*

*That section of the introduction has been restructured*

If you're going to use a term as loaded as "knowledge apartheid" you're going to need to do much more explaining as to what they means and why that is appropriate.

*The whole statement has been removed.*

Traditional medicine is not used by high percentages of Americans, Australians, Germans and Canadians.

*The statement is removed.*

You need to do significantly more reading on the background of traditional religion in Africa as the authors do not seem to grasp the socio-religious context of the subject they are treating here. This is very concerning.

*More reading has been done on the background of traditional health cultures in Africa and we focused on socio-cultural context of health, the subject we are dealing with.*

*We endeavored not to dwell in the various African cultural-religious aspects, because it is not the focus of the study. The study focus was on socio-cultural context of health.*

The authors need to do significant more research about traditional healer practices already documented throughout east and southern Africa.

*Already documented traditional healing practices in East and Southern Africa have been researched, read and related with.*

Traditional healing" as you are examining it is directly related to pre-Christian and pre-Islamic African traditional religions. They are still present today in a syncretic form with Christianity and Islam. This is why they exist so significantly in Africa and not in the places you mention.

*Partly true,*

*the traditional healing, we are examining is directly related to the socio-cultural health related beliefs (not necessarily religious) that existed before Christianity and Islam in African, that are still present to the current day amongst traditional spiritual healers amidst Christianity and Islam.*

More reading should be done about studies of traditional healing in other countries in east and southern Africa (which share many of the same traditional spiritual belief systems and practices related to traditional healing).

*It is done, thank you*

*More reading and reference have been made about traditional healing in other countries including in Eastern and Southern Africa that share similar traditional spiritual belief systems and healing practices*

**Corrections**

introduced education curricula banned people using...replace with: introduction of education curricula banned people from using...

*Done*

in relation to concepts ...replace with: about concepts...

*Done*

Insistence should be for traditional ...replace with: emphasis should be on traditional...

*Done*

**Methods**

The authors mentioned that Uganda is divided into four regions, while the study covered only the central part? The authors did not explain the reason behind choosing this particular region to conduct the study. So, please explain the reasons for more clarity

*The basis for the selection of central region has been clarified.*

**Correction**:

Additional identification was done by district and cultural leaders...replace with: District and cultural leaders completed additional identification....

*Done*

Although the study consumed a great deal of effort from researchers to deal with spiritual healers and understand the rituals used in addition to translating terminology, the number of participants is very small to explore this issue, especially since there is a difference in some participants’ opinions and explanations regarding some terminology.

I'm concerned about the disproportionate numbers of men interviewed compared to women.

- There needs to be a more thorough justification of this imbalance.

*Out of the 149 participants identified, only 12 qualified to participate in the study. There were no alternative to increase the numbers as reflected in the process of participant recruitment*

*All the identified participants were considered on the same basis as presented in the study profile Figure 1. The inclusion and exclusion criteria were not sensitive to gender.*

*We did not make any added effort to balance the gender, as that would interfere with the neutral findings on the field.*

- How did this affect the findings?

*The inclusion of the need to be a spirit medium for Muwanga might have reduced the female spirit mediums since Muwanga is a male spirit and most of its mediums are made. However, this did not compromise their health-related beliefs and practices.*

*Indeed, the small numbers of the participants is part of the limitations of the study.*

It has been mentioned that the first researcher was very close to the participants and worked with them in their workplaces for two years, while the specific period of the study was approximately one year (between 15th July 2019 and 29th April 2020)...So, please explain that for more clarity.

*All the remaining twelve (10Male; 2Female) study participants were* ***recruited into the study between 15^th^ July 2019 and 29^th^ April 2020*** *and were prospectively interacted with for two years*.

It is noted that the focus has been on the inclusion criteria and merging them with the exclusion criteria, so please separate them and explain them sufficiently for greater clarity. Please separate the inclusion criteria from the exclusion criteria and explain them adequately.

*Done.*

*The inclusion and exclusion criteria is adequately explained in figure 1*.

**Correction:**

The data was transcribed by the researcher and research assistants directly into English while maintaining depth of context in Luganda, the local language of spiritualists...replace with: The researcher and research assistants directly translated the data into English while preserving the rich context in Luganda, the local language of spiritualists.....

*Done*

**Findings**

The results section is difficult to follow.

*The point is noted, thank you.*

*Efforts have been made to reduce some details to make the findings easy to follow.*

Why are all these terms important?

*These terms are important because they are the essence of the study, they are specific and descriptive in nature.*

There are also serious problems with translation into English. For example, the dichotomy you present about "illness" versus "disease" should actually be translated as "supernatural illness" (connected to ancestral spirits and witchcraft) and "natural illness" (connected to biomedical problems).

Thank you for the observation

*In this case we translated in English but maintaining the meaning in the context of the traditional spiritual healers. To them, the issue was not about natural and supernatural illness, it was about the prospected root causes, the effects and affected part of the person and the appropriate respective management approaches and processes*

Many other studies have documented this distinction throughout east and southern Africa. Use terms already established in the literature to avoid confusion.

*Thanks for the advice*

*Efforts have been made to use terms already established in literature, especially were the meaning remains similar. However, where the meaning gets changed, the terms used in the field are maintained.*

Quotes in the divination section should be incorporated into the text. Not listed at the end. Why are these quotes important?

*Thank you for the advice and the question*

*One quote is incorporated into the text. The rest of the quotes have been removed.*

Similar divination practices have been documented widely throughout east and southern Africa. The authors should fully research these examples.

*True*

*Similar documented divination practices have been researched, read, quoted and related to.*

This is the same with ritual cleansing and scarification (often referred to as "cutting" in the literature).

*Thank you.*

*Ritual cleansing and scarifications (cutting) has been researched, read and related to*

**Correction:**

Study participants were twelve (10 Male, 2 Female) ...replace with: The study participants were twelve (10 males and 2 females).....

*Done*

It has been mentioned that most words did not have English equivalents in mainstream medicine. So, when they were analyzed, did they give a meaning close to the intended meaning in the local language?

*Yes please.*

*We used the English words close to the intended meaning in the local language.*

**Discussion**

Please shorten the first paragraph and focus on discussing the results.

*Done*

The discussion needs to be supported by additional references to compare the results of the current study with those of previous studies.

*Done*

The discussion focused on comparison with the results of two previous studies from Ethiopia and Saudi Arabia. Therefore, I suggest that the authors support the discussion section with references and other recent studies, especially from developing countries, which rely heavily on this type of medicine.

*Done*

**Conclusion**

The research offers sample data for the authors to draw conclusions from

*Thanks*

**Reference**

*Reference has been updated*

**Suggestion to correct the error**

There is no need to capitalize words like "Physical" or "Physiological" or "Good Health".

*Corrected*

There are also sloppy mistakes with italics.

*Corrected*

Overall, the text needs to be fully reviewed for spelling, grammar and punctuation more thoroughly.

Grammar: Need revision along the manuscript.

*Efforts have been made to review the text for spellings, grammar and punctuation*
