# Supplementary material for: Perspectives on health, illness, disease and management approaches among Baganda traditional spiritual healers in Central Uganda
Source: PLOS Glob Public Health. 2024 Sep 6;4(9):e0002453. doi: 10.1371/journal.pgph.0002453 (PMC11379289; doi:10.1371/journal.pgph.0002453)
Supplement: Attachment — Submitted filename: Response to reviewers.docx [file pgph.0002453.s016.docx]

**Rebuttal letter – Response to reviewers**

Dear Sir/Madam

Thank you very much for your time to review our manuscript and for your suggestions and

comments. We hereby respond to each of the point raised

Delete the third phrase in the introduction or clarify what “colonizing ideology” means.

*The phrase “colonizing ideology” is deleted and the sentence is rephrased*

Avoid using any language that shows the author’s opinions or personal value judgments: “unethical” in paragraph 1

*The word “unethical” is removed*.

Also what does paragraph 2 in the introduction mean? Healers have had multiple accusations? About what? By whom? And they’ve “lost pride and confidence”? In what? If they didn’t still have confidence, they wouldn’t be doing this work so that sentence makes no sense.

*Paragraph 2 is removed; the first sentence is deleted and the remaining part of the paragraph is connected to the first paragraph*

The authors have chosen to continue creating a distinction between "illness" and "disease" (olumbe and obulwadde in the text). This is a mistake as it doesn't make sense in English.

*The study findings clearly indicate that the Baganda traditional spiritual healers have clear distinctions between illness and disease (olumbe and obulwadde). We are just reporting the findings. It is not true that the authors are creating the distinction between illness and disease (olumbe and obulwadde).*

*It may be partly true that the finding does not make sense in English (for now), but this may not necessarily translate it into being a mistake.*

If authors do not like the distinction of "natural" versus "supernatural" illness they need to find a better way to distinguish them in English as "illness" and "disease" are synonomous and should not be juxtaposed.

*The reviewer suggests or prefers that we, the authors, use the distinctions of natural and supernature illness. The findings indicate that such a suggestion only related to the causes. According to the Baganda traditional spiritual healers, natural causes were more related to disease, while supernatural causes were more related to illness. Otherwise, the spiritual healers were particular in relation to illness and disease (olumbe and obulwadde).*

*The research findings show that although the concepts of illness and disease (olumbe and obulwadde) are universally used and are assumed to mean the same thing, Baganda traditional spiritual healers (Balubaale) use them uniquely and differently. Their particularity about illness and disease is influenced by the root causes of illness and disease, management approaches, cultural settings and association with or without ancestral spirits*

*Furthermore, the use of words “illness and disease” (olumbe and obulwadde) by the traditional spiritual healers highlights that these words are not synonymous as commonly assumed and the spiritual healers juxtaposed these words.*

*The universal definition of disease is based on objective, empirically identifiable, measurable, neutral and value-free pathological issue, engaging microorganisms and health determinants that can be epidemiologically and bio-statistically determined* [1,2].

*On the other hand, Illness, is perceived as personal and interpersonal suffering based on social-cultural standards and values* [3–5]*, is philosophically based on mystical concepts* [6,7]*, which are usually culturally structured* [8–10]*, and attached with various social meanings* [11,12] *often associated with supernatural forces, ancestral spirits and witchcraft* [6,13]*. In a broad context, illness claims are of capricious, lack scientific rigor, and its measures are not clear or objective* [14].

*It is important to realize that significant misunderstanding may occur when these words, “illness and disease” (olumbe and obulwadde), are used infront of the traditional spiritual healers and Western trained health professionals, where the same words may mean different things depending upon their respective background knowledge, yet they are visited by and they treat the same patients, at times concurrently.*

The literature (including many articles cited below) largely utilizes the distinction of "natural" and "supernatural" illnesses and I strongly suggest the authors use either those terms of terms already cited in the literature to highlight these differences. "Illness" and "disease" are unacceptable.

*Thank you very much for the suggested literature. We have reviewed the suggested literature and some more. We have noted the interchangeable use of words illness and sickness [15], illness and disease* *[16]. We have used some of the literature to enrich the overall analysis and context of our study*

1. Abbo, C., Okello, E. S., Musisi, S., Waako, P., & Ekblad, S. (2012). Naturalistic outcome of treatment of psychosis by traditional healers in Jinja and Iganga districts, Eastern Uganda–a 3-and 6 months follow up. International Journal of Mental Health Systems, 6(1), 1-11. <http://www.ijmhs.com/content/6/1/13>
2. Galvin, M., Chiwaye, L., Moolla, A. (2023d). Religious and Medical Pluralism among Traditional Healers in Johannesburg, South Africa. Journal of Religion and Health, 1-17. <https://doi.org/10.1007/s10943-023-01795-7>
3. Galvin, M., Chiwaye, L., Moolla, A. (2023e). Perceptions of Causes and Treatment of Mental Illness Among Traditional Health Practitioners in Johannesburg, South Africa. South African Journal of Psychology, 1-13. <https://doi.org/10.1177/00812463231186264>
4. Louw, G., & Duvenhage, A. (2016). The present-day diagnosis and treatment model of the South African traditional healer. Australasian Medical Journal. <https://doi.org/10.21767/AMJ.2016.2721>
5. Peltzer, K. (1999). Faith healing for mental and social disorders in the Northern Province (South Africa). Journal of Religion in Africa, 29(Fasc. 3), 387–402. <https://www.jstor.org/stable/1581530>
6. Sorsdahl, K. R., Flisher, A. J., Wilson, Z., & Stein, D. J. (2010a). Explanatory models of mental disorders and treatment practices among traditional healers in Mpumulanga, South Africa. African Journal of Psychiatry, 13(4), 284–290. <https://doi.org/10.4314/ajpsy.v13i4.61878>
7. Sorsdahl, K., Stein, D. J., & Flisher, A. J. (2010b). Traditional healer attitudes and beliefs regarding referral of the mentally ill to Western doctors in South Africa. Transcultural Psychiatry, 47(4), 591–609. <https://doi.org/10.1177/1363461510383330>
8. Crawford, T., & Lipsedge, M. (2004). Seeking help for psychological distress: The interface of Zulu traditional healing and Western biomedicine. Mental Health, Religion & Culture, 7(2), 131–148. <https://doi.org/10.1080/13674670310001602463>
9. Shange, S., & Ross, E. (2022). ‘The question is not how but why things happen’: South African traditional healers’ explanatory model of mental illness, its diagnosis and treatment. Journal of Cross-cultural Psychology, 53(5), 503–521. <https://doi.org/10.1177/00220221221077361>
10. Mzimkulu, K., & Simbayi, L. (2006). Perspectives and practices of Xhosa-speaking African traditional healers when managing psychosis. International Journal of Disability, Development and Education, 53(4), 417–431. <https://doi.org/10.1080/10349120601008563>
11. Ngobe, A. (2015). Swati traditional healers’ conceptualisation of causes and treatment of mental illness [Doctoral dissertation]. University of Limpopo. <http://ulspace.ul.ac.za/bitstream/handle/10386/1281/ngobe_aj_2015.pdf>

Journal Requirements:

1. Please review your reference list to ensure that it is complete and correct. If you have cited papers that have been retracted, please include the rationale for doing so in the manuscript text, or remove these references and replace them with relevant current references. Any changes to the reference list should be mentioned in the rebuttal letter that accompanies your revised manuscript. If you need to cite a retracted article, indicate the article’s retracted status in the References list and also include a citation and full reference for the retraction notice.

*The references have been reviewed to ensure they are complete and correct*

*The following references have been* ***removed*** *from the reference list*

1. Kabumba B. Criminalizing Indigenous Belief: The Constitutional Deficits of Uganda’s Witchcraft Act. Oxford Journal of Law and Religion. 2023 Nov 8;rwad014. https://academic.oup.com/ojlr/advance-article/doi/10.1093/ojlr/rwad014/7385751 DOI: 10.1093/ojlr/rwad014
2. Rukundo S. Witch-Killings and The Law in Uganda. J law relig. 2020 Aug;35(2):270–96. https://www.cambridge.org/core/product/identifier/S0748081420000259/type/journal_article DOI: 10.1017/jlr.2020.25

*The following references have been* ***added*** *to the reference list*

1. Galvin M, Chiwaye L, Moolla A. Perceptions of causes and treatment of mental illness among traditional health practitioners in Johannesburg, South Africa. South African Journal of Psychology. 2023;53(3):403–15.
2. Galvin M, Chiwaye L, Moolla A. Religious and Medical Pluralism Among Traditional Healers in Johannesburg, South Africa. J Relig Health. 2024 Apr;63(2):907–23. https://link.springer.com/10.1007/s10943-023-01795-7 DOI: 10.1007/s10943-023-01795-7
3. Duvenhage A, Louw G. The present-day diagnosis and treatment model of the South African traditional healer. AMJ. 2016;9(11). http://amj.net.au/index.php/AMJ/article/view/2721/1443 DOI: 10.21767/AMJ.2016.2721
4. Abbo C, Okello ES, Musisi S, Waako P, Ekblad S. Naturalistic outcome of treatment of psychosis by traditional healers in Jinja and Iganga districts, Eastern Uganda–a 3-and 6 months follow up. International Journal of Mental Health Systems. 2012;6:1–11. http://www.ijmhs.com/content/6/1/13

2. Please provide separate figure files in .tif or .eps format only and remove any figures embedded in your manuscript file. Please also ensure all files are under our size limit of 10MB.

*Done*

**References**

1.	Alqahtani H. Statistical methods for enhanced reporting and analysis of COVID-19 survey data sets generated by internal public health bodies in Saudi Arabia: a biostatistical study [Internet]. In Review; 2022 Jul. https://www.researchsquare.com/article/rs-1804844/v1 DOI: 10.21203/rs.3.rs-1804844/v1

2.	Fugl-Meyer K. A medical social work perspective on rehabilitation. J Rehabil Med. 2016;48(9):758–63. https://medicaljournalssweden.se/jrm/article/view/15475 DOI: 10.2340/16501977-2146

3.	Keiran E. Health is in the eye of the beholder: Exploring normative and empirical conceptions of health and illness. SOPHIA: Journal of Philosophy Volume. 2022;XVI(2020–2022):21–34.

4.	Paulon M. Hysteria: rise and fall of a baffling disease. A review on history of ideas in medicine. Journal of Psychopathology. 2022 Sep;152–61. https://doi.org/10.36148/2284-0249-461 DOI: 10.36148/2284-0249-461

5.	Mwaka AD, Achan J, Orach CG. Traditional health practices: A qualitative inquiry among traditional health practitioners in northern Uganda on becoming a healer, perceived causes of illnesses, and diagnostic approaches. Olorunlana A, editor. PLoS ONE. 2023;18(4). https://dx.plos.org/10.1371/journal.pone.0282491 DOI: 10.1371/journal.pone.0282491

6.	Boarccaech A. Local Knowledge, Traditional Healers and Psychology: Causes, Symptoms And Treatments of Mental, Disorders in Timor.Leste. Diálogos (FFCH - UNTL). 2019;4:169–89. https://dialogosuntl.com/index.php/revista/article/view/72 DOI: 10.53930/27892182.dialogos.4.72

7.	Madeira L, Leal B, Filipe T, Rodrigues MF, Figueira ML. The uncanny of the illness experience: Can phenomenology help? Psychopathology. 2019;52(5):275–82. https://www.karger.com/Article/FullText/504141 DOI: 10.1159/000504141

8.	Ferrari FM. “Illness is nothing but injustice”: The revolutionary element in Bengali folk healing. The Journal of American Folklore. 2015;128(507):46–64. DOI: 10.5406/jamerfolk.128.507.0046

9.	Charan IA, Xin S, Zezhuang W, Yao D. Rethinking efficacy: People’s perception of ritual healing and trance religious practices at shrines in Pakistan. Asian Journal of Psychiatry. 2020 Aug;52:102020. https://linkinghub.elsevier.com/retrieve/pii/S1876201820301313 DOI: 10.1016/j.ajp.2020.102020

10.	Powell R, Scarffe E. ‘Rethinking “Disease”: a fresh diagnosis and a new philosophical treatment.’ J Med Ethics. 2019 Sep;45(9):579–88. https://jme.bmj.com/lookup/doi/10.1136/medethics-2019-105465 DOI: 10.1136/medethics-2019-105465

11.	Kirmayer LJ. Abstracts and Reviews : The Illness Narratives: Suffering, Healing and the Human Condition by Arthur Kleinman. New York: Basic Books (1988). Transcultural Psychiatric Research Review. 1989 Sep;26(3):202–4. http://journals.sagepub.com/doi/10.1177/136346158902600303 DOI: 10.1177/136346158902600303

12.	Ines Amada L, Soledad Burgos V, Ferreyra M, Leguizamón Ibañez DB, Estefanía Lopez V, Rivas Medina DZ, et al. Social construction of illness and disease. Community and Interculturality in Dialogue. 2023 Nov 9;3:65. https://cid.saludcyt.ar/index.php/cid/article/view/65 DOI: 10.56294/cid202365

13.	Guidos B, Calvario J, Hendrika Van Zuilen M. A brief qualitative study of indigenous traditional medicine providers in Timor-Leste. Diálogos (FFCH - UNTL). 2022;7:203–16. https://dialogosuntl.com/index.php/revista/article/view/8 DOI: 10.53930/27892182.dialogos.7.8

14.	Abbo C, Okello ES, Musisi S, Waako P, Ekblad S. Naturalistic outcome of treatment of psychosis by traditional healers in Jinja and Iganga districts, Eastern Uganda–a 3-and 6 months follow up. International Journal of Mental Health Systems. 2012;6:1–11. http://www.ijmhs.com/content/6/1/13

15.	Galvin M, Chiwaye L, Moolla A. Religious and Medical Pluralism Among Traditional Healers in Johannesburg, South Africa. J Relig Health. 2024 Apr;63(2):907–23. DOI: 10.1007/s10943-023-01795-7

16.	Galvin M, Chiwaye L, Moolla A. Perceptions of causes and treatment of mental illness among traditional health practitioners in Johannesburg, South Africa. South African Journal of Psychology. 2023;53(3):403–15. DOI: https://doi.org/10.1177/00812463231186264
