# Supplementary material for: Perspectives on health, illness, disease and management approaches among Baganda traditional spiritual healers in Central Uganda
Source: PLOS Glob Public Health. 2024 Sep 6;4(9):e0002453. doi: 10.1371/journal.pgph.0002453 (PMC11379289; doi:10.1371/journal.pgph.0002453)
Supplement: Attachment — Submitted filename: Response to reviewers.docx [file pgph.0002453.s017.docx]

**Response to reviewers**

Thank you for reviewing our manuscript and for your comments.

Below are the responses to the journal requirement and the reviewers’ comments.

**Journal Requirements**:

1. Please review your reference list to ensure that it is complete and correct. Remove some irrelevant references and replace them with relevant current references.

*Done*

Any changes to the reference list should be mentioned in the rebuttal letter that accompanies your revised manuscript.

*Done*

2. We have noticed that you have not uploaded a list of legends supporting Information. Please add a full list of legends for your Supporting Information files after the references list.

*List of legends uploaded*

**Authors,**

Avoid use of passive voice.

*Done*

the manuscript needs editing and sharpening of the language as there are some passages that are not clear. For instance (the following are illustrative but not exhaustive):

*Done*

p. 3 - The worldviews of traditional spiritual healers regarding health, illness, disease, and their management approaches are rooted in their local oral cultures and traditions, are highly stigmatized as evil and are associated with witchcraft.

*The ending of the sentence has been changed to exclude the aspects of stigmatization. It now reads.*

The worldviews of traditional spiritual healers regarding health, illness, disease, and their management approaches are rooted in their local oral cultures, traditions and are associated with witchcraft (Mwaka et al., 2023).

During colonial period, indigenous people were described as primitive and their knowledge systems rendered obsolete, irrelevant and worthless (Use of passive voice: Described as primitive by whom?)

*The sentence has been paraphrased and completed.*

During colonial period, Western civilization and European explorers described indigenous people as primitive and rendered their indigenous knowledge systems as primitive, superstitious, obsolete, irrelevant and worthless (Western Carolina University & Lefler, 2020).

p. 4 - Although, nearly 80% of the populations in developing countries use traditional medicine [11,20], it should be noted that indigenous spiritual healers and their use of local descriptive words during health management are not acknowledged [7,21]. (Again, the passive voice raises questions: Not acknowledged by who?)

*The sentence is paraphrased and put in active voice*

Although, nearly 80% of the populations in developing countries use traditional medicine [11,20], the Eurocentric science does not acknowledge indigenous spiritual healers and their use of local descriptive words during health management [7,21].

p. 4 - Unfortunately, most efforts have been directed towards understanding herbal medicine from biomedical perspectives, consequently excluding spirituality and the totality of traditional medicine from mainstream health care options (Adu-Gyamfi, 2018; Krah et al., 2018; Page-Reeves et al., 2013) (Most efforts by who?)

*The sentence is paraphrased*

Unfortunately, most efforts by African scientists have been directed towards understanding herbal medicine from biomedical perspectives, consequently excluding spirituality and the totality of traditional medicine from mainstream health care options.

p. 15 - The main findings to the research question “what words do you use to describe health, illness and disease?” were visualized beyond the bodily physical health to include nonphysical aspects of health such as the spirit, soul and good relations with spiritual, social, cultural and ancestral life. (Use of passive voice, not clear what the authors are referring to)

*The sentence is paraphrased into active voice and made clear*

The study participants visualized the “what words do you use to describe health, illness and disease?”beyond the bodily physical health to include non-physical aspects of health such as the spirit, soul and good relations with spiritual, social, cultural and ancestral life.

p. 15-16 - The discussion on disease vs. illness is not well justified in relation to the findings

*Discussion has been improved.*

Please carefully read through the text for any errors including spelling and grammar, particularly in the **introduction** and **discussion**,

*Done*

We have made efforts to minimize spelling and grammatical errors in the document

- use the passive voice only when called for
  - *Done*

We have minimized use of passive voice, instead we have used active voice

- take great care to avoid vague sentences
  - *Done*

*We have avoided vague sentences*

- make sure that readers understand who is performing the actions mentioned, or being referred to.
  - *Done*

**Changes in reference literature**

**Literature removed**

*None*

**Literature added**

1. Ayeni, O. A., Ayeni, O. O., & Jackson, R. (2007). Observations on the Procedural Aspects and Health Effects of Scarification in Sub-Saharan Africa. *Journal of Cutaneous Medicine and Surgery*, *11*(6), 217–221. https://doi.org/10.2310/7750.2007.00026
2. Henry, O. B., Omoregie, R., & Onosen Phebean Ozolua. (2020). *Prevalence and Determinants of Hepatitis C Virus Infection among HIV Infected Subjects in Benin City, Nigeria*. https://doi.org/10.5281/ZENODO.4038671
3. Okafor, I. M., Ugwu, S. O., & Okoroiwu, H. U. (2020). Hepatitis C virus infection and its associated factors among prisoners in a Nigerian prison. *BMC Gastroenterology*, *20*(1), 360. https://doi.org/10.1186/s12876-020-01504-8
4. Uzobo, E., Olomu, O. M., & Ayinmoro, A. D. (2014). The Roles of Indigenous Scarification and Body Marks in Traditional Medicine among the People of Bayelsa in the South-South Zone, Nigeria. *International Journal of Humanities and Social Science Invention*, *3*(2), 36–43. www.ijhssi.org
5. Zheng, Y., Huang, Q., Zhang, Y., Geng, L., Wang, W., Zhang, H., He, X., & Li, Q. (2023). Multimodal roles of transient receptor potential channel activation in inducing pathological tissue scarification. *Frontiers in Immunology*, *14*, 1237992. https://doi.org/10.3389/fimmu.2023.1237992
